# Meta-proteomic analysis of two mammoth’s trunks by EVA technology and high-resolution mass spectrometry for an indirect picture of their habitat and the characterization of the collagen type I, alpha-1 and alpha-2 sequence

**DOI:** 10.1007/s00726-022-03160-6

**Published:** 2022-04-17

**Authors:** Annamaria Cucina, Antonella Di Francesco, Rosaria Saletti, Maria Gaetana Giovanna Pittalà, Gleb Zilberstein, Svetlana Zilberstein, Alexei Tikhonov, Andrey G. Bublichenko, Pier Giorgio Righetti, Salvatore Foti, Vincenzo Cunsolo

**Affiliations:** 1 Laboratory of Organic Mass Spectrometry, Department of Chemical Sciences, University of Catania, Viale A. Doria 6, 95125 Catania, Italy; 2 Spectrophon Ltd, Oppenheimer 7, 7670107 Rehovot, Israel; 3 Zoological Institute, Russian Academy of Sciences, Universitetskaya nab.1, Saint-Petersburg, 199034 Russia; 4 Department of Chemistry, Materials and Chemical Engineering ‘‘Giulio Natta’’, Politecnico di Milano, Via Mancinelli 7, 20131 Milano, Italy

**Keywords:** Meta-paleoproteomics, Shotgun proteomics, Mammoth, Collagen type I, alpha-1 and alpha-2 sequence, Orbitrap fusion tribrid high-resolution mass spectrometer, Chemical modifications, Deamidation

## Abstract

The recent paleoproteomic studies, including paleo-metaproteomic analyses, improved our understanding of the dietary of ancient populations, the characterization of past human diseases, the reconstruction of the habitat of ancient species, but also provided new insights into the phylogenetic relationships between extant and extinct species. In this respect, the present work reports the results of the metaproteomic analysis performed on the middle part of a trunk, and on the portion of a trunk tip tissue of two different woolly mammoths some 30,000 years old. In particular, proteins were extracted by applying EVA (Ethylene–Vinyl Acetate studded with hydrophilic and hydrophobic resins) films to the surface of these tissues belonging to two *Mammuthus primigenus* specimens, discovered in two regions located in the Russian Far East, and then investigated via a shotgun MS-based approach. This approach allowed to obtain two interesting results: (i) an indirect description of the habitat of these two mammoths, and (ii) an improved characterization of the collagen type I, alpha-1 and alpha-2 chains (col1a1 and col1a2). Sequence characterization of the col1a1 and col1a2 highlighted some differences between *M. primigenius* and other Proboscidea together with the identification of three (two for col1a1, and one for col1a2) potentially diagnostic amino acidic mutations that could be used to reliably distinguish the *Mammuthus primigenius* with respect to the other two genera of elephantids (i.e., *Elephas and Loxodonta*), and the extinct American mastodon (i.e., *Mammut americanum*). The results were validated through the level of deamidation and other diagenetic chemical modifications of the sample peptides, which were used to discriminate the “original” endogenous peptides from contaminant ones. The data have been deposited to the ProteomeXchange with identifier < PXD029558 > .

**Supplementary Information:**

The online version contains supplementary material available at 10.1007/s00726-022-03160-6.

## Introduction

**Fig. 1:**
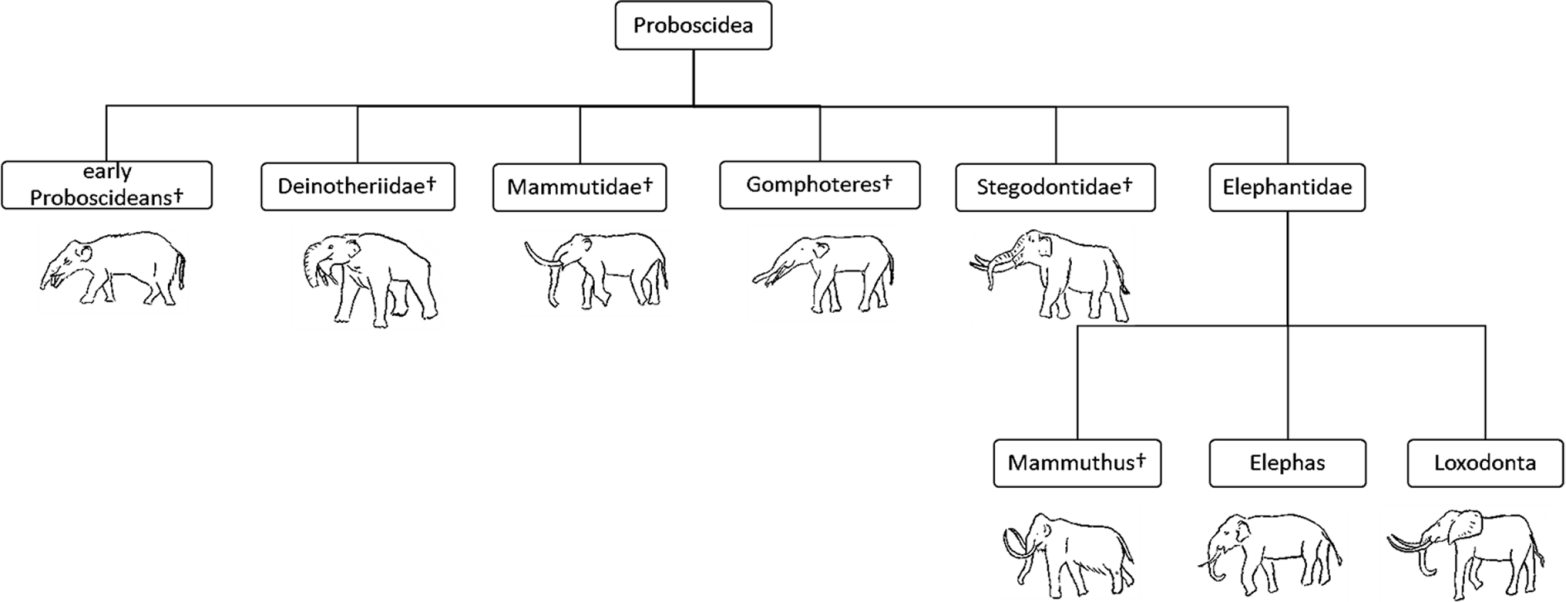
Classification of Proboscidea. †Extinct species

Paleoproteomics represents one of the recent fields which allows the characterization of past human diseases (Hendy et al. 2016; D’Amato et al. 2018), the reconstruction of the human diet (Warinner et al. 2014; Shevchenko et al. 2018; Greco et al. 2018; Tanasi et al. 2021), and helps us to improve our understanding of the phylogenetic relationships of extant and extinct species (Cappellini et al. 2014, 2018; Welker et al. 2015). In this respect, collagen has extraordinary longevity compared to other proteins (Buckley and Collins, 2011; Rybczynski et al. 2013) and, therefore, represents an ideal system for sequencing and investigating phylogeny (Buckley et al. 2011, 2019, Cappellini et al. 2011). In fact, collagen sequence information can give a valuable support to assess species relationships, especially when analysing samples from places not advantageous for genome preservation. This was the case, for example, of the assessment of extant and extinct sloths’ relationship (Presslee et al. 2019), the resolution of the evolutionary history of South America ungulates (Welker et al. 2011), and the reconstruction of extinct and extant camels’ correlation (Buckley et al. 2019). However, it is important to consider the limitations in phylogenetic constructions based on partial sequence data (Buckley et al. 2019). Proboscidea order (see Fig. 1) is composed of various extinct families, most of which (i.e., *Moeritherium)* are grouped as early Proboscideans. Other extinct families are Deinotheriidae, Mammutidae (i.e., *Mammut americanum*), Gomphotheriidae, and Stegodontidae (Shoshani 1998). The Elephantidae is the only extant family. Among the Elephantidae, *Elephas* and the extinct *Mammuthus* are recognized as belonging to the same monophyletic clade at morphological level. *Loxodonta* instead represents a sister group (Shoshani and Tassy 2005). Molecular analyses, and in particular DNA sequencing, support this reconstruction (Rohland et al. 2007; Miller et al. 2008). On the other hand, proteomic studies may help to better understand these relationships but also allow to obtain additional information. Several works about the sequencing of ancient mammoth and mastodon collagen peptides extracted from bones have already been published (Schweitzer et al. 2002; Asara et al. 2007; Buckley et al. 2011). On the contrary, although collagen preservation in soft tissues has been known for a long time (Goodman et al. 1980), a comprehensive collagen sequence characterization in this kind of material has not yet been reported.

As well known, paleoproteomic studies can be significantly challenging due to contaminations, which may mislead the characterization of protein composition or affect the detection of the ancient proteins mainly consisting of short and altered peptide fragments (Cleland et al. 2020). In fact, even if proteins are more resistant than DNA to time, it is important to highlight that diagenesis deeply affects the original protein sequences. Various precautions should be adopted to minimize the effects of contamination from modern proteins; in addition, authentication and validation criteria are needed to discriminate ancient proteins from contaminants (Hendy et al. 2018). In the last decade, ancient proteins have been studied for their patterns of degradation and diagenetic chemical modifications, and how these patterns can be used as markers for endogenous, ancient proteins as opposed to potential modern contaminants (Hill et al. 2012; Cleland et al. 2015; Cappellini et al. 2012). On this respect, it should be highlighted that recently Differential Ion Mobility Spectrometry (DMS) has proven to be a valuable tool in proteomics (Campbell et al. 2015; Winter et al. 2019), because provides separations that are orthogonal to both the MS and the LC (Campbell et al. 2012; Kafle et al. 2014), and in principle may permit a more complete understanding of the proteoforms diversity of biological systems. Although the implementation of DMS in MS workflows poses a number of challenges that must be considered, DMS represents an emerging technology that is capable of resolving multiply modified peptide variants, and should ultimately find utility in the analysis of biomolecules formed from complex biological samples, also including ancient ones. Finally, taking into account the inestimable value of archaeological samples, minimally destructive or non-destructive sampling techniques are needed. One of the more promising non-invasive techniques, known under the acronym EVA (ethylene–vinyl acetate impregnated with hydrophilic and hydrophobic resins) was introduced by Manfredi et al. 2017 and recently applied in our lab to a gut tissue sample of a woolly mammoth (i.e., the Shandrin’s mammoth) (Cucina et al. 2021). In particular, by coupling the EVA technology, a shotgun approach, and the use of high-resolution mass spectrometry it was possible to perform a meta-proteomic analysis which allowed us to get insight into the gut microbiota composition and turned out to be related to the diet of the Shandrin mammoth and its habitat.

In the present paper, we extended this approach to two other woolly mammoth’s tissues some 30,000 years old: the middle part of a trunk tissue sample, discovered in Sanga-Yuryakhsky, Yakutia, Russia (Petrova et al. 2017), and the portion of a trunk tip tissue of a mammoth discovered in the permafrost on the banks of the Bolshaya Baranikha River in the Kolyma district (Flerov 1931).

Particularly, using the same approach carried out on the Shandrin mammoth, we obtained two interesting results: (i) an indirect description of the habitat of these two mammoths; (ii) an improved characterization of the collagen type I, alpha-1 and alpha-2 chains.

## Materials and methods

### Chemicals

The chemicals employed during the analysis were of the highest purity commercially available and used without further purification. Formic Acid (FA), Ammonium bicarbonate (AMBIC), dithiothreitol (DTT), iodoacetamide (IAA) were purchased from Aldrich (St. Louis, Missouri, USA), ammonia from Carlo Erba (Milan, Italy); sequencing Grade Modified Porcine Trypsin from Promega (Madison, WI, USA); water and acetonitrile (ACN) (OPTIMA® LC/MS grade) for LC/MS analyses from Fisher Scientific (Milan, Italy). All the chemicals listed above were exclusively employed for the present study.

### The Kolyma and Sanga-Yuryahskii mammoths

The collection access number of the samples of Kolyma mammoth is 71922. The tip of a mammoth trunk was found in the middle course of the Kolyma River, on the waterside of Bolshaya Baranikha river, in 1924. Later, in 1929 a local resident of Srednekolymsk town Kondratyeva hand over trunk to geologist K. Ya. Pyatkovsky, who donated it to the Leningrad Zoological Museum. The exhibit was described by KK Flerov in 1931. The absolute age of the specimen, as well as the conditions of the finding, are not known.

The collection access number of the samples of Sanga-Yuryah mammoth is 31738/2. A large part of the skeleton, two legs, the middle part of the trunk and separate bones with soft tissues of a small female mammoth, were excavated by K. K. Vollosovich expedition in 1908 on the Sanga Yuryakh river, in the northern part of the Yano-Indigirskaya lowland. The absolute age of the specimen is 29,500 years.

### Protein sampling by EVA diskette

A special plastic-like film based on ethylene–vinyl acetate (EVA) as the binder of ground AG 501 Bio-Rad mix-bed cation/anion exchange resins was prepared as reported by Righetti et al. (2019). Protein sampling by EVA diskettes was carried out at the Zoological Museum of the Russian Academy of Sciences in St. Petersburg. For sampling large sample surfaces, it is necessary to find regions with a higher concentration of proteins. For this search, the fluorescence of phenylalanine, tyrosine, and tryptophan under UV illumination was studied.

**Fig. 2:**
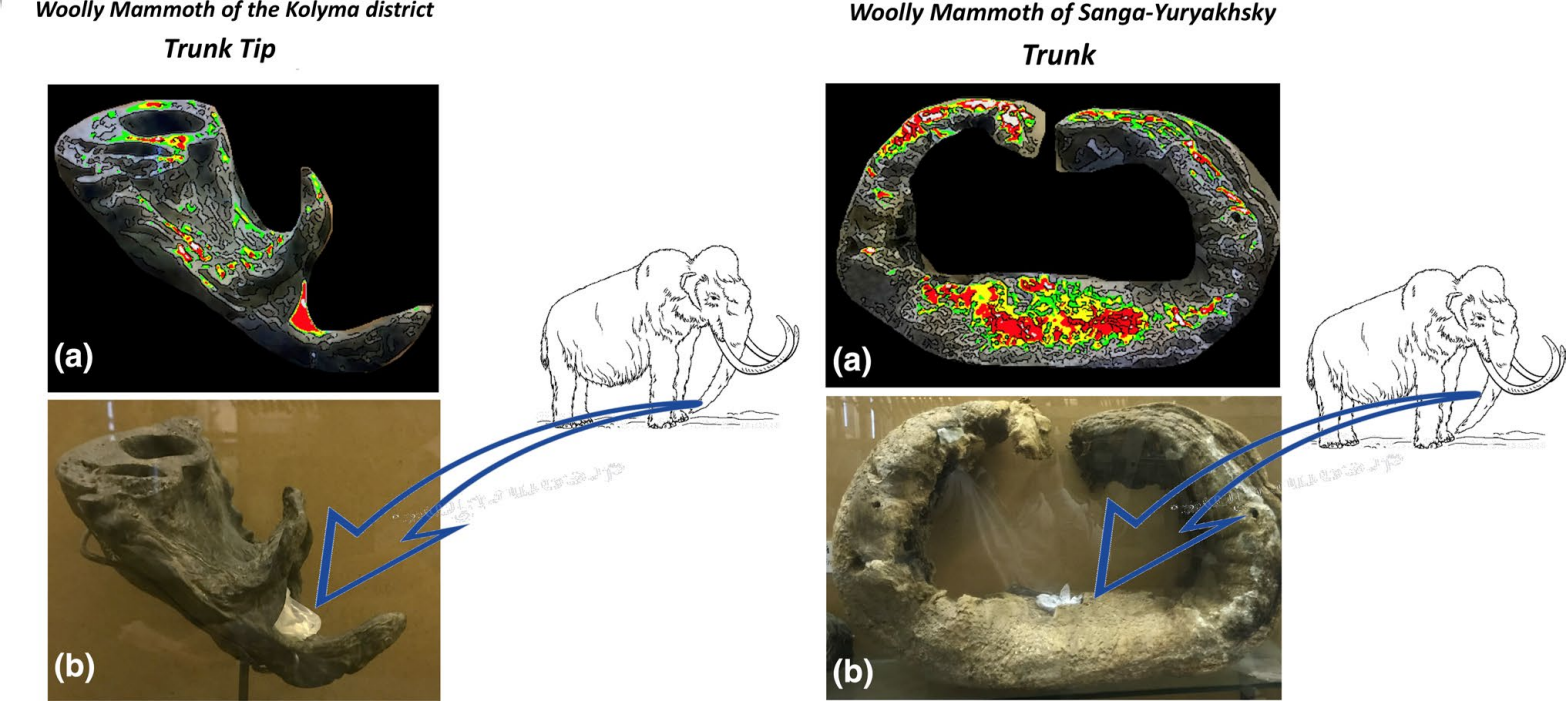
**a** Mapping of fluorescence of phenylalanine, tyrosine and tryptophan under flash UV illumination of the trunk tip of the Woolly Mammoth discovered in the Kolyma district (on the left), and from the middle part of the trunk of a female mammoth discovered in Sanga-Yuryakhsky (on the right); **b** regions of sampling by EVA diskettes

UV LED for illumination and a digital camera with a special optical filter for fluorescence detection were used. The fluorescence level at each point was displayed in pseudo colors on the instrument interface (green, yellow, and red—in order of increasing fluorescence intensity) (see Fig. 2a). This made it possible to quickly identify regions for sampling on paleontological samples. This portable system was made in SpringStyle Tech Design Ltd for quick examination of protein traces’ presence on paleontological and archaeological samples.

A SpringStyle sensor for formaldehyde (FA) residuals detection, to check the gut before sampling was applied. The selection criterion for the Museum's exhibits was the absence of formaldehyde processing of paleontological samples. Most of the samples from the paleontological collection in the 70 s of the twentieth century were treated with formaldehyde. The specimen of the mammoth trunk is one of the few exhibits that did not undergo this formaldehyde treatment.

The EVA diskette was gently humidified with ultrapure water and then placed on sample gut cavities in three regions for 60 min. To prevent drying of the EVA films, they were covered with parafilm® from the outside (Fig. b).

### Protein extraction protocol

With the aim to minimize cross-contamination with other biological samples, protein extraction and sample handling were performed in a laboratory “clean room” dedicated to ancient protein analysis and using dedicated chemicals, lab glassware, and equipment. Surfaces and equipment were washed with 50% 2-propanol before use. Non-latex gloves were used. A section of the EVA diskette (5 mm × 5 mm) was cut with a scalpel and the proteins trapped in its film were eluted sequentially with 200 μL of volatile buffers (formate at pH 3, followed by ammonia at pH 10) and finally with volatile solvents (acetonitrile) to collect positively and negatively charged as well as hydrophobic proteins. The dried eluate was suspended in 50 mM AMBIC and the proteins were quantified by a fluorimetric assay using the Qubit Protein Assay kit with the Qubit 1.0 Fluorometer (ThermoFisher Scientific, Milan, Italy) (Saletti et al. 2018).

Then, about 7 μg of protein eluate was reduced, for 3 h at room temperature, by 5.4 μg of DTT (concentration of the stock solution: 10 mM) and alkylated, 1 h in the dark at 20 °C, by 11 μg of IAA (concentration of the stock solution: 45 mM). The sample was finally digested by 0.14 μg of porcine trypsin at an enzyme–substrate ratio of 1:50 (overnight, 37 °C). The resulting peptide mixture solutions were dried under vacuum (Concentrator Plus, Eppendorf), re-dissolved in 50 µL of 5% aqueous FA, filtered by ultracentrifugation (750 µL, 0.2 µm Nonsterile Micro-Centrifugal Filters, Sepachrom, Rho, Milan), and analyzed by UHPLC/high-resolution nanoESI–MS/MS. An empty diskette of EVA film was used as a control sample. It was processed and analyzed by proteomics in the same way as the EVA diskettes placed in contact with the surface of the mammoth samples. Only a meagre number of background peptides was identified by database search in the EVA control sample (see Supplementary Material, Table S10).

### Mass spectrometry analysis

Mass spectrometry data were acquired via a Thermo Fisher Scientific Orbitrap Fusion Tribrid® (Q-OT-qIT) mass spectrometer (Thermo Fisher Scientific, Bremen, Germany). Liquid chromatography was carried out using a Thermo Scientific Dionex UltiMate 3000 RSLCnano system (Sunnyvale, CA). One microliter of peptide mixture was loaded onto an Acclaim ®Nano Trap C18 Column (100 µm i.d. × 2 cm, 5 µm particle size, 100 Å). After washing the trapping column with solvent A (H_2_O + 0.1% FA) for 3 min at a flow rate of 7 μL/min, the peptides were eluted from the trapping column onto a PepMap® RSLC C18 EASY-Spray column (75 µm i.d. × 50 cm, 2 µm particle size, 100 Å). Peptides were separated by elution at a flow rate of 0.25 µL/min at 40 °C by a linear gradient of solvent B (ACN + 0.1% FA) in A, 5% for 3 min, followed by 5% to 65% in 85 min, 65%–95% in 5 min, finishing by holding 95% B 5 min, 95%–5% in 10 min and re-equilibrating at 5% B for 15 min. The eluting peptide cations were converted to gas-phase ions by electrospray ionization using a source voltage of 1.75 kV and introduced into the mass spectrometer through a heated ion transfer tube (275 °C). Survey scans of peptide precursors from 200 to 1600 m/z were performed at 120 K resolution (@ 200 m/z). Tandem MS was performed by isolation at 1.6 Th with the quadrupole, HCD fragmentation with a normalized collision energy of 35, and rapid scan MS analysis in the ion trap (low-resolution MS/MS analysis). Only those precursors with charge state 2 ÷ 4 and intensity above the threshold of 1∙10^3^ were sampled for MS^2^. The dynamic exclusion duration was set to 60 s with a 10 ppm tolerance around the selected precursor and its isotopes. Monoisotopic precursor selection was turned on. The instrument was run in full speed mode with 3 s cycles, meaning it would continuously perform MS^2^ events until the list of non-excluded precursors diminished to zero or 3 s, whichever is shorter. MS/MS spectral quality was enhanced by enabling the parallelizable time option (i.e., using all parallelizable time during full scan detection for MS/MS precursor injection and detection). Mass spectrometer calibration was performed using the Pierce® LTQ Velos ESI Positive Ion Calibration Solution (Thermo Fisher Scientific). MS data acquisition was carried out by utilizing the Xcalibur v. 3.0.63 software (Thermo Fisher Scientific). To avoid cross-contamination with other biological samples, all solvents were prepared freshly, and ancient samples were not processed or analyzed in one batch with modern references. Moreover, to minimize carryover during nLC-MS/MS runs, from three to five blank runs were performed before each analysis using the same gradient program. Spectra acquired in the last blank run were searched by  PEAKS and MaxQuant softwares against the SwissProt database without taxonomy restrictions and using the same parameters of the archaeological samples.

### Database search for metaproteomic analysis

Mass spectrometry data were processed by MaxQuant (MQ) software 1.6.17.0 (https://www.maxquant.org/). The raw data were analyzed and searched against three different databases separately: (i) a database, including Swiss-Prot and TrEMBL sequences of *Loxodonta africana*, *Elephas maximus*, *Mammut americanum,* and *Mammuthus primigenius* (30,304 entries, February 2021) that in the text is indicated as *Proboscidea* database*;* (ii) the *Viridiplantae* database (Swiss-Prot; 40,656 entries, February 2021); and (iii) a *Bacteria* and *Nematoda* database including 334,868 and 5099 Swiss-Prot entries from bacteria and nematode, respectively (February 2021). Moreover, the common Repository of Adventitious Proteins (c-RAP; https://www.thegpm.org/crap/) contaminant database was enabled in all database searches.

The first step of database search was carried out using the following parameters: (a) tryptic peptides with a maximum of 3 missed cleavage sites; (b) cysteine carbamidomethylation as a fixed modification; (c) oxidation of methionine, the transformation of N-terminal glutamine and N-terminal glutamic acid residue to pyroglutamic acid form, oxidation of proline and the deamidation of asparagine and glutamine as variable modifications. The match type was “match from and to”. The decoy mode was “revert”. PSM, Protein, and Site decoy fraction FDR were set at 0.01 as the threshold for peptide and protein identifications. The minimum score for modified and unmodified peptides was set at 40. All the other parameters were set as default. In the data analysis, only peptides with intensity over the Max Quant threshold were considered.

Each database was investigated to identify additional chemical modifications and improve peptides identifications. All the parameters were the same as the previous step; in particular, the following chemical modifications were investigated, as variable modifications: (i) oxidation, di-oxidation, formation of kynurenine, and formation of oxo-lactone, for tryptophan residues; (ii) oxidation, di-oxidation, iodination and di-iodination, and formation of dopaquinone, for tyrosine residues; (iii) acetylation of lysine; (iv) di-oxidation of methionine; (v) tri-oxidation of cysteine. A protein was considered identified if a minimum of two peptides were matched. Finally, to be sure of the species assigned by the software to each protein identified, all the identified peptides underwent BLASTp (Basic Local Alignment Search Tool for protein) searches through the NCBI database (http://blast.ncbi.nlm.nih.gov/Blast.cgi) to validate species identifications and to rule out conserved peptides between species. The peptides in common between the three groups were not considered as valid identification either for further calculations.

### Calculation of the level of deamidation and other chemical modifications

An estimation of the percentage of deamidation for each sample was calculated with the aid of a freely available command-line script for Python 2.x (https://github.com/dblyon/deamidation), which uses the MaxQuant “evidence.txt” file (Mackie et al. 2018). The calculations were done separately for potentially original peptides and potential contaminants peptides as previously reported (Mackie et al. 2018; Tanasi et al. 2021). Analogously, estimation of the percentage of the other chemical modifications investigated was obtained applying the same model of the deamidation script, separately for potentially original and potentially contaminant peptides (See Supplementary Material).

### Metaproteomics analysis at peptide level

The metaproteomics analyses were performed by consulting the open-source web application Unipept (Unipept 4.3; http://unipept.ugent.be) (Mesuere et al. 2015), using the peptide matches with an ion score greater than 40, assigned by Max Quant to all the peptides matched, and with intensity over the Max Quant threshold.

The tool for metaproteomics analysis is realized for tryptic peptides, obtained with a shotgun approach, from environmental samples. It can calculate the Lowest Common Ancestors (LCA) of a group of peptides, giving an insight into the biodiversity of the sample, and integrating complementary functional analysis (Mesuere et al. 2018). Thanks to its algorithm, it shows the most specific taxonomic level for each peptide. Ubiquitarian peptides are generically assigned to “organism”.

### Database search for sequence characterization of collagen

With the aim to characterize collagen amino acid sequence, nLC-nESI MS/MS data were analyzed and searched against the all UniProt publicly available entries of: (i) collagen type I, alpha 1 chain (col1a1; total of 772 sequences), and (ii) alpha 2 chain (col1a2; total of 694 sequences) using integrated PEAKS X De Novo sequencing (v. 10.0, Bioinformatics Solutions Inc., Waterloo, ON Canada) and the MaxQuant software.

The amino acid sequences generated by PEAKS de novo sequencing software from each spectrum were searched using the SPIDER algorithm, a dedicated search tool of PEAKS that is specially designed to detect peptide mutations and perform cross-species homology search (Han et al. 2005). Database search was carried out using the following parameters: (i) tryptic peptides with a maximum of 3 missed cleavage sites; (ii) cysteine carbamidomethylation as a fixed modification; (iii) oxidation of methionine, oxidation of proline, the transformation of N-terminal glutamine and N-terminal glutamic acid residue to pyroglutamic acid form, and the deamidation of asparagine and glutamine as variable modifications. Database search by PEAKS was carried out using the following parameters: the precursor mass tolerance threshold was set to 15 ppm and the maximum fragment mass error was set to 0.6 Da; Peptide spectral matches (PSM) were validated using a Target Decoy PSM Validator node based on *q* values at a 1% False Discovery Rate (FDR); score thresholds for Peptide spectral matches (PSMs) were set to obtain for each database search FDR values, for PSMs, Peptide sequences, and Proteins identified, below the 1.0% value.

Database search by Max Quant software was carried out using the parameters previously described for metaproteomics analysis and an ion score greater than 50.

## Results

### Metaproteomic analysis

Metaproteomic analysis was carried out, by MQ software, investigating three different databases: *Proboscidea*, *Viridiplantae,* and *Bacteria/Nematoda.* The results were analyzed at both protein and peptide levels (i.e., not considering also the proteins from which these peptides come). In particular, all the “original peptides” identified by Max Quant were used to perform an additional analysis by Unipept search engine, which uses the UniProt database, a version of the NCBI taxonomy, and an LCA algorithm (see Material and Methods section), to achieve a global vision about the taxonomic distribution of all peptides.

### Protein identification

#### Proteins related to Mammoth

By searching the *Proboscidea* database, 35 and 14 proteins with at least 2 peptides were identified in “trunk” and “trunk tip” samples, respectively. Four proteins were identified in both the samples investigated. To validate species identifications, all the peptides which allowed the designation of these proteins were subjected to a sequence search by BLASTp. By this search, the identified proteins were classified into three groups: (i) 27 proteins that may be specifically related to mammoth; these proteins were authenticated by at least a peptide (peptide marker) related only with the *Elephantidae* species (*Loxodonta africana*, *Mammut americanum* or more in general *Elephantidae*); (ii) 14 proteins presenting peptides related to more species (*Mammalia*, *Eutheria*, *Afrotheria*, and not specific), but not coming from *Homo sapiens*; and finally (iii) 8 proteins related to *Mammalia* or not specific, but also including human. The list of proteins is reported in Table 1 (the complete list of proteins and peptides is reported in Supplementary Tables S1 and S2). As expected, most of these proteins, such as collagens, plakophilin, desmoglein, junction plakoglobin, are skin-related proteins. We also identified three keratins (i.e., keratin 32, keratin 35, and the IF rod domain-containing protein, see Supplementary Table S7) that were characterized by peptide trait sequences shared between the *Loxodonta africana* and human keratins. Therefore, also these proteins might be related to the mammoth, and considered as potentially endogenous components of the sample. Nevertheless, no *L. africana* specific peptide belonging to these keratins was recognized. Moreover, the deamidation level of the keratin-related peptides showed a similar profile to the other peptides coming from protein contaminants. Therefore, since it was not possible to establish the exact origin of these peptides, as a precaution, the above-mentioned keratins were considered contaminants.

**Table 1:** Classification of the proteins identified by searching Proboscidea database and after peptide BLAST search in “trunk” and “trunk tip” samples. More details are reported in the Supplementary Material (Tables S1 and S2)

Proteins	Taxonomy^a^	Razor + unique peptides	Chemical Modifications^b^	Trunk	Trunk tip
60S ribosomal protein L40	Not specific (including Homo sapiens)	3	–	x	x
Annexin	Not specific (including Homo sapiens)	3	pyro-E	x	
ATP synthase subunit alpha	Not specific (including Homo sapiens)	2	-	x	
CD109 molecule	Loxodonta africana	2	K^Acetyl^; De(NQ); M^ox^		x
Collagen type I alpha 1 chain (col1a1)	Loxodonta africana^c^	46	De(NQ); pyro-E; P^ox^; M^ox^	x	
Collagen type I alpha 2 chain (col1a2)	Loxodonta africana	32	P^ox^; P^Carbox^;	x	
Collagen type IV alpha 2 chain (col4a2)	Loxodonta africana	3	M^ox^; P^ox^	x	
Collagen type V alpha 1 chain (col5a1)	Mammalia (including Homo sapiens)	2	De(NQ); P^ox^	x	
Collagen type XVII alpha 1 chain (col17a1)	Loxodonta africana	2	P^ox^; M^ox^	x	
Uncharacterized protein (Acc. No. G3T4X7, similar to col2a1)	Not specific (including Homo sapiens)	2	De(NQ); P^ox^	x	
Uncharacterized protein (Acc. No.G3TH25, similar to col3a1)	Loxodonta africana	13	De(NQ); P^ox^	x	x
CTD small phosphatase-like 2	Not specific (not Homo sapiens)	2	De(NQ)	x	
Dedicator of cytokinesis 9	Elephantidae	2	De(NQ); Y^2ox^; Y^iodin^; Y^di−iodin^	x	
Desmin	Loxodonta africana	2	–	x	
Desmoglein 1	Loxodonta africana	3	M^ox^; pyro-E		x
Elastin microfibril interfacer 3	Loxodonta africana	2	M^ox^; Trp → Kyn	x	
Formin-like 1	Loxodonta africana	2	pyro-E; P^ox^	x	
Golgin B1	Mammalia (not Homo sapiens)	2	K^Acetyl^; M^2ox^; De(NQ)	x	
GTF2I repeat domain containing 1	Loxodonta africana	2	Tyr → DQ	x	
IF rod domain-containing protein	Eutheria (not Homo sapiens)	6	De(NQ); Y^iodin^	x	
IF rod domain-containing protein	Mammalia (not Homo sapiens)	2	–	x	
IF rod domain-containing protein (KRT74)	Not specific (not Homo sapiens)	2	Y^ox^	x	
Inositol-polyphosphate 5-phosphatase	Mammalia (not Homo sapiens)	3	De(NQ); Y^ox^	x	
Junction plakoglobin	Mammalia (including Homo sapiens)	9	De(NQ); P^ox^; M^ox^	x	x
Kelch-like family member 32	Loxodonta africana	2	De(NQ); W^2ox^; Trp → Kyn	x	
Matrix remodeling associated 5	Loxodonta africana	2	De(NQ); W^oxolact^; M^ox^		x
Microtubule actin crosslinking factor 1	Loxodonta africana	2	De(NQ); M^ox^		x
Myosin heavy chain 7	Mammalia (not Homo sapiens)	6	De(NQ); W^2ox^; M^ox^	x	
Nebulin	Loxodonta africana	3	Tyr → DQ, De(NQ); Y^ox^; Y^2ox^	x	
PATJ crumbs cell polarity complex component	Elephantidae	2	De(NQ); Trp → Kyn	x	
PEAK1 related, kinase-activating pseudokinase 1	Loxodonta africana	2	P^ox^; De(NQ)		x
Plakophilin 1	Not specific (including Homo sapiens)	2	–	x	
Regulatory factor X1	Loxodonta africana	2	M^ox^; P^ox^; De(NQ)		x
RING-type E3 ubiquitin transferase	Elephantidae	2	De(NQ); W^2ox^; Trp → Oxo	x	
Sacsin molecular chaperone	Loxodonta africana	2	pyro-E; De(NQ); K^Acetyl^	x	
Secreted frizzled related protein 4	Loxodonta africana	2	De(NQ)		x
SH3 domain-containing protein	Afrotheria	7	M^ox^; pyro-E	x	x
SWI/SNF related, matrix associated, actin dependent regulator of chromatin, subfamily d, member 1	Loxodonta africana	3	De(NQ)	x	
Ubiquitin carboxyl-terminal hydrolase	Mammalia (including Homo sapiens)	2	pyro-E; De(NQ); Y^ox^	x	
Uncharacterized protein (Acc. No. G3TGT5; similar to titin)	Loxodonta africana	2	M^ox^; Y^2ox^; De(NQ); Trp → Kyn; P^ox^	x	x
Uncharacterized protein (Acc. No. G3U5E2, similar to nuclear body protein SP140)	Loxodonta africana	2	M^ox^; pyro-E, De(NQ)		x
Uncharacterized protein (Acc. No. G3U5V7, similar to RUN domain-containing protein)	Loxodonta africana	2	K^Acetyl^; pyro-E; De(NQ)		x
VLIG-type G domain-containing protein	Mammalia (not Homo sapiens)	2	pyro-E; Y^2ox^	x	
Zinc finger and BTB domain containing 26	Elephantidae	2	De(NQ);, Trp → Kyn; K^Acetyl^;M^ox^	x	

^a^Taxonomy classification after the BLASTp search

^b^Chemical Modifications: pyro-E (pyro-glutamic form of glutamine or glutamic acid residues at the N-term of the peptide); P^ox^ (oxidation of proline to hydroxiproline); M^ox^ (oxidation of methionine to methionine sulfoxide); M^2ox^ (oxidation of methionine to methionine sulfone); Y^ox^ (mono-oxidation of tyrosine); Y^2ox^ (di-oxidation of tyrosine); Tyr → DQ (tyrosine oxidation to dopaquinone) De(NQ) (Deamidation of asparagine/glutamine residues); K^Acetyl^ (acetylation of lysine residue); Trp → Kyn (oxidative modification of tryptophan to kynurenine); W^2ox^ (di-oxidation of tryptophan); Trp → Oxo (tryptophan oxidation to oxolactone)

^c^Three of the peptides are razor + unique of *Mammut americanum*

### Proteins related to *Viridiplantae*, *Bacteria*, and *Nematoda*

MS data were also used to investigate separately the *Viridiplantae,* and the *Bacteria/Nematoda* databases. By searching the *Viridiplantae* database a total of eight proteins with at least two peptides (Table 2; the complete lists of proteins and peptides are reported in the Supplementary Tables S3 and S4) were identified. Four proteins were identified in the trunk, whereas the others were in the trunk tip sample. Species validation, carried out as above reported, evidenced that five proteins presented diagnostic peptides of *Mesangiospermae*, the other three showed marker peptides of *Brassicaceae, Arabidopsis thaliana*, and *Amborella trichopoda*, respectively.

**Table 2:** Classification of the proteins identified by searching *Viridiplantae* and *Bacteria/Nematoda* database and after peptide BLAST search in “trunk” and “trunk tip” samples. More details are reported in the Supplementary Material (Tables S3, S4, S5 and S6)

Proteins	Taxonomy	Razor + unique peptides	PTMs	Sample
*Viridiplantae* database				
14–3-3 protein 1	Mesangiospermae	2	M^ox^,De(NQ), Y^2ox^	Trunk
ATP synthase subunit alpha	Mesangiospermae	2	–	Trunk
DNA polymerase alpha catalytic subunit	Mesangiospermae	2	De(NQ), Trp- > Kyn, C^3ox^	Tip
DNA-directed RNA polymerase subunit beta	Mesangiospermae	2	De(NQ)	Trunk
Glyceraldehyde-3-phosphate dehydrogenase	Mesangiospermae	3	–	Tip
Histidine kinase CKI1	Brassicaceae	2	M^ox^,De(NQ)	Trunk
Protein TIC 214	Amborella trichopoda	2	De(NQ)	Tip
tRNA-specific adenosine deaminase TAD2	Arabidopsis thaliana	2	Tyr → DQ, De(NQ), M^2ox^	Tip
*Bacteria/Nematoda* database				
Cytidylate kinase	Mesoplasma florum	2	De(NQ)	tip

(a) Taxonomy classification after the BLASTp search

(b) Chemical Modifications: M^ox^ (oxidation of methionine to methionine sulfoxide); M^2ox^ (oxidation of methionine to methionine sulfone); Y^2ox^ (di-oxidation of tyrosine); Tyr → DQ (tyrosine oxidation to dopaquinone) De(NQ) (Deamidation of asparagine/glutamine residues); Trp → Kyn (oxidative modification of tryptophan to kynurenine

By the same approach, investigation of the database including only *Bacteria* and *Nematode*, allowed the identification of only one protein (i.e., the cytidylate kinase) with at least two peptides, which was specific of the *Mesoplasma florum*, a bacterium isolated from plants and insects (Table 2; the complete lists of proteins and peptides are reported in the Supplementary Tables S5 and S6).

### Unipept analysis

#### Peptides related to mammoth

**Fig. 3:**
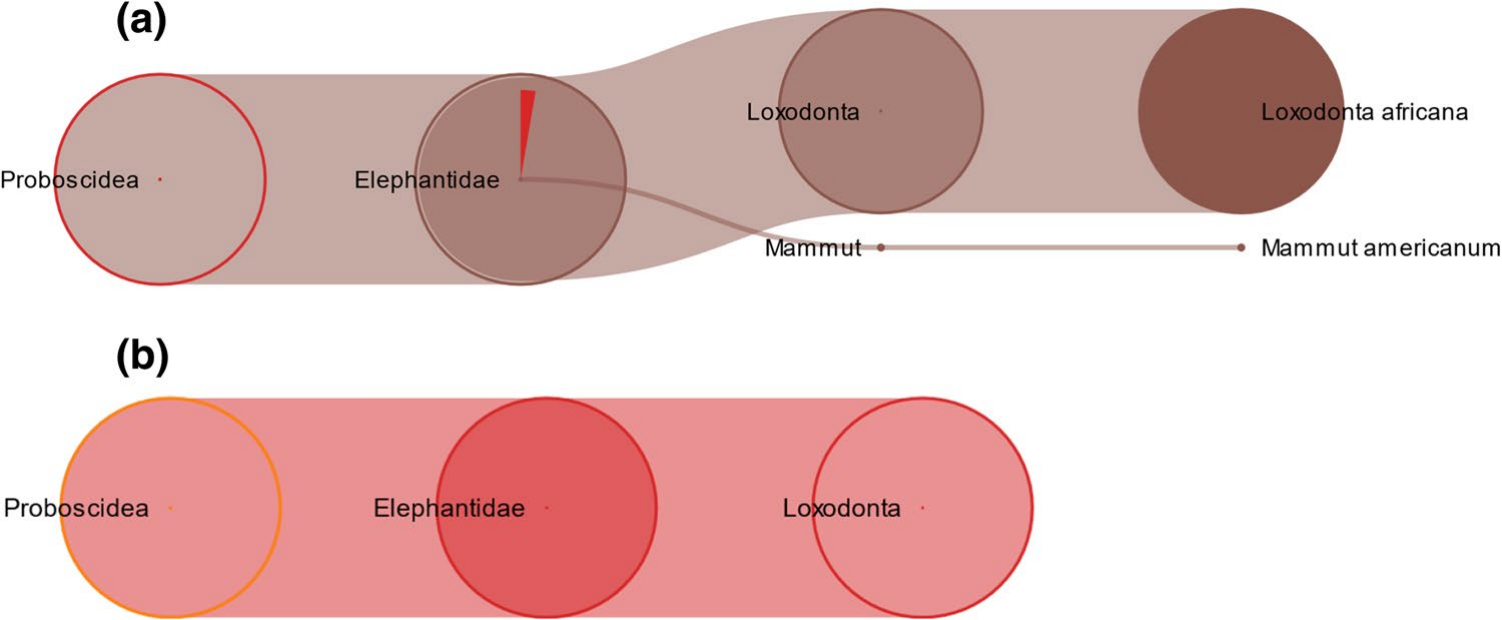
TreeView of the peptides belonging to Proboscidea in (**a**) trunk and (**b**) trunk tip sample

To achieve a comprehensive vision about the taxonomic distribution of all peptides identified by Max Quant, the Unipept search analysis was performed. In the “trunk” sample, unipept analysis of the 774 peptides characterized by searching the *Proboscidea* database allowed the classification of 651 sequences that were specific for the domain of Eukaryota. Among these sequences, 190 were specific for the family of *Elephantidae*, in particular specific of *Loxodonta africana,* and only one sequence specific of *Mammut americanum* (Fig. 3a).

In the “trunk tip” sample, unipept analysis of the 479 peptides allowed the classification of 409 sequences that were specific for the domain of Eukaryota. 158 sequences were specific for the superorder of *Afrotheria*, and included 135 sequences which instead were specific for the family of *Elephantidae* (Fig. 3b), and in particular *Loxodonta africana.*

### Peptides related to *Viridiplantae*

**Fig. 4:**
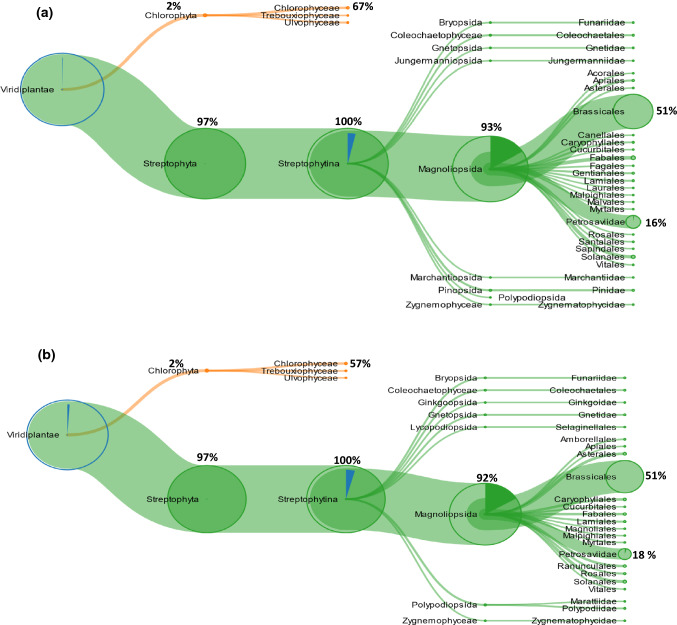
Metaproteomic analysis: tree-view of the identified peptides belonging to Viridiplantae in **a** trunk and **b** trunk tip sample. The percentage of peptides is calculated considering the 100% as the total number of peptides of the previous node

In the “trunk” sample, among the 496 peptides identified by searching the *Viridiplantae* database, 396 sequences were classified, by unipept analysis, specific for the clade *Viridiplantae*. Figure 4a shows the tree-graph results of Unipept investigation. Most of the peptides (386 sequences, 97%) were related to the clade of *Streptophyta*, whereas about 2% (9 peptides) to the clade of *Chlorophyta*. Moreover, this approach revealed that among the *Streptophyta*-related sequences, 93% (360 sequences) belong to the class of *Magnoliopsida*, and were mainly specific (185 sequences, 51%) of the *Brassicaceae* family. Among *Magnoliopsida,* the clade of *Petrosavidae* was well represented (64 sequences), but also the class of *Fabales* and *Solanales* (11 sequences) were identified.

In the “trunk tip” sample, among the 361 peptides identified by searching the *Viridiplantae* database, 295 sequences were classified, by unipept analysis, specific for the clade *Viridiplantae*. The tree-graph results of Unipept investigation, reported in Fig. 4b, show the same distribution of the peptides classified in “trunk” sample.

### Peptides related to *Bacteria* and *Nematoda*

**Fig. 5:**
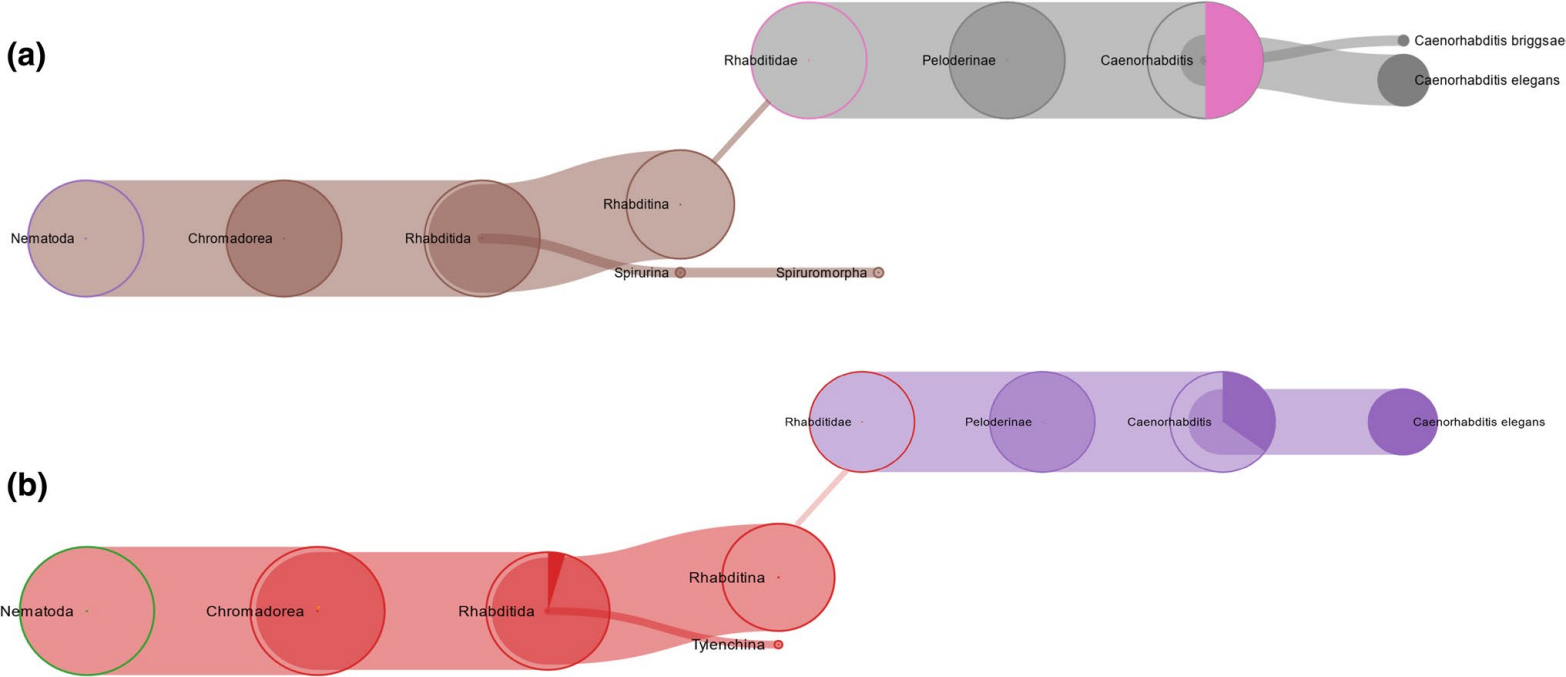
Metaproteomic analysis: tree-view of the identified peptides belonging to Nematoda in **a** trunk and **b** trunk tip sample

**Fig. 6:**
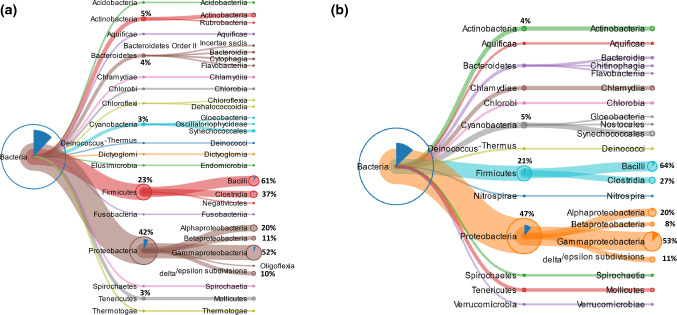
Metaproteomic analysis: tree-view of the identified peptides belonging to Bacteria in **a** trunk and **b** trunk tip sample. The percentage of peptides is calculated considering the 100% as the total number of peptides of the previous node

In the “trunk” sample, among the peptides identified by searching the Bacteria/Nematoda database, 359 were specific to Bacteria, whereas 26 sequences were specific to Nematoda phylum. All the peptides related to Nematoda were specific to Rhabditida (Fig. 5a), an order of phytoparasitic and zooparasitic microbivorous nematodes living in soil, and most of them were specific to the Caenorhabditis genus. Only one sequence was referred to Spirurina infraorder. In Bacteria (Fig. 6a) two main phyla were identified: Proteobacteria (42%; corresponding to 151 peptides), and Firmicutes (23%; corresponding to 84 peptides). The remaining peptides were related to other phyla including Actinobacteria (5%; 17 peptides), Bacteroidetes (4%; 13 peptides), Tenericutes (3%; 12 peptides), Cyanobacteria (3%; 11 peptides), and other bacteria phyla represented by a lower number of peptides.

In the “trunk tip” sample, 319 peptides were specific to Bacteria and 25 ones were specific to Nematoda phylum. The tree-graph results of Unipept investigation, reported in Fig. 5b (for Nematoda) and 6b (for Bacteria), show a similar distribution of the peptides classified in “trunk” sample.

#### Level of deamidation and other chemical modifications

To discriminate the original endogenous components, present in the investigated samples, from components that are instead probably contaminants related to the post-excavation history of the sample, we calculated the deamidation level. As known, asparagine and glutamine residues naturally deamidate over time (Robinson et al. 2001, 2002; Schroeter et al. 2016). Even if different environmental factors, such as temperature, pH, and the inherent properties of proteins, may affect the level of deamidation process, it has been observed that the level of this modification is generally higher in ancient molecules than in modern ones. Therefore, the deamidation levels of asparagine and, mainly, of glutamine residues may be used as biomolecular indicators of deterioration and the natural aging of proteins in archeology and paleo-materials.

**Fig. 7:**
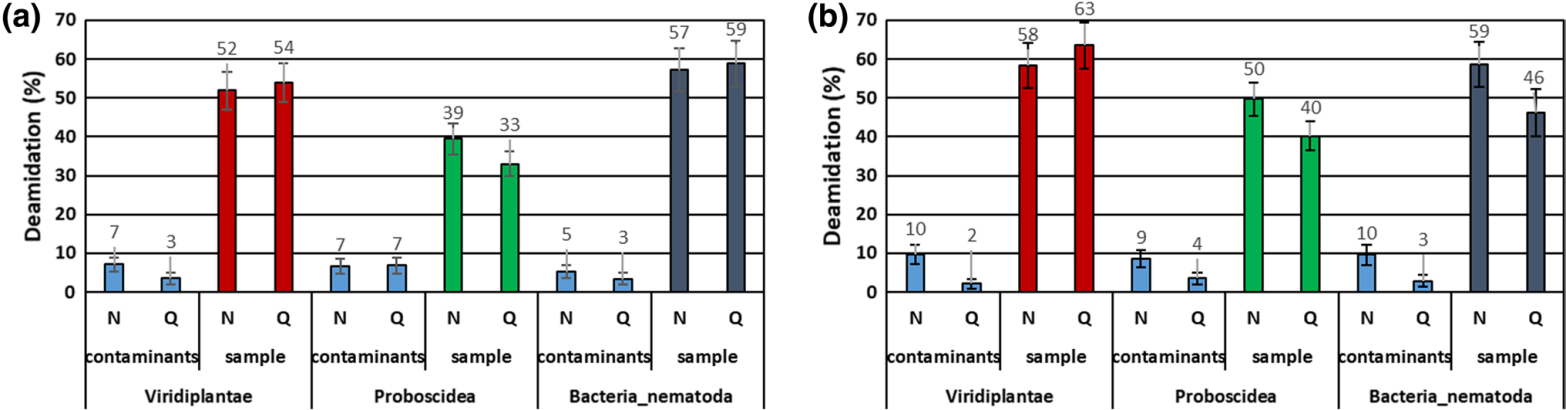
Percentage of deamidation of asparagine (N) and glutamine (Q) amino acids in **a** trunk and **b** trunk tip samples. Error bars indicate a confidence interval around 1000 bootstrap replicates

The deamidation levels of asparagine and glutamine residues were calculated for all the three types of peptides classified as “original”: i.e., peptides related to *Mammoth*, *Viridiplantae*, and *Bacteria/Nematoda*. These results were compared with the deamidation level of those peptides classified as “contaminants” (Fig. 7), because belonging to the proteins of the c-RAP database. Figure 7 shows that the deamidation level of the “original peptides” ranges from 35 to 63% in both samples investigated. On the contrary, “contaminant peptides” present a deamidation level that is always below 10%. Moreover, taking into account that other forms of spontaneous, and non-enzymatic, modifications could also be a sign of proteins damage due to the exposure to light or oxidative environmental factors (Pattinson 2012; Davies 2016), the level of the oxidation products at tryptophan, tyrosine, cysteine, and methionine residues was calculated for both the “original” and “contaminant” peptides (Stadtman et al. 2005; Pattison et al. 2012; Mikšík et al. 2016; Cannizzo et al. 2012). Interestingly, the comparison of the level of the oxidation products in original and in contaminant peptides confirms the trend already observed for the deamidation (see Supplementary Figure S1).

#### Characterization of the alpha-1 type I collagen sequence

**Fig. 8:**
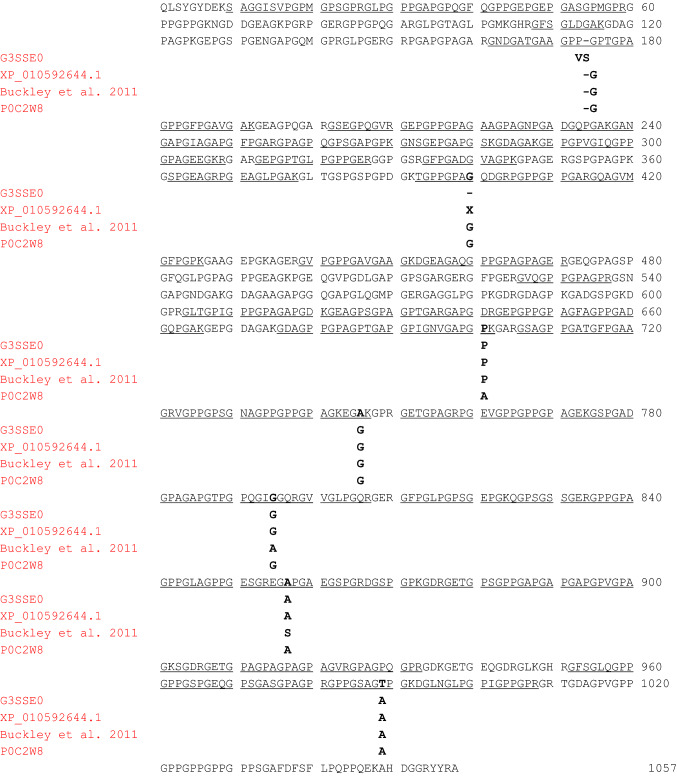
Primary structure of the col1a1 of the woolly mammoth (*Mammuthus primigenus*) identified in the protein extracts of an EVA diskette (see text). Amino acid sequence was characterized by tryptic digestion and nLC-nESI MS/MS. The sequence characterized by MS/MS data is underlined. The amino acid differences with respect to the two col1a1 unreviewed sequences of *L. africana* (UniProt Acc. No. G3SSE0, and NCBI Acc. No. XP_010592644.1), the experimentally determined Elephantidae sequences by Buckley (Buckley et al. 2011) and the reviewed sequence of *M. americanum* (UniProt Acc. No. P0C2W8), are reported in bold. The experimentally determined Elephantidae sequences by Buckley (Buckley et al. 2011) include col1a1 of a North Sea *M. primigenius, L. africana* and *E. maximus*

**Fig. 9:**
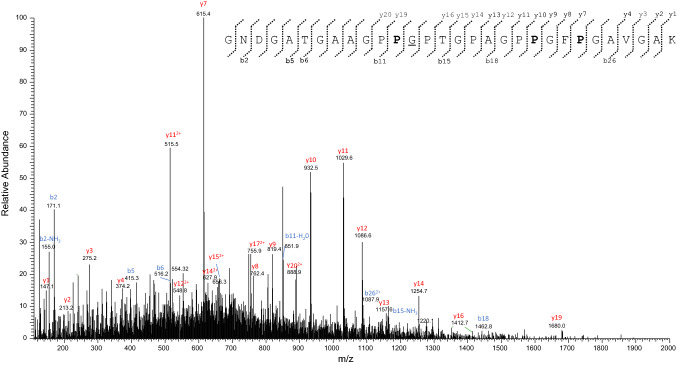
MS/MS spectrum of the triply-charged molecular ion at *m*/*z* 850.4013 (molecular mass 2548.1802) of the *Mammuthus primigenius* col1a1 tryptic peptide. The proline residues identified as hydroxyproline are reported in bold. De novo deduced sequence corresponds to the tryptic fragment Gly^162^-Lys^192^ of the col1a1 from *L. africana*, but showing the differences commented in the manuscript (see the text for details)

The dedicated database search of MS data against all the UniProt publicly available entries of col1a1 by the PEAKS X and MaxQuant software allowed the identification of a total of 40 tryptic fragments (see Supplementary Table S8). These tryptic sequences are mainly related to the col1a1 unreviewed entry (UniProt Accession No. G3SSE0; length: 1058 amino acid residues in the mature protein (Exposito et al. 2002; Buckley et al. 2011)) of the modern African elephant (*Loxodonta africana*), that, therefore, was chosen as the reference sequence. Overall, MS data allowed to confidently characterize about 65% of the *Mammuth primigenus* col1a1 primary structure (Fig. 8). Most identified sequences were identical to the reference from *L. africana*, although some differences were also observed. In particular, de novo interpretation of the MS/MS spectrum of the triply-charged ion at *m/z* 850.4013 (Fig. 9) allowed to deduce the sequence GNDGATGAAGPPGPTGPAGPPGFPGAVGAK which corresponds to the tryptic fragment G^162^NDGATGAAGPPV^174^SPTGPAGPPGFPGAVGAK^192^ of the col1a1 unreviewed entry (UniProt Accession No. G3SSE0) of *L. africana*, but carrying the deletion of the valine residue at position n. 174 and the substitution of the serine at position n. 175 with a glycine residue.

- GNDGATGAAGPP**-G**PTGPAGPPGFPGAVGAK (*Mammuth primigenus*)
- ^162^GNDGATGAAGPP**VS**PTGPAGPPGFPGAVGAK^192^ (*Loxodonta africana*)

This result was confirmed by the identification of many MS/MS scans (see Supplementary Table S8). It is important to highlight that the differences observed at positions 174 and 175 are most likely due to an error in the unreviewed entry G3SSE0_LOXAF. Indeed, it is well known that collagen molecules are made up of three alpha chains involved in the formation of a triple-helical structure (Exposito et al. 2010). At the primary structure level, the sequence of alpha chains consists of repeating Gly-Xaa-Yaa triplets, called the collagenous domain or triple helix motif. The triple helix is stabilized by the presence of glycine as every third residue, a high content of proline and hydroxyproline, interchain hydrogen bonds, and electrostatic interactions (Persikov et al. 2005), involving lysine and aspartate (Fallas et al. 2009). The presence of the VS trait in the G3SSE0_LOXAF entry would interrupt the repetition of Gly-Xaa-Yaa triplets, impairing the collagen triple helix; thus it represents a very unlikely mutation in collagen. On the other hand, a glycine residue is reported in the same position in another annotated version of the sequence of collagen alpha-1(I) chain of *Loxodonta africana* (UPI0005406912 entry; NCBI Reference Sequence: XP_010592644.1).

**Fig. 10:**
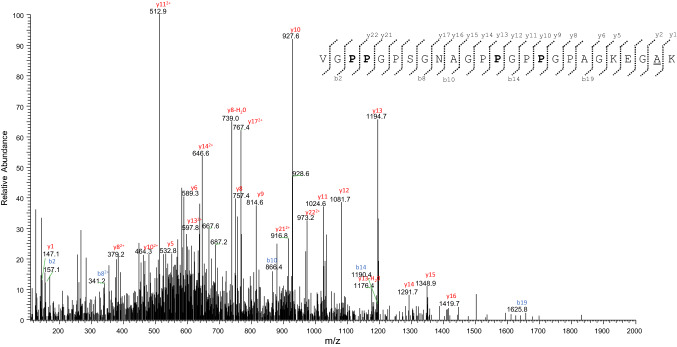
MS/MS spectrum of the triply-charged molecular ion at *m*/*z*  738.6978 (molecular mass 2213.0697) of the col1a1 *Mammuthus primigenius* tryptic peptide. The proline residues identified as hydroxyproline are reported in bold. De novo deduced sequence corresponds to the tryptic fragment Val^723^-Lys^747^ of the col1a1 from *L. africana*, but showing the substitution of the glycine at position n. 746 with an alanine residue, that is underlined (see the text for details)

**Fig. 11:**
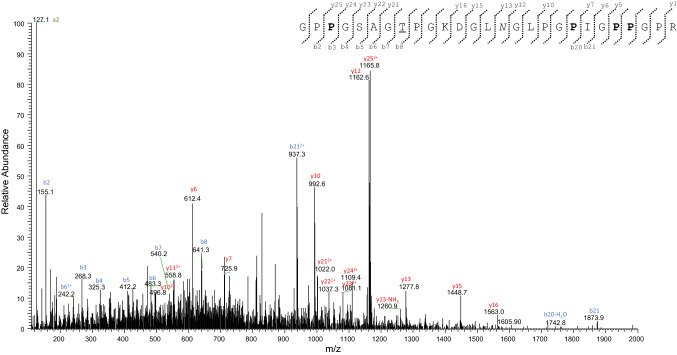
MS/MS spectrum of the triply-charged molecular ion at *m*/*z*  828.8729 (molecular mass 2483.595) of the col1a1 *Mammuthus primigenius* tryptic peptide (position 982–1008). The proline residues identified as hydroxyproline are reported in bold. The asparagine identified in deamidated form is reported in italics. De novo deduced sequence corresponds to the tryptic fragment Gly^982^-Arg^1008^ of the col1a1 from *L. africana*, but showing the substitution of the alanine at position n. 989 with a threonine residue, that is underlined (see the text for details)

Moreover, a glycine residue at position 175 was previously detected by Buckley (Buckley et al. 2011; see Fig. 8), that has characterized the col1a1 sequence extracted from the bone powder of a woolly mammoth dredged from the North Sea, a modern African elephant (*Loxodonta africana*), and an Asian elephant (*Elephas maximus*) specimens, and is also typical of the col1a1 sequence of *M. americanum* (SwissProt Accession No. P0C2W8). An additional error in the unreviewed entry G3SSE0_LOXAF was detected. The tryptic fragment T^393^GPPGPAG^400^QDGRPGPPGPPGAR^414^ (Table S8, Fig. 8) shows as expected, at position 400, the presence of a glycine residue, which instead is lacking in the G3SSE0_LOXAF entry. Again, the absence of the glycine residue at this position would interrupt the repetition of Gly-Xaa-Yaa triplets, impairing the collagen triple helix; thus it represents a mistake in the entry G3SSE0_LOXAF. De novo interpretation of the MS/MS spectra of the triply charged ions at *m/z* 738.6978 and 828.8729, allowed the identification of two amino acid substitutions in the *M. primigenius* col1a1 here investigated with respect to the *L. africana* counterpart. In detail, our experimental MS/MS data allowed to deduce the sequences VGPPGPSGNAGPPGPPGPAGKEGAK, and GPPGSAGTPGKDGLNGLPGPIGPPGPR, respectively (Figs. 10 and 11).

The first one corresponds to the sequence V^723^GPPGPSGNAGPPGPPGPAGKEGGK^747^, but shows the substitution of the glycine at position 746 with an alanine residue. The second one instead corresponds to the tryptic fragment G^982^PPGSAGAPGKDGLNGLPGPIGPPGPR^1008^, carrying the amino acid substitution Ala^989^ → Thr. It is important to highlight that comparison of the col1a1 sequences coming from different Proboscidea species (Fig. 8) reveals that the presence of an alanine residue at position 746 and a threonine at position 989 appear unique of the col1a1 sequence of the *M. primigenus* here investigated, and, therefore, could be used to reliably distinguish the *Mammuthus primigenius* with respect to the other two genera of elephantids (i.e., *Elephas and Loxodonta*), and the extinct American mastodon (i.e., *Mammut americanum*).

Furthermore, MS data here obtained allowed to detect the presence of a proline residue at position 701, which appears characteristic of Elephantidae, and could be used as marker to distinguish them from the *M. americanum*, which instead has an alanine (Fig. 8). Finally, our MS data show that the primary structure of the *M. primigenius* col1a1 sequence here investigated has a glycine and an alanine at positions 795 and 857, respectively (Fig. 8). These two positions are shared with the unreviewed col1a1 entry of *L. africana*, and the reviewed entry counterpart of *M. americanum*. On the contrary, the Elephantidae (i.e., *M. primigenius, L. africana* and *E. maximus*) col1a1 sequences reported by Buckley (Buckley et al., 2011) show the presence of an alanine (at position 795) and a serine (at position 857).

#### Charaterization of the alpha-2 type I collagen sequence

**Fig. 12:**
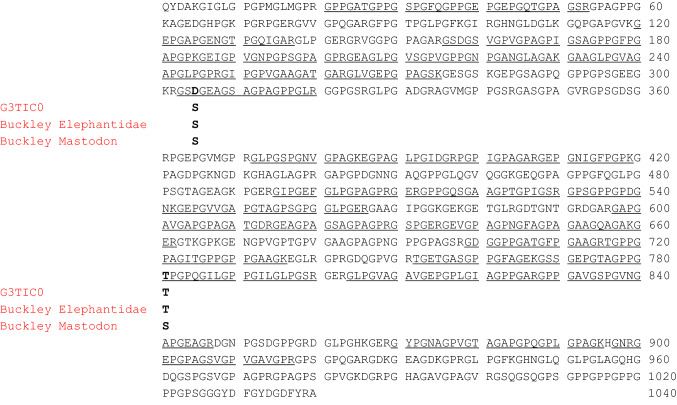
Primary structure of the col1a2 of the woolly mammoth (*Mammuthus primigenus*) identified in the protein extracts of an EVA diskette (see text). Amino acid sequence was characterized by tryptic digestion and nLC-nESI MS/MS and using the known mature form of the unreviewed col1a2 from *Loxodonta africana* (UniProt Acc. No. G3TIC0) as reference. The sequence characterized by MS/MS data is underlined. The amino acid differences with respect to the col1a2 unreviewed sequence of *L. africana*, the experimentally determined Elephantidae sequences by Buckley et al. 2011 and the experimentally determined sequences of *M. Americanum* by Buckley et al. 2011 are reported in bold. The experimentally determined Elephantidae sequences by Buckley et al. (Buckley et al., 2011) include col1a2 of a North Sea *M. primigenius, L. africana* and *E. maximus*

**Fig. 13:**
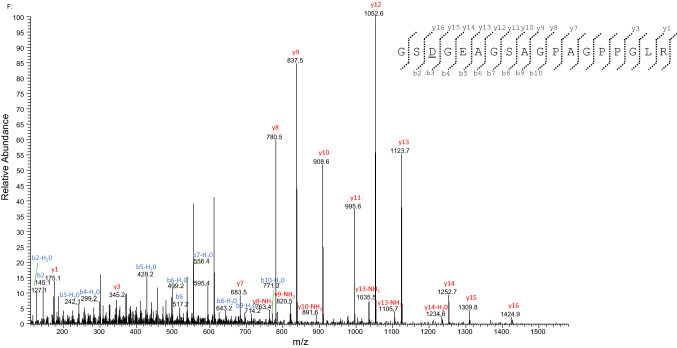
MS/MS spectrum of the double charged molecular ion at *m*/*z*  784.8686 (molecular mass 1567.7214) of *Mammuthus primigenius* col1a2 tryptic peptide. De novo deduced sequence corresponds to the tryptic fragment Gly^303^- Arg^320^ of the unreviewed col1a2 from *Loxodonta africana* (UniProt Acc. No. G3TIC0) but carrying the substitution of the serine at position n. 305 with an aspartic acid residue, that is underlined (see the text for details)

The dedicated database search of MS data against all the UniProt publicly available entries of alpha-2 type I collagen (col1a2) by the PEAKS X and MaxQuant softwares allowed the identification of a total of 31 tryptic fragments (see Supplementary Table S9). These tryptic sequences are mainly related to the col1a2 unreviewed entry (UniProt Accession No. G3TIC0; length: 1040 amino acid residues in the mature protein) of the modern African elephant (*Loxodonta africana*), that thus was selected as reference sequence. Overall, MS data allowed to confidently characterize about 50% of the *Mammuth primigenus* col1a2 primary structure (Fig. 12). The characterized sequence was identical to the reference from *L. africana*, except for an amino acid point difference. In particular, de novo interpretation of the MS/MS spectrum of the double-charged ion at *m/z* 784.8686 (Fig. 13) allowed to deduce the sequence GSDGEAGSAGPAGPPGLR which corresponds to the tryptic fragment G^303^SS^305^GEAGSAGPAGPPGLR^320^ of *L. africana*, but carrying a substitution of the serine residue at position n. 305 with an aspartic acid. This substitution is supported by the presence of the MS/MS spectrum of the triple-charged ion at *m/z* 575.6152, corresponding to the sequence RGSDGEAGSAGPAGPPGLR (see Supplementary Table S9). However, although the corresponding amino acid trait with the asparagine at position 305 wasn’t detected, the presence of a deamidated asparagine instead of the aspartic acid, at this position, cannot be excluded. It is interesting to note that the col1a2 sequences of *Elephantidae* (i.e., *M. primigenius*, *L. africana*, and *E. maximus*) and of *M. americanum*, as reported by Buckley (Buckley et al. 2011), show a serine residue at position n. 305. Thus the presence of a different amino acid residue at this position could be used as marker to distinguish the Siberian *Mammuthus primigenius* with respect to the other Proboscidea species.

Furthermore, as previously suggested by Buckley (Buckley et al. 2011), MS data here obtained allowed to detect the presence of a threonine residue at position 781, which seems peculiar of Elephantidae, and could be used as a marker to distinguish them from the *M. americanum*, which instead has a serine (Fig. 12).

## Discussion

### Metaproteomic analysis

Metaproteomics analysis of two well-preserved mammoth remains was carried out. In particular, we investigated a mammoth trunk tip, found in 1924 in the permafrost soil on the banks of the Bolshaya Baranikha River in the Kolyma district, and a trunk fragment of a female mammoth discovered in Sanga-Yuryakhsky (Yakutia, Russia), dated as 29,500 years, on the basis of radiocarbon analyses. The structure of the bilobed trunk tip of the mammoth, discovered in the Kolyma district, suggested that it could pluck large bunches of grass and moss much easier than the Indian and African elephants. Probably, the mammoth ate mainly arboreal vegetation during winter, and herbaceous plants during summer (Boeskorov et al. 2016). Paleoecological conditions in the Kolyma region have been studied via the pollen spectra analysis (PSA) of a ground sample that adhered to a rhinoceros corpse and two samples from enclosing sediments. The composition of the pollen was dominated by grasses and shrubs; the taxonomic distribution presented mainly Gramineae (i.e., Poaceae), Asteraceae (wormwoods) and Cyperaceae, fewer Caryophillaceae, and Ranunculaceae. Other taxa observed were Fabaceae, Brassicaceae, Lamiales and other families of plants. Tree-shrub vegetation was highlighted by the presence of pollen of dwarf birches, willows, alder and larch. A similar environment could be attributed to the Yakutia mammoth. In fact, also the trunk fragment of this female mammoth was discovered in the proximity of a palustris area in East Siberia (Tolmachoff 1929).

Most of the results of our metaproteomic analysis for both samples agree with the previous paleobotanical studies, with a considerable identification of Graminaceae (Petrosavidae) peptides, followed by the other families mentioned above. An exception concerns the high percentage of Brassicaceae peptides, although this result could be reasonably related to the predominance of *Arabidopsis thaliana* entries in the *Viridiplantae* database. Furthermore, the identification of Chlorophyta species is probably due to the presence of a river in the proximity of the mammoth remains. Interestingly, an *Amborella trichopoda* protein (i.e., the protein TIC 214) was identified in the trunk tip sample. This plant is considered one of the ancestral angiosperms, and it is typical of a pluvial forest. Altogether, these results supported the hypothesis of a different North Siberia habitat during the Late Pleistocene. Moreover, the composition of microorganism-related peptides found in the trunks surface reflected that observed in the Shandrin mammoth gut (Cucina et al. 2021), with a predominance of Proteobacteria, followed by Firmicutes, probably attributed to the presence of grass more than tree shrub. Interestingly, in the trunks surface the presence of Firmicutes, and particularly of Bacilli, seemed to be higher. According to Omeliansky, who in 1908 carried out bacteriological research on the “Sanga-Yurach”, during the late Pleistocene quite a diverse flora of microorganisms, different kinds of bacteria, mold, and yeast existed, while in the modern trunk, mucosal microflora presented mainly a variety of aporose bacilli (Neustroev et al. 2014). Thus, it is complicated to establish if the presence of these bacteria should be attributed to a posteriori colonization, or if it reflects the presence of ancient microorganisms very similar to the bacilli that colonize the trunk microflora of the modern Proboscidea.

Finally, our metaproteomic analysis revealed that most of the peptides identified in Nematoda database belong to Caenorhabditis, a genus of nematodes that lives in bacteria-rich environments, such as compost piles, decaying dead animals and rotting fruit (Frézal and Félix 2015).

#### Collagen type I, alpha-1 and alpha-2 chains

By coupling the non-invasive EVA technology and a shotgun proteomic approach based on the high-resolution mass spectrometry, we were able to characterize about 65% of the col1a1 and 50% of the col1a2 sequences of the Siberian woolly mammoth (*Mammuthus primigenius*), including some amino acid traits of the protein never characterized before. At least for the part of the covered sequence, it appears that the col1a1 and the col1a2 of the wolly mammoth and the unreviewed col1a1 and col1a2 entries of *Loxodonta africana* reported in the databases (G3SSE0_LOXAF, XP_010592644.1, and G3TICO_LOXAF), are almost identical, showing only two amino acid substitutions for col1a1 and one amino acid substitution for col1a2. Our data, obtained at protein level, confirm the sequencing of the nuclear genome which suggests a difference at amino acid level of about 0.22% among Elephantids (Miller et al. 2008). More in detail, our data highlighted some errors in the unreviewed entry G3SSE0_LOXAF of col1a1 at positions 174, 175, and 400 that would interrupt the repetition of Gly-Xaa-Yaa triplets, impairing the collagen triple helix; thus they represent a very unlikely mutation in collagen.

Instead, the three observed substitutions (i.e., Gly^746^ → Ala, and Ala^989^ → Thr for col1a1 and Ser^305^ → Asp for col1a2) seem more interesting, because appear unique of the col1a1 and col1a2 sequences of the *M. primigenus* here investigated, and, therefore, could be used to reliably distinguish the Siberian mammoth with respect to the other two genera of elephantids (i.e., *Elephas* and *Loxodonta*), and the extinct American mastodon (i.e., *Mammut americanum*). However, the limited extent of this analysis may prevent a definitive conclusion, and a larger number of samples together with a combination of different digestion approaches might implement and support these identifications.

## Conclusions

Shotgun proteomic analysis of mammoth trunk samples carried out by coupling the EVA film technology and high-resolution mass spectrometry allowed in-depth exploration of the metaproteome composition together with an improved characterization of the primary structure of the alpha-1 and alpha-2 chains of collagen type I.

Metaproteomic analysis allowed to identify proteins specific to the tissue investigated, but also some plant proteins related to the animal environment. Moreover, taxonomic distribution of all the identified peptides evidenced the predominance of some specific taxonomies among *Viridiplantae, Bacteria*, and *Nematoda* which are in agreement with the reconstructed habitat of Late Pleistocene Siberia. It should be noted that our results represent an indirect picture of the proteins present in the samples, because the intrinsic nature of the surface sampling of EVA technology. Thus the information obtained might not be fully exhaustive. However, it should be also highlighted that the use of EVA diskettes avoids the destruction of part of a sample of ancient tissue, a practice which is (obviously) discouraged by most museums.

Concerning the characterization of the collagen type I, alpha-1 and alpha-2 sequences, our MS data allowed to improve the previous sequence coverage obtained from the bone powder of a woolly mammoth dredged from the North Sea, as reported by Buckley (Buckley et al. 2011). In addition, we detected some differences between *M. primigenius* and other Proboscidea, and identified three potentially diagnostic amino acid mutations that could be used to reliably distinguish the *Mammuthus primigenius* with respect to the other two genera of elephantids (i.e., *Elephas* and *Loxodonta*), and the extinct American mastodon (i.e., *Mammut americanum*).

In conclusion, it is important to evidence, in paleoproteomics, the importance of authentication of ancient environmental proteins and peptides, particularly for open systems, such as skin samples. Overall, the approach here employed, including the authentication method based on a deep analysis of peptides modifications and damage, highlight the importance of this kind of investigation, which could integrate and support the classical paleoclimatology studies, but also improve our understanding of the phylogenetic relationships between extant and extinct species.

## Supplementary Information

Below is the link to the electronic supplementary material.

Supplementary file1 (DOCX 1518 kb)
