# Supplementary material for: Meta-proteomic analysis of two mammoth’s trunks by EVA technology and high-resolution mass spectrometry for an indirect picture of their habitat and the characterization of the collagen type I, alpha-1 and alpha-2 sequence
Source: Amino Acids. 2022 Apr 17;54(6):935–54. doi: 10.1007/s00726-022-03160-6 (PMC9213349; doi:10.1007/s00726-022-03160-6)
Supplement: Supplementary file 1 — Supplementary file1 (DOCX 1518 kb) [file 726_2022_3160_MOESM1_ESM.docx]

***Proteins and Peptides Identification***

The results of the research with the different chemical modifications were combined and the complete lists of the identified peptides and proteins are reported in the Supplementary Tables S1-S6. Also proteins identified with only one peptide are listed.

In detail:

Tables S1 and S2 show the list of *Proboscidea* proteins with the corresponding identified peptides in trunk and trunk tip samples, respectively.

Tables S3 and S4 relate the complete list of peptides and proteins identified from *Viridiplantae* database in trunk and trunk tip samples, respectively.

Tables S5 and S6 report the complete list of peptides and proteins of *Bacteria/Nematoda* detected in trunk and trunk tip samples, respectively.

Table S7 lists of keratins identified by peptides shared between mammoth and human.

Table S8 presents the list of the identified col1a1 peptides in trunk sample.

Table S9 presents the list of the identified col1a2 peptides in trunk sample.

Table S10 details information about the EVA diskette blank.

Figure S1 shows the level of oxidative products for endogenous “potentially original” (*Proboscidea, Viridiplantae, and Bacteria/Nematode*) and C-Rap peptides (i.e. the “potentially contaminant” peptides).

Table S1. List of peptides and proteins of Proboscidea in trunk sample: for each peptide all the features (same sequence and different modifications) with the highest intensity are reported.

| Acc. No | Description (*Organism*) | Peptide Sequence | Modifications | Charge | Mass | Mass error [ppm] | MaxQuant Intensity | Score |
| --- | --- | --- | --- | --- | --- | --- | --- | --- |
| G3SSE0 | Collagen type I alpha 1 chain  *(Loxodonta africana)* | DGLNGLPGPIGPPGPR | Deamidation (NQ),3 Oxidation (P) | 2 | 1561.77 | 1.70 | 10514000 | 139 |
|  |  |  | 3 Oxidation (P) | 2 | 1560.79 | 2.00 | 1665800 | 119 |
|  |  | GDAGAKGEPGPVGIQGPPGPAGEEGKR | 2 Oxidation (P) | 4 | 2516.23 | -0.03 | 791610 | 54 |
|  |  | GDAGPPGPAGPTGAPGPIGNVGAPGPK | 2 Oxidation (P) | 3 | 2294.13 | 2.56 | 2860300 | 61 |
|  |  |  | 3 Oxidation (P) | 3 | 2310.12 | 1.57 | 5494800 | 78 |
|  |  | GEPGPVGIQGPPGPAGEEGK | 2 Oxidation (P) | 2 | 1860.89 | -0.13 | 1216700 | 80 |
|  |  |  | Oxidation (P) | 2 | 1844.89 | 0.81 | 995220 | 65 |
|  |  | GEPGPVGIQGPPGPAGEEGKR | 2 Oxidation (P) | 3 | 2016.99 | -0.01 | 4139800 | 111 |
|  |  |  | Deamidation (NQ),2 Oxidation (P) | 3 | 2017.97 | 0.42 | 813390 | 81 |
|  |  | GPPGSAGAPGKDGLNGLPGPIGPPGPR | Deamidation (NQ),4 Oxidation (P) | 3 | 2454.21 | 0.90 | 19035000 | 92 |
|  |  | DGEAGAQGPPGPAGPAGER | Unmodified | 2 | 1689.77 | -0.17 | 9793200 | 184 |
|  |  |  | Oxidation (P) | 2 | 1705.77 | -0.44 | 8071800 | 209 |
|  |  |  | Deamidation (NQ) | 2 | 1690.75 | -0.04 | 2381000 | 101 |
|  |  |  | Deamidation (NQ),Oxidation (P) | 2 | 1706.75 | -0.71 | 1789700 | 167 |
|  |  | DGSPGPKGDRGETGPSGPPGAPGAPGAPGPVGPAGK | 5 Oxidation (P) | 4 | 3182.49 | 0.38 | 991000 | 56 |
|  |  | GANGAPGIAGAPGFPGAR | Deamidation (NQ),3 Oxidation (P) | 2 | 1585.75 | 0.63 | 72510000 | 144 |
|  |  | GAPGDRGEPGPPGPAGFAGPPGADGQPGAK | 4 Oxidation (P) | 3 | 2702.23 | 0.05 | 14964000 | 53 |
|  |  |  | Deamidation (NQ),4 Oxidation (P) | 3 | 2703.22 | 1.10 | 5541600 | 86 |
|  |  |  | 3 Oxidation (P) | 3 | 2686.24 | 0.06 | 5011200 | 92 |
|  |  |  | 5 Oxidation (P) | 3 | 2718.23 | -0.92 | 3976400 | 69 |
|  |  | GDAGAPGAPGSQGAPGLQGMPGER | Oxidation (M),4 Oxidation (P) | 2 | 2213.96 | -0.53 | 5016100 | 68 |
|  |  | GDAGPAGPKGEPGSPGENGAPGQMGPR | Deamidation (NQ),4 Oxidation (P) | 3 | 2509.08 | -1.29 | 23397000 | 103 |
|  |  |  | Oxidation (M),Deamidation (NQ),2 Oxidation (P) | 3 | 2493.08 | 0.39 | 2421000 | 63 |
|  |  | GDRGETGPSGPPGAPGAPGAPGPVGPAGK | 4 Oxidation (P) | 3 | 2528.19 | -0.29 | 3484600 | 142 |
|  |  |  | 3 Oxidation (P) | 3 | 2512.19 | 0.01 | 2728600 | 61 |
|  |  |  | 2 Oxidation (P) | 3 | 2496.20 | 0.48 | 1059900 | 41 |
|  |  | GEPGPPGPAGAAGPAGNPGADGQPGAK | 4 Oxidation (P) | 3 | 2315.04 | 2.12 | 3781600 | 81 |
|  |  |  | Deamidation (NQ),3 Oxidation (P) | 3 | 2300.03 | 0.90 | 3340400 | 78 |
|  |  |  | 3 Oxidation (P) | 3 | 2299.05 | 0.80 | 2701900 | 59 |
|  |  |  | Deamidation (NQ),4 Oxidation (P) | 3 | 2316.03 | -0.04 | 2537500 | 75 |
|  |  | GEPGPPGPAGFAGPPGADGQPGAK | 3 Oxidation (P) | 3 | 2132.98 | 0.46 | 1053800 | 53 |
|  |  | GEPGPTGLPGPPGER | 2 Oxidation (P) | 2 | 1448.69 | 0.04 | 92221000 | 109 |
|  |  |  | 3 Oxidation (P) | 2 | 1464.68 | -0.06 | 73675000 | 88 |
|  |  |  | 4 Oxidation (P) | 2 | 1480.68 | -0.85 | 2286000 | 88 |
|  |  | GEPGSPGENGAPGQMGPR | Oxidation (M),2 Deamidation (NQ),2 Oxidation (P) | 2 | 1743.70 | -0.62 | 2530000 | 107 |
|  |  |  | Oxidation (M),Deamidation (NQ),Oxidation (P) | 2 | 1726.72 | 0.81 | 786860 | 93 |
|  |  | GETGPAGPAGPAGPAGVR | Unmodified | 2 | 1517.76 | 0.47 | 57437000 | 145 |
|  |  |  | Oxidation (P) | 2 | 1533.75 | 1.31 | 789400 | 69 |
|  |  | GETGPAGRPGEVGPPGPPGPAGEK | 3 Oxidation (P) | 3 | 2215.05 | -0.68 | 28991000 | 79 |
|  |  |  | 2 Oxidation (P) | 3 | 2199.06 | -0.29 | 9089900 | 115 |
|  |  |  | 4 Oxidation (P) | 3 | 2231.05 | -0.97 | 6429700 | 60 |
|  |  |  | 5 Oxidation (P) | 3 | 2247.04 | 0.80 | 1317700 | 55 |
|  |  | GETGPSGPPGAPGAPGAPGPVGPAGK | 4 Oxidation (P) | 2 | 2200.04 | -0.31 | 1271500 | 123 |
|  |  |  | 3 Oxidation (P) | 2 | 2184.04 | -0.67 | 1135700 | 90 |
|  |  | GFSGLQGPPGPPGSPGEQGPSGASGPAGPR | 3 Oxidation (P) | 3 | 2704.25 | 0.74 | 16042000 | 106 |
|  |  |  | Deamidation (NQ),3 Oxidation (P) | 3 | 2705.23 | -0.69 | 8641200 | 92 |
|  |  |  | 4 Oxidation (P) | 3 | 2720.24 | 0.34 | 7981300 | 58 |
|  |  |  | 2 Oxidation (P) | 3 | 2688.25 | -0.57 | 6671700 | 61 |
|  |  |  | Deamidation (NQ),2 Oxidation (P) | 3 | 2689.24 | -0.14 | 4588700 | 77 |
|  |  |  | Deamidation (NQ),4 Oxidation (P) | 3 | 2721.23 | 0.31 | 3578900 | 70 |
|  |  |  | Oxidation (P) | 3 | 2672.26 | -0.36 | 1589900 | 66 |
|  |  |  | 2 Deamidation (NQ),3 Oxidation (P) | 3 | 2706.22 | -0.84 | 1394100 | 71 |
|  |  | GLPGPPGAPGPQGFQGPPGEPGEPGASGPMGPR | 5 Oxidation (P) | 3 | 3083.40 | -0.22 | 8479200 | 59 |
|  |  |  | 4 Oxidation (P) | 3 | 3067.41 | -0.88 | 8372600 | 63 |
|  |  |  | 3 Oxidation (P) | 3 | 3051.41 | -0.49 | 3623300 | 42 |
|  |  | GLTGPIGPPGPAGAPGDK | Oxidation (P) | 2 | 1573.81 | 0.13 | 2998300 | 64 |
|  |  |  | 2 Oxidation (P) | 2 | 1589.80 | 0.68 | 620720 | 100 |
|  |  | GLTGPIGPPGPAGAPGDKGEAGPSGPAGPTGAR | 2 Oxidation (P) | 3 | 2852.41 | -0.43 | 70249000 | 88 |
|  |  |  | Oxidation (P) | 3 | 2836.41 | -0.09 | 34055000 | 121 |
|  |  |  | 3 Oxidation (P) | 3 | 2868.40 | -0.23 | 10374000 | 52 |
|  |  |  | 4 Oxidation (P) | 3 | 2884.40 | -0.50 | 1838100 | 53 |
|  |  | GPAGPQGPR | Unmodified | 2 | 835.43 | -0.03 | 2082200 | 101 |
|  |  | GPAGPQGPSGAPGPK | Deamidation (NQ),Oxidation (P) | 2 | 1290.62 | 0.27 | 52826000 | 206 |
|  |  |  | Unmodified | 2 | 1273.64 | 0.77 | 672300 | 139 |
|  |  |  | Deamidation (NQ) | 2 | 1274.63 | 0.85 | 509090 | 109 |
|  |  | GPPGPAGPPGLAGPPGESGR | 2 Oxidation (P) | 2 | 1755.85 | -0.65 | 62387000 | 109 |
|  |  |  | 3 Oxidation (P) | 2 | 1771.85 | 1.33 | 40395000 | 323 |
|  |  |  | Oxidation (P) | 2 | 1739.86 | 0.25 | 10252000 | 121 |
|  |  |  | 4 Oxidation (P) | 2 | 1787.84 | 1.20 | 2250500 | 63 |
|  |  |  | Unmodified | 2 | 1723.86 | 0.26 | 1621600 | 83 |
|  |  | GPPGPAGPPGLAGPPGESGREGAPGAEGSPGR | 5 Oxidation (P) | 3 | 2869.32 | 0.46 | 1379500 | 73 |
|  |  | GQAGVMGFPGPK | Oxidation (M),Oxidation (P) | 2 | 1176.56 | 0.90 | 34427000 | 106 |
|  |  |  | Oxidation (M),Deamidation (NQ),Oxidation (P) | 2 | 1177.54 | 0.45 | 22832000 | 79 |
|  |  |  | Oxidation (M),Deamidation (NQ),2 Oxidation (P) | 2 | 1193.54 | 3.02 | 2251600 | 73 |
|  |  | GSAGPPGATGFPGAAGR | 2 Oxidation (P) | 2 | 1458.69 | -0.39 | 2,91E+08 | 100 |
|  |  |  | 3 Oxidation (P) | 2 | 1474.68 | -1.15 | 6546900 | 176 |
|  |  | GSEGPQGVRGEPGPPGPAGAAGPAGNPGADGQPGAK | 2 Deamidation (NQ),3 Oxidation (P) | 3 | 3168.43 | 0.32 | 2613600 | 67 |
|  |  |  | 3 Oxidation (P) | 3 | 3166.47 | -0.31 | 2524900 | 40 |
|  |  |  | 4 Oxidation (P) | 3 | 3182.46 | -0.82 | 2419800 | 52 |
|  |  | GSPGADGPAGAPGTPGPQGIGGQR | Oxidation (P) | 3 | 2073.98 | 1.46 | 5927700 | 112 |
|  |  |  | 2 Oxidation (P) | 3 | 2089.98 | 0.54 | 12363000 | 52 |
|  |  |  | Deamidation (NQ),2 Oxidation (P) | 2 | 2090.96 | 0.49 | 7865100 | 96 |
|  |  |  | Deamidation (NQ),Oxidation (P) | 2 | 2074.97 | 0.11 | 3926100 | 85 |
|  |  |  | Deamidation (NQ),3 Oxidation (P) | 2 | 2106.96 | -0.48 | 3106700 | 95 |
|  |  |  | 3 Oxidation (P) | 3 | 2105.97 | 0.89 | 2749800 | 58 |
|  |  |  | 2 Deamidation (NQ),2 Oxidation (P) | 2 | 2091.95 | -0.19 | 1450000 | 58 |
|  |  |  | 2 Deamidation (NQ),Oxidation (P) | 2 | 2075.95 | -0.46 | 1303200 | 69 |
|  |  |  | 2 Deamidation (NQ),3 Oxidation (P) | 2 | 2107.94 | -0.43 | 489590 | 99 |
|  |  | GSPGEAGRPGEAGLPGAK | 2 Oxidation (P) | 3 | 1638.80 | 0.46 | 2707400 | 119 |
|  |  | GVPGPPGAVGAAGKDGEAGAQGPPGPAGPAGER | 3 Oxidation (P) | 3 | 2853.36 | -0.23 | 2306000 | 70 |
|  |  |  | 2 Oxidation (P) | 3 | 2837.37 | -0.13 | 1586700 | 54 |
|  |  | GVQGPPGPAGPR | Oxidation (P) | 2 | 1104.57 | 0.03 | 85243000 | 91 |
|  |  |  | Deamidation (NQ),Oxidation (P) | 2 | 1105.55 | -0.19 | 34494000 | 124 |
|  |  |  | Unmodified | 2 | 1088.57 | 0.41 | 1682400 | 99 |
|  |  |  | Deamidation (NQ) | 2 | 1089.56 | -1.26 | 615130 | 112 |
|  |  | GVVGLPGQR | Deamidation (NQ),Oxidation (P) | 2 | 898.49 | 0.94 | 59944000 | 115 |
|  |  |  | Oxidation (P) | 2 | 897.50 | -0.93 | 1,64E+08 | 133 |
|  |  | GVVGLPGQRGER | Oxidation (P) | 3 | 1239.67 | -0.51 | 476420 | 82 |
|  |  |  | Deamidation (NQ),Oxidation (P) | 3 | 1240.65 | -0.20 | 4137000 | 70 |
|  |  | PGEVGPPGPPGPAGEK | 3 Oxidation (P) | 2 | 1489.70 | -1.05 | 1759400 | 123 |
|  |  | QGPSGSSGERGPPGPAGPPGLAGPPGESGR | Gln->pyro-Glu,2 Oxidation (P) | 3 | 2681.24 | 0.25 | 2826200 | 70 |
|  |  |  | Gln->pyro-Glu,3 Oxidation (P) | 3 | 2697.24 | -0.64 | 1407400 | 68 |
|  |  |  | 2 Oxidation (P) | 3 | 2698.27 | -0.08 | 837580 | 62 |
|  |  | SGDRGETGPAGPAGPAGPAGVR | Unmodified | 3 | 1932.94 | -0.23 | 54038000 | 75 |
|  |  | VGPPGPSGNAGPPGPPGPAGK | Deamidation (NQ),4 Oxidation (P) | 2 | 1828.86 | 0.03 | 9116300 | 162 |
|  |  |  | 4 Oxidation (P) | 2 | 1827.88 | 0.21 | 11634000 | 79 |
|  |  |  | Deamidation (NQ),3 Oxidation (P) | 2 | 1812.86 | 0.07 | 5296200 | 93 |
|  |  |  | 5 Oxidation (P) | 2 | 1843.87 | 0.09 | 3798100 | 70 |
|  |  |  | 3 Oxidation (P) | 2 | 1811.88 | -0.38 | 5787300 | 77 |
|  |  | GFPGLPGPSGEPGK | Oxidation (P) | 2 | 1311.65 | 0.45 | 3665500 | 84 |
|  |  |  | 3 Oxidation (P) | 2 | 1343.64 | 0.12 | 3118300 | 89 |
| P0C2W8 | Collagen alpha-1(I) chain  (*Mammut americanum*) | GNDGATGAAGPPGPTGPAGPPGFPGAVGAK | 3 Oxidation (P) | 3 | 2547.20 | -0.34 | 15346000 | 52 |
|  |  |  | 2 Oxidation (P) | 3 | 2531.20 | -0.67 | 2670900 | 51 |
|  |  | GNDGATGAAGPPGPTGPAGPPGFPGAVGAKGEAGPQGAR | 4 Oxidation (P) | 3 | 3386.59 | -0.05 | 2700300 | 48 |
|  |  | GPPGSAGAPGKDGLNGLPGPPGPPGPR | 4 Oxidation (P) | 3 | 2437.20 | 4.41 | 566880 | 83 |
|  |  | TGPPGPAGQDGRPGPPGPPGAR | 5 Oxidation (P) | 3 | 2071.97 | -3.51 | 1,31E+08 | 80 |
|  |  |  | 4 Oxidation (P) | 3 | 2055.97 | -3.67 | 35081000 | 105 |
|  |  |  | Deamidation (NQ),3 Oxidation (P) | 3 | 2040.96 | -0.08 | 1330900 | 84 |
| G3TIC0 | Collagen type I alpha 2 chain  (*Loxodonta africana*) | EGPAGLPGIDGR | Oxidation (P) | 2 | 1153.57 | 0.88 | 13966000 | 114 |
|  |  | EGPAGLPGIDGRPGPIGPAGAR | 2 Oxidation (P) | 3 | 2043.05 | 0.75 | 19449000 | 58 |
|  |  |  | Oxidation (P) | 3 | 2027.05 | -0.22 | 18661000 | 81 |
|  |  | GAAGLPGVAGAPGLPGPR | 3 Oxidation (P) | 2 | 1561.82 | -0.50 | 26893000 | 123 |
|  |  | GAPGAVGAPGPAGATGDR | 2 Oxidation (P) | 2 | 1509.72 | -0.72 | 38353000 | 146 |
|  |  | GAPGAVGAPGPAGATGDRGEAGPAGSAGPAGPR | 2 Oxidation (P) | 3 | 2742.31 | -0.10 | 1,31E+08 | 78 |
|  |  |  | 3 Oxidation (P) | 3 | 2758.30 | -0.32 | 4699800 | 48 |
|  |  | GDGGPPGATGFPGAAGR | Oxidation (P) | 2 | 1456.67 | 0.69 | 5927700 | 153 |
|  |  |  | 2 Oxidation (P) | 2 | 1472.66 | 0.16 | 68098000 | 140 |
|  |  |  | 3 Oxidation (P) | 2 | 1488.66 | 1.94 | 3542600 | 126 |
|  |  | GEAGLPGVSGPVGPPGNPGANGLAGAK | Deamidation (NQ),3 Oxidation (P) | 2 | 2346.15 | -0.31 | 2323900 | 67 |
|  |  | GEAGPAGSAGPAGPR | Unmodified | 2 | 1250.60 | -1.28 | 1348700 | 84 |
|  |  | GEIGPVGNPGPSGPAGPR | Oxidation (P) | 2 | 1630.81 | -0.02 | 3785100 | 66 |
|  |  |  | Deamidation (NQ),Oxidation (P) | 2 | 1631.79 | 0.01 | 1934600 | 136 |
|  |  |  | Unmodified | 2 | 1614.81 | 0.28 | 867510 | 80 |
|  |  | GEPGAPGENGTPGQIGAR | Deamidation (NQ),3 Oxidation (P) | 2 | 1712.76 | 0.14 | 1041700 | 80 |
|  |  | GEPGNIGFPGPK | 2 Oxidation (P) | 2 | 1200.58 | -0.75 | 18268000 | 108 |
|  |  |  | Deamidation (NQ),2 Oxidation (P) | 2 | 1201.56 | -0.24 | 5553900 | 87 |
|  |  | GEPGPAGSVGPVGAVGPR | Oxidation (P) | 2 | 1575.80 | 0.47 | 33985000 | 102 |
|  |  |  | Unmodified | 2 | 1559.81 | 0.03 | 26436000 | 133 |
|  |  | GEPGSAGPQGPPGPSGEEGKR | 2 Oxidation (P) | 3 | 1978.90 | 0.42 | 3406300 | 51 |
|  |  | GEPGVVGAPGTAGPSGPGGLPGER | 3 Oxidation (P) | 2 | 2120.01 | -0.24 | 3379300 | 107 |
|  |  | GERGPPGQSGAAGPTGPIGSR | Deamidation (NQ),Oxidation (P) | 3 | 1921.92 | 0.72 | 6191900 | 92 |
|  |  |  | Oxidation (P) | 3 | 1920.94 | 0.61 | 5845100 | 121 |
|  |  |  | Deamidation (NQ),2 Oxidation (P) | 3 | 1937.92 | 0.88 | 491610 | 48 |
|  |  | GEVGPAGPNGFAGPAGAAGQAGAK | 2 Deamidation (NQ) | 2 | 2009.94 | 3.38 | 852500 | 76 |
|  |  |  | Deamidation (NQ) | 2 | 2008.96 | -1.31 | 754490 | 67 |
|  |  | GEVGPAGPNGFAGPAGAAGQAGAKGER | 2 Deamidation (NQ),Oxidation (P) | 3 | 2368.10 | 0.31 | 2581000 | 63 |
|  |  |  | Deamidation (NQ),Oxidation (P) | 3 | 2367.12 | 0.13 | 2540200 | 54 |
|  |  | GIPGEFGLPGPAGPR | 3 Oxidation (P) | 2 | 1468.73 | 0.36 | 2931000 | 103 |
|  |  |  | 2 Oxidation (P) | 3 | 1452.74 | -0.32 | 1186600 | 103 |
|  |  |  | 2 Oxidation (P) | 2 | 1452.74 | -0.56 | 1,91E+08 | 159 |
|  |  |  | 4 Oxidation (P) | 2 | 1484.73 | -0.32 | 468530 | 65 |
|  |  | GIPGPVGAAGATGAR | Oxidation (P) | 3 | 1266.67 | 1.28 | 1237000 | 91 |
|  |  |  | 2 Oxidation (P) | 2 | 1282.66 | 0.08 | 640730 | 71 |
|  |  | GLPGSPGNVGPAGK | 2 Oxidation (P) | 2 | 1238.63 | 0.49 | 37766000 | 122 |
|  |  |  | Deamidation (NQ),2 Oxidation (P) | 2 | 1239.61 | 0.56 | 19563000 | 108 |
|  |  | GLPGVAGAVGEPGPLGIAGPPGAR | 2 Oxidation (P) | 2 | 2098.12 | 0.52 | 26282000 | 94 |
|  |  |  | 4 Oxidation (P) | 2 | 2130.11 | -0.04 | 1590200 | 158 |
|  |  |  | 3 Oxidation (P) | 2 | 2114.11 | -0.41 | 61195000 | 147 |
|  |  | GLVGEPGPAGSK | Unmodified | 2 | 1067.56 | -0.67 | 1731100 | 70 |
|  |  | GPAGPSGPVGK | 2 Oxidation (P) | 1 | 954.48 | 0.35 | 1030900 | 51 |
|  |  | GPPGATGPPGSPGFQGPPGEPGEPGQTGPAGSR | 5 Oxidation (P) | 3 | 3031.35 | -0.40 | 6304400 | 89 |
|  |  |  | 4 Oxidation (P) | 3 | 3015.36 | -3.75 | 3021100 | 79 |
|  |  |  | 3 Oxidation (P) | 3 | 2999.36 | 0.16 | 1725900 | 66 |
|  |  |  | Deamidation (NQ),3 Oxidation (P) | 3 | 3000.35 | 0.52 | 1669300 | 53 |
|  |  | GPPGAVGSPGVNGAPGEAGR | Deamidation (NQ),3 Oxidation (P) | 2 | 1751.81 | -0.13 | 35756000 | 267 |
|  |  |  | 3 Oxidation (P) | 2 | 1750.82 | 0.01 | 1831100 | 136 |
|  |  |  | Deamidation (NQ),4 Oxidation (P) | 2 | 1767.80 | -0.15 | 1028600 | 70 |
|  |  |  | Deamidation (NQ),2 Oxidation (P) | 2 | 1735.81 | -0.08 | 571150 | 124 |
|  |  | GPPGQSGAAGPTGPIGSR | Deamidation (NQ),Oxidation (P) | 2 | 1579.76 | 0.46 | 64754000 | 206 |
|  |  |  | Oxidation (P) | 2 | 1578.78 | -0.19 | 58068000 | 196 |
|  |  |  | 2 Oxidation (P) | 2 | 1594.77 | -0.17 | 4739300 | 120 |
|  |  |  | Deamidation (NQ),2 Oxidation (P) | 2 | 1595.75 | 1.13 | 1497500 | 127 |
|  |  | GPSGPPGPDGNKGEPGVVGAPGTAGPSGPGGLPGER | 5 Oxidation (P) | 3 | 3212.50 | -0.18 | 12195000 | 115 |
|  |  |  | 4 Oxidation (P) | 3 | 3196.50 | -0.56 | 7259100 | 122 |
|  |  |  | 3 Oxidation (P) | 3 | 3180.51 | -1.95 | 1130400 | 47 |
|  |  |  | Deamidation (NQ),3 Oxidation (P) | 3 | 3181.49 | 4.41 | 1089200 | 52 |
|  |  | GSPGERGEVGPAGPNGFAGPAGAAGQAGAK | Deamidation (NQ),Oxidation (P) | 3 | 2608.23 | -0.62 | 1140700 | 43 |
|  |  | GSSGEPGTAGPPGTPGPQGILGPPGILGLPGSR | 4 Oxidation (P) | 3 | 2998.50 | 0.11 | 1773200 | 46 |
|  |  |  | 3 Oxidation (P) | 3 | 2982.50 | -1.70 | 548030 | 44 |
|  |  | GYPGNAGPVGTAGAPGPQGPLGPAGK | 2 Oxidation (P) | 3 | 2276.12 | -0.31 | 4310900 | 59 |
|  |  | TGETGASGPPGFAGEK | Oxidation (P) | 2 | 1477.67 | 0.12 | 28000000 | 158 |
|  |  | TGPPGPAGITGPPGPPGAAGK | 4 Oxidation (P) | 2 | 1816.90 | 1.08 | 11682000 | 117 |
|  |  |  | 5 Oxidation (P) | 2 | 1832.89 | 0.53 | 9216600 | 99 |
|  |  |  | 3 Oxidation (P) | 2 | 1800.90 | 0.01 | 6440100 | 98 |
| G3TH25 | Uncharacterized protein (COL3A1)  (*Loxodonta africana*) | DGPPGPPGSNGSPGTPGVSGPK | Deamidation (NQ),4 Oxidation (P) | 2 | 1979.87 | -0.78 | 607780 | 45 |
|  |  | DGTSGHPGPIGPPGPR | 2 Oxidation (P) | 3 | 1529.72 | -0.61 | 357860 | 48 |
|  |  | GDSGAPGERGPPGQAGPPGVR | 3 Oxidation (P) | 3 | 1962.91 | -3.40 | 2900300 | 106 |
|  |  |  | Deamidation (NQ),3 Oxidation (P) | 3 | 1963.90 | 0.82 | 266170 | 52 |
|  |  | GETGPAGPAGAPGPAGAR | Oxidation (P) | 2 | 1505.72 | -0.58 | 8145500 | 92 |
|  |  | GPAGANGLPGEKGPSGER | Deamidation (NQ),Oxidation (P) | 3 | 1666.79 | -0.37 | 3539900 | 151 |
|  |  |  | Deamidation (NQ),2 Oxidation (P) | 3 | 1682.79 | -0.22 | 320640 | 84 |
|  |  | GPPGAQGLPGLAGAAGEPGR | 3 Oxidation (P) | 2 | 1776.88 | -1.52 | 2405800 | 113 |
|  |  |  | Deamidation (NQ),3 Oxidation (P) | 2 | 1777.86 | 1.20 | 1149300 | 118 |
|  |  | GPPGAQGLPGLAGAAGEPGRDGNPGSDGLPGR | 5 Oxidation (P) | 3 | 2931.37 | 0.77 | 1677300 | 54 |
|  |  | GPPGPPGTNGIPGLR | Deamidation (NQ),3 Oxidation (P) | 2 | 1434.71 | 0.08 | 31341000 | 108 |
|  |  |  | Deamidation (NQ),4 Oxidation (P) | 2 | 1450.71 | 0.92 | 1165300 | 126 |
|  |  | GPPGPQGPPGAPGPLGLAGTTGSR | Deamidation (NQ),3 Oxidation (P) | 3 | 2144.05 | -0.26 | 791190 | 45 |
|  |  |  | 2 Oxidation (P) | 3 | 2127.07 | -1.31 | 535190 | 45 |
|  |  | GSDGLPGPPGPPGTSGFPGSPGAK | 5 Oxidation (P) | 2 | 2169.98 | -0.73 | 3545500 | 71 |
|  |  | GSPGGPGAAGFPGAR | 3 Oxidation (P) | 2 | 1302.60 | -0.30 | 28183000 | 67 |
|  |  | PGPPGSQGESGRPGPPGPSGPR | 4 Oxidation (P) | 3 | 2087.96 | -3.53 | 20618000 | 66 |
|  |  | SGDRGETGPAGPAGAPGPAGAR | Oxidation (P) | 3 | 1920.90 | 1.21 | 21946000 | 115 |
| G3T5Y9 | IF rod domain-containing protein  (*Loxodonta africana*) | ATAENEFVALK | Unmodified | 2 | 1191.61 | 0.07 | 13965000 | 148 |
|  |  | EQIKNLNSR | 2 Deamidation (NQ) | 2 | 1102.56 | 0.96 | 5224300 | 71 |
|  |  | GLTGGFGSR | Unmodified | 2 | 850.43 | -2.62 | 13408000 | 113 |
|  |  | LAGLEEALQK | Unmodified | 2 | 1070.60 | 0.61 | 19761000 | 231 |
|  |  | LEAAVTQAEQQGEVALNDAR | Unmodified | 3 | 2112.04 | -0.67 | 3974700 | 153 |
|  |  | AQYDDIASR | Unmodified | 2 | 1037.48 | 1.54 | 18666000 | 150 |
|  |  |  | Iodination | 2 | 1163.37 | 1.81 | 2776100 | 119 |
| G3UML8 | Myosin heavy chain 7  (*Loxodonta africana*) | DIDDLELTLAK | Unmodified | 2 | 1244.65 | 0.34 | 1127600 | 132 |
|  |  | DSLLIIQWNIR | Di-oxidation W | 2 | 1401.76 | -0.87 | 353310 | 97 |
|  |  |  | Deamidation (NQ),Di-oxidation W | 2 | 1402.75 | 1.15 | 1217300 | 142 |
|  |  | IEELEEELEAER | Unmodified | 2 | 1487.70 | -0.43 | 4428100 | 88 |
|  |  | NNLLQAELEELR | Deamidation (NQ) | 2 | 1441.74 | 1.09 | 1053100 | 104 |
|  |  |  | 2 Deamidation (NQ) | 2 | 1442.73 | 1.00 | 419240 | 118 |
|  |  | TLEDQMNEHRSK | Oxidation (M) | 2 | 1502.68 | -4.32 | 15592000 | 73 |
|  |  |  | Oxidation (M),Deamidation (NQ) | 2 | 1503.66 | -3.32 | 1556700 | 68 |
|  |  | VVDSLQTSLDAETR | Deamidation (NQ) | 2 | 1533.75 | 0.42 | 1728100 | 171 |
| G3SM37 | SWI/SNF related, matrix associated, actin dependent regulator of chromatin, subfamily d, member 1  (*Loxodonta africana*) | PGLAQSGMDQSRK | 2 Deamidation (NQ) | 2 | 1375.64 | -1.06 | 1317600 | 44 |
|  |  | RLDIQEALK | Deamidation (NQ) | 2 | 1085.61 | 0.39 | 1108000 | 113 |
|  |  | ETQPGQSQMPHQEHPVQK | 4 Deamidation (NQ) | 2 | 2088.91 | -2.86 | 2816800 | 59 |
| G3SR99 | Junction plakoglobin  (*Loxodonta africana*) | ALMGSPQLVAAVVR | Oxidation (M) | 2 | 1426.80 | 0.43 | 3656700 | 135 |
|  |  | LLNDEDPVVVTK | Unmodified | 2 | 1340.72 | 4.41 | 3474600 | 102 |
|  |  | NEGTATYAAAVLFR | Unmodified | 2 | 1482.75 | -0.64 | 1940200 | 115 |
|  |  | VSVELTNSLFK | Unmodified | 2 | 1235.68 | -0.27 | 2499700 | 156 |
| G3T2X7 | Collagen type IV alpha 2 chain  (*Loxodonta africana*) | GLPGEVLGAQPGPR | Oxidation (P) | 2 | 1362.73 | 0.75 | 1799600 | 86 |
|  |  | GVPGDIGPPGPK | Oxidation (P) | 2 | 1105.58 | -0.17 | 4127500 | 97 |
|  |  | PGPPGPPGPK | 3 Oxidation (P) | 1 | 947.47 | 0.11 | 483650 | 40 |
| G3TRF5 | SH3 domain-containing protein  (*Loxodonta africana*) | AELIVQPELK | Unmodified | 2 | 1138.66 | 0.17 | 2729000 | 109 |
|  |  | ALLQALLQTEDMLK | Oxidation (M) | 2 | 1601.87 | -0.50 | 1616400 | 103 |
|  |  | GFFDPNTEENLTYLQLK | Unmodified | 2 | 2027.98 | 1.01 | 1209200 | 113 |
|  |  | IEVLEEELR | Unmodified | 2 | 1128.60 | -1.64 | 2844600 | 151 |
|  |  | LLEAQIATGGIIDPK | Unmodified | 2 | 1537.87 | -0.86 | 4036200 | 143 |
|  |  | TLELQGLINDLQR | Unmodified | 2 | 1511.83 | 0.73 | 998860 | 135 |
|  |  | VLLQEEGAR | Unmodified | 2 | 1013.55 | -0.29 | 1159800 | 134 |
| G3TSV8 | ATP synthase subunit beta  (*Loxodonta africana*) | FTQAGSEVSALLGR | Unmodified | 2 | 1434.75 | -0.64 | 2621000 | 183 |
|  |  |  | Deamidation (NQ) | 2 | 1435.73 | 0.29 | 705710 | 127 |
|  |  | IGLFGGAGVGK | Unmodified | 2 | 974.55 | 0.23 | 353820 | 93 |
|  |  | TIAMDGTEGLVR | Oxidation (M) | 2 | 1277.63 | -0.04 | 1180000 | 118 |
| G3T4G5 | Nebulin  (*Loxodonta africana*) | KNKINYSESLYR | Oxidation Y,O-2H (Y) | 2 | 1543.76 | 0.93 | 1451500 | 74 |
|  |  | LQSENLYRR | 2 Deamidation (NQ) | 2 | 1179.59 | 1.61 | 435050 | 81 |
|  |  | RNQENFSSVLYK | dioxidation Y | 2 | 1515.73 | -2.89 | 570970 | 65 |
| G3T4X7 | Uncharacterized protein (COL2A1)  (*Loxodonta africana*) | GAQGPPGATGFPGAAGR | 3 Oxidation (P) | 2 | 1515.71 | 0.23 | 23706000 | 79 |
|  |  | GPSGLAGPKGANGDPGRPGEPGLPGAR | Deamidation (NQ),2 Oxidation (P) | 3 | 2471.22 | 0.23 | 2789500 | 52 |
| G3T7E7 | Annexin  (*Loxodonta africana*) | GVDEVTIVNILTNR | Unmodified | 2 | 1541.84 | -0.45 | 1096900 | 130 |
|  |  | QDIAFAYQR | Gln->pyro-Glu | 2 | 1093.52 | 0.74 | 558970 | 72 |
|  |  | TNQELQEINR | Unmodified | 2 | 1243.62 | -0.74 | 955490 | 105 |
| G3T8C4 | Collagen type XVII alpha 1 chain  (*Loxodonta africana*) | GPMGPPGPK | 3 Oxidation (P) | 1 | 884.41 | -3.06 | 471970 | 65 |
|  |  | GSVGEPGMEGPMGLRGR | 2 Oxidation (M) | 2 | 1717.79 | 1.35 | 650580 | 45 |
| G3T9K9 | Inositol-polyphosphate 5-phosphatase  (*Loxodonta africana*) | ENAAPVPSEPR | Deamidation (NQ) | 2 | 1166.56 | 3.16 | 989460 | 57 |
|  |  | LQVTQHSYR | 2 Deamidation (NQ),Oxidation Y | 2 | 1148.55 | 1.70 | 540820 | 42 |
| G3TQI0 | 60S ribosomal protein L40  (*Loxodonta africana*) | IQDKEGIPPDQQR | Unmodified | 3 | 1522.77 | -0.59 | 471300 | 83 |
|  |  | TITLEVEPSDTIENVK | Unmodified | 2 | 1786.92 | 1.03 | 5378700 | 157 |
|  |  | TLSDYNIQK | Unmodified | 2 | 1080.55 | 2.06 | 2663500 | 119 |
| G3TW48 | Elongation factor 1-alpha  (*Loxodonta africana*) | IGGIGTVPVGR | Unmodified | 2 | 1024.60 | 0.86 | 3483000 | 158 |
|  |  | LPLQDVYK | Unmodified | 2 | 974.54 | -0.36 | 1617100 | 118 |
|  |  | QTVAVGVIK | Unmodified | 2 | 913.56 | 0.71 | 1074400 | 96 |
| G3SL94 | IF rod domain-containing protein (KRT74)  (*Loxodonta africana*) | AEAEALYQSK | Oxidation Y | 2 | 1124.53 | 0.15 | 45038000 | 122 |
|  |  | FLEQQNQVLETK | Unmodified | 2 | 1475.76 | -0.22 | 31092000 | 229 |
| G3STK4 | Ubiquitin carboxyl-terminal hydrolase  (*Loxodonta africana*) | EHLQNLENSAFTADR | Glu->pyro-Glu,Deamidation (NQ) | 2 | 1726.79 | -1.99 | 1046100 | 51 |
|  |  | HYEDAQVPLTNHK | Deamidation (NQ),Oxidation Y | 2 | 1567.73 | -2.40 | 17335000 | 82 |
| G3STP4 | Collagen type IV alpha 4 chain  (*Loxodonta africana*) | GIPGDPAYGYPGPPGK | 2 Oxidation Y | 2 | 1573.74 | -0.86 | 464540 | 50 |
| G3SXH0 | Kinesin family member 7  (*Loxodonta africana*) | QGSAHSQQQIRR | 2 Deamidation (NQ) | 2 | 1396.68 | 0.67 | 1773700 | 54 |
| G3SYB4 | GTF2I repeat domain containing 1  (*Loxodonta africana*) | RQGFQENYDAR | Unmodified | 2 | 1382.63 | 2.64 | 178610 | 56 |
|  |  | SIPRSTLEHSSDVYLLRK | O-2H (Y) | 3 | 2114.11 | 0.17 | 27115000 | 53 |
| G3T0G1 | Sacsin molecular chaperone  (*Loxodonta africana*) | EVMNAFWPGR | Glu->pyro-Glu,Deamidation (NQ) | 2 | 1188.54 | 4.16 | 555050 | 50 |
|  |  | KSAHLGGAEEK | Acetyl (K) | 2 | 1167.59 | -2.06 | 1373000 | 55 |
| G3T0Y3 | PATJ crumbs cell polarity complex component  (*Loxodonta africana*) | LGNEDFNSIIQQMAQGR | 2 Deamidation (NQ) | 2 | 1921.88 | -2.09 | 534410 | 49 |
|  |  | LRAGSWPSSRK | Trp->Kynurenine | 2 | 1247.67 | 1.03 | 604680 | 73 |
| G3T316 | Desmin  (*Loxodonta africana*) | ADVDAATLSR | Unmodified | 2 | 1017.51 | 0.77 | 2541000 | 191 |
|  |  | VAELYEEELR | Unmodified | 2 | 1249.62 | 1.22 | 1203500 | 142 |
| G3T386 | Actin gamma 1  (*Loxodonta africana*) | AVFPSIVGR | Unmodified | 2 | 944.54 | -1.64 | 634230 | 117 |
|  |  | SYELPDGQVITIGNER | Unmodified | 2 | 1789.88 | 0.92 | 1190500 | 88 |
|  |  | VAPEEHPVLLTEAPLNPK | Unmodified | 3 | 1953.06 | 0.95 | 1544900 | 58 |
| G3T3E4 | CTD small phosphatase like 2  (*Loxodonta africana*) | KSQVNGEAGSYEMTNQHVK | Deamidation (NQ) | 2 | 2106.96 | 1.48 | 288350 | 49 |
|  |  | PSINNGLEEAEGTVNR | 2 Deamidation (NQ) | 2 | 1700.79 | -1.54 | 749650 | 52 |
| G3T5I6 | DNA-directed DNA/RNA polymerase mu  (*Loxodonta africana*) | LVNMSWFTESMAAGQPVPVECR | Deamidation (NQ),2 di-oxidation (M) | 3 | 2573.12 | 1.12 | 3011800 | 50 |
| G3T930 | Protein kinase domain-containing protein  (*Loxodonta africana*) | VEYQENGPLFSELKFYQR | 2 Deamidation (NQ),Iodination | 2 | 2373.97 | 0.24 | 1435800 | 40 |
| G3T9Y7 | Plakophilin 1  (*Loxodonta africana*) | LDAEVPTR | Unmodified | 2 | 899.47 | 0.44 | 1123500 | 125 |
|  |  | SPNQNVQQAAAGALR | Unmodified | 2 | 1523.78 | -1.26 | 1926700 | 147 |
| G3TDU4 | ATP synthase subunit alpha  (*Loxodonta africana*) | TGAIVDVPVGEELLGR | Unmodified | 2 | 1623.88 | -0.20 | 511330 | 74 |
|  |  | VLSIGDGIAR | Unmodified | 2 | 999.57 | -0.04 | 615140 | 99 |
| G3TE09 | Golgin B1  (*Loxodonta africana*) | DKLIAEMDR | Acetyl (K),di-oxidation (M) | 2 | 1163.55 | -2.81 | 4106000 | 71 |
|  |  | ELQSNKELVKSQMK | 3 Deamidation (NQ) | 2 | 1663.83 | -0.20 | 1415700 | 50 |
| G3TGT5 | Uncharacterized protein  (*Loxodonta africana*) | LPGLMEGQEYSFRVR | Oxidation (M),dioxidation Y | 2 | 1828.88 | -4.39 | 3642100 | 49 |
|  |  | PGTAQWNRINK | Deamidation (NQ),Trp->Kynurenine | 2 | 1288.65 | -2.64 | 577650 | 43 |
| G3TJF5 | Desmoglein 1  (*Loxodonta africana*) | PGSKTYVVNSKMGANYR | Deamidation (NQ),Oxidation Y,dioxidation Y | 2 | 1919.90 | 0.61 | 852890 | 64 |
| G3TK66 | Elastin microfibril interfacer 3  (*Loxodonta africana*) | PREGLWGHMDQLNR | Oxidation (M),Trp->Kynurenine | 2 | 1727.82 | -1.18 | 776870 | 41 |
|  |  | SPSGCSWFGTMPSAVTRPSQR | Trp->Kynurenine | 3 | 2299.05 | -0.70 | 3210500 | 46 |
| G3TM52 | VLIG-type G domain-containing protein  (*Loxodonta africana*) | EKGNRSIEQHK | Glu->pyro-Glu | 2 | 1306.67 | -0.80 | 381440 | 42 |
|  |  | LGIDHYYPK | 2 dioxidation Y | 2 | 1168.54 | -2.87 | 602560 | 55 |
| G3TQH2 | Zinc finger and BTB domain containing 26  (*Loxodonta africana*) | GLQWHHQCPKCTR | 2 Deamidation (NQ),Trp->Kynurenine | 2 | 1712.75 | 4.16 | 1041700 | 45 |
|  |  | PKQPMNSK | Acetyl (K),Oxidation (M),Deamidation (NQ) | 2 | 987.47 | -3.14 | 637600 | 64 |
| G3TQN3 | RING-type E3 ubiquitin transferase  (*Loxodonta africana*) | VNEYIDARDTNMGAWFEAQVVRVTR | Deamidation (NQ),Di-oxidation W | 3 | 2972.41 | -2.15 | 730310 | 42 |
|  |  | YLLRRDDEEPGPWTK | O-2H (W) | 3 | 1887.91 | 0.88 | 4464400 | 41 |
| G3TW77 | Uncharacterized protein (HSPA8)  (*Loxodonta africana*) | DAGTIAGLNVLR | Unmodified | 2 | 1198.67 | 0.72 | 352310 | 99 |
|  |  | IINEPTAAAIAYGLDK | Unmodified | 2 | 1658.89 | 0.15 | 1161900 | 91 |
|  |  | TTPSYVAFTDTER | Unmodified | 2 | 1486.69 | 0.86 | 1949200 | 101 |
| G3TID3 | IF rod domain-containing protein  (*Loxodonta africana*) | TVNALEVELQAQHNLR | Unmodified | 3 | 1833.97 | 1.61 | 6371800 | 191 |
|  |  | DSLENTLTETEAR | Unmodified | 2 | 1477.69 | 1.30 | 40714000 | 290 |
| G3U1Z7 | Uncharacterized protein  (*Loxodonta africana*) | GPRSSSATFQLQTLQIVCNGSWAEEDR | Deamidation (NQ),O-2H (W) | 3 | 3051.40 | 4.35 | 3098800 | 45 |
| G3U3N5 | Kelch like family member 32  (*Loxodonta africana*) | LQQPLSTRPWNTTR | 2 Deamidation (NQ),Di-oxidation W | 2 | 1730.86 | 0.79 | 807880 | 41 |
|  |  | PWNTTRASMHSWQTGR | Deamidation (NQ),Trp->Kynurenine | 2 | 1919.87 | 2.94 | 908500 | 44 |
| G3U5X4 | Dendrin  (*Loxodonta africana*) | ENVEQSWLLK | Di-oxidation W | 3 | 1276.63 | -1.49 | 2010800 | 69 |
| G3U7J1 | Dedicator of cytokinesis 9  (*Loxodonta africana*) | HTQPYTIYNNHFYVYPK | 2 Deamidation (NQ),Iodination,Di-iodination,dioxidation Y | 4 | 2595.69 | 1.61 | 16581000 | 46 |
|  |  | YAYIQVTHVTPFFDEK | 2 Di-iodination | 4 | 2460.55 | 2.39 | 2048400 | 43 |
| G5E786 | Shroom family member 2  (*Loxodonta africana*) | ADGSSTENLLYKVGLWESCR | Trp->Kynurenine | 3 | 2288.07 | -0.94 | 711300 | 47 |
| Q6B812 | Histone H4  (*Mammuthus primigenius*) | ISGLIYEETR | Unmodified | 2 | 1179.61 | 0.17 | 3591500 | 159 |
|  |  | VFLENVIR | Unmodified | 2 | 988.57 | 0.11 | 1344900 | 111 |
| G3U7X8 | Collagen type V alpha 1 chain  (*Loxodonta africana*) | GPNGPQGPTGFPGPK | 2 Deamidation (NQ),Oxidation (P) | 2 | 1424.66 | -3.24 | 424360 | 56 |
|  |  | PGLPGADGLPGPPGTMLMLPFR | 3 Oxidation (P) | 3 | 2238.12 | 3.46 | 653460 | 44 |
| G3UNI5 | Formin like 1  (*Loxodonta africana*) | EASGRRER | Glu->pyro-Glu | 1 | 941.48 | 2.99 | 852260 | 43 |
|  |  | GEPSGPKSPPK | 2 Oxidation (P) | 2 | 1111.55 | -0.63 | 2770700 | 83 |
| G3TIE2 | IF rod domain-containing protein (KRT32)  (*Loxodonta africana*) | LESEINTYR | Deamidation (NQ) | 2 | 1124.53 | 1.49 | 937280 | 165 |
|  |  |  | Unmodified | 2 | 1123.55 | -1.42 | 23927000 | 238 |
|  |  |  | Iodination | 2 | 1249.45 | 0.92 | 1902800 | 214 |
| G3TEV7 | Tudor domain-containing protein 5  (*Loxodonta africana*) | TSKSNAVETSR | Unmodified | 2 | 1178.59 | 2.66 | 1005800 | 58 |
|  |  |  | Deamidation (NQ) | 2 | 1179.57 | 2.96 | 302610 | 52 |
| G3UL08 | Histone H2B  (*Loxodonta africana*) | AMGIMNSFVNDIFER | 2 Oxidation (M) | 2 | 1774.80 | 0.44 | 770180 | 62 |
|  |  |  | Oxidation (M),di-oxidation (M) | 2 | 1790.80 | 3.30 | 316070 | 64 |
| G3UD76 | PALM2 and AKAP2 fusion  (*Loxodonta africana*) | QFQLMENSR | Oxidation (M),Deamidation (NQ) | 2 | 1168.52 | -1.04 | 7992900 | 90 |
|  |  |  | Oxidation (M),2 Deamidation (NQ) | 2 | 1169.50 | -2.72 | 893000 | 93 |
| G3UG71 | Integrator complex subunit 6  (*Loxodonta africana*) | PGEPNMQGIPK | Oxidation (M),Deamidation (NQ),Oxidation (P) | 2 | 1199.55 | -3.19 | 5019900 | 67 |
|  |  | PGEPNMQGIPKR | 2 Oxidation (P) | 2 | 1354.67 | 0.61 | 362950 | 78 |
| G3TU05 | Collectin-12  (*Loxodonta africana*) | PGPQGPSGDPGPPGPPGK | Deamidation (NQ),3 Oxidation (P) | 2 | 1643.74 | 0.90 | 1090000 | 56 |
| G3T1Z8 | Peroxiredoxin 2  (*Loxodonta africana*) | QITVNDLPVGR | Gln->pyro-Glu | 2 | 1193.64 | 1.26 | 1345200 | 105 |
|  |  |  | Unmodified | 2 | 1210.67 | 0.39 | 951430 | 93 |
| G3SPJ3 | Coronin  (*Loxodonta africana*) | PGSELLSPQPHPPER | Deamidation (NQ),3 Oxidation (P) | 2 | 1688.80 | 0.02 | 2217600 | 61 |
| G3SR76 | DNA (cytosine-5)-methyltransferase  (*Loxodonta africana*) | PRKEPVDEDLYPEHYR | dioxidation Y,O-2H (Y) | 3 | 2087.95 | -0.63 | 20618000 | 53 |
|  |  |  | Oxidation Y,O-2H (Y) | 3 | 2071.96 | 0.86 | 1,07E+08 | 40 |
| G3T5Y2 | EF-hand calcium binding domain 12  (*Loxodonta africana*) | DNFLVYLQCWK | 2 Deamidation (NQ),Oxidation Y | 2 | 1502.68 | -2.68 | 15084000 | 74 |
|  |  |  | 2 Deamidation (NQ),Oxidation (W) | 2 | 1502.68 | -3.27 | 13713000 | 41 |
| A0A173GHX3 | Cytochrome c oxidase subunit 2  (*Mammuthus sp.*) | MVLPTDLPVR | Oxidation (M) | 2 | 1155.63 | 1.65 | 2242500 | 131 |
| C6FWG3 | Glyceraldehyde dehydrogenase  (*Elephas maximus*) | QASEGPLK | Gln->pyro-Glu | 1 | 811.41 | -0.71 | 750840 | 63 |
| G3SKX9 | Interleukin 12 receptor subunit beta 2  (*Loxodonta africana*) | LILYKFYRK | Iodination,Oxidation Y | 2 | 1384.64 | 4.26 | 820030 | 60 |
| G3SKZ3 | Purinergic receptor P2Y14  (*Loxodonta africana*) | INCMDLKSQLGQK | 2 Deamidation (NQ),di-oxidation (M) | 2 | 1567.72 | 0.36 | 34359000 | 65 |
| G3SL22 | Protein RIC1 homolog7  (*Loxodonta africana*) | SISLSQSAENVPASK | Deamidation (NQ) | 3 | 1517.76 | -1.76 | 2019300 | 46 |
| G3SLB3 | Uncharacterized protein  (*Loxodonta africana*) | VENEQKVK | 2 Deamidation (NQ) | 2 | 974.49 | -1.81 | 96427000 | 81 |
| G3SLF8 | ATP synthase F1 subunit epsilon  (*Loxodonta africana*) | AVRDAMKIEFK | Oxidation (M) | 3 | 1322.70 | 3.39 | 169520 | 53 |
| G3SLR4 | TBC1 domain family member 10A  (*Loxodonta africana*) | GPLVGGWAEYLPGYYSEK | Oxidation Y,O-2H (Y) | 2 | 2014.93 | -3.71 | 2073600 | 69 |
| G3SLY5 | Phospholipid-transporting ATPase, EC 7.6.2.1  (*Loxodonta africana*) | DCTWQVKANDR | 2 Deamidation (NQ),Oxidation (W) | 2 | 1409.59 | 2.15 | 707550 | 49 |
| G3SLZ6 | COMM domain containing 6  (*Loxodonta africana*) | IKGQLVDFQWK | Deamidation (NQ),Oxidation (W) | 2 | 1377.73 | -3.33 | 1301700 | 49 |
| G3SM13 | TBC1 domain family member 2  (*Loxodonta africana*) | GPQREEQPSPPDPSTPGKEPADSPK | Acetyl (K),Deamidation (NQ) | 3 | 2670.24 | 0.93 | 993280 | 59 |
| G3SMA5 | Homeobox A13  (*Loxodonta africana*) | VINKLKTTS | 2 Acetyl (K),Deamidation (NQ) | 2 | 1087.61 | 0.62 | 2086100 | 70 |
| G3SMC8 | Kinesin-like protein  (*Loxodonta africana*) | RLEARLQGMVTETTMK | Oxidation (M) | 3 | 1878.97 | -4.41 | 648500 | 46 |
| G3SMK0 | SWI/SNF related, matrix associated, actin dependent regulator of chromatin, subfamily a, member 1  (*Loxodonta africana*) | QPNVQDFQFFPPR | Deamidation (NQ) | 3 | 1619.77 | 3.34 | 758750 | 41 |
| G3SMM6 | AAA domain-containing protein  (*Loxodonta africana*) | KPNIFYSGPASPAR | Deamidation (NQ),2 Oxidation (P) | 2 | 1536.76 | -1.78 | 244920 | 84 |
| G3SMS5 | Non-specific serine/threonine protein kinase, EC 2.7.11.1  (*Loxodonta africana*) | NNILNLKR | 2 Deamidation (NQ) | 2 | 985.56 | -0.18 | 1335700 | 67 |
| G3SMT9 | Glutamyl-tRNA synthetase, EC 6.1.1.17 (Probable glutamate--tRNA ligase, mitochondrial)  (*Loxodonta africana*) | TALYNYIFARKHR | Deamidation (NQ),Oxidation Y,dioxidation Y | 2 | 1700.86 | 2.86 | 366320 | 46 |
| G3SMU3 | Partner and localizer of BRCA2  (*Loxodonta africana*) | MTVFANNPVINK | Oxidation (M),2 Deamidation (NQ) | 2 | 1364.66 | 4.08 | 1163600 | 67 |
| G3SMX4 | PWI domain-containing protein  (*Loxodonta africana*) | EAAYQERLK | Glu->pyro-Glu,dioxidation Y | 2 | 1120.55 | 1.61 | 359370 | 45 |
| G3SN23 | WD repeat domain 20  (*Loxodonta africana*) | QGESFAVHTCKSKSTR | Acetyl (K),Trioxidation (C) | 2 | 1911.87 | 4.02 | 2952300 | 75 |
| G3SNA0 | SMG6 nonsense mediated mRNA decay factor  (*Loxodonta africana*) | DFMPTNKEEPIR | Acetyl (K),di-oxidation (M) | 2 | 1549.71 | -0.69 | 1005700 | 46 |
| G3SNE2 | Anion exchange protein  (*Loxodonta africana*) | RGHGPPPPNPELLRTGR | 3 Oxidation (P) | 3 | 1897.99 | -1.36 | 794110 | 41 |
| G3SNQ7 | Calponin  (*Loxodonta africana*) | LAQKYDHQR | 2 Deamidation (NQ),O-2H (Y) | 2 | 1173.54 | 1.12 | 2887500 | 76 |
| G3SNW1 | Uncharacterized protein  (*Loxodonta africana*) | SVNGAEIIM | Deamidation (NQ) | 1 | 933.45 | 1.55 | 631230 | 43 |
| G3SNZ9 | Twinfilin actin binding protein 1  (*Loxodonta africana*) | LSNRQLNYVQLEIDIK | 2 Deamidation (NQ),Iodination | 2 | 2072.93 | -3.67 | 8319600 | 75 |
| G3SP18 | Transmembrane 7 superfamily member 2  (*Loxodonta africana*) | QLLVSGWWGMVR | Oxidation (M),O-2H (W) | 2 | 1460.72 | 1.10 | 1771100 | 67 |
| G3SPF3 | ARFGEF family member 3  (*Loxodonta africana*) | VSGIGGAANLYR | Deamidation (NQ) | 2 | 1177.61 | -0.60 | 389180 | 50 |
| G3SPH3 | Calcium-activated neutral proteinase 2, EC 3.4.22.53 (Calpain M-type) (Calpain-2 catalytic subunit) (Calpain-2 large subunit) (Millimolar-calpain)  (*Loxodonta africana*) | IQNYQKIYR | Deamidation (NQ),Oxidation Y,dioxidation Y | 2 | 1273.63 | -3.58 | 355100 | 65 |
| G3SPI5 | Ring finger and WD repeat domain 3  (*Loxodonta africana*) | GQARKCPQCNK | 3 Deamidation (NQ) | 2 | 1348.59 | 2.88 | 1385900 | 44 |
| G3SPJ0 | Netrin 5  (*Loxodonta africana*) | RPAWPGALGGPER | 2 Oxidation (P) | 2 | 1394.71 | 2.34 | 755680 | 43 |
| G3SPQ6 | Hydroxysteroid 11-beta dehydrogenase 1  (*Loxodonta africana*) | GIGEEMAYQLAK | Oxidation (M),Deamidation (NQ),O-2H (Y) | 2 | 1339.60 | 4.31 | 1364200 | 71 |
| G3SPR0 | Component of oligomeric Golgi complex 7 (Conserved oligomeric Golgi complex subunit 7)  (*Loxodonta africana*) | QATKSLPR | Acetyl (K),Deamidation (NQ) | 2 | 942.51 | -2.13 | 353570 | 109 |
| G3SPU0 | Nuclear protein MDM1  (*Loxodonta africana*) | TLQKCPSTK | Acetyl (K),Trioxidation (C) | 2 | 1151.55 | -2.77 | 630030 | 65 |
| G3SPY5 | Anoctamin  (*Loxodonta africana*) | KSNPQTEKR | Acetyl (K),Deamidation (NQ) | 2 | 1129.57 | 1.18 | 802660 | 72 |
| G3SQ16 | Matrix metalloproteinase-14, EC 3.4.24.80  (*Loxodonta africana*) | GLPASINTAYER | Deamidation (NQ),Oxidation Y | 2 | 1307.64 | 1.32 | 1027000 | 63 |
| G3SQ65 | Uncharacterized protein  (*Loxodonta africana*) | GPSEKCIRNEK | Deamidation (NQ) | 2 | 1317.63 | -2.55 | 626450 | 58 |
| G3SQE4 | Golgi SNAP receptor complex member 1  (*Loxodonta africana*) | ENMTSQRGMLK | Glu->pyro-Glu | 2 | 1275.61 | 1.57 | 1404100 | 59 |
| G3SQG6 | PDLIM1 interacting kinase 1 like  (*Loxodonta africana*) | EFWALSSIK | O-2H (W) | 2 | 1093.54 | -3.29 | 365940 | 114 |
| G3SQN8 | Cytochrome b-c1 complex subunit 7  (*Loxodonta africana*) | LYLEPYLKEVIR | 2 O-2H (Y) | 2 | 1562.83 | 0.89 | 782070 | 56 |
| G3SQQ0 | Uncharacterized protein  (*Loxodonta africana*) | NWNHPFPR | 2 Deamidation (NQ) | 2 | 1068.48 | 2.89 | 3227700 | 63 |
| G3SR03 | Tudor domain containing 3  (*Loxodonta africana*) | KRDNSMQSR | di-oxidation (M) | 2 | 1152.53 | -1.53 | 1365000 | 77 |
| G3SR52 | Vesicle amine transport 1 like  (*Loxodonta africana*) | QGNIDNPPK | Deamidation (NQ),Oxidation (P) | 1 | 998.47 | -0.48 | 1646200 | 45 |
| G3SR56 | Ectonucleoside triphosphate diphosphohydrolase 1  (*Loxodonta africana*) | NESLETYGALDLGGASTQITFVPK | Deamidation (NQ) | 3 | 2511.24 | 0.67 | 929900 | 44 |
| G3SR83 | alpha-1,2-Mannosidase, EC 3.2.1.-  (*Loxodonta africana*) | PELIESAMYLYR | Oxidation (M),Oxidation (P) | 2 | 1515.73 | 3.67 | 498300 | 76 |
| G3SRK1 | Cellular communication network factor 5  (*Loxodonta africana*) | LGEPCDHLHVCDSSQGLVCQLGRPCAR | Deamidation (NQ) | 3 | 3121.40 | -4.19 | 559920 | 41 |
| G3SRU0 | Kelch like family member 17  (*Loxodonta africana*) | HYHDAFVAMSRMRQR | Oxidation (M),Deamidation (NQ) | 2 | 1920.88 | 0.56 | 1779600 | 45 |
| G3SRU3 | Uncharacterized protein  (*Loxodonta africana*) | NKFCNVYSLKAK | 2 Acetyl (K),Trioxidation (C) | 2 | 1602.77 | 1.20 | 841850 | 51 |
| G3SRW4 | Remodeling and spacing factor 1  (*Loxodonta africana*) | QINYKEDSESDGSQK | 3 Deamidation (NQ) | 2 | 1729.72 | 2.77 | 395690 | 43 |
| G3SS13 | SWI/SNF related, matrix associated, actin dependent regulator of chromatin, subfamily d, member 2  (*Loxodonta africana*) | RPGDLNVK | Deamidation (NQ) | 2 | 898.49 | -1.43 | 59944000 | 71 |
| G3SS16 | COesterase domain-containing protein  (*Loxodonta africana*) | KPLLWAKMIAK | Oxidation (M),Trp->Kynurenine | 2 | 1317.78 | -0.71 | 1558900 | 42 |
| G3SS20 | Nexilin F-actin binding protein  (*Loxodonta africana*) | QKMEEDKR | di-oxidation (M) | 2 | 1094.50 | -2.02 | 726660 | 75 |
| G3SS30 | Potassium channel tetramerization domain containing 18  (*Loxodonta africana*) | PVRFLGPSTSTQIK | Deamidation (NQ),2 Oxidation (P) | 2 | 1562.83 | 1.44 | 705200 | 57 |
| G3SS62 | Chromosome alignment maintaining phosphoprotein 1  (*Loxodonta africana*) | SPPLPEQQK | 2 Deamidation (NQ),2 Oxidation (P) | 2 | 1056.50 | 1.85 | 2966500 | 69 |
| G3SS94 | TRAF3 interacting protein 1  (*Loxodonta africana*) | EKDIVSKEIEK | Acetyl (K),Glu->pyro-Glu | 2 | 1340.72 | 1.68 | 2406800 | 73 |
| G3SSM9 | Non-specific serine/threonine protein kinase, EC 2.7.11.1  (*Loxodonta africana*) | EKENQINSFGKSVPGPLK | Deamidation (NQ),2 Oxidation (P) | 2 | 2004.02 | 3.20 | 441300 | 59 |
| G3SSV8 | Protein SPT2 homolog  (*Loxodonta africana*) | GQGVNNVPK | 2 Deamidation (NQ) | 2 | 913.45 | -0.07 | 309000 | 63 |
| G3SSW2 | Uncharacterized protein  (*Loxodonta africana*) | MRLEQDLKK | Acetyl (K),Deamidation (NQ),di-oxidation (M) | 2 | 1234.62 | 0.26 | 4296700 | 116 |
| G3ST60 | SUZ12 polycomb repressive complex 2 subunit  (*Loxodonta africana*) | QQTEARDDLHCPWCTLNCRK | Gln->pyro-Glu | 3 | 2570.12 | -2.44 | 10238000 | 41 |
| G3ST80 | Trafficking protein particle complex 10  (*Loxodonta africana*) | AVIYSNTR | Deamidation (NQ),dioxidation Y | 2 | 955.46 | 0.45 | 1634000 | 53 |
| G3ST97 | Glycine receptor alpha 2  (*Loxodonta africana*) | DGAAVKATPANPLPPPPK | Deamidation (NQ),2 Oxidation (P) | 2 | 1772.93 | -3.70 | 458150 | 46 |
| G3STE6 | Phosphate transporter  (*Loxodonta africana*) | GVQWMELVK | Oxidation (W) | 2 | 1104.56 | 2.56 | 64427000 | 67 |
| G3STG1 | SET domain bifurcated histone lysine methyltransferase 1  (*Loxodonta africana*) | PGLNQTYR | dioxidation Y | 1 | 979.47 | -0.79 | 903310 | 52 |
| G3STJ5 | 60S ribosomal protein L13  (*Loxodonta africana*) | MAPSRNGMILK | 2 Oxidation (M) | 2 | 1248.63 | 0.72 | 537590 | 43 |
| G3STK9 | Sema domain-containing protein  (*Loxodonta africana*) | AKETGHWLWSR | Oxidation (W),Di-oxidation W | 2 | 1417.67 | -3.20 | 910250 | 83 |
| G3STM0 | PRA1 family protein  (*Loxodonta africana*) | NLKNKLENK | 2 Acetyl (K),2 Deamidation (NQ) | 2 | 1185.62 | -0.26 | 2195600 | 80 |
| G3STQ2 | Eomesodermin  (*Loxodonta africana*) | LSPSTSSNENSPSIK | Deamidation (NQ) | 2 | 1547.73 | -1.94 | 303280 | 45 |
| G3STU7 | ST18 C2H2C-type zinc finger transcription factor  (*Loxodonta africana*) | GAEIEVDENGTLDLSMK | Unmodified | 2 | 1819.85 | -2.14 | 1061000 | 78 |
| G3SU79 | Asporin  (*Loxodonta africana*) | HVFSPSYMTLK | Unmodified | 2 | 1308.65 | 2.61 | 372950 | 63 |
| G3SUE8 | Exocyst complex component 1  (*Loxodonta africana*) | GLDNLYKK | O-2H (Y) | 2 | 963.50 | -0.17 | 267240 | 70 |
| G3SUG6 | Filamin A interacting protein 1  (*Loxodonta africana*) | GPELICPEDNKIK | Deamidation (NQ),Trioxidation (C) | 2 | 1560.73 | 3.41 | 1253500 | 54 |
| G3SUH6 | Dual-specificity kinase, EC 2.7.12.1  (*Loxodonta africana*) | LEIVNYPEIYFVGPNAK | Oxidation Y,dioxidation Y | 2 | 2013.01 | -3.28 | 5610500 | 71 |
| G3SUJ7 | Mitochondrial ribosomal protein L12  (*Loxodonta africana*) | QLRSSSHRR | Gln->pyro-Glu | 2 | 1108.58 | 1.37 | 1292500 | 49 |
| G3SUK6 | Uncharacterized protein  (*Loxodonta africana*) | LQDLPNPQRYTR | 2 Deamidation (NQ),O-2H (Y) | 2 | 1515.73 | -2.77 | 939770 | 77 |
| G3SUK9 | Transmembrane channel-like protein  (*Loxodonta africana*) | LTLNQWLAR | 2 Deamidation (NQ) | 2 | 1115.60 | -2.47 | 2219800 | 89 |
| G3SUL6 | 26S proteasome regulatory subunit RPN11  (*Loxodonta africana*) | HYYSITINYRKNELEQK | Deamidation (NQ),O-2H (Y) | 3 | 2213.08 | -3.95 | 1723700 | 57 |
| G3SUY6 | Potassium calcium-activated channel subfamily M regulatory beta subunit 4  (*Loxodonta africana*) | VAYEYTEAEDKSIR | 2 dioxidation Y | 2 | 1736.77 | -1.80 | 815860 | 52 |
| G3SV01 | RAB3A interacting protein like 1  (*Loxodonta africana*) | QAASEKQLKEAR | Acetyl (K),Gln->pyro-Glu | 2 | 1382.72 | -1.92 | 7219700 | 74 |
| G3SV35 | Histone acetyltransferase, EC 2.3.1.48  (*Loxodonta africana*) | SVILECLYHQNDK | Oxidation Y | 2 | 1633.78 | 4.47 | 19527000 | 83 |
| G3SVF6 | Paraoxonase 2  (*Loxodonta africana*) | PGGILMMDLKEEK | 2 Oxidation (M) | 2 | 1491.73 | 1.90 | 3076900 | 48 |
| G3SVI1 | Ring finger protein 175  (*Loxodonta africana*) | KQTCPYCK | Trioxidation (C) | 2 | 1131.47 | 0.69 | 667460 | 72 |
| G3SVI5 | Phospholipase A2, EC 3.1.1.4  (*Loxodonta africana*) | LSEYNILNNSDK | 3 Deamidation (NQ) | 2 | 1411.64 | 2.20 | 2703600 | 56 |
| G3SVJ8 | THUMP domain containing 3  (*Loxodonta africana*) | KINQNSSK | Acetyl (K),3 Deamidation (NQ) | 2 | 962.46 | 0.68 | 1849600 | 104 |
| G3SVU5 | Uncharacterized protein  (*Loxodonta africana*) | IQMLDDTQEAFEVPQRAPGK | Oxidation (M),Deamidation (NQ),Oxidation (P) | 2 | 2305.09 | 3.21 | 644910 | 50 |
| G3SVV9 | 5'-nucleotidase, EC 3.1.3.5  (*Loxodonta africana*) | IIEMMPEFQK | 2 Oxidation (M),Deamidation (NQ) | 2 | 1297.59 | 0.02 | 694730 | 44 |
| G3SVW1 | Glucocorticoid induced 1  (*Loxodonta africana*) | GPSPPSPTPPPAAAPAEQAPRSK | 2 Oxidation (P) | 3 | 2239.12 | -4.21 | 2225500 | 50 |
| G3SVY2 | Bactericidal permeability-increasing protein  (*Loxodonta africana*) | LGSNPNSGR | 2 Deamidation (NQ) | 2 | 902.41 | -1.75 | 588680 | 53 |
| G3SVY6 | Cathepsin G  (*Loxodonta africana*) | WNDIMLLQLETR | Oxidation (M),Deamidation (NQ),Oxidation (W) | 2 | 1563.76 | -4.19 | 1746800 | 71 |
| G3SVZ0 | F-box and leucine rich repeat protein 4  (*Loxodonta africana*) | WEILWSERPTK | O-2H (W),Di-oxidation W | 2 | 1489.72 | -2.29 | 454050 | 56 |
| G3SW14 | Piccolo presynaptic cytomatrix protein  (*Loxodonta africana*) | HDREPSFNETFR | Unmodified | 2 | 1533.70 | -2.93 | 2228300 | 64 |
| G3SW18 | Torsin family 3 member A  (*Loxodonta africana*) | MLAENLYR | Oxidation Y | 1 | 1024.50 | 3.76 | 1352800 | 61 |
| G3SWC4 | S-adenosylmethionine synthase, EC 2.5.1.6  (*Loxodonta africana*) | KPIYQKTACYGHFGR | Deamidation (NQ),dioxidation Y,O-2H (Y) | 2 | 1871.86 | 2.38 | 190040 | 42 |
| G3SWD3 | Keratin 4  (*Loxodonta africana*) | VEIDPEIQK | Unmodified | 2 | 1069.57 | 1.62 | 5254000 | 140 |
| G3SWF0 | Transmembrane protein 209  (*Loxodonta africana*) | QAAEEVWAR | Oxidation (W) | 2 | 1074.51 | 0.58 | 9824200 | 59 |
| G3SWJ6 | 2-phospho-D-glycerate hydro-lyase, EC 4.2.1.11  (*Loxodonta africana*) | AAVPSGASTGIYEALELR | Unmodified | 2 | 1803.94 | 1.01 | 936470 | 94 |
| G3SWN6 | Lipocln_cytosolic_FA-bd_dom domain-containing protein  (*Loxodonta africana*) | PQEQLRKESYNLSWNK | 2 Deamidation (NQ),O-2H (Y) | 2 | 2034.96 | 4.25 | 616660 | 45 |
| G3SWY1 | Cytoplasmic linker associated protein 1  (*Loxodonta africana*) | MESCLAQVLQK | Oxidation (M),Trioxidation (C) | 2 | 1369.62 | 4.48 | 1155100 | 46 |
| G3SX77 | Uncharacterized protein  (*Loxodonta africana*) | VQDIEQLKDVK | Acetyl (K),Deamidation (NQ) | 2 | 1356.71 | -1.04 | 3158200 | 96 |
| G3SX81 | Anterior gradient 2, protein disulphide isomerase family member  (*Loxodonta africana*) | GWGDQLFWIQTYEEALYK | 2 Deamidation (NQ),Oxidation Y | 2 | 2264.03 | 4.15 | 505570 | 41 |
| G3SX89 | Transmembrane and coiled-coil domai...  (*Loxodonta africana*) | RQWVLNNCR | Deamidation (NQ),Oxidation (W) | 2 | 1261.60 | 3.92 | 186340 | 44 |
| G3SXH4 | REST corepressor 2  (*Loxodonta africana*) | KEVQVSQYR | dioxidation Y | 2 | 1167.59 | -1.12 | 1373000 | 82 |
| G3SXP1 | Histone H2A  (*Loxodonta africana*) | AGLQFPVGR | Unmodified | 2 | 943.52 | 0.17 | 3208100 | 111 |
| G3SXR5 | Semaphorin 6D  (*Loxodonta africana*) | GRPSGNESQHR | 2 Deamidation (NQ) | 2 | 1225.54 | -1.25 | 2899400 | 63 |
| G3SXR7 | Protein tyrosine phosphatase 4A3  (*Loxodonta africana*) | PAPVEVSYKNMR | Acetyl (K),Oxidation (M) | 2 | 1447.71 | -3.92 | 713370 | 69 |
| G3SXV3 | Cytochrome c oxidase polypeptide Va  (*Loxodonta africana*) | LNDFASAVR | Unmodified | 2 | 991.51 | 0.89 | 444380 | 127 |
| G3SXV8 | ASXL transcriptional regulator 1  (*Loxodonta africana*) | IPESLLLASTEYQPR | Oxidation (P) | 3 | 1731.90 | -2.11 | 897320 | 44 |
| G3SY10 | Collagen type V alpha 2 chain  (*Loxodonta africana*) | GDPGTVGPPGTVGER | 2 Oxidation (P) | 2 | 1426.67 | 0.11 | 1254300 | 112 |
| G3SY73 | SEH1 like nucleoporin  (*Loxodonta africana*) | LWKANYMDNWK | Acetyl (K),Oxidation (M) | 2 | 1525.70 | -0.84 | 294210 | 50 |
| G3SY76 | TNF alpha induced protein 1  (*Loxodonta africana*) | YYLIQGLVNMCQSALQDK | Unmodified | 3 | 2143.04 | -3.10 | 3328500 | 51 |
| G3SYC8 | Troponin T2, cardiac type  (*Loxodonta africana*) | EAEDGQVEESKPKPR | Glu->pyro-Glu,Deamidation (NQ) | 2 | 1680.80 | -1.31 | 623020 | 48 |
| G3SYG0 | Zinc finger protein 318  (*Loxodonta africana*) | SLLQNPQDKPVK | Oxidation (P) | 3 | 1381.76 | -0.07 | 2511500 | 85 |
| G3SYL8 | Component of oligomeric Golgi compl...  (*Loxodonta africana*) | LCGEEKVVER | Acetyl (K),Trioxidation (C) | 2 | 1307.60 | -3.19 | 1203900 | 73 |
| G3SYU6 | Nuclear RNA export factor 1  (*Loxodonta africana*) | GPFRWNYGEGNRR | Deamidation (NQ),Oxidation (W) | 2 | 1624.75 | -2.96 | 1466400 | 48 |
| G3SZ07 | DIS3-like exonuclease 1  (*Loxodonta africana*) | GIIFMQTACQAVQHQR | Oxidation (M),2 Deamidation (NQ) | 2 | 1904.89 | 0.17 | 857280 | 49 |
| G3SZ60 | FYN binding protein 2  (*Loxodonta africana*) | FSSQKSETSPAILLANR | Deamidation (NQ),Oxidation (P) | 2 | 1864.95 | 0.70 | 750200 | 53 |
| G3SZ65 | SEC14 like lipid binding 5  (*Loxodonta africana*) | QYQVDSLLQTWR | O-2H (W) | 3 | 1549.75 | -3.22 | 173440 | 49 |
| G3SZ93 | Pyroglutamylated RFamide peptide  (*Loxodonta africana*) | TSWAHLAQWHR | Deamidation (NQ),Di-oxidation W | 2 | 1424.66 | -1.81 | 2021500 | 51 |
| G3SZB0 | Uncharacterized protein  (*Loxodonta africana*) | AEEKIMNTWYPK | di-oxidation (M) | 2 | 1540.72 | 4.37 | 2713600 | 69 |
| G3SZD5 | Kinesin-like protein  (*Loxodonta africana*) | LLKDSIGGNCR | Acetyl (K),Deamidation (NQ),Trioxidation (C) | 2 | 1322.61 | -2.47 | 484990 | 48 |
| G3T038 | Serpin family B member 9  (*Loxodonta africana*) | MPFKINQKEQK | Acetyl (K),Oxidation (M),Deamidation (NQ) | 3 | 1448.73 | 2.49 | 12757000 | 63 |
| G3T087 | Protocadherin gamma subfamily C, 5  (*Loxodonta africana*) | QSSPNLQVSSDGTLK | Deamidation (NQ),Oxidation (P) | 2 | 1576.76 | -3.19 | 703830 | 75 |
| G3T094 | HYDIN axonemal central pair apparat...  (*Loxodonta africana*) | GPNGGAVDPNTR | Deamidation (NQ) | 2 | 1154.53 | -1.53 | 152940 | 48 |
| G3T096 | Tudor domain containing 9  (*Loxodonta africana*) | LRNARVNVDFQK | 3 Deamidation (NQ) | 2 | 1461.76 | -1.49 | 609510 | 46 |
| G3T0C4 | Solute carrier family 35 member E4  (*Loxodonta africana*) | RGLWWRDQPGK | Deamidation (NQ) | 2 | 1398.72 | 1.46 | 886790 | 55 |
| G3T0F1 | UBA domain containing  2  (*Loxodonta africana*) | QRQGGMINWNR | Deamidation (NQ),Di-oxidation W | 2 | 1391.64 | -2.73 | 276140 | 40 |
| G3T0H6 | Olfactory receptor  (*Loxodonta africana*) | EMKTAMRR | Acetyl (K),2 di-oxidation (M) | 2 | 1127.51 | -1.32 | 827760 | 76 |
| G3T0I2 | Zinc finger protein 292  (*Loxodonta africana*) | NWQAYMQYCVLCDKEFLGHR | 3 Deamidation (NQ) | 3 | 2620.12 | 1.31 | 793800 | 54 |
| G3T0L2 | cGMP-dependent protein kinase  (*Loxodonta africana*) | RLDPQQIK | Deamidation (NQ),Oxidation (P) | 2 | 1013.55 | -0.28 | 2143200 | 92 |
| G3T0M4 | Glomulin, FKBP associated protein  (*Loxodonta africana*) | LDSQGKYTLFR | O-2H (Y) | 2 | 1340.67 | -2.82 | 50365000 | 74 |
| G3T0P8 | Pleckstrin homology and RhoGEF doma...  (*Loxodonta africana*) | RESLSYIPK | O-2H (Y) | 2 | 1105.58 | 0.05 | 4127500 | 59 |
| G3T0R8 | DDRGK domain-containing protein 1  (*Loxodonta africana*) | VSITELAQASNSLIAWGR | Unmodified | 3 | 1915.02 | -1.55 | 3935400 | 51 |
| G3T0U0 | Transient receptor potential cation...  (*Loxodonta africana*) | QPQSPKAPAPQPPPVLK | 2 Deamidation (NQ),2 Oxidation (P) | 2 | 1812.96 | 1.80 | 711830 | 45 |
| G3T0U1 | Terminal uridylyl transferase 7  (*Loxodonta africana*) | EIQNKCTER | Acetyl (K),Glu->pyro-Glu | 2 | 1200.56 | -1.22 | 10674000 | 76 |
| G3T0U4 | Polypeptide N-acetylgalactosaminylt...  (*Loxodonta africana*) | GPSRVLEPEFRAHR | 2 Oxidation (P) | 2 | 1681.86 | 0.60 | 1913200 | 100 |
| G3T0V6 | Alpha-crystallin A chain  (*Loxodonta africana*) | NSENIFSEKLK | Deamidation (NQ) | 2 | 1308.66 | 0.18 | 328550 | 55 |
| G3T0Y6 | N-acetylated alpha-linked acidic di...  (*Loxodonta africana*) | KIRMHVHNTNR | Acetyl (K),2 Deamidation (NQ) | 2 | 1448.73 | 3.99 | 1514500 | 52 |
| G3T0Y7 | DEF6 guanine nucleotide exchange fa...  (*Loxodonta africana*) | HWNVQMNR | Oxidation (M),Di-oxidation W | 2 | 1131.49 | 4.37 | 1129800 | 72 |
| G3T118 | Tryptophan 2,3-dioxygenase  (*Loxodonta africana*) | VPYNRRHYR | 2 Oxidation Y | 2 | 1291.65 | -3.00 | 818720 | 44 |
| G3T145 | Homeodomain interacting protein kin...  (*Loxodonta africana*) | REYIDLLKK | Iodination | 2 | 1302.58 | 2.83 | 2181100 | 56 |
| G3T149 | Unc-80 homolog, NALCN channel compl...  (*Loxodonta africana*) | SENKENETVEKR | Acetyl (K),2 Deamidation (NQ) | 2 | 1505.68 | 0.89 | 670210 | 65 |
| G3T193 | mRNA-capping enzyme  (*Loxodonta africana*) | EIINPRHEKMK | Glu->pyro-Glu,di-oxidation (M) | 2 | 1407.73 | -2.75 | 373090 | 82 |
| G3T1G7 | Ral GTPase activating protein catal...  (*Loxodonta africana*) | TQMQQGLISVAAR | Oxidation (M),2 Deamidation (NQ) | 2 | 1419.70 | 0.15 | 178640 | 57 |
| G3T1G8 | Melanin concentrating hormone recep...  (*Loxodonta africana*) | KINNMENTLK | Acetyl (K),2 Deamidation (NQ) | 2 | 1247.61 | -2.21 | 4272500 | 77 |
| G3T1H2 | Unconventional myosin-6  (*Loxodonta africana*) | STLMTREQIQK | Oxidation (M),2 Deamidation (NQ) | 2 | 1351.67 | -2.99 | 945710 | 50 |
| G3T1L5 | Protein inhibitor of activated STAT...  (*Loxodonta africana*) | FCLCETSCPQEDYFPPNLFVKVNGK | Deamidation (NQ),Oxidation (P) | 3 | 3065.36 | -1.01 | 3390500 | 44 |
| G3T1M1 | Uncharacterized protein  (*Loxodonta africana*) | PGAEKTGYRIVSGNEQGR | Deamidation (NQ),dioxidation Y | 2 | 1950.94 | 2.51 | 700880 | 47 |
| G3T1M9 | Retinoic acid receptor gamma  (*Loxodonta africana*) | DKNCIINKVTR | 2 Acetyl (K),Deamidation (NQ),Trioxidation (C) | 2 | 1492.72 | -3.83 | 2453700 | 51 |
| G3T1N6 | Cytokine receptor like factor 1  (*Loxodonta africana*) | DFLFQAKYQIR | 2 Deamidation (NQ),Oxidation Y | 2 | 1445.72 | -2.25 | 2514600 | 66 |
| G3T1V3 | Protein tyrosine phosphatase 4A2  (*Loxodonta africana*) | QLLYLEKYR | dioxidation Y,O-2H (Y) | 2 | 1270.66 | -2.16 | 844030 | 72 |
| G3T1W3 | Ig-like domain-containing protein  (*Loxodonta africana*) | KEVVLQYPK | dioxidation Y | 2 | 1134.63 | 2.16 | 635420 | 59 |
| G3T202 | Synaptic vesicle glycoprotein 2°  (*Loxodonta africana*) | GLDRVQDEYSRR | Deamidation (NQ),Oxidation Y | 2 | 1509.72 | 0.70 | 38353000 | 61 |
| G3T206 | COX assembly mitochondrial protein  (*Loxodonta africana*) | DSGVLMVVKCR | Acetyl (K),Trioxidation (C),di-oxidation (M) | 2 | 1384.63 | -3.32 | 826770 | 43 |
| G3T288 | DAZ interacting zinc finger protein...  (*Loxodonta africana*) | LQEQNELIITQR | 4 Deamidation (NQ) | 2 | 1487.74 | 0.27 | 1352000 | 69 |
| G3T2B8 | Uncharacterized protein  (*Loxodonta africana*) | SVVEQWKK | Trp->Kynurenine | 2 | 1006.54 | -0.91 | 15097000 | 78 |
| G3T2H3 | Cadherin 4  (*Loxodonta africana*) | AVDYELNR | Deamidation (NQ),dioxidation Y | 2 | 1011.45 | 0.76 | 835190 | 65 |
| G3T2L6 | Apoptosis inducing factor mitochond...  (*Loxodonta africana*) | LTPLVRTVCVRSPR | 2 Oxidation (P) | 2 | 1684.94 | -4.08 | 1054300 | 56 |
| G3T2U4 | FRAS1 related extracellular matrix ...  (*Loxodonta africana*) | LPQNGQLQLK | 2 Deamidation (NQ),Oxidation (P) | 2 | 1155.61 | -0.95 | 2380300 | 79 |
| G3T2U7 | Homeobox A4  (*Loxodonta africana*) | DHKLPNTKMR | di-oxidation (M) | 2 | 1270.65 | 4.20 | 844030 | 80 |
| G3T2Y2 | RB transcriptional corepressor like...  (*Loxodonta africana*) | GDLIQFYNSIYIK | dioxidation Y,O-2H (Y) | 2 | 1618.79 | -1.11 | 2164800 | 76 |
| G3T345 | Suppressor of tumorigenicity 14 pro...  (*Loxodonta africana*) | LLPQQLTAR | Oxidation (P) | 2 | 1054.61 | 0.01 | 5954700 | 125 |
| G3T357 | Contactin 1  (*Loxodonta africana*) | AVDLIPWMEYEFR | Oxidation (M),Di-oxidation W | 2 | 1715.79 | -0.30 | 869020 | 44 |
| G3T3G4 | Uncharacterized protein  (*Loxodonta africana*) | PGPPGPPGK | 3 Oxidation (P) | 1 | 850.42 | -0.34 | 750860 | 55 |
| G3T3I4 | Uncharacterized protein  (*Loxodonta africana*) | QLKEEWEQQQRK | Deamidation (NQ) | 2 | 1629.81 | 0.90 | 318990 | 49 |
| G3T3M6 | Protein FAM32A  (*Loxodonta africana*) | AKLLEAMGTSKK | Acetyl (K),Oxidation (M) | 2 | 1333.73 | -1.95 | 609300 | 55 |
| G3T3Q1 | Vacuolar protein sorting 13 homolog...  (*Loxodonta africana*) | ERLSEQQYNR | 2 Deamidation (NQ),Oxidation Y | 2 | 1339.60 | 0.77 | 1458700 | 63 |
| G3T3S6 | Sidekick cell adhesion molecule 2  (*Loxodonta africana*) | VQVQAFNAIGSGPWSQTVVGR | 2 Deamidation (NQ),Trp->Kynurenine | 2 | 2206.10 | -0.69 | 1951100 | 46 |
| G3T3W3 | Exocyst complex component 3 like 4  (*Loxodonta africana*) | EPAQQQTPAR | 3 Deamidation (NQ) | 2 | 1127.51 | -1.17 | 827760 | 59 |
| G3T475 | Aldo-keto reductase family 1 member...  (*Loxodonta africana*) | QEPEMVRPTLEK | di-oxidation (M) | 2 | 1487.73 | -3.32 | 488880 | 74 |
| G3T486 | Adhesion G protein-coupled receptor...  (*Loxodonta africana*) | DGIPGQRNDGNKTK | 2 Deamidation (NQ) | 2 | 1500.72 | 1.93 | 3078900 | 54 |
| G3T4A1 | Uridine-cytidine kinase  (*Loxodonta africana*) | PGEKSDPACEDR | Unmodified | 2 | 1359.57 | -2.14 | 481390 | 69 |
| G3T4P1 | Uncharacterized protein  (*Loxodonta africana*) | CNEIESHIIK | Deamidation (NQ),Trioxidation (C) | 2 | 1290.58 | 3.60 | 726990 | 53 |
| G3T4R7 | NTP_transf_2 domain-containing prot...  (*Loxodonta africana*) | KTLKQLLCR | 2 Acetyl (K),Trioxidation (C) | 2 | 1290.70 | -2.29 | 737170 | 62 |
| G3T4X4 | Receptor interacting serine/threoni...  (*Loxodonta africana*) | ENLGRHWK | Deamidation (NQ),Oxidation (W) | 2 | 1055.51 | -2.73 | 1803500 | 83 |
| G3T507 | Uncharacterized protein  (*Loxodonta africana*) | GNLWATGHFMGK | Oxidation (M),Deamidation (NQ),O-2H (W) | 2 | 1348.59 | 0.97 | 756680 | 44 |
| G3T572 | Midasin  (*Loxodonta africana*) | KKAEYFCQLYK | dioxidation Y,O-2H (Y) | 2 | 1522.71 | 3.78 | 1284700 | 68 |
| G3T5B6 | Apolipoprotein A5  (*Loxodonta africana*) | VEQIQQQK | 3 Deamidation (NQ) | 2 | 1002.49 | -0.74 | 2760600 | 76 |
| G3T5B7 | ABO, alpha 1-3-N-acetylgalactosamin...  (*Loxodonta africana*) | MMYPQPKVLTLCR | Oxidation (M),Trioxidation (C) | 2 | 1699.81 | -4.16 | 495430 | 58 |
| G3T5D2 | G_PROTEIN_RECEP_F1_2 domain-contain...  (*Loxodonta africana*) | GPFEGPNYHIAPR | Deamidation (NQ),dioxidation Y | 2 | 1486.68 | -2.50 | 5418000 | 45 |
| G3T5E7 | Small glutamine rich tetratricopept...  (*Loxodonta africana*) | AYGRMGLALTAMNK | Oxidation (M),Deamidation (NQ),di-oxidation (M) | 2 | 1544.73 | 3.12 | 1205200 | 61 |
| G3T5P9 | PPFIA binding protein 1  (*Loxodonta africana*) | LTFSNFGNLRKK | 2 Deamidation (NQ) | 2 | 1425.76 | -0.47 | 789720 | 47 |
| G3T5R1 | Suppressor of IKBKE 1  (*Loxodonta africana*) | QMLQLMVAKK | Gln->pyro-Glu | 2 | 1171.65 | -0.01 | 1828900 | 73 |
| G3T5T6 | Family with sequence similarity 207 member A  (*Loxodonta africana*) | LRREQWLQK | Deamidation (NQ),O-2H (W) | 2 | 1270.68 | -1.53 | 1952100 | 85 |
| G3T5T9 | Transmembrane protein 169  (*Loxodonta africana*) | AVAAALALNGESTMSR | Oxidation (M) | 2 | 1576.79 | -1.06 | 8193800 | 79 |
| G3T5V9 | Sorting nexin  (*Loxodonta africana*) | LSDLLKYYLR | 2 Oxidation Y | 2 | 1314.72 | -1.49 | 575210 | 98 |
| G3T5W4 | Bromodomain adjacent to zinc finger domain 1A  (*Loxodonta africana*) | LSNPSLVK | Deamidation (NQ),Oxidation (P) | 1 | 873.48 | 0.99 | 596480 | 74 |
| G3T6A4 | IF rod domain-containing protein  (*Loxodonta africana*) | APSAYGGLSVTSSR | Unmodified | 2 | 1351.67 | 1.63 | 954470 | 123 |
| G3T6C7 | Cytohesin 3  (*Loxodonta africana*) | DKPTAERFITMNR | Acetyl (K),Oxidation (M) | 2 | 1635.80 | -3.42 | 1479300 | 67 |
| G3T6D0 | Uncharacterized protein  (*Loxodonta africana*) | SLEPLMEMNKR | Oxidation (M) | 2 | 1362.66 | 0.10 | 1195200 | 76 |
| G3T6N6 | Methylenetetrahydrofolate reductase, EC 1.5.1.20  (*Loxodonta africana*) | RLGMWIEDPRR | Trp->Kynurenine | 2 | 1431.74 | -3.34 | 2544400 | 43 |
| G3T6U4 | Stabilizer of axonemal microtubules 1  (*Loxodonta africana*) | GLVNTMSCK | Oxidation (M) | 1 | 1024.47 | 0.48 | 279120 | 48 |
| G3T6W1 | DUF3715 domain-containing protein  (*Loxodonta africana*) | ALDPTPKHECHVSK | Oxidation (P) | 2 | 1633.79 | -2.47 | 19527000 | 101 |
| G3T748 | Transmembrane serine protease 12  (*Loxodonta africana*) | KAVRYNDYIR | dioxidation Y,O-2H (Y) | 2 | 1342.66 | -0.18 | 10551000 | 58 |
| G3T7C9 | WAS/WASL interacting protein family member 2  (*Loxodonta africana*) | GSSGGYGSGAAALQPK | Deamidation (NQ),Oxidation Y | 2 | 1423.66 | -0.21 | 1166200 | 72 |
| G3T7E0 | Nucleoside-diphosphate kinase, EC 2.7.4.6  (*Loxodonta africana*) | MNTNDAKEYLTR | 2 Deamidation (NQ) | 2 | 1456.65 | -2.51 | 600150 | 41 |
| G3T7I0 | Non-specific serine/threonine protein kinase, EC 2.7.11.1  (*Loxodonta africana*) | SEPPALAQPSR | Deamidation (NQ),2 Oxidation (P) | 2 | 1184.57 | 1.00 | 17507000 | 91 |
| G3T7P5 | T-box brain transcription factor 1  (*Loxodonta africana*) | DNYDTIYTGCDMDR | Oxidation (M),Iodination,Di-iodination | 3 | 2131.35 | 2.36 | 14131000 | 41 |
| G3T7S2 | Collagen IV NC1 domain-containing protein  (*Loxodonta africana*) | GSPGPPGMK | Oxidation (M) | 1 | 842.40 | -3.52 | 699710 | 49 |
| G3T7Y3 | Glutamate receptor  (*Loxodonta africana*) | PAPATNTQNYATYR | 2 Deamidation (NQ),dioxidation Y | 2 | 1600.70 | 1.93 | 858500 | 49 |
| G3T802 | Uncharacterized protein  (*Loxodonta africana*) | GLQNPVPQQMLSNSR | Oxidation (M),Deamidation (NQ),Oxidation (P) | 2 | 1700.82 | -1.40 | 484860 | 60 |
| G3T882 | Galactosylgalactosylxylosylprotein 3-beta-  glucuronosyltransferase, EC 2.4.1.135  (*Loxodonta africana*) | PFAIDMAGFAVNLR | Oxidation (M),Deamidation (NQ),Oxidation (P) | 2 | 1553.75 | -2.41 | 580630 | 61 |
| G3T8A6 | Uncharacterized protein  (*Loxodonta africana*) | KNNPEPWNRVAPNEQYK | Acetyl (K),3 Deamidation (NQ) | 3 | 2127.99 | -1.04 | 4179700 | 54 |
| G3T8C8 | Uncharacterized protein  (*Loxodonta africana*) | TALEQVERERR | Deamidation (NQ) | 2 | 1386.72 | 2.32 | 1613900 | 68 |
| G3T8D8 | Serine palmitoyltransferase long chain base subunit 1  (*Loxodonta africana*) | EQEIEDQKNPR | Glu->pyro-Glu | 2 | 1366.65 | 2.90 | 1823300 | 88 |
| G3T8E6 | BCL2 related protein A1  (*Loxodonta africana*) | QNGGWENGFVKK | 2 Deamidation (NQ),Di-oxidation W | 2 | 1396.63 | -3.43 | 672140 | 44 |
| G3T8P2 | Arsenite methyltransferase  (*Loxodonta africana*) | QQVLQEVYR | Gln->pyro-Glu,Oxidation Y | 2 | 1160.58 | 0.45 | 1486800 | 46 |
| G3T8R2 | Rho GTPase activating protein 24  (*Loxodonta africana*) | RSERGNTIWIQ | 2 Deamidation (NQ),Trp->Kynurenine | 2 | 1364.67 | 2.87 | 1163600 | 53 |
| G3T8R9 | RAD17 checkpoint clamp loader component  (*Loxodonta africana*) | NQAQISFIQDIGR | 2 Deamidation (NQ) | 2 | 1490.74 | -0.07 | 2789900 | 84 |
| G3T8S5 | Stork_head domain-containing protein  (*Loxodonta africana*) | INPDLTVENVMR | Oxidation (M),Deamidation (NQ) | 2 | 1416.69 | 3.48 | 1213000 | 44 |
| G3T8X3 | Mitochondrial ribosomal protein L3  (*Loxodonta africana*) | VWPGTKMPGQTGNR | Acetyl (K),Oxidation (M) | 2 | 1585.77 | 2.90 | 1080500 | 64 |
| G3T8Z5 | Formylglycinamide ribonucleotide amidotransferase, EC 6.3.5.3 (Formylglycinamide ribotide amidotransferase)  (*Loxodonta africana*) | GDPEMEQKMNRVIR | 2 Deamidation (NQ),di-oxidation (M) | 2 | 1735.79 | -2.71 | 235850 | 52 |
| G3T938 | Cyclin dependent kinase 16  (*Loxodonta africana*) | LGEGTYATVYKGKSK | 2 Oxidation Y | 2 | 1632.84 | -4.27 | 1181200 | 49 |
| G3T969 | Tubulin beta chain  (*Loxodonta africana*) | AILVDLEPGTMDSVR | Oxidation (M) | 2 | 1630.82 | -0.63 | 1104800 | 79 |
| G3T975 | Mediator of RNA polymerase II transcription subunit 13  (*Loxodonta africana*) | KYDKQMAVPSR | Oxidation (M),O-2H (Y) | 2 | 1351.66 | -0.49 | 280140 | 55 |
| G3T9E2 | Cilia and flagella associated protein 94 (Dynein  intermediate chain CFAP94, axonemal)  (*Loxodonta africana*) | IEKEKWQQLEAK | 2 Deamidation (NQ),Di-oxidation W | 2 | 1562.78 | -3.67 | 1146500 | 84 |
| G3T9F8 | Troponin I2, fast skeletal type  (*Loxodonta africana*) | VQKSTKELEDMNQK | Acetyl (K),Oxidation (M),2 Deamidation (NQ) | 2 | 1736.81 | -3.20 | 847620 | 57 |
| G3T9G5 | E3 ubiquitin-protein ligase, EC 2.3.2.26  (*Loxodonta africana*) | LRNPEVRGALQTPQNR | 2 Deamidation (NQ),Oxidation (P) | 3 | 1865.97 | -3.17 | 4061000 | 90 |
| G3T9R8 | Actin gamma 2, smooth muscle  (*Loxodonta africana*) | GYSFVTTAER | Unmodified | 2 | 1129.54 | -0.44 | 10945000 | 186 |
| G3T9S0 | Receptor tyrosine kinase like orphan receptor 1  (*Loxodonta africana*) | ISDLGLSR | Unmodified | 2 | 859.48 | 1.21 | 4044100 | 128 |
| G3T9X8 | Cell adhesion molecule 4  (*Loxodonta africana*) | KELKGVSSQQENGK | 2 Deamidation (NQ) | 2 | 1532.77 | 1.47 | 430840 | 83 |
| G3TA02 | GlcNAc kinase, EC 2.7.1.59 (N-acetyl-D-  glucosamine kinase)  (*Loxodonta africana*) | IAEGAQQGDPLSR | 2 Deamidation (NQ),Oxidation (P) | 2 | 1358.63 | 0.46 | 1060700 | 63 |
| G3TA45 | CTR9 homolog, Paf1/RNA polymerase II complex component  (*Loxodonta africana*) | AYPNNYETMK | Oxidation (M),Deamidation (NQ),Oxidation (P) | 2 | 1262.51 | 3.46 | 956500 | 57 |
| G3TA46 | DDB1- and CUL4-associated factor 11  (*Loxodonta africana*) | RTMREDDPK | Unmodified | 2 | 1146.55 | -1.64 | 1856100 | 85 |
| G3TA80 | Cholinergic receptor nicotinic alpha 5 subunit  (*Loxodonta africana*) | NTLEAALDSIRYITRHVMK | Unmodified | 3 | 2230.19 | -0.20 | 869220 | 59 |
| G3TAB4 | Zinc finger and BTB domain containing 41  (*Loxodonta africana*) | KILQCPKCDK | Unmodified | 2 | 1288.66 | 0.30 | 27334000 | 96 |
| G3TAH7 | Isoleucyl-tRNA synthetase, EC 6.1.1.5  (*Loxodonta africana*) | ENGAFTVLVDNYVR | Glu->pyro-Glu,O-2H (Y) | 2 | 1591.76 | -0.51 | 1228800 | 61 |
| G3TAI1 | UTP6 small subunit processome component  (*Loxodonta africana*) | QWIELLLHR | Deamidation (NQ),Di-oxidation W | 3 | 1239.66 | 3.80 | 476420 | 54 |
| G3TAI7 | Uncharacterized protein  (*Loxodonta africana*) | RQWPVPYK | dioxidation Y | 2 | 1104.57 | -3.08 | 64427000 | 77 |
| G3TAK1 | Complement C5  (*Loxodonta africana*) | YILSPYKLNLVATSLFLK | Deamidation (NQ),Iodination,Oxidation Y | 3 | 2225.09 | 3.16 | 2610900 | 75 |
| G3TAQ7 | Uncharacterized protein  (*Loxodonta africana*) | EGKESKEVR | 2 Acetyl (K),Glu->pyro-Glu | 2 | 1126.56 | -0.65 | 396470 | 68 |
| G3TAT5 | PTH/PTHrP type I receptor (Parathyroid hormone 1 receptor) (Parathyroid hormone/parathyroid hormone-related peptide receptor)  (*Loxodonta africana*) | ATLANTGCWDLSSGNK | 2 Deamidation (NQ) | 2 | 1695.74 | -4.44 | 825700 | 50 |
| G3TAY5 | Tripartite motif containing 65  (*Loxodonta africana*) | QNQQVKHCRLPR | Gln->pyro-Glu | 2 | 1545.79 | -2.16 | 3432500 | 49 |
| G3TB38 | Uncharacterized protein  (*Loxodonta africana*) | DYVPLNTIPPWKEEMK | Acetyl (K),Oxidation (M) | 3 | 2016.99 | 0.45 | 4139800 | 54 |
| G3TB66 | E3 ubiquitin-protein ligase, EC 2.3.2.26  (*Loxodonta africana*) | YTSEGVRYFVDHNTR | 2 dioxidation Y | 2 | 1906.84 | 1.00 | 428820 | 52 |
| G3TB93 | Synaptotagmin 6  (*Loxodonta africana*) | SCGKINFSLR | Acetyl (K),Deamidation (NQ),Trioxidation (C) | 2 | 1271.58 | 4.48 | 895890 | 60 |
| G3TB94 | Small nuclear RNA activating complex polypeptide 1  (*Loxodonta africana*) | GDMQNGNKEAK | Deamidation (NQ) | 2 | 1191.52 | 0.85 | 219380 | 84 |
| G3TBC0 | Zinc finger protein 521  (*Loxodonta africana*) | PGLGQNENLSAIEGKGK | Acetyl (K),3 Deamidation (NQ) | 2 | 1755.85 | 1.87 | 62387000 | 83 |
| G3TBC2 | Hexosyltransferase, EC 2.4.1.-  (*Loxodonta africana*) | LGIHPFQNSGFNHWK | Di-oxidation W | 3 | 1812.87 | -2.95 | 1135200 | 42 |
| G3TBD6 | Collagen type VI alpha 3 chain  (*Loxodonta africana*) | VAVVTYNNEVTTEIR | Deamidation (NQ) | 2 | 1707.87 | -0.01 | 313730 | 60 |
| G3TBH2 | Cytoplasmic protein  (*Loxodonta africana*) | LWLLDDSKSWWR | O-2H (W),Oxidation (W) | 2 | 1633.79 | -1.06 | 19527000 | 71 |
| G3TBJ2 | Diaphanous related formin 2  (*Loxodonta africana*) | TMLPDLKEK | Acetyl (K) | 2 | 1115.59 | 0.94 | 2490400 | 95 |
| G3TBK4 | RB transcriptional corepressor 1  (*Loxodonta africana*) | LGVRLYYR | dioxidation Y,O-2H (Y) | 2 | 1084.57 | -4.44 | 1079300 | 50 |
| G3TBR2 | N-glycanase 1, EC 3.5.1.52 (Peptide-N(4)-(N-acetyl-beta-glucosaminyl)asparagine amidase) (Peptide:N-glycanase)  (*Loxodonta africana*) | VETDWNMVYLAR | Oxidation (M),Trp->Kynurenine | 2 | 1515.70 | 3.46 | 23706000 | 57 |
| G3TBT4 | Protein Wnt  (*Loxodonta africana*) | GPPGEGWK | Oxidation (P) | 1 | 842.39 | 0.90 | 699710 | 61 |
| G3TBX7 | Gap junction protein  (*Loxodonta africana*) | IHRELRMLEEQK | Oxidation (M),Deamidation (NQ) | 3 | 1597.82 | 1.29 | 568460 | 44 |
| G3TBZ9 | Tetratricopeptide repeat protein 39B  (*Loxodonta africana*) | DLSENLLVTVEK | Unmodified | 2 | 1358.73 | -0.66 | 2075600 | 96 |
| G3TC80 | Ring finger protein 113°  (*Loxodonta africana*) | ATVRWDYQPDICKDYK | Deamidation (NQ),dioxidation Y | 2 | 2089.94 | 2.80 | 1088700 | 65 |
| G3TCC2 | Uncharacterized protein  (*Loxodonta africana*) | LPDYYTIIK | 2 O-2H (Y) | 2 | 1152.57 | -2.72 | 1647500 | 78 |
| G3TCF4 | Stromal antigen 3  (*Loxodonta africana*) | ERMVSMVMDR | Oxidation (M),di-oxidation (M) | 2 | 1300.56 | -0.68 | 1296300 | 51 |
| G3TCI4 | Uncharacterized protein  (*Loxodonta africana*) | ERWEKTSETFK | O-2H (W) | 3 | 1453.68 | 0.62 | 825140 | 73 |
| G3TCJ7 | Lon protease homolog 2, peroxisomal, EC 3.4.21.- (Lon protease-like protein 2, Lon protease 2) (Peroxisomal Lon protease)  (*Loxodonta africana*) | IQTSSMPEQAHKVCVKEIK | Deamidation (NQ),Oxidation (P) | 3 | 2229.11 | -0.15 | 594230 | 41 |
| G3TCQ1 | DNA-(apurinic or apyrimidinic site) lyase, EC 4.2.99.18  (*Loxodonta africana*) | IGPNNGRNFFVCPLGKEK | Deamidation (NQ),2 Oxidation (P) | 2 | 2079.02 | 3.61 | 546700 | 51 |
| G3TCU3 | C3/C5 convertase, EC 3.4.21.47 (Complement factor B) (Complement factor B Ba fragment) (Complement factor B Bb fragment)  (*Loxodonta africana*) | DAGGTVKWQER | Deamidation (NQ),Oxidation (W) | 2 | 1262.59 | -0.52 | 1965700 | 65 |
| G3TCW0 | Nucleolar and coiled-body phosphoprotein 1  (*Loxodonta africana*) | TLQKAAGTVTPSKPASK | Deamidation (NQ) | 2 | 1684.94 | -0.18 | 1024300 | 72 |
| G3TD02 | Dual adaptor of phosphotyrosine and 3-phosphoinositides 1  (*Loxodonta africana*) | QGGLVKTWK | Di-oxidation W | 2 | 1047.57 | -0.96 | 2637000 | 66 |
| G3TD03 | Pleckstrin homology domain containing A5  (*Loxodonta africana*) | QDSTGMKLWK | Acetyl (K),Oxidation (M),Gln->pyro-Glu | 2 | 1233.57 | 3.22 | 268290 | 46 |
| G3TD91 | Activated leukocyte cell adhesion molecule (CD166 antigen)  (*Loxodonta africana*) | DLGNLEENKK | 2 Deamidation (NQ) | 2 | 1160.56 | 1.60 | 13993000 | 64 |
| G3TDD7 | Uncharacterized protein  (*Loxodonta africana*) | EELQMKISDLITGATR | Oxidation (M),Deamidation (NQ) | 2 | 1820.92 | -2.25 | 441980 | 60 |
| G3TDF5 | Breast cancer type 1 susceptibility protein homolog, EC 2.3.2.27  (*Loxodonta africana*) | GPSQCPLCKNHITK | Unmodified | 3 | 1638.80 | -1.76 | 2081400 | 84 |
| G3TDK6 | 5'-3' exoribonuclease, EC 3.1.13.-  (*Loxodonta africana*) | PNKPKPCGLCNQFGHEVK | Deamidation (NQ),2 Oxidation (P) | 3 | 2142.00 | 1.41 | 3645800 | 49 |
| G3TDN7 | Stress induced phosphoprotein 1  (*Loxodonta africana*) | KCQQAEKILK | Deamidation (NQ),Trioxidation (C) | 2 | 1293.66 | -1.51 | 2707500 | 110 |
| G3TDW1 | CWC22 spliceosome associated protein homolog  (*Loxodonta africana*) | LRMMQEQITDK | Oxidation (M),Deamidation (NQ) | 2 | 1408.67 | 0.70 | 1332600 | 62 |
| G3TDW4 | Ro60, Y RNA binding protein  (*Loxodonta africana*) | ESMKCGMWGR | Oxidation (M),Trioxidation (C) | 2 | 1304.49 | -3.84 | 14193000 | 79 |
| G3TE05 | Acid phosphatase 6, lysophosphatidic  (*Loxodonta africana*) | AVDTALYMLQR | Oxidation (M),O-2H (Y) | 2 | 1309.63 | -0.94 | 2159200 | 99 |
| G3TE42 | Sperm associated antigen 1  (*Loxodonta africana*) | GLKDYQKSLNDLNK | 2 Deamidation (NQ),dioxidation Y | 3 | 1668.82 | 2.23 | 2053600 | 52 |
| G3TE74 | Rubicon like autophagy enhancer  (*Loxodonta africana*) | KYYVSNFAK | dioxidation Y | 2 | 1150.57 | -2.76 | 979040 | 56 |
| G3TE81 | Sphingosine kinase 2  (*Loxodonta africana*) | ADGAAAYEENRAEAQR | Oxidation Y | 3 | 1736.77 | -2.18 | 2096800 | 53 |
| G3TEA6 | Ubiquitin carboxyl-terminal hydrolase, EC 3.4.19.12  (*Loxodonta africana*) | PGEGPEAEPGSPRVR | Oxidation (P) | 2 | 1549.75 | 0.35 | 435510 | 67 |
| G3TEC1 | Collagen type IX alpha 1 chain  (*Loxodonta africana*) | GPPGPRGVQGQQGATGLPGIQGPPGR | 3 Deamidation (NQ),2 Oxidation (P) | 3 | 2470.22 | -3.83 | 9279500 | 65 |
| G3TEG7 | Proteasome assembly chaperone 1  (*Loxodonta africana*) | NIPQSTEMLKKLMVTNEIQSNIYT | Acetyl (K),Oxidation (M),Deamidation (NQ) | 3 | 2853.41 | -1.18 | 2863800 | 48 |
| G3TEH4 | Dolichyl-phosphate-mannose--protein mannosyltransferase, EC 2.4.1.109  (*Loxodonta africana*) | GDILMNQKK | Acetyl (K),Deamidation (NQ),di-oxidation (M) | 2 | 1120.54 | -3.23 | 227160 | 65 |
| G3TEL8 | Sulfotransferase, EC 2.8.2.-  (*Loxodonta africana*) | DKGLPLPQDCRDFLHSLR | Deamidation (NQ) | 3 | 2167.08 | 2.19 | 1235800 | 46 |
| G3TEQ6 | Calcium-transporting ATPase, EC 7.2.2.10  (*Loxodonta africana*) | EAVFGKNFIPPKK | Deamidation (NQ),Oxidation (P) | 2 | 1490.81 | -2.69 | 975500 | 41 |
| G3TEW0 | Cor1 domain-containing protein  (*Loxodonta africana*) | TLYEQFLK | Deamidation (NQ),O-2H (Y) | 2 | 1055.52 | -2.95 | 2051700 | 85 |
| G3TEW2 | Cysteine dioxygenase, EC 1.13.11.20  (*Loxodonta africana*) | NKVIMTFYSK | Acetyl (K),Oxidation (M),Deamidation (NQ) | 2 | 1288.64 | 3.18 | 235360 | 55 |
| G3TEY6 | Serpin family F member 1  (*Loxodonta africana*) | LDLQEINNWVQAQMK | Oxidation (M),2 Deamidation (NQ) | 2 | 1846.88 | 3.61 | 920710 | 46 |
| G3TEZ7 | Histone acetyltransferase, EC 2.3.1.48  (*Loxodonta africana*) | EKEEQDMLSSRANSR | Oxidation (M) | 2 | 1794.82 | -3.88 | 535170 | 54 |
| G3TF15 | ADP ribosylation factor interacting protein 2  (*Loxodonta africana*) | KWGINTYK | Deamidation (NQ),O-2H (Y) | 2 | 1023.50 | -3.73 | 1839500 | 69 |
| G3TF22 | General transcription factor IIIC subunit 4  (*Loxodonta africana*) | QVNKQSFQER | Acetyl (K),3 Deamidation (NQ) | 2 | 1307.60 | -2.05 | 1269400 | 50 |
| G3TF53 | Microtubule-associated protein RP/EB family member 2  (*Loxodonta africana*) | PGSTPSRPSSAKR | 3 Oxidation (P) | 2 | 1374.69 | -0.20 | 1543100 | 62 |
| G3TF54 | 5'-3' exoribonuclease 1  (*Loxodonta africana*) | KYQINQNGEVQLEKQWSK | Deamidation (NQ),Oxidation Y | 3 | 2236.11 | -0.78 | 2158300 | 60 |
| G3TF88 | G_PROTEIN_RECEP_F3_4 domain-containing protein  (*Loxodonta africana*) | QDCPLQGNTGPVPGYQHSFR | Oxidation (P) | 3 | 2273.03 | 0.35 | 1433400 | 48 |
| G3TF94 | Phosphoinositide phospholipase C, EC 3.1.4.11  (*Loxodonta africana*) | KTVSFSSMPTEK | Oxidation (P) | 2 | 1356.66 | 3.41 | 610370 | 72 |
| G3TFC1 | KIAA1143  (*Loxodonta africana*) | MSKRNQVSYVR | Oxidation Y | 2 | 1382.71 | 4.41 | 9099500 | 80 |
| G3TFD9 | SWI/SNF related, matrix associated, actin dependent regulator of chromatin, subfamily e, member 1  (*Loxodonta africana*) | RQVQSLMVHQR | 2 Deamidation (NQ) | 2 | 1382.71 | 3.45 | 3973200 | 54 |
| G3TFK2 | Uncharacterized protein  (*Loxodonta africana*) | TNVALMCMLR | Oxidation (M),di-oxidation (M) | 2 | 1255.57 | 1.00 | 1409600 | 48 |
| G3TFT1 | Uncharacterized protein  (*Loxodonta africana*) | LAQEGAHVVISSRKQQNVDR | 4 Deamidation (NQ) | 3 | 2238.12 | 1.94 | 17240000 | 48 |
| G3TFY5 | Sushi, von Willebrand factor type A, EGF and pentraxin domain containing 1  (*Loxodonta africana*) | NQATCVDELNSYSCK | Deamidation (NQ) | 2 | 1788.73 | 2.90 | 1002100 | 49 |
| G3TG56 | Receptor associated protein of the synapse  (*Loxodonta africana*) | GLQLYQSNQTEKALQVWMK | Deamidation (NQ),Oxidation Y | 3 | 2281.14 | -3.54 | 1631100 | 52 |
| G3TG62 | Dysferlin  (*Loxodonta africana*) | AVDEQGWEYSITIPPDRKPR | Deamidation (NQ) | 3 | 2357.17 | 0.83 | 396730 | 54 |
| G3TG81 | ETS transcription factor ELK4  (*Loxodonta africana*) | NKPNMNYDKLSR | 2 Acetyl (K) | 2 | 1562.75 | -2.50 | 879450 | 45 |
| G3TGJ2 | Oxysterol-binding protein  (*Loxodonta africana*) | SLLPPTDTRFR | Oxidation (P) | 2 | 1317.70 | -0.13 | 240260 | 82 |
| G3TGJ4 | Peptidyl-prolyl cis-trans isomerase, PPIase, EC 5.2.1.8  (*Loxodonta africana*) | GPFADENFKLR | Deamidation (NQ),Oxidation (P) | 2 | 1309.63 | 0.69 | 2159200 | 76 |
| G3TGN5 | Sorting nexin 19  (*Loxodonta africana*) | QLEQEINHTIQMIIR | Deamidation (NQ) | 3 | 1865.97 | -3.41 | 4448500 | 91 |
| G3TGW6 | Polyamine modulated factor 1 binding protein 1  (*Loxodonta africana*) | LEKELAQNWDALKNK | Acetyl (K),Deamidation (NQ) | 3 | 1841.95 | -0.84 | 503700 | 64 |
| G3TGX6 | SUN domain containing ossification factor  (*Loxodonta africana*) | TIVKLQNTSR | Acetyl (K) | 3 | 1200.68 | -2.76 | 276350 | 54 |
| G3TGY4 | Interferon stimulated exonuclease gene 20 like 2  (*Loxodonta africana*) | QPPSRVARLHSDPPK | 3 Oxidation (P) | 3 | 1731.90 | 2.11 | 796770 | 50 |
| G3TGZ8 | CtBP-interacting protein (DNA endonuclease RBBP8)  (*Loxodonta africana*) | KPNSNGDKHDK | Acetyl (K),Deamidation (NQ) | 2 | 1281.59 | 4.31 | 268010 | 62 |
| G3TH05 | Dedicator of cytokinesis 10  (*Loxodonta africana*) | STQMEASALLYFFMR | Oxidation (M),O-2H (Y) | 2 | 1823.82 | -2.55 | 1153800 | 44 |
| G3TH42 | Septin  (*Loxodonta africana*) | RSLFNYHDTR | O-2H (Y) | 2 | 1321.62 | 1.04 | 1037800 | 60 |
| G3TH59 | Kinetochore associated 1  (*Loxodonta africana*) | QTLLTNAFVQKANDENERTYR | Unmodified | 3 | 2510.25 | -3.76 | 710420 | 50 |
| G3TH98 | AFG3 like matrix AAA peptidase subunit 2  (*Loxodonta africana*) | GLGYAQYLPK | 2 Oxidation Y | 2 | 1140.58 | -2.37 | 293640 | 57 |
| G3THI2 | Calcium-transporting ATPase, EC 7.2.2.10  (*Loxodonta africana*) | IGIFGENEEVAER | Unmodified | 2 | 1461.71 | -0.84 | 4969800 | 98 |
| G3THK7 | Activating transcription factor 7 interacting protein  (*Loxodonta africana*) | PGNDINSNNNVPYRNAGTVR | 2 Deamidation (NQ),dioxidation Y | 2 | 2205.00 | 2.94 | 755300 | 45 |
| G3THU7 | Testis specific serine kinase substrate  (*Loxodonta africana*) | QRGLAPACPSCQRLHK | 2 Trioxidation (C) | 2 | 1973.92 | -3.83 | 886990 | 48 |
| G3THV0 | BCL6 corepressor like 1  (*Loxodonta africana*) | VKQEGVSVFACK | Unmodified | 2 | 1350.70 | 3.48 | 440890 | 66 |
| G3THV5 | Homeostatic iron regulator  (*Loxodonta africana*) | WLKDKQLLEAK | Deamidation (NQ),Di-oxidation W | 2 | 1403.77 | 0.01 | 791360 | 62 |
| G3THW2 | Uncharacterized protein  (*Loxodonta africana*) | RFYPVQISSR | Iodination | 2 | 1377.57 | -0.47 | 1020300 | 68 |
| G3THW4 | Calreticulin  (*Loxodonta africana*) | IKDPYAQKPR | Deamidation (NQ),O-2H (Y) | 2 | 1229.64 | -1.06 | 1937800 | 74 |
| G3THY8 | RAB40C, member RAS oncogene family  (*Loxodonta africana*) | SFSMANGMNAVMMHGR | Deamidation (NQ),di-oxidation (M) | 2 | 1772.71 | 3.75 | 3707600 | 54 |
| G3TIC7 | Transformer 2 beta homolog  (*Loxodonta africana*) | RSPSPYYSRGGYR | 2 Oxidation (P) | 2 | 1576.74 | -0.05 | 1053600 | 46 |
| G3TII4 | ASXL transcriptional regulator 3  (*Loxodonta africana*) | NVSSSSPPEKEQPPR | Deamidation (NQ) | 3 | 1638.78 | 4.22 | 1649600 | 58 |
| G3TIP2 | Protein O-glucosyltransferase 1  (*Loxodonta africana*) | EDLARSAAQWPWK | Glu->pyro-Glu,Oxidation (W) | 2 | 1554.76 | 2.42 | 653870 | 45 |
| G3TIQ5 | Polypyrimidine tract binding protein 2  (*Loxodonta africana*) | NQPIYIQYSNHK | 2 Deamidation (NQ) | 2 | 1505.72 | -3.35 | 6611900 | 70 |
| G3TIS1 | Kinesin-like protein  (*Loxodonta africana*) | NTVCVNVELTAEQWK | 2 Deamidation (NQ),Oxidation (W) | 2 | 1807.83 | -4.46 | 720160 | 51 |
| G3TIU4 | MEF2 activating motif and SAP domain containing transcriptional regulator  (*Loxodonta africana*) | PKGNLTYHQYMPPEPR | Deamidation (NQ),Oxidation Y,O-2H (Y) | 2 | 1957.90 | 1.81 | 1226400 | 43 |
| G3TJ26 | Uncharacterized protein  (*Loxodonta africana*) | GPDSVGAGDGKNPK | Oxidation (P) | 2 | 1313.62 | 4.05 | 286620 | 73 |
| G3TJ56 | Phosphoinositide 5-phosphatase, EC 3.1.3.36  (*Loxodonta africana*) | GVLGDQWSQGSWPRK | Deamidation (NQ),Oxidation (W) | 2 | 1716.82 | -4.02 | 1045000 | 44 |
| G3TJ67 | Kallikrein B1  (*Loxodonta africana*) | SVEECQKR | Acetyl (K),Trioxidation (C) | 2 | 1124.48 | -0.62 | 476440 | 52 |
| G3TJB6 | Gamma-tubulin complex component  (*Loxodonta africana*) | RIQEFQESIPK | 2 Deamidation (NQ) | 2 | 1375.70 | -0.23 | 13708000 | 99 |
| G3TJE7 | Transmembrane and coiled-coil domain family 2  (*Loxodonta africana*) | EIEQNGPSRQPKDVLR | Deamidation (NQ) | 3 | 1865.96 | -1.14 | 4372500 | 48 |
| G3TJG2 | Desmoglein 4  (*Loxodonta africana*) | TGEIQFSR | Unmodified | 2 | 936.47 | 1.01 | 1219900 | 153 |
| G3TJK2 | Stearoyl-CoA desaturase  (*Loxodonta africana*) | AEKMLMFQR | di-oxidation (M) | 2 | 1184.57 | 0.28 | 17507000 | 79 |
| G3TJW1 | PITPNM family member 3  (*Loxodonta africana*) | GPPKFESVP | 2 Oxidation (P) | 2 | 988.49 | -4.42 | 873700 | 107 |
| G3TJW8 | Pericentrin  (*Loxodonta africana*) | AVPQGQDPQRQEKPLK | 3 Deamidation (NQ) | 2 | 1820.93 | -4.16 | 405660 | 40 |
| G3TJX3 | Decaprenyl diphosphate synthase subunit 1  (*Loxodonta africana*) | HTVNKIWGEKK | Deamidation (NQ),Oxidation (W) | 2 | 1355.72 | 0.78 | 1499300 | 48 |
| G3TJX4 | Flotillin  (*Loxodonta africana*) | KAAYDIEVNTRR | O-2H (Y) | 3 | 1448.74 | -0.25 | 12757000 | 70 |
| G3TK10 | S-adenosylmethionine synthase, EC 2.5.1.6  (*Loxodonta africana*) | FVIGGPQGDAGLTGR | Unmodified | 2 | 1443.75 | 0.64 | 605680 | 72 |
| G3TK51 | FYVE, RhoGEF and PH domain containing 1  (*Loxodonta africana*) | ESSNLNLPR | Glu->pyro-Glu,Deamidation (NQ) | 2 | 1011.50 | -2.20 | 554880 | 49 |
| G3TK53 | DAZ associated protein 1  (*Loxodonta africana*) | PKEGWQKGPR | O-2H (W) | 2 | 1195.61 | 0.78 | 813920 | 74 |
| G3TK61 | Testis expressed 15, meiosis and synapsis associated  (*Loxodonta africana*) | MESEEKYMQDTLNLGTVSAR | O-2H (Y) | 3 | 2315.04 | 2.69 | 3781600 | 50 |
| G3TK70 | Membrane associated guanylate kinase, WW and PDZ domain containing 1  (*Loxodonta africana*) | LNKDLRHFLNQR | 2 Deamidation (NQ) | 2 | 1554.83 | -0.53 | 572390 | 46 |
| G3TK82 | Chromodomain helicase DNA binding protein 1 like  (*Loxodonta africana*) | KEKEEAEHK | Unmodified | 2 | 1126.56 | 0.88 | 2110300 | 96 |
| G3TKF5 | CD163 molecule like 1  (*Loxodonta africana*) | MSSPAEEAWITCEDK | Oxidation (M),Trp->Kynurenine | 2 | 1772.72 | -1.97 | 4000100 | 50 |
| G3TKI5 | Scavenger receptor class B member 1  (*Loxodonta africana*) | PQVQERGPYVYR | Deamidation (NQ),dioxidation Y,O-2H (Y) | 2 | 1537.72 | -3.08 | 823840 | 51 |
| G3TKM5 | Actin related protein T3  (*Loxodonta africana*) | GLITSWGDMEIMWK | O-2H (W),Trp->Kynurenine | 2 | 1683.76 | -1.72 | 188850 | 50 |
| G3TKN2 | Fanconi-associated nuclease, EC 3.1.4.1  (*Loxodonta africana*) | LGAEVEVCHVVAVGAKSKGLS | Acetyl (K),Trioxidation (C) | 3 | 2199.12 | 4.23 | 1914100 | 44 |
| G3TKX0 | Presequence translocase associated motor 16  (*Loxodonta africana*) | VNDKSVGGSFYLQSK | 2 Deamidation (NQ),Iodination | 2 | 1755.69 | 3.81 | 16606000 | 55 |
| G3TL19 | Nuclear receptor subfamily 5 group A member 1  (*Loxodonta africana*) | NNLLIEMLQAKQT | Oxidation (M),Deamidation (NQ) | 2 | 1531.79 | 4.22 | 30950000 | 84 |
| G3TL89 | Zinc finger protein 879  (*Loxodonta africana*) | QLGISTWRK | Deamidation (NQ),Oxidation (W) | 2 | 1104.59 | 0.35 | 3867200 | 59 |
| G3TLC6 | Non-specific serine/threonine protein kinase, EC 2.7.11.1  (*Loxodonta africana*) | LQMLQDCPKRR | Oxidation (M),Deamidation (NQ) | 2 | 1460.72 | -0.82 | 1473100 | 61 |
| G3TM20 | P-type Cu(+) transporter, EC 7.2.2.8  (*Loxodonta africana*) | GMTCASCVHK | Trioxidation (C),di-oxidation (M) | 2 | 1229.45 | -0.75 | 4914800 | 42 |
| G3TM31 | NFAT activating protein with ITAM motif 1  (*Loxodonta africana*) | SFIVLYYYVDRQNK | Deamidation (NQ),2 Di-iodination,Oxidation Y | 3 | 2327.50 | -2.99 | 4600300 | 43 |
| G3TM79 | Dynein axonemal heavy chain 2  (*Loxodonta africana*) | KIQAQISSGMTANASQLQNYLK | Acetyl (K),2 Deamidation (NQ) | 3 | 2437.22 | -4.17 | 566880 | 53 |
| G3TME7 | Protein KRI1 homolog  (*Loxodonta africana*) | VASMSAPEKPAPQKR | Oxidation (M),Oxidation (P) | 3 | 1627.84 | 0.75 | 530920 | 44 |
| G3TMU7 | BCL2 like 2  (*Loxodonta africana*) | LREGNWASVR | Di-oxidation W | 2 | 1218.61 | 1.33 | 2821400 | 54 |
| G3TMW0 | SIX homeobox 4  (*Loxodonta africana*) | NALKELYKQNR | Acetyl (K),3 Deamidation (NQ) | 2 | 1420.72 | 0.44 | 697910 | 45 |
| G3TMX0 | HORMA domain containing 2  (*Loxodonta africana*) | VSEPVKVFVPNR | 2 Oxidation (P) | 2 | 1401.76 | 1.25 | 353310 | 76 |
| G3TN13 | Ras-related protein Rab  (*Loxodonta africana*) | ENKNINEAMR | Acetyl (K),2 Deamidation (NQ) | 2 | 1261.56 | -0.95 | 919650 | 56 |
| G3TN38 | [Heparan sulfate]-glucosamine N-sulfotransferase, EC 2.8.2.8  (*Loxodonta africana*) | QDNELSEMATEVNCGDLQHLPYKLMEVK | Deamidation (NQ) | 3 | 3291.51 | -2.26 | 4109000 | 42 |
| G3TN55 | ITI_HC_C domain-containing protein  (*Loxodonta africana*) | PGAPSQPK | Oxidation (P) | 1 | 796.41 | 1.03 | 990080 | 57 |
| G3TN87 | Uncharacterized protein | VRIGCGSFVGNFFEYYR | Deamidation (NQ),O-2H (Y) | 3 | 2084.94 | 4.42 | 498240 | 52 |
| G3TN91 | Non-specific serine/threonine protein kinase, EC 2.7.11.1  (*Loxodonta africana*) | TSFTQGMSK | Unmodified | 1 | 985.45 | -2.51 | 1741400 | 46 |
| G3TNL4 | Collagen type IV alpha 1 chain  (*Loxodonta africana*) | GDPGTPGVPGK | 2 Oxidation (P) | 1 | 1012.48 | -0.15 | 2685600 | 71 |
| G3TNP1 | Syndecan  (*Loxodonta africana*) | KDEGSYSLEEPKQANGGAYQK | O-2H (Y) | 4 | 2312.06 | 2.53 | 2626000 | 68 |
| G3TNZ5 | Solute carrier family 16 member 8  (*Loxodonta africana*) | PGPGPQLPAAPPGGR | Deamidation (NQ),3 Oxidation (P) | 2 | 1416.70 | -1.38 | 1213000 | 71 |
| G3TP39 | Uncharacterized protein  (*Loxodonta africana*) | LRQAGWEQMWK | Deamidation (NQ),Oxidation (W) | 2 | 1448.69 | 0.60 | 1219000 | 53 |
| G3TP54 | Phosphodiesterase, EC 3.1.4.-  (*Loxodonta africana*) | FHEEITPMLDGITNNR | Oxidation (M),2 Deamidation (NQ) | 2 | 1903.86 | 4.25 | 311160 | 46 |
| G3TPA6 | Wnt ligand secretion mediator  (*Loxodonta africana*) | VWFAMKTFLTPSIFIIMVWYWR | O-2H (W),Di-oxidation W | 3 | 2880.45 | -3.50 | 2372700 | 58 |
| G3TPK9 | NADH dehydrogenase [ubiquinone] flavoprotein 1, mitochondrial, EC 7.1.1.2  (*Loxodonta africana*) | MQRFAQQHQARQVAF | Oxidation (M) | 3 | 1860.92 | 0.81 | 189160 | 51 |
| G3TPX1 | G3BP stress granule assembly factor 2  (*Loxodonta africana*) | GPGGPRGIVGGGMMR | Oxidation (M),2 Oxidation (P) | 2 | 1445.69 | 1.32 | 4129700 | 54 |
| G3TQ07 | Coiled-coil domain containing 33  (*Loxodonta africana*) | SLSWCLGRAQEAGKQMPEEAQEMSNYR | 2 Deamidation (NQ),Di-oxidation W | 3 | 3189.38 | 0.07 | 933450 | 42 |
| G3TQ10 | Ankyrin 3  (*Loxodonta africana*) | MGISASTMTLKK | Oxidation (M) | 2 | 1282.66 | 1.83 | 2436400 | 40 |
| G3TQ25 | Anaphase-promoting complex subunit 1  (*Loxodonta africana*) | WVDSNVPQIIR | Deamidation (NQ),O-2H (W) | 2 | 1340.67 | -0.28 | 3128300 | 78 |
| G3TQ47 | EMAP like 4  (*Loxodonta africana*) | QVCMWNSVEHR | Oxidation (M),Di-oxidation W | 2 | 1492.62 | 3.92 | 776300 | 51 |
| G3TQ84 | E2F transcription factor 8  (*Loxodonta africana*) | YAEQIMMIK | Oxidation (M),Deamidation (NQ),di-oxidation (M) | 2 | 1174.53 | -1.85 | 963730 | 43 |
| G3TQ94 | Uncharacterized protein  (*Loxodonta africana*) | GLAEYLKEYK | O-2H (Y) | 2 | 1226.62 | -3.44 | 1319700 | 66 |
| G3TQH3 | Chromosome 2 open reading frame 68  (*Loxodonta africana*) | PDLQVYLPR | Deamidation (NQ),Oxidation (P) | 2 | 1116.58 | -3.62 | 7530200 | 93 |
| G3TQI1 | Lymphocyte expansion molecule  (*Loxodonta africana*) | IGIKAYQMLLGSWNPVGVGR | Iodination | 3 | 2284.07 | 1.01 | 1912900 | 46 |
| G3TQK2 | F-box and leucine-rich repeat protein 5 (F-box/LRR-repeat protein 5)  (*Loxodonta africana*) | GLSLWNQAEER | 2 Deamidation (NQ),Trp->Kynurenine | 2 | 1307.60 | -1.16 | 1269400 | 60 |
| G3TR20 | Thimet oligopeptidase 1  (*Loxodonta africana*) | QEGVLNGKVGMDYR | Oxidation (M) | 2 | 1580.76 | -0.15 | 586700 | 59 |
| G3TR31 | Transducin beta like 2  (*Loxodonta africana*) | AVVEEMQGLLK | Deamidation (NQ),di-oxidation (M) | 2 | 1248.63 | 3.69 | 537590 | 55 |
| G3TR37 | VPS8 subunit of CORVET complex  (*Loxodonta africana*) | GLNPKQDYCSVCLQQYKR | Unmodified | 3 | 2256.08 | -1.42 | 957920 | 53 |
| G3TRD7 | Uncharacterized protein  (*Loxodonta africana*) | EQLEEWQHSEAVLSGQLK | Oxidation (W) | 2 | 2126.03 | -3.45 | 1116900 | 66 |
| G3TRN8 | Olfactory receptor  (*Loxodonta africana*) | EVMGAMSKLWEK | Glu->pyro-Glu,Oxidation (W) | 2 | 1405.67 | 0.63 | 1597600 | 65 |
| G3TRV0 | ATP binding cassette subfamily A member 9  (*Loxodonta africana*) | PTAGQVGVGKWGGLPRSR | Deamidation (NQ),2 Oxidation (P) | 3 | 1854.97 | -1.35 | 2310800 | 68 |
| G3TRX5 | Retinoic acid induced 14  (*Loxodonta africana*) | VTEEEIDVLK | Unmodified | 2 | 1173.61 | -1.28 | 1093300 | 93 |
| G3TS11 | Glutamate receptor  (*Loxodonta africana*) | GEWNGMVKELIDHR | Deamidation (NQ),Oxidation (W) | 2 | 1699.80 | 2.22 | 1019400 | 63 |
| G3TS67 | Aldo-keto reductase family 7 like (gene/pseudogene)  (*Loxodonta africana*) | RMDAPASAEAVR | di-oxidation (M) | 3 | 1304.61 | -0.88 | 925050 | 62 |
| G3TSK2 | Neuregulin 2  (*Loxodonta africana*) | QNMCPAHQNR | 2 Deamidation (NQ),Trioxidation (C) | 2 | 1304.49 | -0.12 | 15646000 | 75 |
| G3TSL2 | Tyrosine 3-monooxygenase/tryptophan 5-monooxygenase activation protein beta  (*Loxodonta africana*) | DSTLIMQLLR | Oxidation (M) | 2 | 1204.65 | 0.86 | 508570 | 107 |
| G3TST0 | Uncharacterized protein  (*Loxodonta africana*) | YWFMNNMHNPK | Oxidation (M),Deamidation (NQ),Oxidation (W) | 2 | 1513.61 | -2.30 | 1224400 | 41 |
| G3TSU6 | Fucosyltransferase 4  (*Loxodonta africana*) | GLVAWVVSHWDERHAR | O-2H (W),Di-oxidation W | 2 | 1962.94 | -4.17 | 354610 | 40 |
| G3TT32 | ArfGAP with GTPase domain, ankyrin repeat and PH domain 3  (*Loxodonta africana*) | VIDDSRARK | Unmodified | 2 | 1058.58 | 1.39 | 2818700 | 105 |
| G3TT73 | RNA helicase, EC 3.6.4.13  (*Loxodonta africana*) | GNIFASVDTRKNYQGK | Deamidation (NQ),O-2H (Y) | 2 | 1811.88 | -0.61 | 5787300 | 45 |
| G3TTS5 | Palmitoyltransferase, EC 2.3.1.225  (*Loxodonta africana*) | SHLLLQMNLCGRNLR | 2 Deamidation (NQ),Trioxidation (C) | 2 | 1873.91 | 4.41 | 922220 | 50 |
| G3TU70 | Matrix metallopeptidase 17  (*Loxodonta africana*) | GLPLHLDSVDAVYERTSDHK | Oxidation Y | 3 | 2267.12 | 1.45 | 1063900 | 41 |
| G3TUH6 | SLAIN motif family member 2  (*Loxodonta africana*) | GMEYSRVSPQPMISRLQQPR | di-oxidation (M) | 3 | 2391.18 | 3.81 | 1865000 | 49 |
| G3TUK4 | Voltage-dependent T-type calcium channel subunit alpha  (*Loxodonta africana*) | VVSLGLYFGEEAYLR | Oxidation Y,dioxidation Y | 2 | 1762.88 | -3.57 | 1320100 | 49 |
| G3TUP2 | Epiplakin 1  (*Loxodonta africana*) | GLMHQDTYLLVSDQKLMK | Deamidation (NQ),Oxidation Y | 3 | 2136.06 | 3.64 | 591990 | 48 |
| G3TUT3 | CD109 molecule  (*Loxodonta africana*) | KQLTDNLR | Acetyl (K),Deamidation (NQ) | 2 | 1029.55 | 0.69 | 8799100 | 139 |
| G3TV10 | Myosin light chain kinase 3  (*Loxodonta africana*) | GPGAGNPEPGK | Deamidation (NQ) | 1 | 980.46 | 0.00 | 422150 | 46 |
| G3TVE4 | Plakophilin 2  (*Loxodonta africana*) | VKEQYQDMPMPEEK | Acetyl (K),Deamidation (NQ),di-oxidation (M) | 2 | 1825.78 | 3.37 | 5923700 | 66 |
| G3TVH4 | Uncharacterized protein  (*Loxodonta africana*) | VANASYYRK | Deamidation (NQ),Oxidation Y | 2 | 1087.53 | 0.07 | 38458000 | 67 |
| G3TVH5 | 6-phosphofructo-2-kinase, EC 2.7.1.105, EC 3.1.3.46 (Fructose-2,6-bisphosphatase)  (*Loxodonta africana*) | RTIQTAEALGVPYEQWK | O-2H (Y) | 2 | 2003.01 | 4.32 | 536310 | 50 |
| G3TVH8 | Ring finger protein 111  (*Loxodonta africana*)  (*Loxodonta africana*) | RLPCMHLFHQVCVDQWLITNKK | O-2H (W) | 4 | 2836.41 | 2.44 | 1186600 | 41 |
| G3TVK9 | Uncharacterized protein  (*Loxodonta africana*) | NNITAQGFQDIAVAMEK | Oxidation (M),2 Deamidation (NQ) | 2 | 1866.87 | 1.91 | 1136200 | 54 |
| G3TVP3 | Helix-destabilizing protein (Heterogeneous nuclear ribonucleoprotein A1) (Heterogeneous nuclear ribonucleoprotein A1, N-terminally processed) (hnRNP core protein A1)  (*Loxodonta africana*) | SPKEPEQLRK | Oxidation (P) | 2 | 1226.66 | 0.00 | 1805300 | 104 |
| G3TVR4 | Glycerophosphodiester phosphodiesterase domain containing 1  (*Loxodonta africana*) | LKEPHTMSRCQK | Oxidation (M),Deamidation (NQ) | 2 | 1530.73 | 2.22 | 647140 | 66 |
| G3TVU2 | Uncharacterized protein  (*Loxodonta africana*) | PALLQWMR | Oxidation (M),O-2H (W) | 2 | 1043.52 | 2.43 | 562920 | 63 |
| G3TVZ7 | Uncharacterized protein  (*Loxodonta africana*) | TQIPENWEHAK | Deamidation (NQ),O-2H (W) | 2 | 1366.62 | 2.65 | 949500 | 42 |
| G3TWH5 | E1A binding protein p400  (*Loxodonta africana*) | APNDENQDYYHK | 3 Deamidation (NQ) | 2 | 1495.57 | 2.29 | 47124 | 50 |
| G3TWJ7 | Family with sequence similarity 91 member A1  (*Loxodonta africana*) | LGNSQREYEK | Deamidation (NQ),O-2H (Y) | 2 | 1237.56 | 1.55 | 2305600 | 66 |
| G3TWN7 | LDL receptor related protein 1  (*Loxodonta africana*) | QPDAVPNHPCKVNNGGCSNLCLLSPR | Deamidation (NQ),Oxidation (P) | 3 | 2920.34 | 3.55 | 858170 | 42 |
| G3TWQ6 | Small integral membrane protein 5  (*Loxodonta africana*) | VAPSFAQEMHAMGEKLLLK | Unmodified | 3 | 2099.09 | -1.17 | 737490 | 42 |
| G3TWX2 | DERPC proline and glycine rich nuclear protein  (*Loxodonta africana*) | LGGLPGTGPMSNQR | 2 Deamidation (NQ),di-oxidation (M) | 2 | 1417.65 | -3.35 | 710150 | 61 |
| G3TX58 | Protein Wnt  (*Loxodonta africana*) | AETVSSCNCK | 2 Trioxidation (C) | 2 | 1250.44 | -4.46 | 3738000 | 80 |
| G3TX86 | Kinesin family member 13B  (*Loxodonta africana*) | LSPEKQNYR | Deamidation (NQ),dioxidation Y | 2 | 1166.56 | 1.83 | 839270 | 85 |
| G3TXC8 | Azurocidin 1  (*Loxodonta africana*) | STWQGKGEGQGLPRGLR | Oxidation (P) | 3 | 1841.95 | 0.90 | 503700 | 58 |
| G3TXQ1 | CD302 molecule  (*Loxodonta africana*) | KQWKGPADILLGMFYDTDDACFK | Acetyl (K),Deamidation (NQ) | 3 | 2761.28 | -1.39 | 831060 | 43 |
| G3TXR8 | Solute carrier family 2 member 10  (*Loxodonta africana*) | QSSAGIQYRRIEVSSAS | Gln->pyro-Glu | 2 | 1820.90 | -0.45 | 2612200 | 47 |
| G3TXW7 | Zinc finger protein 609  (*Loxodonta africana*) | ESSNPLTPGK | Glu->pyro-Glu,Oxidation (P) | 2 | 1026.50 | -0.73 | 1324500 | 70 |
| G3TY18 | TTF-type domain-containing protein  (*Loxodonta africana*) | QILPHSNYQAVDHR | 2 Deamidation (NQ),Oxidation (P) | 2 | 1694.80 | -1.86 | 558590 | 54 |
| G3TY41 | Polyadenylate-binding protein, PABP  (*Loxodonta africana*) | MNGMLLNDRK | Oxidation (M) | 2 | 1206.58 | 2.81 | 985260 | 93 |
| G3TY50 | High mobility group box 2  (*Loxodonta africana*) | LGEMWSEQSAK | Di-oxidation W | 2 | 1296.57 | -2.28 | 129260 | 42 |
| G3TY97 | Arginase, EC 3.5.3.1  (*Loxodonta africana*) | VMEETLSYLLGR | Oxidation (M) | 2 | 1425.72 | -2.16 | 530800 | 96 |
| G3TYF5 | BZIP domain-containing protein  (*Loxodonta africana*) | TVDKHSDEYK | Acetyl (K) | 3 | 1262.58 | 4.49 | 303250 | 42 |
| G3TYV4 | RBR-type E3 ubiquitin transferase, EC 2.3.2.31  (*Loxodonta africana*) | RSRTGMHW | Oxidation (M),Trp->Kynurenine | 2 | 1049.48 | -2.47 | 1296200 | 55 |
| G3TYW3 | HYLS1 centriolar and ciliogenesis associated  (*Loxodonta africana*) | EAQSTQYDPYSKASVTPGK | Glu->pyro-Glu,dioxidation Y | 2 | 2069.95 | 2.86 | 361410 | 50 |
| G3TYY5 | DNA helicase, EC 3.6.4.12  (*Loxodonta africana*) | IEEIEREIIK | Unmodified | 2 | 1270.71 | 1.31 | 808680 | 96 |
| G3TZ29 | Intersectin 2  (*Loxodonta africana*) | QRELQEQEWK | Deamidation (NQ),Oxidation (W) | 2 | 1389.65 | -0.38 | 3331000 | 50 |
| G3TZ73 | Solute carrier family 25 member 43  (*Loxodonta africana*) | GPWATGLQVWR | Oxidation (W),Di-oxidation W | 2 | 1317.65 | -4.38 | 172240 | 61 |
| G3TZX9 | EF-hand calcium binding domain 13  (*Loxodonta africana*) | TVEKPLNKEQCK | Acetyl (K),Deamidation (NQ),Trioxidation (C) | 2 | 1563.75 | 4.04 | 1815000 | 67 |
| G3U013 | Intracisternal A particle-promoted polypeptide  (*Loxodonta africana*) | FYSFLQTSK | dioxidation Y | 2 | 1151.55 | -2.01 | 630030 | 73 |
| G3U077 | Coronin  (*Loxodonta africana*) | DAGPLLISLKDGYVPPK | Oxidation (P) | 2 | 1797.99 | 1.08 | 559230 | 50 |
| G3U0E8 | DNA polymerase, EC 2.7.7.7  (*Loxodonta africana*) | PCAPILGAKVQSYER | Deamidation (NQ),O-2H (Y) | 3 | 1702.83 | -2.97 | 257340 | 46 |
| G3U0I0 | Talin 1  (*Loxodonta africana*) | EVIQEWNLTNIKRWAASPK | Glu->pyro-Glu,Deamidation (NQ),Di-oxidation W | 3 | 2297.18 | -2.97 | 1427100 | 72 |
| G3U0L1 | FTCD_N domain-containing protein  (*Loxodonta africana*) | ESIGEIWK | O-2H (W) | 1 | 974.47 | 1.15 | 2551600 | 56 |
| G3U0R2 | Dmx like 2  (*Loxodonta africana*) | NKAMLEPENTPFK | Oxidation (M),Deamidation (NQ) | 2 | 1534.73 | -2.51 | 1214900 | 59 |
| G3U0T6 | Matrix remodeling associated 5  (*Loxodonta africana*) | AQITWELPDKSHLTAGTQAR | Deamidation (NQ),Oxidation (P) | 3 | 2239.12 | -4.18 | 1990200 | 54 |
| G3U0W1 | Tubulin alpha chain  (*Loxodonta africana*) | AVFVDLEPTVIDEVR | Unmodified | 2 | 1700.90 | 1.39 | 2116700 | 199 |
| G3U0Z1 | Oxysterol-binding protein  (*Loxodonta africana*) | AVAPCRGGGCSGR | 2 Trioxidation (C) | 2 | 1399.56 | 4.22 | 778200 | 52 |
| G3U157 | [Histone H3]-dimethyl-L-lysine(36) demethylase, EC 1.14.11.27  (*Loxodonta africana*) | PGQMDNRSKLR | Oxidation (M),Deamidation (NQ) | 2 | 1317.65 | -0.73 | 257570 | 55 |
| G3U191 | Uncharacterized protein  (*Loxodonta africana*) | EQMEFEESMR | Oxidation (M),Deamidation (NQ),di-oxidation (M) | 2 | 1363.49 | -1.15 | 1793100 | 62 |
| G3U1B9 | Myb-like domain-containing protein  (*Loxodonta africana*) | SSQPGVCASSQEKPPR | Acetyl (K),Deamidation (NQ),Trioxidation (C) | 2 | 1804.79 | 3.60 | 1177500 | 55 |
| G3U1C9 | Alpha-1A adrenergic receptor (Alpha-1A adrenoreceptor) (Alpha-1C adrenergic receptor)  (*Loxodonta africana*) | YIGVSYPLR | Oxidation (P) | 2 | 1082.58 | 0.25 | 1593300 | 114 |
| G3U1M9 | Ventral anterior homeobox 1  (*Loxodonta africana*) | GPAGPCRR | Oxidation (P) | 1 | 885.42 | -4.18 | 784280 | 49 |
| G3U1Y8 | Glycylpeptide N-tetradecanoyltransferase, EC 2.3.1.97  (*Loxodonta africana*) | MKGFDVFNALDLMENK | Acetyl (K),Oxidation (M) | 3 | 1928.90 | 3.76 | 679670 | 56 |
| G3U2F4 | ArfGAP with GTPase domain, ankyrin repeat and PH domain 2  (*Loxodonta africana*) | RSGNSLNKEWK | Deamidation (NQ),Trp->Kynurenine | 2 | 1322.66 | 0.24 | 419970 | 52 |
| G3U2G3 | RNA pseudouridine synthase D4  (*Loxodonta africana*) | KEELNLVCK | Acetyl (K),Trioxidation (C) | 2 | 1221.59 | 4.36 | 2571700 | 96 |
| G3U2Z5 | Transporter  (*Loxodonta africana*) | PGQLPSEQYWNK | 2 Deamidation (NQ),Trp->Kynurenine | 2 | 1451.66 | -2.30 | 467100 | 59 |
| G3U301 | DNA polymerase epsilon catalytic subunit, EC 2.7.7.7  (*Loxodonta africana*) | TILDWDYYIER | Trp->Kynurenine | 2 | 1489.71 | -1.29 | 2968300 | 70 |
| G3U3B6 | Uncharacterized protein  (*Loxodonta africana*) | TLQQMEMQK | Oxidation (M),Deamidation (NQ) | 2 | 1152.52 | -2.12 | 470800 | 79 |
| G3U3L3 | Protein kinase domain-containing protein  (*Loxodonta africana*) | ENPPNKMTQEK | Deamidation (NQ) | 2 | 1315.61 | -3.62 | 1537100 | 48 |
| G3U3U4 | F-box and leucine rich repeat protein 6  (*Loxodonta africana*) | RAYRSPEEVQWCLEQLLTSPLFPS | Deamidation (NQ),Trp->Kynurenine | 3 | 2910.42 | 3.79 | 3222700 | 42 |
| G3U4G0 | Gastrokine 3, pseudogene  (*Loxodonta africana*) | GLTYTVLPSR | Unmodified | 2 | 1105.61 | -1.27 | 4766900 | 92 |
| G3U4K1 | tRNA wybutosine-synthesizing protein 4, EC 2.1.1.290, EC 2.3.1.231 (tRNA(Phe) (7-(3-amino-3-(methoxycarbonyl)propyl)wyosine(37)-N)-methoxycarbonyltransferase) (tRNA(Phe) (7-(3-amino-3-carboxypropyl)wyosine(37)-O)-methyltransferase)  (*Loxodonta africana*) | WRHSATEVSYQNQK | 3 Deamidation (NQ) | 2 | 1735.78 | 2.51 | 212520 | 60 |
| G3U4Y3 | GDNF inducible zinc finger protein 1  (*Loxodonta africana*) | RHVLQVHEGGGERHQCQQCGK | Deamidation (NQ),Trioxidation (C) | 2 | 2548.14 | -0.51 | 746200 | 49 |
| G3U506 | Uncharacterized protein  (*Loxodonta africana*) | IINEPTAAAIAYGLDR | Unmodified | 2 | 1686.89 | -0.09 | 831240 | 100 |
| G3U591 | MAGE family member D1  (*Loxodonta africana*) | GPNVAYDFSQAATTNK | Unmodified | 3 | 1682.79 | -3.53 | 413320 | 63 |
| G3U599 | Ring finger protein 139  (*Loxodonta africana*) | HMYRIYGLQLLMEDTWK | Oxidation (M),Deamidation (NQ) | 3 | 2213.06 | 1.54 | 1600300 | 54 |
| G3U5B6 | DM1 locus, WD repeat containing  (*Loxodonta africana*) | SIDLNKPIDKR | Deamidation (NQ),Oxidation (P) | 2 | 1314.71 | 1.67 | 575210 | 91 |
| G3U5C4 | Kinesin-like protein  (*Loxodonta africana*) | LENQQMMKR | Oxidation (M) | 2 | 1192.57 | 3.01 | 1071000 | 76 |
| G3U5R5 | Nuclear receptor binding SET domain protein 2  (*Loxodonta africana*) | GVVSPPGRK | 2 Oxidation (P) | 2 | 927.51 | -1.12 | 713470 | 90 |
| G3U5R8 | Uncharacterized protein  (*Loxodonta africana*) | DLGPNGHVLYSLR | Deamidation (NQ),dioxidation Y | 2 | 1472.73 | 1.29 | 776320 | 54 |
| G3U5V7 | Uncharacterized protein  (*Loxodonta africana*) | IQEENVNLKNPLEK | 2 Deamidation (NQ) | 3 | 1668.86 | -1.27 | 969020 | 49 |
| G3U5W6 | PR/SET domain 13  (*Loxodonta africana*) | RGAAPLLACYSLCR | O-2H (Y) | 2 | 1620.79 | -4.31 | 660810 | 48 |
| G3U5Y3 | Non-specific serine/threonine protein kinase, EC 2.7.11.1  (*Loxodonta africana*) | STANQSFRKVVK | Acetyl (K),2 Deamidation (NQ) | 2 | 1407.74 | -1.30 | 400560 | 52 |
| G3U633 | Uncharacterized protein  (*Loxodonta africana*) | ELQTFIDRLEVPKSSEDR | Glu->pyro-Glu | 3 | 2143.09 | -2.60 | 333240 | 43 |
| G3U634 | Family with sequence similarity 155 member A  (*Loxodonta africana*) | IWLAAPRENEK | Deamidation (NQ),O-2H (W) | 2 | 1340.67 | -2.29 | 7713800 | 64 |
| G3U641 | ADAM metallopeptidase domain 22  (*Loxodonta africana*) | QSARLWETSI | Deamidation (NQ),Oxidation (W) | 2 | 1206.59 | 1.54 | 2529200 | 52 |
| G3U660 | Rho-associated protein kinase 2, EC 2.7.11.1 (Rho-associated, coiled-coil-containing protein kinase 2) (Rho-associated, coiled-coil-containing protein kinase II) (p164 ROCK-2)  (*Loxodonta africana*) | RGNDTDVRR | Unmodified | 2 | 1087.55 | 1.63 | 2084300 | 93 |
| G3U6B1 | G_PROTEIN_RECEP_F1_2 domain-containing protein  (*Loxodonta africana*) | GLQSQAPPVINDQHPVQPDAL | 2 Deamidation (NQ) | 3 | 2225.10 | 0.11 | 2610900 | 52 |
| G3U6I6 | Phosphatidate phosphatase, EC 3.1.3.4  (*Loxodonta africana*) | QQDGTYQCSPFHVR | 2 Deamidation (NQ),Trioxidation (C) | 2 | 1771.71 | -1.14 | 4162400 | 49 |
| G3U6K7 | PH domain-containing protein  (*Loxodonta africana*) | VGLEQQYWLYQLSR | Deamidation (NQ),dioxidation Y,O-2H (Y) | 2 | 1828.86 | -1.02 | 9116300 | 65 |
| G3U6R3 | Plexin A3  (*Loxodonta africana*) | YYRDIAKMASISDQDMDAYLVEQSR | 2 Deamidation (NQ),Oxidation Y,2 dioxidation Y | 3 | 3049.32 | 0.21 | 3808700 | 52 |
| G3U6S0 | Uncharacterized protein  (*Loxodonta africana*) | QMKEKEEAGTNK | Acetyl (K),Gln->pyro-Glu | 2 | 1416.66 | -1.99 | 301140 | 45 |
| G3U6Z8 | Sterile alpha motif domain containing 4A  (*Loxodonta africana*) | NPAANPTIMKQGRQNLWFANPGGSNSMPSR | Oxidation (M),3 Deamidation (NQ),Di-oxidation W | 3 | 3291.50 | -0.75 | 1706700 | 50 |
| G3U716 | Sodium channel protein  (*Loxodonta africana*) | IRKCFQGVFFR | Deamidation (NQ),Trioxidation (C) | 2 | 1505.74 | 3.27 | 1710800 | 51 |
| G3U786 | Cell cycle progression 1  (*Loxodonta africana*) | KQRTVNIENSR | 3 Deamidation (NQ) | 2 | 1346.68 | -3.54 | 1840400 | 46 |
| G3U7D1 | Peptidase S1 domain-containing protein  (*Loxodonta africana*) | IIPHPDYNSTTIDNDIMLIK | Deamidation (NQ) | 3 | 2313.16 | 0.35 | 510280 | 41 |
| G3U7R3 | Uncharacterized protein  (*Loxodonta africana*) | LDQCFQLR | 2 Deamidation (NQ),Trioxidation (C) | 2 | 1128.48 | -2.44 | 302600 | 74 |
| G3U8B5 | Leucine rich repeat containing 74B  (*Loxodonta africana*) | VNQTLRILISR | 2 Deamidation (NQ) | 3 | 1313.77 | -2.27 | 741170 | 52 |
| G3U8C3 | Keratin 10  (*Loxodonta africana*) | AEIECQNAEYQQLLDIK | 2 Deamidation (NQ),Trioxidation (C) | 2 | 2113.94 | 3.53 | 6084100 | 109 |
| G3U8L2 | 2'-phospho-ADP-ribosyl cyclase, EC 2.4.99.20, EC 3.2.2.6 (2'-phospho-ADP-ribosyl cyclase/2'-phospho-cyclic-ADP-ribose transferase) (2'-phospho-cyclic-ADP-ribose transferase) (ADP-ribosyl cyclase 1) (ADP-ribosyl cyclase/cyclic ADP-ribose hydrolase 1) (Cyclic ADP-ribose hydrolase 1)  (*Loxodonta africana*) | FTCQKEYRLAR | Trioxidation (C) | 2 | 1518.72 | 3.39 | 7173200 | 98 |
| G3U8Q2 | ATP binding cassette subfamily B member 1  (*Loxodonta africana*) | KQFFHAVMR | Acetyl (K),Oxidation (M),Deamidation (NQ) | 2 | 1221.60 | 2.01 | 2744100 | 89 |
| G3U8T2 | Matrix metallopeptidase 24  (*Loxodonta africana*) | GIPQAPQGAFISKEGYYTYFYK | Deamidation (NQ) | 3 | 2528.23 | -3.69 | 414190 | 63 |
| G3U8U7 | E3 ubiquitin-protein ligase, EC 2.3.2.27  (*Loxodonta africana*) | RNYYDPSSAPGK | Deamidation (NQ) | 2 | 1354.62 | 2.05 | 1076100 | 63 |
| G3U907 | F-box protein 46  (*Loxodonta africana*) | RVWDGIAAKINGITSWK | Di-oxidation W | 2 | 1946.04 | -1.37 | 4270500 | 57 |
| G3U9E9 | Migration and invasion inhibitory protein  (*Loxodonta africana*) | EPAVPEWNWR | Deamidation (NQ),Di-oxidation W | 2 | 1315.58 | -2.66 | 3128700 | 53 |
| G3U9Q8 | Uncharacterized protein  (*Loxodonta africana*) | PGIKNVYNGK | 2 Deamidation (NQ),dioxidation Y | 2 | 1122.56 | -0.93 | 5826100 | 57 |
| G3U9U5 | Chromodomain-helicase-DNA-binding protein 8, CHD-8, EC 3.6.4.12 (ATP-dependent helicase CHD8)  (*Loxodonta africana*) | GSAPAGNPGATGPPLK | Deamidation (NQ),2 Oxidation (P) | 2 | 1423.69 | 1.62 | 1652600 | 63 |
| G3U9Z9 | Asparaginase, EC 3.5.1.1  (*Loxodonta africana*) | PSLQGSPLGR | 2 Oxidation (P) | 2 | 1042.54 | 0.48 | 2860300 | 127 |
| G3UA10 | Sulfhydryl oxidase, EC 1.8.3.2  (*Loxodonta africana*) | KRICQNQPDTR | Acetyl (K),2 Deamidation (NQ) | 2 | 1458.69 | -2.87 | 415860 | 55 |
| G3UA29 | ArfGAP with SH3 domain, ankyrin repeat and PH domain 2  (*Loxodonta africana*) | STPLINKGQPR | Acetyl (K),2 Deamidation (NQ) | 2 | 1253.66 | 1.51 | 1001500 | 75 |
| G3UAA1 | Dystonin  (*Loxodonta africana*) | GGDQYFDNVTPK | Deamidation (NQ),O-2H (Y) | 2 | 1354.57 | -0.36 | 522470 | 52 |
| G3UAB5 | Coronin  (*Loxodonta africana*) | VLQEANCKNHR | Unmodified | 2 | 1367.67 | 3.00 | 614450 | 68 |
| G3UAC2 | Phosphatidylinositol-3-phosphate phosphatase, EC 3.1.3.64  (*Loxodonta africana*) | LALTTSGCPLVIQCK | 2 Trioxidation (C) | 2 | 1755.84 | 1.65 | 2784600 | 61 |
| G3UAI3 | Uncharacterized protein  (*Loxodonta africana*) | DAWERNVIKIHSSQR | Trp->Kynurenine | 3 | 1841.95 | 0.40 | 503700 | 77 |
| G3UAJ1 | RRM domain-containing protein  (*Loxodonta africana*) | LDFLQNPKQRDK | 2 Deamidation (NQ) | 2 | 1502.77 | -0.41 | 2435300 | 78 |
| G3UB70 | Coiled-coil domain-containing protein 128 (KLRAQ motif-containing protein 1) (Protein phosphatase 1 regulatory subunit 21)  (*Loxodonta africana*) | YYYNALNVPLHNR | 2 dioxidation Y | 2 | 1699.80 | 3.00 | 749310 | 91 |
| G3UB74 | Forkhead box A2  (*Loxodonta africana*) | PGKGSFWTLHPSGNMR | Deamidation (NQ),Oxidation (W) | 2 | 1787.84 | 3.70 | 1926700 | 51 |
| G3UB83 | Collagen type XIV alpha 1 chain  (*Loxodonta africana*) | PGSPGPPGSPGPR | 5 Oxidation (P) | 2 | 1238.55 | -0.07 | 520130 | 76 |
| G3UBC8 | Disintegrin and metalloproteinase domain-containing protein 10, EC 3.4.24.81 (Kuzbanian protein homolog) (Mammalian disintegrin-metalloprotease)  (*Loxodonta africana*) | NISQVLEKK | Acetyl (K),2 Deamidation (NQ) | 2 | 1101.59 | 1.03 | 1348500 | 96 |
| G3UBI1 | E1A binding protein p400  (*Loxodonta africana*) | KQCLDYHYKEMEVLK | 2 Acetyl (K),Deamidation (NQ) | 2 | 2067.96 | 3.08 | 1830300 | 70 |
| G3UBQ3 | Solute carrier family 5 member 3  (*Loxodonta africana*) | KKEMEDGSR | Oxidation (M) | 2 | 1094.50 | -3.37 | 737420 | 80 |
| G3UC07 | MUS81 structure-specific endonuclease subunit  (*Loxodonta africana*) | LQQHRASGGDHAPVSPPGEK | 2 Deamidation (NQ),2 Oxidation (P) | 2 | 2100.98 | -0.19 | 460080 | 46 |
| G3UC39 | Tr-type G domain-containing protein  (*Loxodonta africana*) | IGSIGTVPVGR | Unmodified | 2 | 1054.61 | -0.83 | 3097400 | 163 |
| G3UC82 | Janus kinase and microtubule interacting protein 2  (*Loxodonta africana*) | ENLEMIQREK | Deamidation (NQ),di-oxidation (M) | 2 | 1321.62 | -2.51 | 1037800 | 70 |
| G3UC84 | Protein phosphatase 2 scaffold subunit Abeta  (*Loxodonta africana*) | GPGEGTGKLFPFMEK | 2 Oxidation (P) | 2 | 1625.78 | -4.46 | 542960 | 63 |
| G3UC99 | Solute carrier family 25 member 39  (*Loxodonta africana*) | ELGTCVQAAVAQGGWR | 2 Deamidation (NQ),O-2H (W) | 2 | 1717.77 | 2.98 | 672450 | 45 |
| G3UCF9 | RNA polymerase II subunit C  (*Loxodonta africana*) | PESWGRYSPGPLTLGQPR | Oxidation Y | 3 | 2013.01 | -2.10 | 4245600 | 52 |
| G3UCL9 | 40S ribosomal protein S8  (*Loxodonta africana*) | YELGRPAANTK | Oxidation (P) | 2 | 1234.63 | -4.01 | 4689600 | 85 |
| G3UCM8 | Otoancorin  (*Loxodonta africana*) | EVSLFDLWR | Glu->pyro-Glu,O-2H (W) | 2 | 1159.57 | -2.12 | 6948000 | 46 |
| G3UCT3 | Exonuclease 5  (*Loxodonta africana*) | SISLQKWKR | Deamidation (NQ),O-2H (W) | 2 | 1159.63 | -2.76 | 2484000 | 103 |
| G3UD90 | Cholinergic receptor nicotinic beta 3 subunit  (*Loxodonta africana*) | RLFLQQLPKLLCMK | Deamidation (NQ),Trioxidation (C) | 2 | 1836.00 | 0.89 | 1900600 | 56 |
| G3UDC1 | RAD51 associated protein 2  (*Loxodonta africana*) | ERAGPNMHK | Oxidation (M) | 1 | 1054.50 | -3.67 | 812230 | 46 |
| G3UDE1 | NCCRP1, F-box associated domain containing  (*Loxodonta africana*) | VTDSSVSVQLRE | Unmodified | 2 | 1318.67 | 2.00 | 1027600 | 94 |
| G3UDL6 | Uncharacterized protein  (*Loxodonta africana*) | EGWPNTAYGVSK | Glu->pyro-Glu | 2 | 1289.60 | -2.88 | 2003800 | 45 |
| G3UDM4 | 40S ribosomal protein SA (37 kDa laminin receptor precursor, 37LRP) (37/67 kDa laminin receptor, LRP/LR) (67 kDa laminin receptor, 67LR) (Laminin receptor 1, LamR) (Laminin-binding protein precursor p40, LBP/p40)  (*Loxodonta africana*) | AIVAIENPADVSVISSR | Unmodified | 2 | 1739.94 | 0.38 | 587230 | 90 |
| G3UDT7 | APC down-regulated 1 like  (*Loxodonta africana*) | LGLSQASQDCAR | Unmodified | 3 | 1304.61 | 0.54 | 1635100 | 46 |
| G3UEW3 | Notum, palmitoleoyl-protein carboxylesterase  (*Loxodonta africana*) | GQQPPPPPPPPPPLR | 3 Oxidation (P) | 2 | 1618.85 | -2.46 | 2129700 | 79 |
| G3UFA9 | Uncharacterized protein  (*Loxodonta africana*) | SLCQAGPEAAPGLNAAR | Deamidation (NQ) | 3 | 1682.80 | 4.16 | 2448600 | 76 |
| G3UFJ9 | Eukaryotic translation initiation factor 2D  (*Loxodonta africana*) | LQPAYQVTFPGQEPIVKKGK | 3 Deamidation (NQ) | 3 | 2230.19 | -1.03 | 685230 | 46 |
| G3UFZ4 | G-patch domain containing 4  (*Loxodonta africana*) | GMKFAEEQLLK | Deamidation (NQ) | 2 | 1293.66 | -3.03 | 2707500 | 92 |
| G3UGD2 | Alcohol dehydrogenase [NADP(+)], EC 1.1.1.19, EC 1.1.1.2, EC 1.1.1.20, EC 1.1.1.372, EC 1.1.1.54 (Aldehyde reductase) (Aldo-keto reductase family 1 member A1) (Glucuronate reductase) (Glucuronolactone reductase)  (*Loxodonta africana*) | KVVSIPKSITPSR | Oxidation (P) | 3 | 1426.85 | 0.36 | 1687200 | 45 |
| G3UGJ4 | Uncharacterized protein  (*Loxodonta africana*) | TAPAAAVSPMQR | Oxidation (M),Deamidation (NQ),Oxidation (P) | 2 | 1231.59 | -0.97 | 3820500 | 63 |
| G3UGN6 | Regulator of chromosome condensation 2  (*Loxodonta africana*) | IKKLPEYNPR | O-2H (Y) | 2 | 1270.70 | -4.06 | 1354700 | 122 |
| G3UH39 | Keratin 75  (*Loxodonta africana*) | LAGLEDALQKAK | Acetyl (K) | 2 | 1297.72 | 0.33 | 1446500 | 121 |
| G3UH45 | Suppressor of cytokine signaling 5  (*Loxodonta africana*) | DKVGKMWNNFK | Acetyl (K),Deamidation (NQ) | 2 | 1408.68 | -1.77 | 719450 | 70 |
| G3UHD5 | IQ motif and ankyrin repeat containing 1  (*Loxodonta africana*) | VQQLTQEQQQR | 3 Deamidation (NQ) | 2 | 1387.66 | -4.37 | 329100 | 61 |
| G3UHK7 | BMP and activin membrane-bound inhibitor homolog  (*Loxodonta africana*) | LQDQRQQMLSR | 3 Deamidation (NQ) | 2 | 1404.67 | -1.15 | 664330 | 53 |
| G3UHL8 | Protein tyrosine phosphatase receptor type B  (*Loxodonta africana*) | VSLSNLISVR | Deamidation (NQ) | 2 | 1087.62 | 2.33 | 1413900 | 113 |
| G3UHQ8 | Tetratricopeptide repeat domain 9  (*Loxodonta africana*) | SQGAQCYKDKK | 2 Acetyl (K),Deamidation (NQ) | 2 | 1396.63 | -2.51 | 457710 | 67 |
| G3UHS7 | Tripartite motif containing 66  (*Loxodonta africana*) | WLCSSCTEEHRHGPAPGDPLFPRAQK | Deamidation (NQ),Oxidation (P) | 3 | 3049.39 | -2.70 | 931370 | 54 |
| G3UHZ7 | Uncharacterized protein  (*Loxodonta africana*) | MQEEEARKLQQK | 2 Deamidation (NQ) | 2 | 1518.73 | -2.06 | 471810 | 91 |
| G3UI02 | 40S ribosomal protein S28  (*Loxodonta africana*) | EGDVLTLLESER | Unmodified | 2 | 1359.69 | -0.83 | 486170 | 103 |
| G3UID3 | Hydroperoxy icosatetraenoate dehydratase, EC 1.14.14.1, EC 4.2.1.152  (*Loxodonta africana*) | CKHVQAQLR | Acetyl (K),Deamidation (NQ),Trioxidation (C) | 2 | 1229.58 | 3.09 | 271920 | 63 |
| G3UIE9 | BCL2 like 14  (*Loxodonta africana*) | SLSQKGLECLSANDPLTLGLR | 2 Deamidation (NQ),Oxidation (P) | 3 | 2289.15 | 0.61 | 2337900 | 47 |
| G3UIN5 | Phosphofurin acidic cluster sorting protein 2  (*Loxodonta africana*) | REGNKLQIMLQR | Acetyl (K),3 Deamidation (NQ),di-oxidation (M) | 2 | 1561.78 | -1.44 | 10097000 | 72 |
| G3UIP6 | Beta-sarcoglycan  (*Loxodonta africana*) | QSSNGPIK | Gln->pyro-Glu | 1 | 812.40 | 0.54 | 605860 | 44 |
| G3UIQ0 | Synaptotagmin like 2  (*Loxodonta africana*) | DDQQLKNMSGQWFYEAK | Oxidation (M),2 Deamidation (NQ) | 2 | 2104.90 | -4.37 | 1106300 | 68 |
| G3UIQ4 | TBC1 domain family member 10B  (*Loxodonta africana*) | ETRGELQYR | Glu->pyro-Glu,dioxidation Y | 2 | 1164.55 | -1.85 | 1835300 | 48 |
| G3UIX9 | Killer cell lectin like receptor F1  (*Loxodonta africana*) | KQIYKDFVCWFVMIK | Acetyl (K),Trioxidation (C),di-oxidation (M) | 2 | 2126.02 | -0.28 | 1116900 | 56 |
| G3UJ71 | Uncharacterized protein  (*Loxodonta africana*) | SDGIYMVNLKR | Deamidation (NQ),O-2H (Y) | 2 | 1309.63 | -1.67 | 2038300 | 82 |
| G3UJ85 | Synaptonemal complex protein 2  (*Loxodonta africana*) | EMQEEELLNVRK | Deamidation (NQ) | 2 | 1517.74 | 4.47 | 847990 | 124 |
| G3UJN9 | Spectrin repeat containing nuclear envelope protein 1  (*Loxodonta africana*) | LSLDQALVK | Deamidation (NQ) | 2 | 986.56 | 2.26 | 1596900 | 104 |
| G3UJW6 | HAUS augmin like complex subunit 1  (*Loxodonta africana*) | LAELKQQTKPLK | 2 Deamidation (NQ) | 2 | 1397.81 | -0.37 | 760140 | 48 |
| G3UKH1 | Apoptotic chromatin condensation inducer 1  (*Loxodonta africana*) | KTKAAPCIYWLPLTDSQTCSK | Trp->Kynurenine | 3 | 2471.22 | -2.63 | 2789500 | 49 |
| G3UKS7 | Corneodesmosin  (*Loxodonta africana*) | GSPGVPSFAAGPPISEGK | Unmodified | 2 | 1653.84 | -0.79 | 1102400 | 105 |
| G3ULD9 | Dermatan sulfate epimerase like  (*Loxodonta africana*) | NGAGNWLAQQIRK | 2 Deamidation (NQ),Di-oxidation W | 2 | 1488.73 | 1.42 | 845660 | 52 |
| G3ULI0 | BICD family like cargo adaptor 2  (*Loxodonta africana*) | LGPGPAGGFLSNLFRRT | Deamidation (NQ),Oxidation (P) | 2 | 1775.93 | -1.51 | 545100 | 59 |
| G3ULT5 | Zinc finger CW-type and PWWP domain containing 1  (*Loxodonta africana*) | GKDQYNKQPK | Deamidation (NQ),O-2H (Y) | 2 | 1219.58 | 0.42 | 561150 | 70 |
| G3ULU8 | SERTA domain containing 3  (*Loxodonta africana*) | WGWGPAGPR | Oxidation (W) | 2 | 998.47 | -4.00 | 1551100 | 96 |
| G3UM67 | SH3 and PX domains 2A  (*Loxodonta africana*) | TVSKLAQGSPAVAR | Acetyl (K),Deamidation (NQ) | 3 | 1426.78 | -2.25 | 724410 | 58 |
| G3UM73 | Non-specific serine/threonine protein kinase, EC 2.7.11.1  (*Loxodonta africana*) | QRLLEEQAKLQQQMDLQK | 2 Deamidation (NQ) | 2 | 2228.15 | 3.76 | 804150 | 55 |
| G3UML1 | ITPR interacting domain containing 2  (*Loxodonta africana*) | AQMKVCSQRVGR | Deamidation (NQ) | 2 | 1419.71 | -3.41 | 567060 | 64 |
| G3UMM9 | Potassium voltage-gated channel modifier  subfamily V member 2  (*Loxodonta africana*) | DDLCPRSFLEELGYWGVR | Unmodified | 3 | 2211.04 | 2.61 | 336260 | 67 |
| G3UMR1 | Uncharacterized protein  (*Loxodonta africana*) | TPGPGAQSALR | Unmodified | 2 | 1053.56 | -1.18 | 416120 | 106 |
| G3UMX4 | General transcription factor IIA subunit 1  (*Loxodonta africana*) | TLWENKLMQSR | Oxidation (M),O-2H (W) | 2 | 1434.69 | -0.18 | 743620 | 42 |
| G3UMX8 | Tankyrase 1 binding protein 1  (*Loxodonta africana*) | RDSLGAYTSRDASLR | Oxidation Y | 3 | 1682.83 | 3.77 | 778130 | 72 |
| G3UN05 | Chromosome 6 open reading frame 47  (*Loxodonta africana*) | GPEAPVEKPGR | 2 Oxidation (P) | 2 | 1167.59 | -0.83 | 1302400 | 74 |
| G3UN45 | Dmx like 1  (*Loxodonta africana*) | ESLAFPLWESNK | Glu->pyro-Glu | 2 | 1401.69 | -4.28 | 311650 | 55 |
| G3UN58 | LSDAT_euk domain-containing protein  (*Loxodonta africana*) | PGSLEQAMLDALVMDR | 2 di-oxidation (M) | 2 | 1808.83 | -0.93 | 2548600 | 52 |
| G3UN85 | Amine oxidase, EC 1.4.3.-  (*Loxodonta africana*) | DGLGQTVEMKR | Oxidation (M) | 2 | 1248.61 | -0.60 | 492900 | 44 |
| G3UNA5 | E3 ubiquitin-protein ligase TRIP12, EC 2.3.2.26 (HECT-type E3 ubiquitin transferase TRIP12) (Thyroid receptor-interacting protein 12)  (*Loxodonta africana*)  (*Loxodonta africana*) | PGPSGLQAK | Deamidation (NQ),Oxidation (P) | 1 | 870.44 | 0.91 | 409600 | 61 |
| G3UNA8 | Kelch like family member 35  (*Loxodonta africana*) | VLQTLNAYR | Deamidation (NQ),dioxidation Y | 2 | 1109.57 | 0.04 | 2630300 | 46 |
| G3UNB8 | DDHD domain containing 1  (*Loxodonta africana*) | PGNTGSQDHILPREICNR | Deamidation (NQ),2 Oxidation (P) | 2 | 2095.97 | 2.09 | 527250 | 53 |
| G3UNF4 | Uncharacterized protein  (*Loxodonta africana*) | REYEEEGPWAIYK | 2 Oxidation Y | 2 | 1700.77 | -3.49 | 1750100 | 46 |
| G5E6Y9 | MutS homolog 3  (*Loxodonta africana*) | ALENDGPVKKR | Deamidation (NQ) | 2 | 1226.66 | -0.93 | 2121700 | 121 |
| G5E6Z5 | Endophilin-3-interacting protein (SH3-containing GRB2-like protein 3-interacting protein 1)  (*Loxodonta africana*) | FNIKIKPLQSK | Acetyl (K),2 Deamidation (NQ) | 2 | 1358.78 | -0.77 | 6575200 | 48 |
| G5E707 | DNA topoisomerase 2, EC 5.6.2.2  (*Loxodonta africana*) | QTWMNNMMK | 2 di-oxidation (M) | 2 | 1246.48 | -2.87 | 3016200 | 64 |
| G5E797 | Twinfilin actin binding protein 2  (*Loxodonta africana*) | QKMINYIQLK | 2 Deamidation (NQ),O-2H (Y) | 2 | 1293.66 | -3.62 | 2707500 | 104 |
| G5E7C4 | Palmitoyltransferase, EC 2.3.1.225  (*Loxodonta africana*) | GKVGMSNPALTMENET | Oxidation (M),Deamidation (NQ) | 2 | 1694.75 | 1.62 | 970500 | 51 |
| Q0QES2 | IDP, EC 1.1.1.42 (NADP(+)-specific ICDH) (Oxalosuccinate decarboxylase)  (*Loxodonta africana*) | GLPNVQRSDYLNTFVFMD | O-2H (Y) | 3 | 2128.99 | -2.20 | 8340000 | 42 |
| Q0QF47 | Malate dehydrogenase, EC 1.1.1.37  (*Loxodonta africana*) | IFGVTTLDIVR | Unmodified | 2 | 1232.71 | -2.39 | 555320 | 72 |
| Q6B810 | Histone H4  *(Mammuthus primigenius)* | ISAMIYEETR | Oxidation (M) | 2 | 1227.58 | 0.50 | 812160 | 104 |
| W8BZ48 | Eukaryotic translation initiation factor 4C  (*Loxodonta africana*) | LEAMCFDGVKR | Acetyl (K),Oxidation (M) | 2 | 1382.63 | 1.55 | 178610 | 67 |

Table S2 . List of peptides and proteins of Proboscidea in trunk tip sample: for each peptide all the features (same sequence and different modifications) with the highest intensity are reported.

| **Acc. No.** | **Description *(Organism)*** | **Peptide Sequence** | **Modifications** | **Charge** | **Mass** | **Mass error [ppm]** | **MaxQuant Intensity** | **Score** |
| --- | --- | --- | --- | --- | --- | --- | --- | --- |
| G3SR49 | Glyceraldehyde-3-phosphate dehydrogenase  (*Loxodonta africana*) | QASEGPLK | Gln->pyro-Glu | 1 | 811.41 | 0.92 | 736460 | 55 |
|  |  | VIISAPSADAPMFVMGVNHEK | 2 Oxidation (M) | 3 | 2244.09 | -1.13 | 496370 | 51 |
| G3SR99 | Junction plakoglobin  (*Loxodonta africana*) | VSVELTNSLFK | Unmodified | 2 | 1235.68 | 0.38 | 2283700 | 94 |
|  |  |  | Deamidation (NQ) | 2 | 1236.66 | 0.44 | 333510 | 70 |
|  |  | LLNDEDPVVVTK | Unmodified | 2 | 1340.72 | 0.85 | 3144100 | 124 |
|  |  | AAMIVNQLSKK | Oxidation (M) | 3 | 1217.68 | 0.40 | 120230 | 50 |
|  |  | TMQNTSDLDTAR | Unmodified | 2 | 1351.60 | 1.03 | 374730 | 98 |
|  |  | ALMGSPQLVAAVVR | Unmodified | 2 | 1410.80 | 1.11 | 713620 | 91 |
|  |  |  | Oxidation (M) | 3 | 1426.80 | -0.55 | 236830 | 52 |
|  |  | NEGTATYAAAVLFR | Unmodified | 2 | 1482.75 | 0.96 | 1329700 | 158 |
|  |  | RALMGSPQLVAAVVR | Oxidation (M) | 3 | 1582.90 | 0.36 | 308470 | 53 |
|  |  | HVAAGTQQPYTDGVR | Unmodified | 3 | 1598.78 | 0.06 | 142940 | 73 |
|  |  | LIILANGGPQALVQIMR | Deamidation (NQ) | 2 | 1807.04 | 1.00 | 403390 | 102 |
|  |  |  | Oxidation (M),Deamidation (NQ) | 2 | 1823.03 | -0.37 | 957770 | 63 |
| G3STF4 | PEAK1 related, kinase-activating pseudokinase 1  (*Loxodonta africana*) | PAIPPKLSK | 3 Oxidation (P) | 2 | 997.58 | 0.32 | 2738300 | 77 |
|  |  | AGQAAPAQGQGQGRTGNTWAQK | 3 Deamidation (NQ) | 3 | 2185.01 | 0.94 | 488790 | 43 |
| G3SWF5 | Carboxypeptidase A4  (*Loxodonta africana*) | MDIFLLPVANPDGYVYTQTK | Unmodified | 3 | 2284.14 | -1.07 | 1960400 | 44 |
| G3T386 | Actin gamma 1  (*Loxodonta africana*) | SYELPDGQVITIGNER | Unmodified | 2 | 1789.88 | 0.01 | 595330 | 80 |
|  |  | VAPEEHPVLLTEAPLNPK | Unmodified | 3 | 1953.06 | 1.30 | 1390400 | 61 |
| G3T533 | Regulatory factor X1  (*Loxodonta africana*) | AKGETPIAVMGEVG | Oxidation (M) | 2 | 1373.69 | -3.41 | 175050 | 55 |
|  |  | LEPVNAASFGKLIR | Deamidation (NQ),Oxidation (P) | 3 | 1530.84 | -1.34 | 343830 | 44 |
| G3T7E7 | Annexin  (*Loxodonta africana*) | QDIAFAYQR | Gln->pyro-Glu | 2 | 1093.52 | 0.93 | 651790 | 91 |
|  |  | TNQELQEINR | Unmodified | 2 | 1243.62 | 0.66 | 1159000 | 153 |
|  |  | GVDEVTIVNILTNR | Unmodified | 2 | 1541.84 | -0.21 | 1734400 | 196 |
| G3TGT5 | Uncharacterized protein  (*Loxodonta africana*) | ERKVPEPTK | 2 Oxidation (P) | 2 | 1114.60 | -1.05 | 8015700 | 104 |
|  |  | KYEIVTDGR | O-2H (Y) | 2 | 1093.54 | 1.66 | 681960 | 63 |
| G3TJ97 | Retinoic acid induced 1  (*Loxodonta africana*) | MASRAAFQGAMKTK | Unmodified | 2 | 1496.76 | -1.41 | 4233300 | 92 |
| G3TJF5 | Desmoglein 1  (*Loxodonta africana*) | QEPSDSPMFIINR | Oxidation (M),Gln->pyro-Glu | 2 | 1531.70 | 1.03 | 1298800 | 80 |
|  |  |  | Gln->pyro-Glu | 2 | 1515.70 | 1.38 | 621690 | 79 |
|  |  | DGSNVIVTER | Unmodified | 2 | 1088.55 | 0.02 | 991750 | 134 |
|  |  | IIRQEPSDSPMFIINR | Oxidation (M) | 3 | 1930.99 | 0.04 | 525650 | 100 |
| G3TKQ3 | Elongation factor for RNA polymerase II 2  (*Loxodonta africana*) | AYKKPELLAR | O-2H (Y) | 2 | 1201.68 | -2.42 | 1316900 | 46 |
| G3TQI0 | 60S ribosomal protein L40  (*Loxodonta africana*) | TLSDYNIQK | Unmodified | 2 | 1080.55 | 0.42 | 856270 | 99 |
|  |  | IQDKEGIPPDQQR | Unmodified | 3 | 1522.77 | -1.69 | 419400 | 60 |
|  |  | TITLEVEPSDTIENVK | Unmodified | 2 | 1786.92 | 0.51 | 4953200 | 237 |
|  |  | TITLEVEPSDTIENVKAK | Unmodified | 3 | 1986.05 | 1.25 | 696650 | 106 |
| G3TRF5 | SH3 domain-containing protein  (*Loxodonta africana*) | QVQNLVNK | Gln->pyro-Glu | 2 | 924.50 | 0.25 | 1255400 | 128 |
|  |  | GIVDSITGQR | Unmodified | 2 | 1044.56 | 0.30 | 330380 | 101 |
|  |  | IEVLEEELR | Unmodified | 2 | 1128.60 | 0.27 | 2301600 | 138 |
|  |  | AELIVQPELK | Unmodified | 2 | 1138.66 | 0.75 | 2852100 | 116 |
|  |  | LLQLQEQMR | Unmodified | 2 | 1157.62 | 0.20 | 901720 | 93 |
|  |  | TLELQGLINDLQR | Unmodified | 2 | 1511.83 | 1.24 | 748470 | 134 |
|  |  | LLEAQIATGGIIDPK | Unmodified | 2 | 1537.87 | -0.12 | 5758600 | 53 |
|  |  | ALLQALLQTEDMLK | Oxidation (M) | 2 | 1601.87 | 0.18 | 2170100 | 124 |
| G3TUT3 | CD109 molecule  (*Loxodonta africana*) | KQLTDNLR | Acetyl (K),Deamidation (NQ) | 2 | 1029.55 | 0.94 | 7350900 | 118 |
|  |  | AQEALNMLTQR | Oxidation (M),2 Deamidation (NQ) | 2 | 1291.61 | -2.74 | 136790 | 45 |
| G3U0T6 | Matrix remodeling associated 5  (*Loxodonta africana*) | DLCWTSPTTAQPQR | Deamidation (NQ),O-2H (W) | 2 | 1674.73 | -2.33 | 939920 | 51 |
|  |  | EEGDYTCFAENQVGKDEMTVRVK | Oxidation (M),Deamidation (NQ) | 3 | 2721.19 | -3.62 | 802200 | 50 |
| G3U131 | Secreted frizzled related protein 4  (*Loxodonta africana*) | NVNARSAPKR | Deamidation (NQ) | 2 | 1112.61 | 1.95 | 134120 | 53 |
|  |  | TVQDKKQTAGR | 2 Deamidation (NQ) | 2 | 1232.64 | 4.09 | 260090 | 53 |
| G3U5E2 | Uncharacterized protein  (*Loxodonta africana*) | EPMWLNKIK | Oxidation (M),Glu->pyro-Glu | 2 | 1155.61 | 2.34 | 2727900 | 84 |
|  |  | TFNQSLLEAMFSK | Oxidation (M),2 Deamidation (NQ) | 2 | 1532.71 | 2.75 | 906670 | 50 |
| G3U5N8 | Microtubule actin crosslinking factor 1  (*Loxodonta africana*) | TLQNQLVDLK | Deamidation (NQ) | 2 | 1171.64 | 1.16 | 10844000 | 82 |
|  |  | SSLEATREMVTR | Oxidation (M) | 2 | 1394.68 | -2.72 | 854770 | 53 |
| G3U5V7 | Uncharacterized protein  (*Loxodonta africana*) | ESNRKGPK | Acetyl (K),Glu->pyro-Glu,Deamidation (NQ) | 2 | 939.48 | -3.21 | 375870 | 65 |
|  |  | IQEENVNLKNPLEK | 2 Deamidation (NQ) | 3 | 1668.86 | 2.29 | 1038000 | 65 |
| G3SRN5 | Guanylate cyclase  (*Loxodonta africana*) | ISINTFCR | Trioxidation (C) | 2 | 1057.49 | -3.34 | 3468200 | 63 |
|  |  |  | Deamidation (NQ),Trioxidation (C) | 2 | 1058.47 | -2.14 | 3052900 | 63 |
| G3T1Z8 | Peroxiredoxin 2  (*Loxodonta africana*) | QITVNDLPVGR | Gln->pyro-Glu | 2 | 1193.64 | -1.56 | 2317200 | 97 |
|  |  |  | Unmodified | 2 | 1210.67 | -0.87 | 1370700 | 118 |
| G3T8D8 | Serine palmitoyltransferase long chain base subunit 1  (*Loxodonta africana*) | EQEIEDQKNPR | Glu->pyro-Glu | 2 | 1366.65 | 0.61 | 4619700 | 115 |
|  |  |  | Glu->pyro-Glu,Deamidation (NQ) | 2 | 1367.63 | 0.00 | 521970 | 52 |
| G3SKZ6 | 26S proteasome non-ATPase regulatory subunit 6 (26S proteasome regulatory subunit RPN7)  (*Loxodonta africana*) | MKKANEEELK | Acetyl (K),Deamidation (NQ),di-oxidation (M) | 2 | 1293.61 | -1.66 | 171990 | 67 |
| G3SL14 | Glycoprotein-N-acetylgalactosamine 3-beta-galactosyltransferase 1, EC 2.4.1.122  (*Loxodonta africana*) | EGRDQLYWK | O-2H (Y) | 2 | 1207.56 | 2.86 | 1578700 | 71 |
| G3SL15 | Uncharacterized protein  (*Loxodonta africana*) | QEVEYRSKLK | Deamidation (NQ),O-2H (Y) | 2 | 1293.66 | 0.83 | 712550 | 86 |
| G3SL16 | Cytochrome P450 family 51 subfamily A member 1  (*Loxodonta africana*) | QSEEKIDDILQTLLDSTYK | Di-iodination | 3 | 2489.92 | 2.70 | 1202400 | 50 |
| G3SL28 | ELKS/RAB6-interacting/CAST family membe  r 1  (*Loxodonta africana*) | KTQEEVAALKR | Acetyl (K),Deamidation (NQ) | 2 | 1314.71 | 3.18 | 188010 | 96 |
| G3SL61 | Mitochondrial ribosomal protein L19  (*Loxodonta africana*) | VTTADPYANGK | Deamidation (NQ),Oxidation (P) | 2 | 1152.53 | -0.79 | 506390 | 90 |
| G3SLA1 | Coiled-coil domain containing 82  (*Loxodonta africana*) | KKYGQLEEYLSLADYFQDEK | Deamidation (NQ),Oxidation Y | 3 | 2483.17 | -3.44 | 404710 | 46 |
| G3SLM5 | Sprouty related EVH1 domain containing 2  (*Loxodonta africana*) | VMHPEGNGR | Oxidation (M),Deamidation (NQ),Oxidation (P) | 2 | 1028.43 | -2.78 | 70318 | 54 |
| G3SM01 | LIM domain 7  (*Loxodonta africana*) | LLQEKYQR | Acetyl (K),2 Deamidation (NQ) | 2 | 1120.58 | 2.02 | 216930 | 63 |
| G3SM16 | Guanylate binding protein family member 6  (*Loxodonta africana*) | EMQRIMEHKLK | Oxidation (M) | 2 | 1457.75 | 2.53 | 966150 | 91 |
| G3SM22 | Uncharacterized protein  (*Loxodonta africana*) | NLPGLQKLDNQTVTEEELSR | Oxidation (P) | 3 | 2299.17 | -4.47 | 834750 | 76 |
| G3SM37 | SWI/SNF related, matrix associated, actin dependent regulator of chromatin, subfamily d, member 1  (*Loxodonta africana*) | RLDIQEALK | Deamidation (NQ) | 2 | 1085.61 | 0.03 | 877120 | 93 |
| G3SM70 | Uncharacterized protein  (*Loxodonta africana*) | SMENNKEAK | Oxidation (M) | 2 | 1065.48 | 1.47 | 384550 | 59 |
| G3SMC0 | Receptor protein-tyrosine kinase, EC 2.7.10.1  (*Loxodonta africana*) | GRQNKAEINPR | Deamidation (NQ) | 2 | 1282.67 | 1.60 | 2947500 | 48 |
| G3SMC8 | Kinesin-like protein  (*Loxodonta africana*) | AIVTEPK | Unmodified | 1 | 756.44 | -0.68 | 148620 | 45 |
| G3SMI5 | HEPACAM family member 2  (*Loxodonta africana*)  (*Loxodonta africana*) | LVYQWLKNGR | Acetyl (K),Deamidation (NQ) | 2 | 1318.70 | -3.84 | 307630 | 62 |
| G3SML6 | HECT domain E3 ubiquitin protein ligase 3  (*Loxodonta africana*) | EVLYKLYKDPAGPSR | dioxidation Y | 2 | 1766.92 | -1.81 | 1354400 | 56 |
| G3SMT8 | Hyaluronan synthase 2, EC 2.4.1.212 (Hyaluronate synthase 2) (Hyaluronic acid synthase 2)  (*Loxodonta africana*) | WLNQQTRWSK | Deamidation (NQ),O-2H (W) | 2 | 1360.65 | -2.55 | 8763000 | 76 |
| G3SMX7 | Uncharacterized protein  (*Loxodonta africana*) | GENFYYKHK | 2 Oxidation Y | 2 | 1216.55 | -4.02 | 1736600 | 52 |
| G3SN02 | Transcription termination factor 1  (*Loxodonta africana*) | FSVKNKQLEK | 2 Deamidation (NQ) | 2 | 1221.66 | 0.65 | 658610 | 48 |
| G3SN10 | Plexin domain containing 2  (*Loxodonta africana*) | NLDSLKAVDTNR | Acetyl (K) | 2 | 1386.71 | 0.15 | 1999600 | 131 |
| G3SN69 | Retroelement silencing factor 1  (*Loxodonta africana*) | EFPYGIEAVNSR | Glu->pyro-Glu,Deamidation (NQ) | 2 | 1363.64 | -2.59 | 491050 | 45 |
| G3SN82 | PDZ binding kinase  (*Loxodonta africana*) | SLNDLIEER | Deamidation (NQ) | 2 | 1088.53 | -0.24 | 2243500 | 77 |
| G3SNW1 | Uncharacterized protein  (*Loxodonta africana*) | SVNGAEIIM | Deamidation (NQ) | 1 | 933.45 | 0.79 | 638690 | 65 |
| G3SP14 | Zinc finger protein-like 1  (*Loxodonta africana*) | AAADSDPNLDPLMNPHIRVGPS | 3 Oxidation (P) | 3 | 2334.09 | 0.12 | 472010 | 44 |
| G3SP74 | RAB8B, member RAS oncogene family  (*Loxodonta africana*) | TSFFRCSLL | Unmodified | 2 | 1129.56 | 2.53 | 139100 | 66 |
| G3SPM4 | PARP-1 binding protein (PARP1-binding protein) (PCNA-interacting partner)  (*Loxodonta africana*) | ERIQKSMQEK | Deamidation (NQ),di-oxidation (M) | 2 | 1308.63 | -2.59 | 4532100 | 80 |
| G3SPN1 | TPX2 microtubule nucleation factor  (*Loxodonta africana*) | PNMVISQEPFVPK | 2 Deamidation (NQ),2 Oxidation (P) | 2 | 1518.73 | 2.32 | 1712500 | 75 |
| G3SPR0 | Component of oligomeric Golgi complex 7 (Conserved oligomeric Golgi complex subunit 7)  (*Loxodonta africana*) | QATKSLPR | Acetyl (K),Deamidation (NQ) | 2 | 942.51 | 0.27 | 465530 | 96 |
| G3SPV8 | C-C motif chemokine  (*Loxodonta africana*) | GAIPLKKLTSWYR | Di-iodination | 2 | 1783.68 | 2.83 | 1709800 | 43 |
| G3SPX0 | Retinoid X receptor beta  (*Loxodonta africana*) | QKYPEQQGR | Deamidation (NQ),Oxidation Y | 2 | 1149.54 | 0.41 | 386810 | 69 |
| G3SQ06 | ASH1 like histone lysine methyltransferase  (*Loxodonta africana*) | KPPKNLENYVCR | 2 Deamidation (NQ) | 2 | 1518.75 | 1.94 | 1406200 | 64 |
| G3SQ67 | Uncharacterized protein  (*Loxodonta africana*) | VNLQGSAKIWGR | 2 Deamidation (NQ),O-2H (W) | 2 | 1343.68 | -2.05 | 145330 | 63 |
| G3SQE4 | Golgi SNAP receptor complex member 1  (*Loxodonta africana*) | GMLKSIQSR | Acetyl (K),Deamidation (NQ),di-oxidation (M) | 2 | 1093.54 | -3.65 | 575620 | 64 |
| G3SQF0 | Dedicator of cytokinesis 7  (*Loxodonta africana*) | IMENNLENELK | Oxidation (M),2 Deamidation (NQ) | 2 | 1363.62 | -2.74 | 446890 | 62 |
| G3SQF9 | Uncharacterized protein  (*Loxodonta africana*) | LWQEVVPQGSNASSLAR | Deamidation (NQ),Oxidation (P) | 3 | 1857.92 | 1.84 | 429220 | 40 |
| G3SQG9 | Connector enhancer of kinase suppressor of Ras 1  (*Loxodonta africana*) | WRRCWFVLK | Di-oxidation W | 2 | 1381.71 | 1.79 | 843280 | 76 |
| G3SQR0 | Band 4.1 (Erythrocyte membrane protein band 4.1) (Protein 4.1)  (*Loxodonta africana*) | GVPWNFTFNVK | Di-oxidation W | 2 | 1339.66 | -0.48 | 1603500 | 63 |
| G3SQR3 | Mitochondrial trans-2-enoyl-CoA reductase  (*Loxodonta africana*) | PEMKNIFKAIPQPR | Deamidation (NQ) | 2 | 1668.90 | 1.87 | 1069800 | 78 |
| G3SQX4 | Solute carrier family 27 member 3  (*Loxodonta africana*) | FSAGQFWEDCQQHR | 2 Deamidation (NQ) | 2 | 1796.72 | -2.87 | 1527800 | 70 |
| G3SR76 | DNA (cytosine-5)-methyltransferase, EC 2.1.1.37  (*Loxodonta africana*) | ISWVGEAVK | Di-oxidation W | 2 | 1019.53 | 0.89 | 3847900 | 57 |
| G3SRU3 | Uncharacterized protein  (*Loxodonta africana*) | VNMDMEKSKLK | Deamidation (NQ) | 2 | 1322.66 | -0.37 | 479010 | 48 |
| G3SS15 | 2-hydroxyacyl-CoA lyase 2 (Acetolactate synthase-like protein) (IlvB-like protein)  (*Loxodonta africana*) | PTAQHLNPVK | 2 Deamidation (NQ),2 Oxidation (P) | 2 | 1137.57 | -0.08 | 955250 | 76 |
| G3SS34 | Mitochondrial calcium uniporter regulator 1  (*Loxodonta africana*) | AENEKIKLELLQLK | 2 Deamidation (NQ) | 2 | 1669.95 | -1.70 | 671930 | 59 |
| G3SS40 | BTB/POZ domain-containing protein 16  (*Loxodonta africana*) | QQKPQLER | Deamidation (NQ),Oxidation (P) | 2 | 1042.54 | -0.37 | 1528000 | 104 |
| G3SS62 | Chromosome alignment maintaining phosphoprotein 1  (*Loxodonta africana*) | SPPLPEQQK | 2 Deamidation (NQ),2 Oxidation (P) | 2 | 1056.50 | 0.83 | 9416800 | 89 |
| G3SS94 | TRAF3 interacting protein 1  (*Loxodonta africana*) | EKDIVSKEIEK | Acetyl (K),Glu->pyro-Glu | 2 | 1340.72 | 0.28 | 962760 | 61 |
| G3SSA1 | Testis expressed 14, intercellular bridge forming factor  (*Loxodonta africana*)  (*Loxodonta africana*) | KNDKPGGNGK | Acetyl (K) | 2 | 1055.54 | 3.38 | 1631800 | 88 |
| G3SSB4 | Furin, paired basic amino acid cleaving enzyme  (*Loxodonta africana*) | EPQVQWLEQQVAKR | Di-oxidation W | 2 | 1769.91 | -0.55 | 285300 | 40 |
| G3SSE9 | Uncharacterized protein  (*Loxodonta africana*) | VQSLCYLQLTR | Deamidation (NQ),Oxidation Y | 2 | 1396.70 | 3.12 | 2104400 | 46 |
| G3SSW2 | Uncharacterized protein  (*Loxodonta africana*) | MRLEQDLKK | Acetyl (K),Deamidation (NQ),di-oxidation (M) | 2 | 1234.62 | -1.08 | 3536300 | 76 |
| G3SSW8 | Uncharacterized protein  (*Loxodonta africana*) | TRLVDAIHSQLTDMEK | Oxidation (M) | 3 | 1871.94 | -2.65 | 197230 | 43 |
| G3ST29 | Glutamate decarboxylase like 1  (*Loxodonta africana*) | MLMAGIQCCALLVK | Deamidation (NQ) | 2 | 1607.79 | -3.53 | 1140500 | 68 |
| G3ST52 | Importin subunit alpha  (*Loxodonta africana*) | LLSKEPNPPIDQVIQK | Deamidation (NQ),Oxidation (P) | 2 | 1835.00 | 0.93 | 3187400 | 63 |
| G3STB0 | Uncharacterized protein  (*Loxodonta africana*) | AAFLDNLMPQMPSELPPSSRR | Unmodified | 3 | 2356.17 | 0.86 | 757070 | 44 |
| G3STQ6 | RING-type E3 ubiquitin transferase, EC 2.3.2.27  (*Loxodonta africana*) | NMGETGPKQR | Oxidation (M),Deamidation (NQ) | 2 | 1133.51 | 1.23 | 449660 | 44 |
| G3STW5 | FIP-RBD domain-containing protein  (*Loxodonta africana*) | EKEGAPPEIKPK | Acetyl (K),Glu->pyro-Glu | 2 | 1345.72 | 0.71 | 132340 | 53 |
| G3STX3 | Phospholipid-transporting ATPase, EC 7.6.2.1  (*Loxodonta africana*) | PESLLETDPVNISLTLKPK | Oxidation (P) | 3 | 2109.16 | -0.08 | 376060 | 45 |
| G3SU16 | Calcium-activated neutral proteinase 1, EC 3.4.22.52 (Calpain mu-type) (Calpain-1 catalytic subunit) (Calpain-1 large subunit) (Micromolar-calpain)  (*Loxodonta africana*) | LEICNLTPDALQSR | 2 Deamidation (NQ),Trioxidation (C) | 2 | 1678.77 | -3.94 | 353110 | 45 |
| G3SU67 | Ganglioside induced differentiation associated protein 1  (*Loxodonta africana*) | RNWGNGKR | Acetyl (K),Deamidation (NQ) | 2 | 1029.51 | -0.11 | 519140 | 89 |
| G3SU74 | Uncharacterized protein  (*Loxodonta africana*) | TLELSDHFIVKVEQAK | Acetyl (K),Deamidation (NQ) | 3 | 1899.00 | -0.90 | 476470 | 44 |
| G3SU96 | Dynein regulatory complex subunit 3  (*Loxodonta africana*) | EELTQAQWEEAQAQK | Deamidation (NQ),Di-oxidation W | 2 | 1820.81 | 1.08 | 4026600 | 54 |
| G3SUG6 | Filamin A interacting protein 1  (*Loxodonta africana*) | SRNQGSESSSNGHISCPK | Deamidation (NQ) | 3 | 1931.84 | -2.55 | 1060400 | 54 |
| G3SUP4 | Kinetochore protein SPC25  (*Loxodonta africana*) | LQFIFTNIDPK | 2 Deamidation (NQ) | 3 | 1336.69 | -3.65 | 2088100 | 46 |
| G3SUR3 | Keratin 35  (*Loxodonta africana*) | KTLSTKAENAR | 2 Acetyl (K),Deamidation (NQ) | 3 | 1302.68 | -0.19 | 1814400 | 74 |
| G3SV62 | Scm like with four mbt domains 1  (*Loxodonta africana*) | KTDLYPIGWCEQNKK | Oxidation (W) | 2 | 1894.92 | 1.15 | 844150 | 70 |
| G3SVE7 | Uncharacterized protein  (*Loxodonta africana*) | EDYKPYQVPK | Iodination,dioxidation Y | 2 | 1423.52 | -3.13 | 249560 | 46 |
| G3SVF1 | Queuine tRNA-ribosyltransferase accessory subunit 2 (Queuine tRNA-ribosyltransferase domain-containing protein 1)  (*Loxodonta africana*) | SVIIGVIEGGDVMEER | Oxidation (M) | 3 | 1717.86 | 2.17 | 4679400 | 64 |
| G3SVI5 | Phospholipase A2, EC 3.1.1.4  (*Loxodonta africana*) | LSEYNILNNSDK | 2 Deamidation (NQ),Oxidation Y | 2 | 1426.65 | 0.04 | 317380 | 44 |
| G3SVS8 | Laminin subunit beta 3  (*Loxodonta africana*) | AQQACSHGACYPPVGDLLIGRTQFLR | Deamidation (NQ),O-2H (Y) | 3 | 2929.40 | -3.77 | 1450300 | 43 |
| G3SVY6 | Cathepsin G  (*Loxodonta africana*) | WNDIMLLQLETR | Deamidation (NQ),di-oxidation (M) | 2 | 1563.76 | -3.86 | 306700 | 57 |
| G3SVZ1 | BEN domain containing 3  (*Loxodonta africana*) | DTEQRRSYQQQR | Deamidation (NQ),O-2H (Y) | 2 | 1608.72 | 3.63 | 193960 | 45 |
| G3SW20 | Chromosome 12 open reading frame 73  (*Loxodonta africana*) | YYRPDLTIPEIPPK | 2 Oxidation (P) | 3 | 1732.90 | 0.60 | 3793000 | 42 |
| G3SWA3 | A-kinase anchoring protein 10  (*Loxodonta africana*) | ASMSVHSPQK | Oxidation (M),Deamidation (NQ) | 2 | 1087.50 | -0.37 | 344030 | 73 |
| G3SWD3 | Keratin 4  (*Loxodonta africana*) | VEIDPEIQK | Unmodified | 2 | 1069.57 | 0.02 | 13570000 | 170 |
| G3SWJ6 | 2-phospho-D-glycerate hydro-lyase, EC 4.2.1.11  (*Loxodonta africana*) | AAVPSGASTGIYEALELR | Unmodified | 2 | 1803.94 | 0.60 | 305420 | 72 |
| G3SWN3 | DDB1 and CUL4 associated factor 8  (*Loxodonta africana*) | QPVLDFESGHK | Gln->pyro-Glu,Oxidation (P) | 2 | 1254.59 | -0.01 | 2827200 | 48 |
| G3SWP9 | G2/M-phase specific E3 ubiquitin protein ligase  (*Loxodonta africana*) | QEFLSLLMQHLENSSLFEGSLSK | 2 Deamidation (NQ) | 3 | 2638.28 | -0.43 | 768580 | 45 |
| G3SX15 | Proto-oncogene c-Ski (Ski oncogene)  (*Loxodonta africana*) | AAEPLSVSIQPRK | 2 Oxidation (P) | 2 | 1426.78 | 3.01 | 5828300 | 95 |
| G3SX18 | FYVE, RhoGEF and PH domain containing 6  (*Loxodonta africana*) | SIEEYAKKR | dioxidation Y | 2 | 1154.59 | 0.49 | 414680 | 56 |
| G3SX27 | SMC5-SMC6 complex localization factor 2  (*Loxodonta africana*) | EKEDTQRLDELQK | 2 Deamidation (NQ) | 2 | 1632.78 | -0.37 | 382960 | 54 |
| G3SX44 | WW domain binding protein 4  (*Loxodonta africana*) | ADYWKSQPK | Deamidation (NQ),Oxidation Y | 2 | 1138.53 | -2.47 | 84334 | 45 |
| G3SX61 | Ectonucleoside triphosphate diphosphohydrolase 7  (*Loxodonta africana*) | DRQYERYLAR | Deamidation (NQ),O-2H (Y) | 2 | 1383.65 | -3.73 | 221190 | 50 |
| G3SXF5 | Uncharacterized protein  (*Loxodonta africana*) | SLPDLPPPKIIPDRK | Oxidation (P) | 3 | 1700.98 | -0.94 | 205810 | 49 |
| G3SXH4 | REST corepressor 2  (*Loxodonta africana*) | KEVQVSQYR | dioxidation Y | 2 | 1167.59 | 0.78 | 1568600 | 78 |
| G3SXP9 | RNA helicase, EC 3.6.4.13  (*Loxodonta africana*) | NVTEMAMNPHIK | 2 Oxidation (M) | 2 | 1415.65 | -1.21 | 317400 | 69 |
| G3SXQ8 | Lysozyme, EC 3.2.1.17  (*Loxodonta africana*) | STDYGIFQINSR | Unmodified | 2 | 1399.67 | -1.12 | 612400 | 111 |
| G3SXV9 | Tudor and KH domain containing  (*Loxodonta africana*) | ASGAKITCDK | Acetyl (K),Trioxidation (C) | 2 | 1139.51 | 1.03 | 329100 | 65 |
| G3SXX6 | Engulfment and cell motility 3  (*Loxodonta africana*) | PELMGLIRQQR | Oxidation (M),2 Deamidation (NQ) | 2 | 1357.70 | -2.56 | 245330 | 62 |
| G3SY20 | Filamin A  (*Loxodonta africana*) | YGGDEIPFSPYRVR | Iodination,dioxidation Y | 2 | 1812.70 | 3.84 | 391870 | 44 |
| G3SY67 | DNA polymerase delta interacting protein 2  (*Loxodonta africana*) | DVASTASEKAENPAGHGSK | Deamidation (NQ) | 3 | 1855.85 | -1.02 | 230880 | 42 |
| G3SYG0 | Zinc finger protein 318  (*Loxodonta africana*) | SLLQNPQDKPVK | Oxidation (P) | 3 | 1381.76 | 0.23 | 10403000 | 76 |
| G3SYY5 | Tankyrase_bdg_C domain-containing protein  (*Loxodonta africana*) | LEWLWDQLK | Deamidation (NQ),Trp->Kynurenine | 2 | 1234.62 | 1.86 | 1949700 | 76 |
| G3SZ07 | DIS3-like exonuclease 1  (*Loxodonta africana*) | KMEIKENLFSNK | Oxidation (M),Deamidation (NQ) | 2 | 1496.75 | 1.65 | 2400700 | 100 |
| G3SZ47 | Trichoplein keratin filament-binding protein  (*Loxodonta africana*) | LRQQWDQNR | Deamidation (NQ),Trp->Kynurenine | 2 | 1247.60 | 1.65 | 2513100 | 63 |
| G3SZ90 | EvC ciliary complex subunit 2  (*Loxodonta africana*) | HQAWWKALEGKLR | Trp->Kynurenine | 3 | 1625.88 | -1.83 | 589320 | 66 |
| G3SZA2 | AarF domain containing kinase 1  (*Loxodonta africana*) | NAEKVAQMLK | Deamidation (NQ),di-oxidation (M) | 2 | 1163.59 | -2.96 | 488320 | 83 |
| G3SZH1 | PDZ domain containing 2  (*Loxodonta africana*) | ESCSPPHSR | Glu->pyro-Glu,Trioxidation (C) | 2 | 1085.42 | 1.37 | 1547900 | 56 |
| G3T0I2 | Zinc finger protein 292  (*Loxodonta africana*) | NWQAYMQYCVLCDKEFLGHR | 3 Deamidation (NQ) | 3 | 2620.12 | -2.10 | 698690 | 49 |
| G3T0M4 | Glomulin, FKBP associated protein  (*Loxodonta africana*) | LDSQGKYTLFR | O-2H (Y) | 2 | 1340.67 | -3.35 | 3087300 | 79 |
| G3T0S5 | Lactotransferrin  (*Loxodonta africana*) | GTNFQLSQLQGK | 2 Deamidation (NQ) | 2 | 1321.65 | -0.56 | 3107700 | 104 |
| G3T0U0 | Transient receptor potential cation channel subfamily V member 4  (*Loxodonta africana*) | LTGTYSIMIQK | Deamidation (NQ),Oxidation Y | 2 | 1270.65 | 1.07 | 173900 | 44 |
| G3T0X2 | Small ArfGAP 1  (*Loxodonta africana*) | EEDNKYCADCEAK | Glu->pyro-Glu,Deamidation (NQ) | 2 | 1613.60 | 2.91 | 432400 | 42 |
| G3T0Z7 | VWFA domain-containing protein  (*Loxodonta africana*) | QLLAEMMQSHVVK | Oxidation (M),2 Deamidation (NQ) | 2 | 1530.74 | 0.05 | 1462100 | 72 |
| G3T125 | Protein kinase domain-containing protein  ù(*Loxodonta africana*) | LIDFGCAKR | Trioxidation (C) | 2 | 1126.54 | -3.16 | 223960 | 69 |
| G3T158 | Uncharacterized protein  (*Loxodonta africana*) | NESIVPFSRYEVKVGVYNNK | O-2H (Y) | 3 | 2355.19 | 2.06 | 349280 | 41 |
| G3T160 | Guanylate cyclase activator 1A (Guanylyl cyclase-activating protein 1)  (*Loxodonta africana*) | VEQKLRWYFK | Oxidation Y | 2 | 1411.76 | 3.95 | 529600 | 72 |
| G3T169 | U3 small nucleolar RNA-associated protein 11, U3 snoRNA-associated protein 11  (*Loxodonta africana*) | LMRTQDVK | Oxidation (M) | 2 | 1005.53 | 0.28 | 604030 | 71 |
| G3T175 | HECT domain E3 ubiquitin protein ligase 4  (*Loxodonta africana*) | EEEACQELLR | Glu->pyro-Glu,Deamidation (NQ) | 2 | 1258.55 | 2.23 | 69888 | 59 |
| G3T1D5 | Serine-protein kinase ATM, EC 2.7.11.1  (*Loxodonta africana*) | TMLAVVDYMR | Oxidation (M),O-2H (Y) | 2 | 1227.56 | -1.76 | 119270 | 49 |
| G3T1I4 | Laminin subunit alpha 3  (*Loxodonta africana*) | AQTLYNNVDR | 2 Deamidation (NQ),Oxidation Y | 2 | 1210.55 | -0.36 | 4515000 | 44 |
| G3T1P5 | Acrosin-binding protein (Acrosin-binding protein, 32 kDa form, mature form) (Acrosin-binding protein, 60 kDa form) (Acrosin-binding protein, mature form) (Proacrosin-binding protein sp32)  (*Loxodonta africana*) | ICDTDYVQYPNYCAFK | Iodination,Di-iodination,O-2H (Y) | 3 | 2447.54 | -0.61 | 7412800 | 40 |
| G3T1S1 | Uncharacterized protein  (*Loxodonta africana*) | RVCAGEGLAR | Unmodified | 2 | 1087.56 | -3.19 | 1015500 | 51 |
| G3T1V3 | Protein tyrosine phosphatase 4A2  (*Loxodonta africana*) | QLLYLEKYR | dioxidation Y,O-2H (Y) | 2 | 1270.66 | -2.62 | 855970 | 59 |
| G3T1X5 | Calcium homeostasis modulator family member 5  (*Loxodonta africana*) | SQVSHLQLSFWKTYAQKEK | Deamidation (NQ),Di-oxidation W | 3 | 2340.18 | 1.87 | 313700 | 41 |
| G3T1Z2 | BCLAF1 and THRAP3 family member 3  (*Loxodonta africana*) | QSVYFTKSNCGKFVQK | Acetyl (K),Gln->pyro-Glu | 2 | 1944.94 | 2.51 | 774140 | 53 |
| G3T225 | Vacuolar protein sorting 13 homolog A  (*Loxodonta africana*) | MLQYKADGIHRK | Oxidation (M) | 2 | 1474.77 | 4.31 | 1519300 | 73 |
| G3T237 | Coronin  (*Loxodonta africana*) | QLALWNPK | 2 Deamidation (NQ) | 2 | 970.51 | -3.06 | 617270 | 74 |
| G3T269 | Kelch like family member 24  (*Loxodonta africana*) | VFEMDPKSLTGR | Oxidation (M),Oxidation (P) | 2 | 1410.68 | 3.12 | 424900 | 53 |
| G3T287 | Proteasome subunit alpha type  (*Loxodonta africana*) | LQMKEMTCR | Oxidation (M) | 2 | 1211.55 | -3.94 | 2252600 | 69 |
| G3T2J1 | Uncharacterized protein  (*Loxodonta africana*) | KLQKPQNSYK | Acetyl (K),Deamidation (NQ) | 2 | 1275.68 | -0.51 | 173810 | 57 |
| G3T2S8 | Coronin  (*Loxodonta africana*) | DAEPILISLR | Unmodified | 1 | 1125.64 | -0.97 | 886290 | 55 |
| G3T2U4 | FRAS1 related extracellular matrix 1  (*Loxodonta africana*) | LPQNGQLQLK | 2 Deamidation (NQ),Oxidation (P) | 2 | 1155.61 | 0.35 | 2484800 | 92 |
| G3T2U6 | Zinc finger E-box binding homeobox 2  (*Loxodonta africana*) | KEFSNSNNLDNK | 2 Deamidation (NQ) | 2 | 1410.63 | 1.82 | 779200 | 53 |
| G3T2U8 | Uncharacterized protein  (*Loxodonta africana*) | ILEDEGFYVQR | Deamidation (NQ),Oxidation Y | 2 | 1384.65 | -2.24 | 865670 | 92 |
| G3T2Y2 | RB transcriptional corepressor like 2  (*Loxodonta africana*) | NVPSEKLER | Deamidation (NQ),Oxidation (P) | 2 | 1087.55 | -0.94 | 359360 | 88 |
| G3T325 | Lactase  (*Loxodonta africana*) | YINEAGLDYYLR | 2 Oxidation Y | 2 | 1520.71 | 3.80 | 1174600 | 76 |
| G3T3D3 | Uncharacterized protein  (*Loxodonta africana*) | TNQLAVGFSDGYLALWNMK | 2 Deamidation (NQ),Di-oxidation W | 2 | 2161.00 | -1.54 | 387430 | 43 |
| G3T3V8 | Bardet-Biedl syndrome 7 protein homolog  (*Loxodonta africana*) | SDNISTISILKDVLSKEATK | 2 Acetyl (K) | 3 | 2245.21 | -4.02 | 2721100 | 57 |
| G3T3W5 | RB binding protein 6, ubiquitin ligase  (*Loxodonta africana*) | EDYAAGQSHRSR | Deamidation (NQ),Oxidation Y | 2 | 1392.60 | 2.29 | 339750 | 64 |
| G3T445 | Growth hormone inducible transmembrane protein  (*Loxodonta africana*) | NQWHLTPSR | 2 Deamidation (NQ),Trp->Kynurenine | 2 | 1143.53 | 1.80 | 165830 | 75 |
| G3T471 | Coiled-coil domain containing 18  (*Loxodonta africana*) | EQSLQKSQEECIKLK | Trioxidation (C) | 2 | 1894.93 | -1.04 | 844150 | 67 |
| G3T4C7 | Uncharacterized protein  (*Loxodonta africana*) | LSMENEELLWK | O-2H (W) | 2 | 1404.66 | 1.72 | 405230 | 80 |
| G3T4F8 | Serine incorporator 5  (*Loxodonta africana*) | WNKNWTAGTATNK | 2 Deamidation (NQ) | 2 | 1492.69 | -3.34 | 784900 | 68 |
| G3T4G2 | Phosphodiesterase 4D interacting protein  (*Loxodonta africana*) | TGLEGKLAEELR | Unmodified | 2 | 1314.71 | 3.94 | 188010 | 75 |
| G3T4I4 | Ribonuclease P, EC 3.1.26.5  (*Loxodonta africana*) | PLNSEEWDKLK | Di-oxidation W | 2 | 1389.68 | 0.31 | 752530 | 64 |
| G3T4L4 | Ornithine carbamoyltransferase, mitochondrial (Ornithine transcarbamylase, mitochondrial)  (*Loxodonta africana*) | VLSSMTDAVLARVYK | O-2H (Y) | 2 | 1665.88 | 1.17 | 1731500 | 57 |
| G3T4W3 | 78 kDa glucose-regulated protein (Binding-immunoglobulin protein) (Heat shock protein 70 family protein 5) (Heat shock protein family A member 5) (Immunoglobulin heavy chain-binding protein)  (*Loxodonta africana*) | SQIFSTASDNQPTVTIK | Unmodified | 2 | 1835.93 | 0.04 | 504470 | 76 |
| G3T5D5 | Synaptotagmin 4  (*Loxodonta africana*) | NEVIGRLVLGAAAEGSGGEHWK | Deamidation (NQ),Di-oxidation W | 3 | 2282.13 | -2.20 | 458730 | 54 |
| G3T5E3 | Uncharacterized protein  (*Loxodonta africana*) | CPPKSPAQCLPK | 3 Oxidation (P) | 2 | 1429.67 | 0.47 | 177300 | 65 |
| G3T5E4 | Coiled-coil domain containing 152  (*Loxodonta africana*) | SHEQEYKNNIVK | Deamidation (NQ),O-2H (Y) | 2 | 1502.70 | 1.58 | 924140 | 54 |
| G3T5J3 | Ubiquitin protein ligase E3 component n-recognin 4  (*Loxodonta africana*) | ALATNPALR | Deamidation (NQ) | 2 | 926.52 | 0.86 | 553940 | 74 |
| G3T5K7 | Dipeptidase, EC 3.4.13.19  (*Loxodonta africana*) | GWSEEELWGVLR | Oxidation (W),Di-oxidation W | 2 | 1507.69 | 2.58 | 290780 | 57 |
| G3T5M1 | ALMS1 centrosome and basal body associated protein  (*Loxodonta africana*) | EWSSRQQQQKNK | Glu->pyro-Glu,4 Deamidation (NQ) | 2 | 1531.69 | 1.86 | 580230 | 74 |
| G3T5Q2 | MANSC domain containing 4  (*Loxodonta africana*) | LSNIPIPSQLNSSK | 2 Deamidation (NQ),2 Oxidation (P) | 2 | 1530.78 | 0.80 | 271860 | 48 |
| G3T5T7 | Calcium dependent secretion activator  (*Loxodonta africana*) | LSEYAKIEGKK | dioxidation Y | 2 | 1296.69 | 0.87 | 331990 | 70 |
| G3T5V7 | E2F associated phosphoprotein  (*Loxodonta africana*) | SYHGFGIQRPRQQR | Oxidation Y | 2 | 1744.89 | -1.04 | 203700 | 57 |
| G3T5W0 | SOS Ras/Rac guanine nucleotide exchange factor 1  (*Loxodonta africana*) | EAVQYVLPR | Oxidation Y | 2 | 1089.58 | 3.79 | 936760 | 82 |
| G3T662 | WD_REPEATS_REGION domain-containing protein  (*Loxodonta africana*) | LNRYLVTGDLDGWLK | Deamidation (NQ),Trp->Kynurenine | 2 | 1766.92 | -0.97 | 926240 | 47 |
| G3T6B5 | KCNQ_channel domain-containing protein  (*Loxodonta africana*) | ERVRMASPR | Oxidation (M) | 2 | 1116.58 | -2.77 | 2967900 | 74 |
| G3T6V8 | Coiled-coil domain containing 66  (*Loxodonta africana*) | GEKNKMTFSPTK | Oxidation (M),Deamidation (NQ) | 2 | 1383.67 | -0.78 | 23727000 | 84 |
| G3T6W1 | DUF3715 domain-containing protein  (*Loxodonta africana*) | ALDPTPKHECHVSK | Oxidation (P) | 2 | 1633.79 | -1.75 | 3221800 | 94 |
| G3T768 | RNA exonuclease 4  (*Loxodonta africana*) | EVNKKQGSGPR | 2 Deamidation (NQ) | 2 | 1200.61 | -0.19 | 3926900 | 45 |
| G3T7E3 | Zinc finger ZZ-type containing 3  (*Loxodonta africana*) | PEPVQIQK | Deamidation (NQ),2 Oxidation (P) | 2 | 970.50 | -0.52 | 4939000 | 105 |
| G3T7F3 | RAB11A, member RAS oncogene family  (*Loxodonta africana*) | AQIWDTAGQERYR | Deamidation (NQ),dioxidation Y | 2 | 1625.74 | -1.05 | 166700 | 62 |
| G3T7I0 | Non-specific serine/threonine protein kinase, EC 2.7.11.1  (*Loxodonta africana*) | SEPPALAQPSR | Deamidation (NQ),2 Oxidation (P) | 2 | 1184.57 | 0.74 | 5647000 | 99 |
| G3T7S4 | Tudor domain containing 1  (*Loxodonta africana*) | SLAEYCQQK | Unmodified | 2 | 1125.51 | 4.40 | 274760 | 84 |
| G3T850 | Eyes absent homolog, EC 3.1.3.48  (*Loxodonta africana*) | VKEMYNTYK | Acetyl (K),di-oxidation (M) | 2 | 1248.57 | 4.11 | 1162500 | 66 |
| G3T8D1 | Uncharacterized protein  (*Loxodonta africana*) | NCIGKQFAMNKLK | 2 Deamidation (NQ) | 2 | 1552.77 | -0.52 | 1286600 | 86 |
| G3T8G5 | Threonyl-tRNA synthetase, EC 6.1.1.3  (*Loxodonta africana*) | PMNCPGHCLMFAHR | Oxidation (M),2 Trioxidation (C) | 2 | 1838.70 | 1.91 | 759290 | 44 |
| G3T8H2 | Phosphoglycerate kinase, EC 2.7.2.3  (*Loxodonta africana*) | NNQITNNQR | 2 Deamidation (NQ) | 2 | 1102.50 | -2.14 | 96347 | 56 |
| G3T8I3 | EF-hand domain-containing protein  (*Loxodonta africana*) | IDATRMGYR | Oxidation (M),Oxidation Y | 2 | 1113.52 | -2.11 | 379470 | 49 |
| G3T8N8 | Uncharacterized protein  (*Loxodonta africana*) | QWNNCAFLESSAKSK | Acetyl (K),Gln->pyro-Glu | 2 | 1793.80 | 2.87 | 10870000 | 52 |
| G3T8V1 | Mitochondrial ribosome recycling factor  (*Loxodonta africana*) | QNTNKAKDSLR | 3 Deamidation (NQ) | 3 | 1276.63 | 0.50 | 2070600 | 66 |
| G3T938 | Cyclin dependent kinase 16  (*Loxodonta africana*) | LGEGTYATVYKGKSK | O-2H (Y) | 2 | 1614.83 | -0.57 | 112290 | 52 |
| G3T9C6 | Nucleoporin 43  (*Loxodonta africana*) | DRIEITSLLPNR | Deamidation (NQ) | 2 | 1426.78 | 2.16 | 5828300 | 68 |
| G3T9S0 | Receptor tyrosine kinase like orphan receptor 1  (*Loxodonta africana*) | ISDLGLSR | Unmodified | 2 | 859.48 | 3.35 | 8895700 | 128 |
| G3T9Y7 | Plakophilin 1  (*Loxodonta africana*) | SPNQNVQQAAAGALR | Unmodified | 2 | 1523.78 | 0.36 | 1028400 | 179 |
| G3TA01 | Otoferlin  (*Loxodonta africana*) | LRGVLEELSCGCYR | Oxidation Y | 2 | 1726.81 | 0.09 | 1819500 | 70 |
| G3TA68 | NACHT domain-containing protein  (*Loxodonta africana*) | LDIKEIFSTGPRTTPK | Unmodified | 3 | 1801.99 | 1.79 | 781160 | 53 |
| G3TA84 | Proteasome subunit beta  (*Loxodonta africana*) | LAAIAESGVER | Unmodified | 2 | 1114.60 | -0.18 | 537440 | 96 |
| G3TAK1 | Complement C5  (*Loxodonta africana*) | YILSPYKLNLVATSLFLK | Iodination,Oxidation Y | 3 | 2224.10 | 3.85 | 5680800 | 92 |
| G3TAL6 | Nucleolin  (*Loxodonta africana*) | IIDSIWCFR | Trioxidation (C) | 2 | 1256.59 | 2.03 | 745960 | 85 |
| G3TAQ7 | Uncharacterized protein  (*Loxodonta africana*) | EGKESKEVR | 2 Acetyl (K),Glu->pyro-Glu | 2 | 1126.56 | 0.31 | 716200 | 76 |
| G3TAU0 | Matrix metallopeptidase 8  (*Loxodonta africana*) | IVQNYLEKFYR | Deamidation (NQ),dioxidation Y | 2 | 1504.76 | -3.29 | 4124300 | 73 |
| G3TAX5 | KIAA0355  (*Loxodonta africana*) | MYCCSAQDSK | Oxidation (M),2 Trioxidation (C) | 2 | 1360.42 | -3.57 | 4437100 | 54 |
| G3TB22 | Centrosomal protein 290  (*Loxodonta africana*) | EILQAIKEMQK | Acetyl (K),Oxidation (M),Deamidation (NQ) | 2 | 1388.72 | 2.61 | 98048 | 80 |
| G3TB23 | DNA polymerase kappa  (*Loxodonta africana*) | RSEMKWNHQDTFK | 2 Deamidation (NQ) | 2 | 1707.77 | 0.30 | 263690 | 50 |
| G3TBH6 | GCS light chain (Gamma-ECS regulatory subunit) (Gamma-glutamylcysteine synthetase regulatory subunit) (Glutamate--cysteine ligase modifier subunit)  (*Loxodonta africana*) | ARTLHLQTGNLLNWGR | 2 Deamidation (NQ),O-2H (W) | 2 | 1864.95 | 0.44 | 367180 | 55 |
| G3TBN9 | Axin 2  (*Loxodonta africana*) | ANGQVSLPHFPRTHR | 2 Deamidation (NQ) | 3 | 1717.86 | -2.64 | 4807400 | 48 |
| G3TBS8 | CREB3 regulatory factor  (*Loxodonta africana*) | YFWEYSEQLTPSQQERMLR | Oxidation (M),Deamidation (NQ) | 3 | 2507.14 | 3.95 | 516580 | 42 |
| G3TBY0 | Dihydropyrimidinase like 5  (*Loxodonta africana*) | GVNSFQMSMTYK | Oxidation (M),Deamidation (NQ),di-oxidation (M) | 2 | 1440.59 | 1.97 | 387660 | 48 |
| G3TCD8 | Polyadenylate-binding protein, PABP  (*Loxodonta africana*) | EERQAHLTNQYMQR | Oxidation (M),2 Deamidation (NQ) | 2 | 1820.81 | -1.62 | 4026600 | 52 |
| G3TCH1 | Fatty acid-binding protein 1 (Fatty acid-binding protein, liver) (Liver-type fatty acid-binding protein)  (*Loxodonta africana*) | TVVNLEGNNK | Deamidation (NQ) | 2 | 1087.55 | 2.94 | 666140 | 72 |
| G3TCV2 | Clathrin heavy chain  (*Loxodonta africana*) | WLLLTGISAQQNR | 2 Deamidation (NQ),Trp->Kynurenine | 2 | 1504.79 | -1.43 | 1388400 | 55 |
| G3TCW0 | Nucleolar and coiled-body phosphoprotein 1  (*Loxodonta africana*) | TLQKAAGTVTPSKPASK | Deamidation (NQ) | 2 | 1684.94 | -0.38 | 1462000 | 77 |
| G3TD18 | GATOR complex protein WDR24  (*Loxodonta africana*) | QDQLFTEHK | Gln->pyro-Glu | 2 | 1127.52 | 0.19 | 355940 | 57 |
| G3TD90 | Inter-alpha-trypsin inhibitor heavy chain 2  (*Loxodonta africana*) | NPKKPEASMEVK | Deamidation (NQ),di-oxidation (M) | 2 | 1389.68 | -1.36 | 434100 | 54 |
| G3TD91 | Activated leukocyte cell adhesion molecule (CD166 antigen)  (*Loxodonta africana*) | DLGNLEENKK | 2 Deamidation (NQ) | 2 | 1160.56 | -0.04 | 1807300 | 56 |
| G3TDA6 | ADAM metallopeptidase domain 23  (*Loxodonta africana*) | CLQIQALNMSSCPLDSK | Oxidation (M),2 Deamidation (NQ) | 2 | 1981.88 | 0.52 | 477290 | 55 |
| G3TDK1 | Cyclin dependent kinase like 2  (*Loxodonta africana*) | ALEDIAQNSR | 2 Deamidation (NQ) | 2 | 1117.53 | -2.55 | 243200 | 61 |
| G3TDN7 | Stress induced phosphoprotein 1  (*Loxodonta africana*) | KCQQAEKILK | Deamidation (NQ),Trioxidation (C) | 2 | 1293.66 | -3.54 | 1036100 | 108 |
| G3TDS8 | Serine peptidase inhibitor, Kunitz type 1  (*Loxodonta africana*) | WYYSPPEQVCK | 2 dioxidation Y | 2 | 1519.63 | -1.60 | 575730 | 46 |
| G3TDU4 | ATP synthase subunit alpha  (*Loxodonta africana*) | AVDSLVPIGR | Unmodified | 2 | 1025.59 | 0.63 | 391150 | 102 |
| G3TE09 | Golgin B1  (*Loxodonta africana*) | ELQSNKELVKSQMK | 3 Deamidation (NQ) | 2 | 1663.83 | 0.12 | 561160 | 47 |
| G3TED3 | Guanylate cyclase, EC 4.6.1.2  (*Loxodonta africana*) | NVQGDLPQRKTSR | Acetyl (K),Deamidation (NQ) | 3 | 1540.80 | 0.60 | 254640 | 50 |
| G3TEE5 | ADAM metallopeptidase with thrombospondin type 1 motif 17  (*Loxodonta africana*) | RGLWEKMSAK | Acetyl (K),di-oxidation (M) | 2 | 1278.64 | -2.64 | 878790 | 45 |
| G3TEF2 | DnaJ heat shock protein family (Hsp40) member B12  (*Loxodonta africana*) | AIQSNQPDR | 2 Deamidation (NQ) | 2 | 1029.47 | -2.11 | 299060 | 67 |
| G3TES7 | Proteasome subunit beta  (*Loxodonta africana*) | ATAGAYIASQTVK | Unmodified | 2 | 1279.68 | 0.33 | 176880 | 95 |
| G3TEU1 | G_PROTEIN_RECEP_F3_4 domain-containing protein  (*Loxodonta africana*) | VQEESLAGDLDTPALQKR | Unmodified | 3 | 1969.01 | 0.58 | 2118800 | 71 |
| G3TEV7 | Tudor domain-containing protein 5  (*Loxodonta africana*) | QEGLGAQEK | Deamidation (NQ) | 1 | 959.46 | 3.76 | 172280 | 43 |
| G3TF62 | Polycystin 1, transient receptor potential channel interacting  (*Loxodonta africana*) | LNQATEEVYQLER | 2 Deamidation (NQ),O-2H (Y) | 2 | 1607.73 | -2.44 | 587270 | 60 |
| G3TFA6 | KIAA1755  (*Loxodonta africana*) | SSWPLRATR | Di-oxidation W | 2 | 1104.57 | 0.97 | 158380 | 45 |
| G3TFF5 | Zona pellucida glycoprotein 2  (*Loxodonta africana*) | NINVSQLHDNGIER | 3 Deamidation (NQ) | 2 | 1610.75 | -4.34 | 375410 | 46 |
| G3TFH5 | R3H domain containing like  (*Loxodonta africana*) | SLTGLGVPR | Unmodified | 2 | 898.52 | -0.06 | 57381000 | 80 |
| G3TFJ0 | Receptor protein-tyrosine kinase, EC 2.7.10.1  (*Loxodonta africana*) | KLSIAESACGK | Trioxidation (C) | 2 | 1210.59 | -0.27 | 129240 | 50 |
| G3TFN4 | Ribosome binding protein 1  (*Loxodonta africana*) | EMQQLQGKVR | 2 Deamidation (NQ),di-oxidation (M) | 2 | 1249.60 | -2.34 | 193010 | 72 |
| G3TFU1 | AP complex subunit beta  (*Loxodonta africana*) | DSDYYNMLLK | Oxidation (M),O-2H (Y) | 2 | 1290.54 | 3.57 | 108880 | 51 |
| G3TG61 | Homeobox domain-containing protein  (*Loxodonta africana*) | QEIAQTVGVAPQR | 2 Deamidation (NQ) | 2 | 1397.72 | 0.67 | 4713700 | 76 |
| G3TGA6 | NIMA related kinase 4  (*Loxodonta africana*) | VNINILPAER | Deamidation (NQ) | 2 | 1138.63 | 0.58 | 458100 | 80 |
| G3TGL3 | Solute carrier family 8 member A3  (*Loxodonta africana*) | EMIRILKDLK | Acetyl (K),Oxidation (M) | 2 | 1315.75 | -1.05 | 3138400 | 49 |
| G3TH71 | Family with sequence similarity 81 member B  (*Loxodonta africana*) | LEKSENKMEEK | Acetyl (K),Deamidation (NQ),di-oxidation (M) | 2 | 1438.65 | 3.93 | 199860 | 55 |
| G3TH82 | Tetratricopeptide repeat domain 37  (*Loxodonta africana*) | LALVNNTQPKR | 2 Deamidation (NQ) | 2 | 1254.69 | -2.41 | 159480 | 46 |
| G3THF8 | Cadherin-1 (E-Cad/CTF1) (E-Cad/CTF2) (E-Cad/CTF3) (Epithelial cadherin)  (*Loxodonta africana*) | LKVTDADVPDTPAWK | Oxidation (W) | 3 | 1670.85 | 3.14 | 588640 | 59 |
| G3THU4 | SH3 domain binding protein 2  (*Loxodonta africana*) | GEPQNGLYCIRNSSTKSGK | Deamidation (NQ),dioxidation Y | 3 | 2127.99 | 3.50 | 16321000 | 52 |
| G3TI97 | Beta-transducin repeat containing E3 ubiquitin protein ligase  (*Loxodonta africana*) | VTSDGMLWK | Oxidation (M),O-2H (W) | 2 | 1065.48 | -2.18 | 384550 | 68 |
| G3TIB8 | Proteasome subunit alpha type  (*Loxodonta africana*) | ITSPLMEPSSIEK | Oxidation (M) | 2 | 1446.73 | 0.39 | 243640 | 90 |
| G3TIG7 | Non-specific serine/threonine protein kinase, EC 2.7.11.1  (*Loxodonta africana*) | SLDWNSLLRQK | Unmodified | 2 | 1358.73 | -0.05 | 3053600 | 115 |
| G3TIH5 | Cullin 7  (*Loxodonta africana*) | EPKTQCSTTPLQHLVEGYGPAGK | Deamidation (NQ),Oxidation Y | 3 | 2514.21 | 1.64 | 5422400 | 40 |
| G3TII7 | Repulsive guidance molecule BMP co-receptor a  (*Loxodonta africana*) | AGWMGMGRGAGR | Oxidation (M) | 3 | 1221.55 | 3.43 | 738510 | 56 |
| G3TIJ0 | Transcription factor EC  (*Loxodonta africana*) | WLQKEQQRAR | Deamidation (NQ),O-2H (W) | 2 | 1356.69 | -0.82 | 462410 | 80 |
| G3TIR5 | Proteasome subunit alpha type  (*Loxodonta africana*) | AINQGGLTSVAVR | Unmodified | 2 | 1284.72 | 1.08 | 329310 | 92 |
| G3TJ33 | SEC7 domain-containing protein  (*Loxodonta africana*) | QAEEEAIKRSR | Acetyl (K),Gln->pyro-Glu | 2 | 1340.67 | 0.75 | 8913500 | 58 |
| G3TJB6 | Gamma-tubulin complex component  (*Loxodonta africana*) | RIQEFQESIPK | 2 Deamidation (NQ) | 2 | 1375.70 | 1.58 | 8096500 | 71 |
| G3TJG5 | Tensin 4  (*Loxodonta africana*) | VTEQGITLTDVQR | Deamidation (NQ) | 2 | 1459.75 | -0.28 | 1834700 | 71 |
| G3TJL4 | Centrosomal protein 83  (*Loxodonta africana*) | YNQAREKLQR | Iodination | 2 | 1430.59 | -3.68 | 169390 | 85 |
| G3TJY1 | Disco interacting protein 2 homolog A  (*Loxodonta africana*) | KAILSMSGLSYGVIR | dioxidation Y | 2 | 1625.88 | -3.65 | 714230 | 65 |
| G3TJY5 | NOP14 nucleolar protein  (*Loxodonta africana*) | KGQLQTPGKR | 2 Acetyl (K),Deamidation (NQ) | 2 | 1196.65 | -1.27 | 3694700 | 86 |
| G3TKC3 | VWFA domain-containing protein  (*Loxodonta africana*) | TKAEMIKATTR | Acetyl (K) | 3 | 1290.70 | -2.54 | 194000 | 42 |
| G3TKL7 | Ecotropic viral integration site 5  (*Loxodonta africana*) | QAEIECKSK | Trioxidation (C) | 2 | 1139.51 | 1.45 | 239380 | 62 |
| G3TKN5 | Myotubularin related protein 10  (*Loxodonta africana*) | FRFDESGPESAKK | Oxidation (P) | 3 | 1512.72 | 3.19 | 5250500 | 52 |
| G3TKT5 | Dynein cytoplasmic 1 intermediate chain 1  (*Loxodonta africana*) | KKEADMQQK | Oxidation (M),Deamidation (NQ) | 2 | 1121.54 | -0.87 | 3360900 | 59 |
| G3TKW3 | Cysteine and glycine rich protein 3  (*Loxodonta africana*) | CGKSVYAAEKVMGGGK | Acetyl (K),Trioxidation (C) | 2 | 1730.80 | -0.22 | 338830 | 48 |
| G3TKX6 | RING-type domain-containing protein  (*Loxodonta africana*) | RAAIMRGAQQQR | 2 Deamidation (NQ) | 2 | 1386.72 | -2.74 | 1999600 | 102 |
| G3TL45 | Nuclear receptor corepressor 1  (*Loxodonta africana*) | LENTSPMVKSKK | Oxidation (M),Oxidation (P) | 3 | 1392.73 | -2.14 | 632880 | 47 |
| G3TM07 | RGS domain-containing protein  (*Loxodonta africana*) | ACNENDVILMRSKINVILK | Deamidation (NQ) | 3 | 2230.18 | 3.14 | 1248500 | 66 |
| G3TM74 | Steroid receptor RNA activator 1  (*Loxodonta africana*) | LALLQEQWAGGK | Deamidation (NQ),Di-oxidation W | 2 | 1345.69 | -1.37 | 927030 | 46 |
| G3TMX8 | Uncharacterized protein  (*Loxodonta africana*) | GILANSEER | Deamidation (NQ) | 2 | 988.48 | -0.27 | 1185200 | 77 |
| G3TN62 | 2-oxoisovalerate dehydrogenase subunit alpha, EC 1.2.4.4 (Branched-chain alpha-keto acid dehydrogenase E1 component alpha chain)  (*Loxodonta africana*) | EAGGVLMYR | Oxidation (M),O-2H (Y) | 2 | 1024.46 | -2.73 | 1365500 | 67 |
| G3TN87 | Uncharacterized protein  (*Loxodonta africana*) | KEMKQDTLR | Acetyl (K),di-oxidation (M) | 2 | 1221.60 | -3.17 | 618180 | 93 |
| G3TND3 | Component of oligomeric Golgi complex 3 (Conserved oligomeric Golgi complex subunit 3)  (*Loxodonta africana*) | DAAFKILNPMTVPR | Oxidation (M),Deamidation (NQ) | 2 | 1588.83 | -3.70 | 1012700 | 52 |
| G3TNV5 | RF_PROK_I domain-containing protein  (*Loxodonta africana*) | LYQQIVEKDR | Deamidation (NQ),O-2H (Y) | 2 | 1305.66 | 0.84 | 175510 | 50 |
| G3TNX2 | Protein maelstrom homolog  (*Loxodonta africana*) | LLESISSCSSNIHK | Deamidation (NQ),Trioxidation (C) | 2 | 1622.75 | -2.03 | 1432200 | 71 |
| G3TP91 | MAGE domain-containing protein  (*Loxodonta africana*) | AREKCHQAK | Unmodified | 2 | 1126.57 | -3.30 | 2198200 | 93 |
| G3TPL5 | Agrin  (*Loxodonta africana*) | TLEPQGLLLYNGNAR | 3 Deamidation (NQ) | 2 | 1660.83 | 1.04 | 419600 | 59 |
| G3TQ25 | Anaphase-promoting complex subunit 1  (*Loxodonta africana*) | WVDSNVPQIIR | Deamidation (NQ),O-2H (W) | 2 | 1340.67 | -2.06 | 8913500 | 64 |
| G3TQ99 | CCR4-NOT transcription complex subunit 3  (*Loxodonta africana*) | YEADLKKEIK | Unmodified | 2 | 1235.68 | -1.47 | 129090 | 80 |
| G3TQA4 | PYM homolog 1, exon junction complex associated factor  (*Loxodonta africana*) | RALEEELEDLELGL | Unmodified | 3 | 1627.83 | 4.10 | 1127900 | 45 |
| G3TQH8 | Cytochrome P450 family 26 subfamily A member 1  (*Loxodonta africana*) | IEENIRSKICR | Acetyl (K) | 3 | 1458.76 | -2.19 | 3757100 | 50 |
| G3TQI5 | Uncharacterized protein  (*Loxodonta africana*) | SILNQHIECQR | Deamidation (NQ),Trioxidation (C) | 2 | 1445.66 | -2.06 | 1463300 | 51 |
| G3TQJ5 | G protein-coupled receptor 151  (*Loxodonta africana*) | TQNLRNQIRSK | 2 Deamidation (NQ) | 2 | 1358.73 | 3.93 | 2610000 | 44 |
| G3TQM2 | Tubulin tyrosine ligase like 10  (*Loxodonta africana*) | INKTSQSNQAK | 2 Deamidation (NQ) | 2 | 1219.60 | -0.18 | 176050 | 64 |
| G3TQS8 | ATP binding cassette subfamily A member 2  (*Loxodonta africana*) | EMDKMIEDLELSNK | Oxidation (M),Glu->pyro-Glu | 2 | 1691.77 | 3.11 | 186300 | 46 |
| G3TRA2 | Ras responsive element binding protein 1  (*Loxodonta africana*) | SLGGCQKAR | Deamidation (NQ),Trioxidation (C) | 2 | 1024.46 | 0.79 | 1365500 | 65 |
| G3TRM0 | Ankyrin repeat domain 17  (*Loxodonta africana*) | TPLMKAAR | Oxidation (P) | 1 | 902.50 | -4.43 | 1243400 | 57 |
| G3TRR6 | Uncharacterized protein  (*Loxodonta africana*) | IIAMSFPSSGKQSFYRNPIK | O-2H (Y) | 3 | 2284.17 | -4.26 | 223090 | 41 |
| G3TRW7 | Phosphatidylinositol-3,4,5-trisphosphate 5-phosphatase, EC 3.1.3.86  (*Loxodonta africana*) | GGLKELTDLDYR | Oxidation Y | 2 | 1394.70 | 1.16 | 268300 | 59 |
| G3TRX4 | WD repeat domain 93  (*Loxodonta africana*) | IQPTIYSPLEEIK | Deamidation (NQ) | 2 | 1530.82 | -1.67 | 12188000 | 82 |
| G3TSC0 | RBBP8 N-terminal like  (*Loxodonta africana*) | LPSSGGMVNEPR | Deamidation (NQ),Oxidation (P) | 2 | 1259.58 | 2.17 | 5776400 | 102 |
| G3TSK2 | Neuregulin 2  (*Loxodonta africana*) | QNMCPAHQNR | 2 Deamidation (NQ),Trioxidation (C) | 2 | 1304.49 | 1.68 | 16114000 | 51 |
| G3TSU4 | 59 kDa serine/threonine-protein kinase (Beta-integrin-linked kinase) (ILK-1) (ILK-2) (Integrin-linked protein kinase) (p59ILK)  (*Loxodonta africana*) | FDMIVPILEKMQDK | Oxidation (M),Deamidation (NQ),di-oxidation (M) | 2 | 1754.85 | 2.54 | 1071400 | 89 |
| G3TSW9 | Solute carrier family 25 member 43  (*Loxodonta africana*) | TQGMLGLWNGLTANLLK | Deamidation (NQ) | 3 | 1829.97 | 1.10 | 1401000 | 55 |
| G3TSZ9 | Nucleolar complex protein 3 homolog, NOC3 protein homolog  (*Loxodonta africana*) | NKQFKQQSTLK | Acetyl (K),2 Deamidation (NQ) | 3 | 1392.72 | 0.39 | 632880 | 51 |
| G3TT32 | ArfGAP with GTPase domain, ankyrin repeat and PH domain 3  (*Loxodonta africana*) | VIDDSRARK | Unmodified | 2 | 1058.58 | 2.78 | 1342900 | 108 |
| G3TT37 | Mucolipin 2  (*Loxodonta africana*) | ITYDNKAHSGKIK | Deamidation (NQ) | 3 | 1474.78 | 0.55 | 262770 | 65 |
| G3TT72 | Bromodomain and WD repeat domain containing 1  (*Loxodonta africana*) | DLQAWRRR | O-2H (W) | 2 | 1113.58 | -0.51 | 359050 | 69 |
| G3TTL3 | Uncharacterized protein  (*Loxodonta africana*) | LEAKLQAK | Deamidation (NQ) | 2 | 900.53 | 0.59 | 126280 | 69 |
| G3TTQ0 | Poly [ADP-ribose] polymerase, PARP, EC 2.4.2.-  (*Loxodonta africana*) | KNYDRVMK | Acetyl (K),Deamidation (NQ),di-oxidation (M) | 2 | 1127.53 | -2.34 | 265270 | 53 |
| G3TTU7 | Trafficking kinesin protein 2  (*Loxodonta africana*) | ELLQGETNAQMSR | 2 Deamidation (NQ),di-oxidation (M) | 2 | 1509.66 | -3.48 | 673450 | 59 |
| G3TU16 | Endoribonuclease Dicer, EC 3.1.26.3  (*Loxodonta africana*) | SLPADFRYPNLDFGWK | Oxidation (P) | 3 | 1940.94 | -3.42 | 1529300 | 46 |
| G3TU29 | Uncharacterized protein  (*Loxodonta africana*) | QALIEKKVAYHLQK | 2 Deamidation (NQ) | 2 | 1669.94 | 4.27 | 1169700 | 41 |
| G3TU31 | MDS1 and EVI1 complex locus  (*Loxodonta africana*) | HKRMHADCR | Acetyl (K),Oxidation (M) | 2 | 1267.57 | 2.52 | 121230 | 56 |
| G3TUK4 | Voltage-dependent T-type calcium channel subunit alpha  (*Loxodonta africana*) | MSYDQRSLSSSR | Oxidation (M),dioxidation Y | 2 | 1463.63 | 1.08 | 5141500 | 50 |
| G3TUM1 | Flotillin  (*Loxodonta africana*) | EMLDVKFMADTK | 2 Oxidation (M),Glu->pyro-Glu | 2 | 1440.66 | 0.01 | 185020 | 84 |
| G3TUN1 | Bardet-Biedl syndrome 5 protein homolog  (*Loxodonta africana*) | MSVLDALWEDR | Oxidation (M),O-2H (W) | 2 | 1363.61 | 2.29 | 446890 | 81 |
| G3TV17 | Protein-tyrosine-phosphatase, EC 3.1.3.48  (*Loxodonta africana*) | PNSLYYFR | Deamidation (NQ),Oxidation (P) | 2 | 1075.50 | -3.24 | 761320 | 71 |
| G3TV47 | Transportin 1  (*Loxodonta africana*) | GELQNWPDLLPK | Deamidation (NQ) | 3 | 1409.72 | -2.74 | 230090 | 61 |
| G3TVK7 | Solute carrier family 12 member 1  (*Loxodonta africana*) | VANGDGMPGDEQTENK | Oxidation (M),2 Deamidation (NQ) | 2 | 1678.66 | 4.47 | 259820 | 55 |
| G3TVP3 | Helix-destabilizing protein (Heterogeneous nuclear ribonucleoprotein A1) (Heterogeneous nuclear ribonucleoprotein A1, N-terminally processed) (hnRNP core protein A1)  (*Loxodonta africana*) | SPKEPEQLRK | Oxidation (P) | 2 | 1226.66 | -0.84 | 2045700 | 109 |
| G3TVV3 | Low-density lipoprotein receptor relative with 11 ligand-binding repeats (Sortilin-related receptor) (Sorting protein-related receptor containing LDLR class A repeats)  (*Loxodonta africana*) | IITENDHVLLFWK | Oxidation (W) | 2 | 1642.87 | 3.79 | 199140 | 54 |
| G3TVX7 | Sperm associated antigen 1  (*Loxodonta africana*) | IKLQNWNSAFQDCEK | Deamidation (NQ),Trioxidation (C) | 3 | 1928.86 | 2.04 | 1038200 | 69 |
| G3TW05 | Zinc finger protein 784  (*Loxodonta africana*) | LPEAAAAPEQR | Deamidation (NQ),2 Oxidation (P) | 2 | 1184.57 | 0.86 | 4968000 | 76 |
| G3TW77 | Uncharacterized protein  (*Loxodonta africana*) | LLQDFFNGK | 2 Deamidation (NQ) | 2 | 1082.53 | -4.07 | 727470 | 74 |
| G3TWD0 | Carnosine dipeptidase 1  (*Loxodonta africana*) | FSSPAPPSGLLEK | 2 Oxidation (P) | 2 | 1360.69 | -2.66 | 224510 | 76 |
| G3TWF3 | Musculoskeletal embryonic nuclear protein 1  (*Loxodonta africana*) | NQEIKSKTYQVMR | 2 Deamidation (NQ),di-oxidation (M) | 2 | 1657.80 | 3.88 | 526310 | 43 |
| G3TWG1 | Sodium channel protein  (*Loxodonta africana*) | VLGESGEMDALK | Unmodified | 2 | 1247.61 | -2.30 | 2398800 | 102 |
| G3TWK1 | Uncharacterized protein  (*Loxodonta africana*) | INYCYRGQCR | Deamidation (NQ),2 O-2H (Y) | 2 | 1417.55 | -3.37 | 159530 | 44 |
| G3TWM3 | Phosphatidylinositol-3,4-bisphosphate 4-phosphatase, EC 3.1.3.66  (*Loxodonta africana*) | NVDILWQAAEVCRR | Deamidation (NQ),O-2H (W) | 2 | 1743.84 | 1.22 | 724410 | 51 |
| G3TWU3 | Pleckstrin homology domain containing A1  (*Loxodonta africana*) | QGEYPCAKKQVSYR | O-2H (Y) | 2 | 1726.81 | 2.04 | 1819500 | 72 |
| G3TWX4 | Kinesin family member 26B  (*Loxodonta africana*) | KSSLDQKNR | 2 Acetyl (K) | 2 | 1158.60 | -0.26 | 134000 | 52 |
| G3TX86 | Kinesin family member 13B  (*Loxodonta africana*) | LSPEKQNYR | Deamidation (NQ),dioxidation Y | 2 | 1166.56 | -2.24 | 1204100 | 72 |
| G3TXA8 | NTF2 domain-containing protein  (*Loxodonta africana*) | GYEVPQQALDLQR | Deamidation (NQ),Iodination | 2 | 1642.65 | -1.34 | 545000 | 47 |
| G3TXT4 | Diacylglycerol kinase, DAG kinase, EC 2.7.1.107  (*Loxodonta africana*) | IIKMMELENQK | Oxidation (M),Deamidation (NQ),di-oxidation (M) | 2 | 1424.69 | 0.87 | 115380 | 58 |
| G3TY18 | TTF-type domain-containing protein  (*Loxodonta africana*) | VDGGGAPEVSLSAR | Unmodified | 2 | 1313.66 | 1.30 | 349270 | 49 |
| G3TY24 | DNA helicase, EC 3.6.4.12  (*Loxodonta africana*) | THEWMHPQTKR | Acetyl (K),Oxidation (M) | 2 | 1507.70 | -0.74 | 290780 | 62 |
| G3TYF8 | Inositol polyphosphate-5-phosphatase D  (*Loxodonta africana*) | LDQLIEFYK | Oxidation Y | 2 | 1183.61 | -3.65 | 115660000 | 104 |
| G3TYJ6 | Kinesin-like protein  (*Loxodonta africana*) | DMAEMQRVWKEK | Oxidation (M) | 2 | 1565.73 | -1.04 | 2147700 | 72 |
| G3TYV6 | NFKB activating protein  (*Loxodonta africana*) | SLSRSPKPTR | 2 Oxidation (P) | 2 | 1159.63 | -0.45 | 3826500 | 80 |
| G3TYY5 | DNA helicase, EC 3.6.4.12  (*Loxodonta africana*) | IEEIEREIIK | Unmodified | 2 | 1270.71 | 0.25 | 1761900 | 112 |
| G3TYY8 | 3-phosphoinositide-dependent protein kinase 1, EC 2.7.11.1  (*Loxodonta africana*) | IQEVWRQRYQNQPDAAVQ | Deamidation (NQ),O-2H (Y) | 2 | 2243.07 | 0.73 | 1132200 | 47 |
| G3TZ22 | G_PROTEIN_RECEP_F1_2 domain-containing protein  (*Loxodonta africana*) | MLVNIQSRSK | Deamidation (NQ),di-oxidation (M) | 2 | 1207.62 | -3.05 | 215990 | 70 |
| G3TZI4 | Uncharacterized protein  (*Loxodonta africana*) | QQNQVLWKCK | Oxidation (W) | 2 | 1346.68 | -0.08 | 308340 | 52 |
| G3U0A3 | 40S ribosomal protein S27a (Ubiquitin carboxyl extension protein 80) (Ubiquitin-40S ribosomal protein S27a)  (*Loxodonta africana*) | QVEPSDTIENVK | Deamidation (NQ) | 2 | 1358.66 | 0.33 | 469570 | 85 |
| G3U0D3 | StAR related lipid transfer domain containing 13  (*Loxodonta africana*)  (*Loxodonta africana*) | LTATMEKYSMSNK | Acetyl (K),Oxidation (M),Deamidation (NQ) | 2 | 1561.70 | 0.89 | 257190 | 46 |
| G3U0E8 | DNA polymerase, EC 2.7.7.7  (*Loxodonta africana*) | VTGVPLSYLLTR | Iodination | 2 | 1443.66 | -1.25 | 58354 | 45 |
| G3U0V6 | Kruppel like factor 4  (*Loxodonta africana*) | GLVARAGEPSVRR | Oxidation (P) | 3 | 1382.77 | 2.81 | 3629200 | 54 |
| G3U0Y5 | Peptidase M12B domain-containing protein  (*Loxodonta africana*) | ASGTGYFYVLAPK | 2 dioxidation Y | 2 | 1436.68 | 4.37 | 308330 | 44 |
| G3U108 | Adenosine deaminase, EC 3.5.4.4  (*Loxodonta africana*) | MARLGGQLVLK | Oxidation (M),Deamidation (NQ) | 2 | 1201.69 | -3.71 | 1316900 | 46 |
| G3U1F5 | Peptidase S1 domain-containing protein  (*Loxodonta africana*) | KAQLNTAVQTIALPR | 2 Deamidation (NQ) | 2 | 1624.91 | 1.67 | 361110 | 54 |
| G3U1K5 | Phosphatidylinositol transfer protein membrane associated 1  (*Loxodonta africana*) | DVAVYAALGLPQLRSYIVGRAVR | dioxidation Y | 3 | 2518.40 | 0.64 | 2964700 | 46 |
| G3U1Y8 | Glycylpeptide N-tetradecanoyltransferase, EC 2.3.1.97  (*Loxodonta africana*) | MKGFDVFNALDLMENK | Acetyl (K),Oxidation (M) | 3 | 1928.90 | 2.97 | 1483300 | 45 |
| G3U1Z4 | Peptidase A1 domain-containing protein  (*Loxodonta africana*) | QPGITFIAAK | Gln->pyro-Glu | 2 | 1027.57 | 1.48 | 454290 | 71 |
| G3U233 | Nuclear receptor binding SET domain protein 3  (*Loxodonta africana*) | EYHVQFFSNQPER | Glu->pyro-Glu,Iodination | 2 | 1787.66 | 3.47 | 2393300 | 61 |
| G3U2J7 | AP-5 complex subunit beta-1 (Adaptor-related protein complex 5 beta subunit)  (*Loxodonta africana*) | PLLLPLQPR | Deamidation (NQ),3 Oxidation (P) | 2 | 1094.63 | -0.69 | 197070 | 61 |
| G3U316 | Uncharacterized protein  (*Loxodonta africana*) | SPLKRSWVGR | O-2H (W) | 2 | 1198.66 | -0.29 | 96963 | 71 |
| G3U336 | Proline rich coiled-coil 1  (*Loxodonta africana*) | ALAGMYKQRLPPR | Deamidation (NQ),Oxidation (P) | 2 | 1516.82 | -3.78 | 439440 | 57 |
| G3U3K6 | Tyrosine-protein kinase, EC 2.7.10.2  (*Loxodonta africana*) | SDSIIFQFIKCCPPK | Acetyl (K),Trioxidation (C) | 2 | 1928.90 | -2.99 | 496350 | 61 |
| G3U4D5 | Catalase, EC 1.11.1.6  (*Loxodonta africana*) | NLSVEDAAR | Unmodified | 2 | 973.48 | -0.25 | 543330 | 119 |
| G3U4M8 | Gametogenetin-binding protein 2 (Protein ZNF403)  (*Loxodonta africana*) | AEPEFAGGYERR | Iodination | 2 | 1506.54 | -0.83 | 153580 | 80 |
| G3U4V4 | UDP-glucose glycoprotein glucosyltransferase 1  (*Loxodonta africana*) | QLLYDAIKHQK | Deamidation (NQ),dioxidation Y | 2 | 1388.73 | -3.07 | 98048 | 59 |
| G3U4V8 | Uncharacterized protein  (*Loxodonta africana*) | QELCKLMCER | Deamidation (NQ),Trioxidation (C) | 2 | 1414.59 | 1.31 | 6018000 | 66 |
| G3U552 | Bridging integrator 1  (*Loxodonta africana*) | HHYESLQTAKK | O-2H (Y) | 2 | 1354.66 | 1.09 | 638220 | 63 |
| G3U560 | Dynein axonemal heavy chain 5  (*Loxodonta africana*) | KEVVQCMGSFQDGVAEK | Deamidation (NQ) | 3 | 1911.87 | 3.76 | 596230 | 93 |
| G3U5B6 | DM1 locus, WD repeat containing  (*Loxodonta africana*) | SIDLNKPIDKR | Deamidation (NQ),Oxidation (P) | 2 | 1314.71 | -0.33 | 1187400 | 93 |
| G3U5S4 | Calcium binding and coiled-coil domain 2  (*Loxodonta africana*) | LEQTMKEMK | Acetyl (K),Deamidation (NQ),di-oxidation (M) | 2 | 1211.54 | -0.17 | 2252600 | 88 |
| G3U622 | Uncharacterized protein  (*Loxodonta africana*) | VNLDVVMKR | Acetyl (K),Deamidation (NQ) | 2 | 1115.60 | -4.45 | 1269800 | 96 |
| G3U630 | Uncharacterized protein  (*Loxodonta africana*) | TLKDIQNDFMEIMIK | Oxidation (M),Deamidation (NQ) | 3 | 1854.91 | -2.31 | 195850 | 59 |
| G3U6B1 | G_PROTEIN_RECEP_F1_2 domain-containing protein  (*Loxodonta africana*) | GLQSQAPPVINDQHPVQPDAL | Deamidation (NQ) | 3 | 2224.11 | -0.11 | 7348300 | 54 |
| G3U6M5 | Glutathione transferase, EC 2.5.1.18  (*Loxodonta africana*) | PQYLEELPDKMR | Oxidation (M),Oxidation Y | 2 | 1549.74 | 2.09 | 225520 | 43 |
| G3U6Q3 | Coiled-coil domain containing 88A  (*Loxodonta africana*) | IGNLEKENK | 2 Deamidation (NQ) | 2 | 1045.53 | -0.88 | 402290 | 63 |
| G3U778 | 1-phosphatidylinositol-3-phosphate 5-kinase, EC 2.7.1.150  (*Loxodonta africana*) | SQDSDLKQYWMPDSQCK | Deamidation (NQ),Di-oxidation W | 2 | 2147.88 | -0.68 | 597720 | 43 |
| G3U7G5 | Delta-like protein  (*Loxodonta africana*) | YPAVDYNLVQDLK | Iodination,Oxidation Y | 2 | 1678.67 | -2.01 | 275580 | 74 |
| G3U7X5 | Uncharacterized protein  (*Loxodonta africana*) | SYSVNQKTMSAKK | Oxidation (M) | 2 | 1486.74 | -2.69 | 21553000 | 57 |
| G3U7Y0 | Ig-like domain-containing protein  (*Loxodonta africana*) | NISFDEENWTLR | 2 Deamidation (NQ),Di-oxidation W | 2 | 1556.66 | -2.25 | 273350 | 53 |
| G3U7Z5 | THADA armadillo repeat containing  (*Loxodonta africana*) | DIGDYFKQHLLQSR | Deamidation (NQ),O-2H (Y) | 2 | 1733.84 | 1.45 | 488470 | 49 |
| G3U812 | Matrix remodeling associated 8  (*Loxodonta africana*) | RLVDMYSAGEQRVYEPR | O-2H (Y) | 3 | 2082.00 | 1.61 | 1254900 | 50 |
| G3U884 | Protein phosphatase 1 regulatory subunit 9A  (*Loxodonta africana*) | NQLQQNIEENK | Unmodified | 2 | 1356.66 | 1.79 | 384140 | 84 |
| G3U8H0 | Tyrosine-protein kinase receptor, EC 2.7.10.1  (*Loxodonta africana*) | NLFERNPNKELK | 2 Deamidation (NQ) | 2 | 1502.77 | -1.21 | 7975300 | 74 |
| G3U8L9 | Non-specific serine/threonine protein kinase, EC 2.7.11.1  (*Loxodonta africana*) | RAQMWTWFK | Trp->Kynurenine | 2 | 1256.61 | -0.97 | 285640 | 69 |
| G3U8N4 | Phosphatidylinositol-4-phosphate 5-kinase type 1 gamma  (*Loxodonta africana*) | QAPSMTGQQGPGHGKK | 2 Deamidation (NQ),Oxidation (P) | 2 | 1625.75 | -1.74 | 179270 | 63 |
| G3U8T2 | Matrix metallopeptidase 24  (*Loxodonta africana*) | NKAGPQPVTYYKR | Deamidation (NQ),2 dioxidation Y | 2 | 1585.77 | -4.35 | 507110 | 69 |
| G3U8X7 | Carnosine dipeptidase 2  (*Loxodonta africana*) | FKVYMSHGGK | Acetyl (K) | 2 | 1194.59 | 1.83 | 1678200 | 91 |
| G3U907 | F-box protein 46  (*Loxodonta africana*) | RVWDGIAAKINGITSWK | Di-oxidation W | 2 | 1946.04 | 1.08 | 5151800 | 66 |
| G3U942 | Protein kinase C and casein kinase substrate in neurons 2  (*Loxodonta africana*) | ADPSLNPEQLK | Deamidation (NQ) | 2 | 1211.60 | -0.64 | 1619700 | 72 |
| G3U952 | Ubiquitinyl hydrolase 1, EC 3.4.19.12  (*Loxodonta africana*) | SPRGWETGGDSAGADEARR | O-2H (W) | 3 | 1987.87 | 3.36 | 1777300 | 56 |
| G3U9R8 | KIAA1549 like  (*Loxodonta africana*) | NSGYDINGIRK | Deamidation (NQ),dioxidation Y | 2 | 1268.60 | -0.06 | 633060 | 64 |
| G3U9U3 | GRB10 interacting GYF protein 1  (*Loxodonta africana*) | EEMLALYIK | Oxidation (M),O-2H (Y) | 2 | 1138.56 | -4.13 | 457140 | 82 |
| G3U9Z9 | Asparaginase, EC 3.5.1.1  (*Loxodonta africana*) | PSLQGSPLGR | 2 Oxidation (P) | 2 | 1042.54 | 0.73 | 5376900 | 151 |
| G3UA11 | Tubby-like protein  (*Loxodonta africana*) | QEPLMVQANADGR | 2 Deamidation (NQ),di-oxidation (M) | 2 | 1461.64 | 2.88 | 6571600 | 50 |
| G3UA59 | F-box protein 39  (*Loxodonta africana*) | FLNPYNAVLTKK | dioxidation Y | 2 | 1438.78 | -3.80 | 240040 | 48 |
| G3UA69 | Uncharacterized protein  (*Loxodonta africana*) | QRWGQYFR | Gln->pyro-Glu | 2 | 1122.54 | -3.48 | 2602800 | 72 |
| G3UA87 | Ubiquitin carboxyl-terminal hydrolase, EC 3.4.19.12  (*Loxodonta africana*) | CTESEEEEVTKGK | Acetyl (K),Trioxidation (C) | 2 | 1614.66 | -3.70 | 9049700 | 52 |
| G3UAA1 | Dystonin  (*Loxodonta africana*) | CENFTTCWR | Deamidation (NQ),Di-oxidation W | 2 | 1305.48 | -1.54 | 254030 | 55 |
| G3UAG9 | Ubiquitination factor E4B  (*Loxodonta africana*) | KAQMQTDDR | Deamidation (NQ) | 2 | 1092.49 | 1.71 | 437970 | 56 |
| G3UAJ1 | RRM domain-containing protein  (*Loxodonta africana*) | LDFLQNPKQRDK | 2 Deamidation (NQ) | 2 | 1502.77 | 1.26 | 3882300 | 70 |
| G3UAT3 | Desmoglein 2  (*Loxodonta africana*) | NVQEGIYFK | Deamidation (NQ),dioxidation Y | 2 | 1129.53 | -3.17 | 678590 | 67 |
| G3UB70 | Coiled-coil domain-containing protein 128 (KLRAQ motif-containing protein 1) (Protein phosphatase 1 regulatory subunit 21)  (*Loxodonta africana*) | YYYNALNVPLHNR | 2 dioxidation Y | 2 | 1699.80 | 3.23 | 531220 | 50 |
| G3UBH8 | Glycerol-3-phosphate dehydrogenase, EC 1.1.5.3  (*Loxodonta africana*) | ALEHFPMLQK | Oxidation (M),Deamidation (NQ) | 2 | 1229.61 | 1.48 | 135410 | 46 |
| G3UBV9 | VPS35 endosomal protein-sorting factor-like  (*Loxodonta africana*) | CIMEAFINALTLEDEK | Deamidation (NQ),di-oxidation (M) | 2 | 1928.87 | 0.27 | 284390 | 55 |
| G3UC58 | Kinesin family member 13°  (*Loxodonta africana*) | MPVEHNSKPEKK | Unmodified | 3 | 1422.73 | -2.57 | 192660 | 50 |
| G3UC90 | Uncharacterized protein  (*Loxodonta africana*) | TFKQCSNLIK | Deamidation (NQ),Trioxidation (C) | 2 | 1286.62 | -3.75 | 497910 | 78 |
| G3UC99 | Solute carrier family 25 member 39  (*Loxodonta africana*) | ELGTCVQAAVAQGGWR | 2 Deamidation (NQ),O-2H (W) | 2 | 1717.77 | 2.26 | 475680 | 45 |
| G3UCH9 | 4-alpha-glucanotransferase, EC 2.4.1.25, EC 3.2.1.33 (Amylo-alpha-1,6-glucosidase) (Dextrin 6-alpha-D-glucosidase) (Glycogen debrancher)  (Glycogen debranching enzyme) (Oligo-1,4-1,4-glucantransferase)  (*Loxodonta africana*) | VGILHSCQK | Trioxidation (C) | 2 | 1088.53 | -4.14 | 97488 | 61 |
| G3UCN4 | CD101 molecule  (*Loxodonta africana*) | DDSFSYARYAQRVR | Oxidation Y,O-2H (Y) | 2 | 1762.80 | 0.01 | 1491100 | 49 |
| G3UCQ0 | ATG11 domain-containing protein  (*Loxodonta africana*) | MIQQEERYEAIIHK | 2 Deamidation (NQ),di-oxidation (M) | 2 | 1820.86 | -3.12 | 494860 | 48 |
| G3UCT3 | Exonuclease 5  (*Loxodonta africana*) | SISLQKWKR | Deamidation (NQ),O-2H (W) | 2 | 1159.63 | -1.98 | 4699900 | 103 |
| G3UCY3 | Tetratricopeptide repeat domain 6  (*Loxodonta africana*) | ISFSYNLQAQGKFQK | Oxidation Y | 2 | 1773.90 | -0.60 | 184780 | 84 |
| G3UDA1 | Ciliary rootlet coiled-coil, rootletin  (*Loxodonta africana*) | VEDLLAQSR | Deamidation (NQ) | 2 | 1030.53 | 0.62 | 555130 | 119 |
| G3UDD9 | Deubiquitinating enzyme A, EC 3.4.19.12  (*Loxodonta africana*) | ESYLQWLRDQEK | Glu->pyro-Glu,Deamidation (NQ) | 2 | 1576.75 | -3.36 | 346750 | 60 |
| G3UDE1 | NCCRP1, F-box associated domain containing  (*Loxodonta africana*) | VTDSSVSVQLRE | Unmodified | 2 | 1318.67 | 0.03 | 1093000 | 119 |
| G3UDI3 | Phosphatidylinositol-4-phosphate 3-kinase, EC  (*Loxodonta africana*)2.7.1.154 | VGGEVKLSISYK | dioxidation Y | 2 | 1310.71 | 1.69 | 786860 | 55 |
| G3UDP7 | Rac/Cdc42 guanine nucleotide exchange factor 6  (*Loxodonta africana*) | TLMGQCQDLRKR | 2 Deamidation (NQ) | 2 | 1506.73 | 1.31 | 16328000 | 65 |
| G3UDQ1 | Transmembrane 9 superfamily member  (*Loxodonta africana*) | WKKSDVK | Trp->Kynurenine | 2 | 893.50 | -0.02 | 2284200 | 46 |
| G3UDS0 | DNA helicase, EC 3.6.4.12  (*Loxodonta africana*) | WAGLSYWHCSWVK | Unmodified | 2 | 1678.77 | -3.44 | 107710 | 69 |
| G3UDW0 | VPS39 subunit of HOPS complex  (*Loxodonta africana*) | IQQIHVVSQFK | 2 Deamidation (NQ) | 2 | 1327.71 | -2.14 | 1036400 | 81 |
| G3UDZ8 | Catenin delta 1  (*Loxodonta africana*) | NLAVDPRNK | 2 Deamidation (NQ) | 2 | 1027.53 | 1.53 | 220700 | 66 |
| G3UEC2 | ATPase family AAA domain containing 2B  (*Loxodonta africana*) | QNLLSLQKYCR | Deamidation (NQ),Oxidation Y | 2 | 1438.72 | -0.26 | 3170200 | 49 |
| G3UEI7 | Carboxymethylenebutenolidase homolog  (*Loxodonta africana*) | TRNAQKVDK | Acetyl (K),2 Deamidation (NQ) | 2 | 1102.56 | -0.29 | 3646900 | 42 |
| G3UEU2 | Intersectin 1  (*Loxodonta africana*) | EVAMIFVNWKELIMCNIK | Deamidation (NQ) | 3 | 2238.13 | 0.68 | 6439700 | 54 |
| G3UEU8 | Vascular endothelial zinc finger 1  (*Loxodonta africana*) | PKTSFVCTYCSK | Acetyl (K),2 Trioxidation (C) | 2 | 1614.65 | -2.42 | 10154000 | 60 |
| G3UF20 | NCK associated protein 5  (*Loxodonta africana*) | TVTQNNIQRQK | Deamidation (NQ) | 2 | 1329.70 | 3.14 | 6651500 | 102 |
| G3UFA9 | Uncharacterized protein  (*Loxodonta africana*) | SLCQAGPEAAPGLNAAR | Deamidation (NQ) | 3 | 1682.80 | 3.93 | 1515800 | 72 |
| G3UFM4 | Uncharacterized protein  (*Loxodonta africana*) | GDKDMVVKDGR | Acetyl (K),di-oxidation (M) | 2 | 1292.60 | -0.92 | 66063 | 43 |
| G3UFS6 | Uncharacterized protein  (*Loxodonta africana*) | GVHSQQPPTFWQR | 2 Deamidation (NQ),2 Oxidation (P) | 2 | 1600.73 | -2.48 | 1500800 | 70 |
| G3UG67 | Uncharacterized protein  (*Loxodonta africana*) | ASLLQAIEECQQER | Deamidation (NQ),Trioxidation (C) | 2 | 1722.77 | -2.35 | 831100 | 64 |
| G3UGN6 | Regulator of chromosome condensation 2  (*Loxodonta africana*) | IKKLPEYNPR | O-2H (Y) | 2 | 1270.70 | -3.29 | 1347700 | 90 |
| G3UGN8 | Uncharacterized protein  (*Loxodonta africana*) | ENQVVKACNGR | Glu->pyro-Glu,Deamidation (NQ) | 2 | 1256.59 | -2.73 | 782140 | 71 |
| G3UGW4 | Phosphofructokinase, liver type  (*Loxodonta africana*) | PQNLPRSPLGR | 2 Deamidation (NQ),2 Oxidation (P) | 2 | 1267.65 | -0.05 | 532510 | 56 |
| G3UH60 | Suppression of tumorigenicity 7  (*Loxodonta africana*) | PADIVMQKAWR | Oxidation (M),Oxidation (P) | 2 | 1345.68 | 2.53 | 927030 | 53 |
| G3UHD3 | Uncharacterized protein  (*Loxodonta africana*) | AVAKEPDAMNR | Acetyl (K),Deamidation (NQ),di-oxidation (M) | 2 | 1275.58 | 1.97 | 466610 | 46 |
| G3UHK8 | Spectrin beta chain  (*Loxodonta africana*) | LTGMERDLVAIEAKLSDLQK | Unmodified | 3 | 2229.20 | 1.13 | 1251800 | 49 |
| G3UIB5 | NOL1/NOP2/Sun domain family member 2, EC 2.1.1.203 (mRNA cytosine C(5)-methyltransferase) (tRNA cytosine C(5)-methyltransferase)  (*Loxodonta africana*) | NVLLNNSER | 3 Deamidation (NQ) | 2 | 1060.50 | 1.13 | 5000000 | 55 |
| G3UIJ2 | Epididymal sperm binding protein 1  (*Loxodonta africana*) | PDMPLYVLRNLDAK | Deamidation (NQ),Oxidation Y | 3 | 1660.85 | 1.61 | 446730 | 56 |
| G3UIR3 | Sperm flagellar 2  (*Loxodonta africana*) | GKTQGGKQPVK | 2 Acetyl (K),Deamidation (NQ) | 2 | 1211.65 | -0.03 | 1198800 | 54 |
| G3UIV5 | Protein-tyrosine-phosphatase, EC 3.1.3.48  (*Loxodonta africana*) | YQYWVVGEVCSVYMTQKCTR | Deamidation (NQ),Oxidation (W) | 3 | 2573.14 | -2.30 | 436030 | 51 |
| G3UIX4 | Helicase with zinc finger  (*Loxodonta africana*) | PAMSYASALR | Oxidation (M),dioxidation Y | 2 | 1113.51 | -2.62 | 393780 | 57 |
| G3UJE9 | Zinc finger and BTB domain containing 47  (*Loxodonta africana*) | NIQCVTCGK | Deamidation (NQ),Trioxidation (C) | 2 | 1127.46 | 3.71 | 250550 | 56 |
| G3UJF3 | Uncharacterized protein  (*Loxodonta africana*) | LSMEQICK | Oxidation (M),Deamidation (NQ) | 2 | 1024.46 | 3.39 | 1365500 | 65 |
| G3UJN9 | Spectrin repeat containing nuclear envelope protein 1  (*Loxodonta africana*) | LSLDQALVK | Deamidation (NQ) | 2 | 986.56 | 0.34 | 1257300 | 109 |
| G3UJQ9 | Phospholipid-transporting ATPase, EC 7.6.2.1  (*Loxodonta africana*) | ANDREYNEK | Deamidation (NQ),O-2H (Y) | 2 | 1152.47 | 3.77 | 1580300 | 99 |
| G3UJZ4 | 5-hydroxytryptamine receptor 1F (Serotonin receptor 1F)  (*Loxodonta africana*) | EEMNGQVLLESGEKSTR | 2 Deamidation (NQ),di-oxidation (M) | 2 | 1939.87 | 3.13 | 132920 | 52 |
| G3UKF9 | RPGR interacting protein 1  (*Loxodonta africana*) | VIDLDPLEQKGR | Unmodified | 3 | 1381.76 | -0.19 | 10403000 | 67 |
| G3UKI5 | VLIG-type G domain-containing protein  (*Loxodonta africana*) | QYFLRWMELGLAR | Iodination | 2 | 1807.77 | 2.20 | 327830 | 66 |
| G3UKS7 | Corneodesmosin  (*Loxodonta africana*) | GSPGVPSFAAGPPISEGK | Unmodified | 2 | 1653.84 | -0.10 | 1498400 | 97 |
| G3UKT4 | Polybromo 1  (*Loxodonta africana*) | YNESDKQMK | O-2H (Y) | 2 | 1155.49 | 3.10 | 732760 | 54 |
| G3UL53 | UBC core domain-containing protein  (*Loxodonta africana*) | DQLMFDLLK | Oxidation (M),Deamidation (NQ) | 2 | 1138.56 | -2.99 | 485890 | 96 |
| G3ULC2 | Cyclin O  (*Loxodonta africana*) | DYGQSCYDFHK | 2 Oxidation Y | 2 | 1450.55 | -3.35 | 54446 | 49 |
| G3ULV9 | Forkhead box O3  (*Loxodonta africana*) | PPPQPGAAGGSGQPR | Deamidation (NQ),3 Oxidation (P) | 2 | 1421.65 | 0.57 | 766780 | 72 |
| G3UM67 | SH3 and PX domains 2A  (*Loxodonta africana*) | TVSKLAQGSPAVAR | Acetyl (K),Deamidation (NQ) | 2 | 1426.78 | 1.12 | 5374000 | 100 |
| G3UME3 | Uncharacterized protein  (*Loxodonta africana*) | PDSGCQGRGIFITR | Deamidation (NQ) | 2 | 1563.75 | 2.29 | 1111300 | 82 |
| G3UMP4 | Uncharacterized protein  (*Loxodonta africana*) | AFATTQQVIQQVYSR | 2 Deamidation (NQ),O-2H (Y) | 2 | 1754.85 | 1.19 | 835350 | 55 |
| G3UMV7 | Myopalladin  (*Loxodonta africana*) | EFPFNMSVLNSTASPAVTLSSK | Oxidation (M) | 3 | 2342.15 | -1.25 | 293390 | 50 |
| G3UN69 | Uncharacterized protein  (*Loxodonta africana*) | KISKTSTFYHENFK | Acetyl (K),Deamidation (NQ) | 2 | 1771.88 | 1.28 | 237530 | 63 |
| G3UNE5 | Ferritin  (*Loxodonta africana*) | ELGDYITNLHK | O-2H (Y) | 2 | 1315.64 | -3.37 | 665440 | 83 |
| G3UNF6 | Cholesteryl ester transfer protein  (*Loxodonta africana*) | MPKISCRHR | Unmodified | 2 | 1183.61 | 0.30 | 142840000 | 92 |
| G3UNM2 | Uncharacterized protein  (*Loxodonta africana*) | TVLFGVQPKFTNVDIR | Oxidation (P) | 3 | 1849.01 | 3.36 | 286690 | 47 |
| G3X8I2 | WD repeat domain 78  (*Loxodonta africana*) | VLMENVFQSK | 2 Deamidation (NQ),di-oxidation (M) | 2 | 1227.57 | 2.25 | 676380 | 59 |
| G5E6V4 | Centlein  (*Loxodonta africana*) | LYNELHICFETTK | Deamidation (NQ),Iodination | 2 | 1793.68 | 4.14 | 276630 | 42 |
| G5E6Y9 | MutS homolog 3  (*Loxodonta africana*) | ALENDGPVKKR | Deamidation (NQ) | 2 | 1226.66 | 0.21 | 2609800 | 140 |
| G5E707 | DNA topoisomerase 2, EC 5.6.2.2  (*Loxodonta africana*) | QTWMNNMMK | 2 di-oxidation (M) | 2 | 1246.48 | -2.87 | 1169600 | 56 |
| G5E747 | Proline rich coiled-coil 2B  (*Loxodonta africana*) | PEEANGPSLTEPK | Deamidation (NQ),Oxidation (P) | 2 | 1384.64 | -2.48 | 162020 | 54 |
| G5E777 | Protein phosphatase 1 regulatory subunit 18  (*Loxodonta africana*) | PDCGESQEQSSVQLEASQWR | Deamidation (NQ),Oxidation (W) | 3 | 2336.98 | -1.29 | 9092400 | 47 |
| P0C2W8 | Collagen alpha-1(I) chain (Alpha-1 type I collagen)  *(Mammut americanum)* | GSAGPPGATGFPGAAGR | 2 Oxidation (P) | 2 | 1458.69 | -0.17 | 443940 | 94 |
| Q0QES2 | IDP, EC 1.1.1.42 (NADP(+)-specific ICDH) (Oxalosuccinate decarboxylase)  (*Loxodonta africana*) | LDNNNELSFFAK | Unmodified | 2 | 1410.68 | 1.65 | 2354700 | 96 |

Table S3 List of peptides and proteins of Viridiplantae in trunk sample: for each peptide all the features (same sequence and different modifications) with the highest intensity are reported.

| Acc- Number | Description | Specie | Sequence | Modifications | Charge | Mass | Mass error [ppm] | Intensity | Score |
| --- | --- | --- | --- | --- | --- | --- | --- | --- | --- |
| A7Y3B9 | DNA-directed RNA polymerase subunit beta | Ipomoea purpurea | RSNKNTCMHQK | 2 Deam (NQ) | 2 | 1404 .62 | 3 .50 | 7240200 | 67 |
|  |  |  | NTCMHQKPQVQRGK | 2 Deam (NQ) | 3 | 1712 .81 | 2 .28 | 1695200 | 48 |
| O22267 | Histidine kinase CKI1 | Arabidopsis thaliana | ATLINQMEATQQAER | Ox (M),2 Deam (NQ) | 2 | 1720 .79 | 3 .65 | 1705500 | 46 |
|  |  |  | REMHMRATLINQMEATQQAER | 2 Ox (M) | 3 | 2575 .21 | -3 .25 | 485320 | 45 |
| O65316 | Actin | Mesostigma viride | AVFPSIVGR | Umod | 2 | 944 .54 | 1 .11 | 634230 | 117 |
|  |  |  | SYELPDGQVITIGNER | Umod | 2 | 1789 .88 | 0 .83 | 1190500 | 88 |
| P0DH99 | Elongation factor 1-alpha 1 | Arabidopsis thaliana | IGGIGTVPVGR | Umod | 2 | 1024 .60 | 0 .76 | 3483000 | 158 |
|  |  |  | LPLQDVYK | Umod | 2 | 974 .54 | 1 .34 | 1276100 | 108 |
| P11143 | Heat shock 70 kDa protein | Zea mays | IINEPTAAAIAYGLDK | Umod | 2 | 1658 .89 | -0 .47 | 1161900 | 91 |
|  |  |  | TTPSYVAFTDTER | Umod | 2 | 1486 .69 | 1 .29 | 1949200 | 101 |
| P29685 | ATP synthase subunit beta | Hevea brasiliensis | IGLFGGAGVGK | Umod | 2 | 974 .55 | 0 .28 | 353820 | 93 |
|  |  |  | TIAMDGTEGLVR | Ox (M) | 2 | 1277 .63 | -1 .23 | 1180000 | 118 |
| P93206 | 14-3-3 protein 1 | Solanum lycopersicum | DSTLIMQLLR | Ox (M) | 2 | 1204 .65 | -0 .95 | 508570 | 107 |
|  |  |  | NDEHVVLVKDYR | Deam (NQ),diOx Y | 2 | 1518 .73 | -0 .38 | 6459500 | 80 |
| Q9FNA4 | Elongator complex protein 1 | Arabidopsis thaliana | EMELLNSSDDIRK | Ox (M),Glu->pyro-Glu | 2 | 1546 .73 | -2 .08 | 11241000 | 54 |
|  |  |  | EMELLNSSDDIRK | Glu->pyro-Glu | 2 | 1530 .73 | -0 .71 | 4399700 | 63 |
| A0A078CGE6 | MAP3K epsilon protein kinase 1 | Brassica napus | ESSIQMQQR | Glu->pyro-Glu,Deam (NQ),di-Ox (M) | 2 | 1120 .48 | 0 .51 | 1613100 | 57 |
| A0A0P0WFC8 | Transcription factor TGAL6 | Oryza sativa subsp. japonica | LAQLEQELQR | Umod | 2 | 1226 .66 | -0 .53 | 2121700 | 105 |
| A0A2P1GIW4 | Protein REDOX 1 | Catharanthus roseus | ATGEEDVRLKVLYCGVCHSDLHNIK | TriOx (C) | 3 | 2960 .41 | -4 .43 | 1815300 | 46 |
| A0A2U1KZS6 | NADPH--cytochrome P450 reductase 2 | Artemisia annua | EYVQHKLTQK | Glu->pyro-Glu,O-2H (Y) | 2 | 1268 .65 | 0 .88 | 2114900 | 48 |
| A1A6M1 | Protein disulfide isomerase pTAC5, ... | Arabidopsis thaliana | EEQRWIREEQR | Deam (NQ),O-2H (W) | 2 | 1572 .73 | 2 .07 | 4309500 | 60 |
| A1XGU4 | NAD(P)H-quinone oxidoreductase subu... | Ranunculus macranthus | QIDDNGISNMMR | Ox (M),2 Deam (NQ) | 3 | 1410 .58 | -0 .88 | 1063800 | 42 |
| A2RVU1 | Protein MODIFYING WALL LIGNIN-1 | Arabidopsis thaliana | PSLQVENHDKR | Deam (NQ) | 2 | 1322 .66 | -0 .46 | 6877400 | 79 |
| A2X254 | SPX domain-containing protein 2 | Oryza sativa subsp. indica | EEEYVIKQK | Acetyl (K),Glu->pyro-Glu,Deam (NQ) | 2 | 1189 .59 | -0 .04 | 3685400 | 53 |
| A2XN66 | Probable cellulose synthase A catal... | Oryza sativa subsp. indica | ERVDGWKMK | Ox (M),Ox (W) | 2 | 1179 .57 | 3 .63 | 588700 | 40 |
| A3BDI8 | Zinc finger A20 and AN1 domain-cont... | Oryza sativa subsp. japonica | CHKEMIMKQEQAK | Acetyl (K),Ox (M),TriOx (C) | 2 | 1765 .78 | -3 .56 | 4546700 | 51 |
| A4QJI9 | DNA-directed RNA polymerase subunit... | Aethionema grandiflorum | KTKNLCLFEEDMR | Deam (NQ),di-Ox (M) | 2 | 1715 .79 | 1 .07 | 893560 | 53 |
| A4QKG5 | Protein TIC 214 | Barbarea verna | IKKLPQFQIITESNR | 3 Deam (NQ) | 3 | 1816 .99 | -2 .03 | 903920 | 42 |
| A4QLQ5 | Protein TIC 214 | Lobularia maritima | ENLTNSCNKPR | Glu->pyro-Glu,2 Deam (NQ) | 2 | 1315 .58 | -2 .53 | 3206000 | 59 |
| A6MMM8 | 50S ribosomal protein L33, chloropl... | Dioscorea elephantipes | IILECASCVR | 2 TriOx (C) | 2 | 1315 .57 | 2 .87 | 3206000 | 94 |
| A7KTC5 | Low affinity inorganic phosphate tr... | Petunia hybrida | YTALVAKNLKQATNDMSK | Ox (M),2 Deam (NQ) | 2 | 2013 .01 | -2 .23 | 5690100 | 67 |
| A7LFZ6 | Endoribonuclease Dicer homolog 4 | Oryza sativa subsp. japonica | LKNYGYKHR | Deam (NQ),2 diOx Y | 2 | 1242 .60 | -0 .43 | 922820 | 89 |
| A7M910 | Cytochrome f | Cuscuta gronovii | IGNLYFQSYR | 2 diOx Y | 2 | 1323 .61 | 0 .59 | 1786700 | 75 |
| A7Y3K5 | NAD(P)H-quinone oxidoreductase subu... | Ipomoea purpurea | LSNIQVPKR | Deam (NQ) | 2 | 1054 .61 | 0 .27 | 2773600 | 108 |
| A8I4E9 | Cilia- and flagella-associated prot... | Chlamydomonas reinhardtii | EIFLLQMSLDTK | Glu->pyro-Glu,Deam (NQ),di-Ox (M) | 2 | 1451 .72 | -2 .43 | 966190 | 42 |
| A8W3E4 | 50S ribosomal protein L20, plastid | Cuscuta exaltata | CLYMISNGILQI | Ox (M),TriOx (C) | 2 | 1487 .70 | 0 .42 | 294210 | 52 |
| A9SVH7 | Retinoblastoma-related protein | Physcomitrium patens | QSSLMSESVK | Deam (NQ),di-Ox (M) | 2 | 1127 .50 | 4 .45 | 827760 | 70 |
| B0F9L7 | Golgin candidate 2 | Arabidopsis thaliana | ESTLEELKR | Glu->pyro-Glu | 2 | 1085 .57 | 2 .96 | 3063600 | 53 |
| B2X1Z4 | DNA-directed RNA polymerase subunit... | Oedogonium cardiacum | EIVVNFNYLKK | Ox Y | 2 | 1381 .76 | -3 .08 | 1160800 | 80 |
| B2Y1X2 | Cytochrome f | Welwitschia mirabilis | IGNLNFQNYSPSQK | 3 Deam (NQ) | 2 | 1611 .74 | 4 .05 | 753580 | 47 |
| B3EWE9 | Antimicrobial protein 2 | Sesamum indicum | GLQDQQVYQR | 3 Deam (NQ) | 2 | 1236 .56 | -1 .44 | 739130 | 47 |
| B3TN96 | NAD(P)H-quinone oxidoreductase subu... | Brachypodium distachyon | SQNMALMGGLRK | Ox (M) | 2 | 1320 .66 | 3 .26 | 4084300 | 72 |
| B4FR29 | Rubisco accumulation factor 1, chlo... | Zea mays | GEEGWEAFSK | O-2H (W) | 2 | 1152 .47 | -0 .12 | 2932000 | 93 |
| B6SFA4 | Probable helicase MAGATAMA 3 | Arabidopsis thaliana | QLTKENER | Acetyl (K),2 Deam (NQ) | 2 | 1060 .50 | 1 .98 | 6636800 | 73 |
| B7F958 | UDP-glucose 6-dehydrogenase 2 | Oryza sativa subsp. japonica | PLDHWLRDMPAVA | Ox (M),O-2H (W) | 2 | 1549 .73 | 4 .17 | 881790 | 55 |
| B8AEH3 | DNA replication licensing factor MC... | Oryza sativa subsp. indica | YDDLKTAQDNIDLQTTILSR | 2 Deam (NQ) | 3 | 2324 .14 | 0 .79 | 586030 | 47 |
| B8BHK8 | Nijmegen breakage syndrome 1 protei... | Oryza sativa subsp. indica | ISPSLQAVMTSIGAYATR | Deam (NQ) | 2 | 1865 .96 | 3 .16 | 3519400 | 90 |
| B9DFG3 | DExH-box ATP-dependent RNA helicase... | Arabidopsis thaliana | ERVEFRWQR | Deam (NQ),Ox (W) | 2 | 1321 .65 | 1 .07 | 5528000 | 61 |
| B9EY52 | Kinesin-like protein KIN-13B | Oryza sativa subsp. japonica | LNENNVLLYAQCP | Deam (NQ),TriOx (C) | 2 | 1595 .71 | 2 .70 | 1222400 | 61 |
| B9G2A8 | Auxin transport protein BIG | Oryza sativa subsp. japonica | DETLSKNASMQRSAVENFVR | 2 Deam (NQ) | 2 | 2283 .08 | 4 .09 | 1235200 | 50 |
| C0LGD6 | Probable LRR receptor-like serine/t... | Arabidopsis thaliana | PITMTLENSDPNVR | Ox (M),2 Deam (NQ) | 2 | 1603 .74 | 4 .19 | 1117500 | 48 |
| C1K5M2 | Dimethylallylcistransferase CPT1, c... | Solanum lycopersicum | LGIQIITAFAFSTENWKR | Trp->Kynurenine | 3 | 2098 .12 | -2 .14 | 18050000 | 75 |
| C5XEK4 | CASP-like protein 4U1 | Sorghum bicolor | EGGGAPAPPK | Glu->pyro-Glu | 1 | 861 .43 | 0 .88 | 297610 | 63 |
| C7G304 | Galactinol synthase 2 | Solanum lycopersicum | NSNVVMNAVDGEVEAQK | Ox (M),2 Deam (NQ) | 2 | 1820 .81 | -1 .70 | 2144200 | 44 |
| C7J8E5 | Regulatory-associated protein of TO... | Oryza sativa subsp. japonica | IWRNYTQK | 2 Deam (NQ),O-2H (W) | 2 | 1123 .53 | -1 .87 | 937780 | 46 |
| D2YZP9 | (S)-beta-bisabolene synthase | Zingiber officinale | IQSQSYFKECQWR | 2 Deam (NQ),O-2H (Y) | 2 | 1774 .76 | -4 .06 | 1447000 | 46 |
| D7UPN3 | LysM domain receptor-like kinase 10 | Oryza sativa subsp. japonica | GIVFIPVKDPNGSYHPLK | Iodination | 3 | 2105 .98 | -1 .48 | 2749800 | 61 |
| E0ZS48 | Urease | Oryza sativa subsp. indica | LNMKLNDALPK | Acetyl (K),Ox (M),2 Deam (NQ) | 2 | 1315 .67 | -4 .41 | 502010 | 89 |
| F4HNU6 | Nardilysin-like | Arabidopsis thaliana | LYRLEAVK | Iodination | 2 | 1116 .48 | 3 .00 | 487700 | 43 |
| F4HTH8 | Katanin p80 WD40 repeat-containing ... | Arabidopsis thaliana | TSPAPVMPMK | 2 Ox (M) | 2 | 1089 .52 | 1 .01 | 983060 | 42 |
| F4HVZ5 | Protein COP1 SUPPRESSOR 2 | Arabidopsis thaliana | SRQAATDQIMLERFR | 2 Deam (NQ) | 2 | 1822 .90 | 1 .18 | 342550 | 41 |
| F4HZQ7 | WAT1-related protein At1g21890 | Arabidopsis thaliana | SPVKPVDTGKGLAAELEMK | Acetyl (K),di-Ox (M) | 3 | 2043 .06 | -3 .08 | 18873000 | 60 |
| F4I718 | Protein CELLULOSE SYNTHASE INTERACT... | Arabidopsis thaliana | EDAACILWNLCCHSEEIR | Glu->pyro-Glu,TriOx (C) | 2 | 2304 .96 | -0 .52 | 1308800 | 55 |
| F4IIK6 | Non-functional target of rapamycin ... | Arabidopsis thaliana | EEMVYQAHRK | Glu->pyro-Glu | 2 | 1271 .61 | 3 .22 | 1796300 | 40 |
| F4IVR7 | Myosin-10 | Arabidopsis thaliana | KIDESDSK | Acetyl (K) | 2 | 962 .46 | -0 .15 | 1849600 | 120 |
| F4J7T2 | Chromatin modification-related prot... | Arabidopsis thaliana | PGVPTISEPNTADNLLLFDSENK | Deam (NQ) | 3 | 2471 .21 | 2 .55 | 2789500 | 45 |
| F4JGP4 | Kinesin-like protein KIN-14D | Arabidopsis thaliana | ATITEMKENIESLQEKLSK | Acetyl (K),2 Deam (NQ),di-Ox (M) | 3 | 2267 .11 | 3 .71 | 4222000 | 60 |
| F4JLI5 | Xanthine dehydrogenase 2 | Arabidopsis thaliana | QAHRRDDDIAIVNGGMR | Ox (M),Deam (NQ) | 2 | 1939 .93 | -1 .30 | 32668000 | 47 |
| F4JMJ1 | Heat shock 70 kDa protein 17 | Arabidopsis thaliana | EIIKEWETNK | Umod | 2 | 1288 .67 | -2 .86 | 27334000 | 114 |
| F4JN05 | Brefeldin A-inhibited guanine nucle... | Arabidopsis thaliana | CTDPQQQEQQQRK | 2 Deam (NQ) | 2 | 1674 .73 | 0 .02 | 568600 | 47 |
| F4JRF4 | Adenine DNA glycosylase | Arabidopsis thaliana | RDLPWRNR | Deam (NQ),Trp->Kynurenine | 2 | 1116 .58 | 0 .01 | 8005300 | 83 |
| F4K0J3 | Kinesin-like protein KIN-4C | Arabidopsis thaliana | AVINRDPATAQMQRMR | Ox (M),Deam (NQ) | 2 | 1873 .93 | -3 .20 | 740420 | 51 |
| F4K128 | Probable ATP-dependent DNA helicase... | Arabidopsis thaliana | LSQLEGLPSNRGEDLQEK | Deam (NQ) | 3 | 2013 .00 | 0 .58 | 4640200 | 47 |
| F4K5T4 | Probable transcription factor At5g2... | Arabidopsis thaliana | LELVQEQIR | 2 Deam (NQ) | 2 | 1128 .60 | 0 .24 | 2844600 | 134 |
| F4KDH9 | FIP1[V]-like protein | Arabidopsis thaliana | SRPEKNEISYGQR | Deam (NQ),diOx Y | 2 | 1595 .75 | 0 .43 | 1497500 | 69 |
| F4KHQ8 | Neutral ceramidase 3 | Arabidopsis thaliana | NLLKNPTEEQVR | 2 Deam (NQ) | 2 | 1441 .74 | 0 .46 | 1053100 | 66 |
| F4KIA8 | Protein RRC1-like | Arabidopsis thaliana | CRHNGLSLLGGREMMVAR | Ox (M),Deam (NQ) | 3 | 2073 .00 | -2 .11 | 1030600 | 45 |
| F6M8H7 | Probable sesquiterpene synthase | Santalum murrayanum | ENQKMPISSVPNLKDLNMISR | Acetyl (K),Glu->pyro-Glu,2 Deam (NQ),di-Ox (M) | 3 | 2471 .20 | 4 .14 | 1800700 | 51 |
| F6M8I0 | Monoterpene synthase | Santalum album | EENVNPNLLK | Glu->pyro-Glu,2 Deam (NQ) | 2 | 1152 .57 | 1 .25 | 1517200 | 82 |
| H2DH18 | Cytochrome P450 CYP736A12 | Panax ginseng | DDRYDLKGIMNEALTLAGR | Acetyl (K),Ox (M),Deam (NQ) | 2 | 2209 .07 | -1 .69 | 9892000 | 43 |
| H2DH21 | Cytochrome P450 CYP72A219 | Panax ginseng | NSFLWLGPK | Deam (NQ),O-2H (W) | 2 | 1075 .53 | -2 .63 | 2585700 | 81 |
| H2E7U0 | Sterol methyltransferase-like 3 | Botryococcus braunii | EVYRVLKPGAYFALYDGVTK | Ox Y,O-2H (Y) | 3 | 2318 .19 | -3 .22 | 868340 | 52 |
| K7WCC7 | UDP-N-acetylmuramoyl-L-alanyl-D-glu... | Zea mays | GAVAVVADQDLNIEGTLACR | Deam (NQ),TriOx (C) | 2 | 2120 .01 | 3 .75 | 3379300 | 64 |
| O04294 | Importin subunit alpha-3 | Arabidopsis thaliana | EDNLVEIRK | Glu->pyro-Glu,Deam (NQ) | 2 | 1097 .57 | 2 .43 | 605160 | 51 |
| O04336 | Probable WRKY transcription factor ... | Arabidopsis thaliana | KLQSHVSQSLLLDPCQQR | 2 Deam (NQ) | 3 | 2138 .08 | 0 .42 | 2969700 | 53 |
| O04532 | Formin-like protein 8 | Arabidopsis thaliana | RSHSLTRSGSSNYNGGNSSLQVMSK | Ox (M),Deam (NQ),Ox Y | 3 | 2686 .24 | -0 .57 | 3704000 | 49 |
| O04705 | Gibberellin 20 oxidase 1-D | Triticum aestivum | SLLDFTQKHYR | Deam (NQ),diOx Y | 2 | 1439 .70 | -1 .74 | 2694000 | 86 |
| O04862 | Folate synthesis bifunctional prote... | Pisum sativum | VHNVKDNLDAVK | 2 Deam (NQ) | 3 | 1352 .69 | 0 .31 | 2733000 | 52 |
| O22842 | Endochitinase At2g43610 | Arabidopsis thaliana | GPIQITWNYNYGAAGK | 2 Ox Y | 2 | 1783 .85 | -1 .68 | 1859400 | 49 |
| O22873 | bZIP transcription factor 18 | Arabidopsis thaliana | GPYHRRAHSEVQFR | Deam (NQ),diOx Y | 2 | 1771 .85 | 0 .30 | 40395000 | 65 |
| O22918 | GDSL esterase/lipase At2g30220 | Arabidopsis thaliana | DSILYNQK | Deam (NQ),Ox Y | 1 | 996 .48 | -0 .61 | 1657600 | 50 |
| O23317 | Probable disease resistance protein... | Arabidopsis thaliana | IGIWKCGMK | Ox (M),Trp->Kynurenine | 2 | 1111 .55 | -0 .60 | 372580 | 48 |
| O23404 | Pyruvate, phosphate dikinase 1, chl... | Arabidopsis thaliana | ELVEQYKSVYLEAK | Deam (NQ),2 Di-iodination | 3 | 2202 .46 | 2 .05 | 10902000 | 41 |
| O23609 | Peroxidase 41 | Arabidopsis thaliana | GNLPLANQSVPDMLSIFKK | Acetyl (K),Deam (NQ) | 2 | 2114 .11 | 2 .71 | 344700 | 47 |
| O23880 | 13S globulin seed storage protein 2 | Fagopyrum esculentum | ETISKLR | Acetyl (K),Glu->pyro-Glu | 2 | 869 .50 | 0 .16 | 362480 | 65 |
| O24088 | CASP-like protein N24 | Medicago truncatula | VLEEKINMMDPK | Acetyl (K),Ox (M),Deam (NQ) | 2 | 1504 .72 | 4 .21 | 1321800 | 62 |
| O24308 | DNA topoisomerase 2 | Pisum sativum | LGNSATSWK | Deam (NQ),Ox (W) | 2 | 979 .46 | 0 .38 | 1094300 | 46 |
| O24475 | Pinene synthase, chloroplastic | Abies grandis | EVDFPSKLNDLACAILRLR | Umod | 3 | 2229 .19 | 3 .94 | 1590800 | 51 |
| O48533 | Protein POLYCHOME | Arabidopsis thaliana | RVRTLMSMR | Ox (M),di-Ox (M) | 2 | 1196 .61 | 2 .77 | 4633700 | 84 |
| O48573 | Disease resistance protein LAZ5 | Arabidopsis thaliana | NIAMVNLQDNLK | Ox (M),2 Deam (NQ) | 2 | 1389 .68 | -0 .20 | 1167900 | 56 |
| O48682 | Formin-like protein 4 | Arabidopsis thaliana | KLQQSQRDNK | 2 Deam (NQ) | 2 | 1245 .63 | 1 .63 | 1212400 | 65 |
| O49434 | Allantoate deiminase | Arabidopsis thaliana | MYQICDKR | Ox (M),O-2H (Y) | 2 | 1142 .48 | 4 .00 | 430740 | 43 |
| O49809 | Glyoxysomal fatty acid beta-oxidati... | Brassica napus | WALDIAERRK | O-2H (W) | 2 | 1270 .68 | 0 .34 | 1391000 | 68 |
| O64474 | Putative cadmium/zinc-transporting ... | Arabidopsis thaliana | ATSVMQSLMSLAPQK | 2 Ox (M),Deam (NQ) | 2 | 1623 .78 | -2 .08 | 1005900 | 50 |
| O64571 | UBP1-associated proteins 1C | Arabidopsis thaliana | VDLLVSSGVANGYSQAHKKR | 2 Deam (NQ) | 2 | 2130 .11 | 0 .89 | 1590200 | 73 |
| O64801 | Putative F-box protein At1g67450 | Arabidopsis thaliana | IVCKGDANQQFTGFTRYVNESTVCSMR | Deam (NQ) | 3 | 3168 .44 | -1 .79 | 2613600 | 40 |
| O65314 | Actin | Scherffelia dubia | VAPEEHPVLLTEAPLNPK | Umod | 3 | 1953 .06 | 1 .82 | 1544900 | 58 |
| O80689 | Beta-glucosidase 45 | Arabidopsis thaliana | ILDKNNADRAVDQYNR | 2 Deam (NQ),O-2H (Y) | 2 | 1919 .90 | 4 .14 | 852890 | 45 |
| O80690 | Beta-glucosidase 46 | Arabidopsis thaliana | RTPKQSATWYK | Deam (NQ),diOx Y | 2 | 1397 .69 | -1 .88 | 1841300 | 68 |
| O80760 | Polygalacturonase 1 beta-like prote... | Arabidopsis thaliana | GYGDGGNGAVYGFKNYR | Deam (NQ),Iodination,Di-iodination,O-2H (Y) | 3 | 2186 .47 | -0 .20 | 457590 | 42 |
| O81016 | ABC transporter G family member 32 | Arabidopsis thaliana | LGVDFAEIYRNSNLCQR | 2 Deam (NQ) | 3 | 2055 .97 | -1 .78 | 30852000 | 60 |
| O81072 | Probable xyloglucan galactosyltrans... | Arabidopsis thaliana | WLRESQEWK | Ox (W) | 3 | 1276 .62 | 3 .33 | 2010800 | 80 |
| O81345 | Cytochrome P450 79B1 | Sinapis alba | KVVMTELVCPARHR | TriOx (C),di-Ox (M) | 2 | 1774 .88 | -2 .48 | 287980 | 44 |
| O81769 | Probable diphthine methyl ester syn... | Arabidopsis thaliana | LGSEDQTIVAGTMK | Ox (M),Deam (NQ) | 2 | 1465 .70 | -1 .75 | 67057 | 51 |
| O81815 | Monooxygenase 1 | Arabidopsis thaliana | AVRGFTKYPNGHGFPQEVLR | O-2H (Y) | 3 | 2286 .17 | -0 .96 | 3624400 | 53 |
| O81972 | Cytochrome P450 82A2 | Glycine max | NAVVVSNWEMAK | Ox (M),Deam (NQ),Trp->Kynurenine | 2 | 1367 .64 | -1 .70 | 1288000 | 41 |
| O82043 | Ketol-acid reductoisomerase, chloro... | Pisum sativum | IDQTRMWKVGER | Trp->Kynurenine | 2 | 1521 .77 | -2 .05 | 1299100 | 63 |
| O82233 | Eukaryotic translation initiation f... | Arabidopsis thaliana | PGAMASMRNNNNNR | 2 Deam (NQ) | 3 | 1547 .66 | 3 .64 | 420530 | 49 |
| O82368 | Uncharacterized protein At2g29880 | Arabidopsis thaliana | ESGDQAGETSKKK | 2 Acetyl (K),Glu->pyro-Glu | 2 | 1429 .67 | 2 .87 | 2080700 | 54 |
| O82486 | N6-adenosine-methyltransferase MT-A... | Arabidopsis thaliana | SKGGSQVKYYCR | Acetyl (K),Deam (NQ) | 2 | 1474 .69 | -4 .32 | 6546900 | 78 |
| O82549 | Phospholipase D alpha 1 | Brassica oleracea var. capitata | LEGPIAWDVLYNFEQR | 2 Deam (NQ) | 2 | 1950 .94 | 2 .02 | 692050 | 52 |
| O82768 | Histidine biosynthesis bifunctional... | Arabidopsis thaliana | EALSTTISSR | Glu->pyro-Glu | 2 | 1045 .54 | 0 .81 | 1,2E+08 | 56 |
| P05492 | ATP synthase subunit alpha, mitocho... | Oenothera biennis | VISVGDGIAR | Umod | 2 | 985 .56 | -0 .32 | 1335700 | 144 |
| P06353 | Histone H3.3 | Hordeum vulgare | STELLIR | Umod | 2 | 830 .49 | -0 .27 | 3085300 | 143 |
| P06592 | Phytochrome A | Cucurbita pepo | NKVRMIVDCR | Ox (M),Deam (NQ) | 2 | 1306 .65 | 1 .70 | 708180 | 69 |
| P08477 | Glyceraldehyde-3-phosphate dehydrog... | Hordeum vulgare | VPTVDVSVVDLTVR | Umod | 2 | 1497 .84 | 0 .32 | 897230 | 123 |
| P09114 | Acetolactate synthase 2, chloroplas... | Nicotiana tabacum | NKQPHVSICADIK | Deam (NQ),TriOx (C) | 2 | 1557 .75 | -1 .23 | 1039100 | 42 |
| P09186 | Seed linoleate 9S-lipoxygenase-3 | Glycine max | NEPWWPKMQTR | O-2H (W),Di-Ox W | 2 | 1517 .67 | -4 .10 | 973230 | 66 |
| P09441 | Late embryogenesis abundant protein... | Gossypium hirsutum | AKASMQEKVDQMK | Acetyl (K),Ox (M),di-Ox (M) | 2 | 1582 .73 | -4 .11 | 1233900 | 55 |
| P0C130 | Putative auxin-responsive protein I... | Oryza sativa subsp. japonica | NHLWSVGPILQ | 2 Deam (NQ),Ox (W) | 2 | 1280 .64 | -2 .12 | 561380 | 72 |
| P0C523 | Cytochrome b | Oryza sativa subsp. indica | GIPKYYTDETHRTGSFS | Ox Y | 2 | 1973 .91 | -0 .31 | 886990 | 79 |
| P0C7R0 | Pentatricopeptide repeat-containing... | Arabidopsis thaliana | QVFDAMPSR | Ox (M),Gln->pyro-Glu | 2 | 1048 .46 | -3 .38 | 3148400 | 65 |
| P0C945 | Uncharacterized protein At1g21580 | Arabidopsis thaliana | STRQTTASKNEK | 2 Deam (NQ) | 2 | 1351 .66 | 3 .31 | 943240 | 65 |
| P11660 | Reverse transcriptase-like protein | Chlamydomonas reinhardtii | NQHSELNWLGHKVLFPSCTVK | Deam (NQ),Ox (W) | 3 | 2510 .24 | 1 .70 | 710420 | 54 |
| P11703 | DNA-directed RNA polymerase subunit... | Spinacia oleracea | IGPWGSLESPYYEISERSK | diOx Y | 3 | 2229 .06 | 0 .39 | 3513600 | 48 |
| P15590 | Globulin-1 S allele | Zea mays | PWHQRPR | Trp->Kynurenine | 2 | 979 .51 | -2 .57 | 14499000 | 67 |
| P16865 | Histone H2A-III | Volvox carteri | AGLQFPVGR | Umod | 2 | 943 .52 | 0 .19 | 3208100 | 111 |
| P17513 | Acidic endochitinase P | Nicotiana tabacum | YYGRGPIQLTNQNNYEK | Deam (NQ),O-2H (Y) | 3 | 2071 .96 | -0 .80 | 1,09E+08 | 51 |
| P24066 | 30S ribosomal protein S12, chloropl... | Triticum aestivum | MPTVKQLIRNAR | Acetyl (K),Ox (M),Deam (NQ) | 2 | 1484 .81 | -2 .26 | 419040 | 45 |
| P24226 | Histidinol dehydrogenase, chloropla... | Brassica oleracea var. capitata | AIEEEIAKQCK | Acetyl (K),Deam (NQ),TriOx (C) | 2 | 1408 .64 | -2 .39 | 3315800 | 74 |
| P24609 | Protein TAP2 | Antirrhinum majus | EASKENVGGNTNDIYK | Glu->pyro-Glu,O-2H (Y) | 2 | 1733 .79 | 2 .37 | 361610 | 61 |
| P26300 | Enolase | Solanum lycopersicum | AAVPSGASTGIYEALELR | Umod | 2 | 1803 .94 | 0 .32 | 936470 | 94 |
| P28405 | Ribulose bisphosphate carboxylase l... | Drosera capensis | VALEACVQARNHGQDLAR | TriOx (C) | 2 | 2054 .99 | -0 .50 | 1499000 | 58 |
| P29114 | Linoleate 9S-lipoxygenase 1 | Hordeum vulgare | SGNLTFVANSWIYPAANYR | 2 Deam (NQ),O-2H (Y) | 2 | 2159 .00 | 4 .09 | 461710 | 49 |
| P29197 | Chaperonin CPN60, mitochondrial | Arabidopsis thaliana | NVVIEQSWGAPK | 2 Deam (NQ),Di-Ox W | 2 | 1360 .65 | -1 .50 | 2672500 | 45 |
| P29382 | Developmental protein SEPALLATA 1 | Arabidopsis thaliana | ALAMKLDDMIGVR | Acetyl (K),2 di-Ox (M) | 2 | 1537 .75 | 4 .12 | 2154400 | 52 |
| P31155 | S-adenosylmethionine synthase 1 | Petroselinum crispum | TQVTVEYQNDHGAMVPIR | Deam (NQ),Ox Y | 3 | 2073 .98 | 2 .30 | 4129600 | 88 |
| P31167 | ADP,ATP carrier protein 1, mitochon... | Arabidopsis thaliana | GLYFGLYDSVK | diOx Y,O-2H (Y) | 2 | 1306 .61 | 4 .14 | 910550 | 61 |
| P31691 | ADP,ATP carrier protein, mitochondr... | Oryza sativa subsp. japonica | GIGDCFGRTIKDEGFASLWR | O-2H (W) | 3 | 2298 .09 | 1 .74 | 1252100 | 42 |
| P32296 | Delta-1-pyrroline-5-carboxylate syn... | Vigna aconitifolia | PGKIASLANNMR | 2 Deam (NQ),di-Ox (M) | 2 | 1304 .64 | -2 .51 | 514420 | 43 |
| P33628 | Tubulin alpha chain | Picea abies | AVFVDLEPTVIDEVR | Umod | 2 | 1700 .90 | -0 .28 | 2116700 | 199 |
| P34789 | 40S ribosomal protein S28-2 | Arabidopsis thaliana | EGDVLTLLESER | Umod | 2 | 1359 .69 | -0 .63 | 486170 | 103 |
| P34924 | Glyceraldehyde-3-phosphate dehydrog... | Pinus sylvestris | VPTPDVSVVDLTVR | Umod | 2 | 1495 .82 | 0 .68 | 2675900 | 152 |
| P35684 | 60S ribosomal protein L3 | Oryza sativa subsp. japonica | EIQMQLEKMK | Ox (M),Deam (NQ) | 2 | 1293 .63 | 3 .83 | 457590 | 62 |
| P37830 | Glucose-6-phosphate 1-dehydrogenase... | Solanum tuberosum | LQPSEAMYMKLTVK | Acetyl (K),2 di-Ox (M) | 2 | 1743 .84 | -1 .13 | 889710 | 65 |
| P40620 | HMG1/2-like protein | Vicia faba | SVAAVGKACGEEWK | Acetyl (K),TriOx (C) | 2 | 1580 .71 | 1 .19 | 2086100 | 80 |
| P41610 | 50S ribosomal protein L20, chloropl... | Pinus thunbergii | INAAARANGVSYNR | Deam (NQ),diOx Y | 2 | 1508 .73 | 0 .40 | 1182100 | 67 |
| P42158 | Casein kinase 1-like protein 1 | Arabidopsis thaliana | DNFPGSEELLQRSRTGDVSR | Deam (NQ) | 2 | 2263 .08 | 2 .39 | 2149200 | 68 |
| P42774 | G-box-binding factor 1 | Arabidopsis thaliana | KQAECEQLQQR | Acetyl (K) | 3 | 1458 .69 | -1 .06 | 2244600 | 56 |
| P43295 | Probable cysteine protease RD19B | Arabidopsis thaliana | ANLLRAMRHQK | Ox (M),Deam (NQ) | 2 | 1353 .73 | 4 .13 | 644090 | 58 |
| P45739 | Catalase | Helianthus annuus | VVGGLSDPR | Umod | 2 | 898 .49 | 0 .11 | 59944000 | 99 |
| P46640 | Homeobox protein knotted-1-like 2 | Arabidopsis thaliana | ENHVYKR | Deam (NQ),O-2H (Y) | 2 | 959 .45 | 0 .50 | 414350 | 42 |
| P47927 | Floral homeotic protein APETALA 2 | Arabidopsis thaliana | YVYLGLFDTEVEAARAYDK | Iodination,Di-iodination,O-2H (Y) | 4 | 2613 .76 | -3 .99 | 1985600 | 41 |
| P48006 | Elongation factor 1-delta 1 | Arabidopsis thaliana | MAAFPNLNSDAGLKK | Ox (M),Deam (NQ) | 3 | 1592 .79 | -4 .13 | 209600 | 49 |
| P49047 | Vacuolar-processing enzyme alpha-is... | Arabidopsis thaliana | YQKAPEGSARK | Deam (NQ) | 2 | 1234 .63 | -1 .19 | 3860000 | 91 |
| P51061 | Phosphoenolpyruvate carboxylase | Glycine max | KVIEENVKNLNMLQEMYNQWPFFR | Ox (M),3 Deam (NQ),Trp->Kynurenine | 3 | 3092 .46 | 1 .30 | 284930 | 52 |
| P51613 | Basic endochitinase | Vitis vinifera | GPIQISYNYNYGQAGK | Deam (NQ),Ox Y,diOx Y | 2 | 1820 .82 | 4 .21 | 337850 | 58 |
| P52427 | Proteasome subunit alpha type-4 | Spinacia oleracea | ATAIGANNQAAQSMLK | Ox (M),3 Deam (NQ) | 2 | 1606 .75 | -1 .32 | 1639200 | 47 |
| P52780 | Glutamine--tRNA ligase | Lupinus luteus | LNVSNTVMSKR | Ox (M),Deam (NQ) | 2 | 1264 .64 | -3 .08 | 1213200 | 50 |
| P56167 | Major pollen allergen Pha a 5.4 | Phalaris aquatica | PTSAGGYKV | Umod | 1 | 878 .45 | -0 .36 | 772690 | 46 |
| P58051 | Cytochrome P450 71B14 | Arabidopsis thaliana | AQAEVREVIKNK | Acetyl (K),Deam (NQ) | 2 | 1426 .78 | 0 .93 | 7812100 | 115 |
| P60838 | Disease resistance protein SUMM2 | Arabidopsis thaliana | VKNWSSVR | Deam (NQ),Ox (W) | 2 | 991 .51 | 1 .29 | 444380 | 69 |
| P61430 | Synaptonemal complex protein 2 | Arabidopsis thaliana | TRQTTMFQEPQR | 2 Deam (NQ) | 2 | 1523 .70 | -3 .10 | 1367300 | 65 |
| P69310 | Ubiquitin | Avena sativa | IQDKEGIPPDQQR | Umod | 3 | 1522 .77 | 1 .28 | 471300 | 83 |
| P80638 | Unknown protein from spot 75 of 2D-... | Zea mays | EWSELFTK | Glu->pyro-Glu,Di-Ox W | 2 | 1052 .48 | -3 .89 | 650610 | 49 |
| P83442 | Late embryogenesis abundant protein... | Daucus carota | EKTGGAMQATK | Acetyl (K),Glu->pyro-Glu,Deam (NQ) | 2 | 1145 .54 | -2 .08 | 338140 | 60 |
| P85920 | Malate dehydrogenase | Pseudotsuga menziesii | LFGVTTLDVVR | Umod | 2 | 1218 .70 | -0 .80 | 3877200 | 141 |
| P85945 | Histone H4 | Pseudotsuga menziesii | ISGLIYEETR | Umod | 2 | 1179 .61 | -0 .11 | 3591500 | 159 |
| P86451 | Trypsin inhibitor | Enterolobium contortisiliquum | ELLDSDGDILR | Glu->pyro-Glu | 2 | 1226 .61 | -1 .08 | 1319700 | 91 |
| P92958 | SNF1-related protein kinase catalyt... | Arabidopsis thaliana | EIMNEVLK | Ox (M),Glu->pyro-Glu,Deam (NQ) | 2 | 973 .48 | 3 .38 | 1398800 | 51 |
| P93149 | Licodione synthase | Glycyrrhiza echinata | QLLQTNELAFNCR | Deam (NQ),TriOx (C) | 2 | 1654 .76 | -2 .51 | 621170 | 46 |
| P93377 | 40S ribosomal protein S14 | Nicotiana tabacum | TPGPGAQSALR | Umod | 2 | 1053 .56 | 0 .12 | 416120 | 106 |
| P93751 | Zinc finger protein 8 | Arabidopsis thaliana | DKDKDNNNNR | 3 Deam (NQ) | 2 | 1234 .51 | 1 .64 | 443940 | 43 |
| Q00WL5 | Lon protease homolog, mitochondrial | Ostreococcus tauri | TYVGAMPGKLIQCLK | Deam (NQ),Iodination | 2 | 1804 .78 | -1 .48 | 871300 | 41 |
| Q03460 | Glutamate synthase [NADH], amylopla... | Medicago sativa | APGNPWPQWPR | Ox (W),Trp->Kynurenine | 2 | 1324 .63 | 4 .07 | 1151100 | 44 |
| Q03878 | Glycine-rich RNA-binding protein | Daucus carota | EGGGGGYGGGGGYGGRR | Ox Y,diOx Y | 2 | 1517 .62 | 4 .39 | 426320 | 46 |
| Q04648 | Probable cytochrome c biosynthesis ... | Oenothera berteroana | AQPRPQLLWKN | Trp->Kynurenine | 2 | 1353 .75 | -1 .21 | 709030 | 61 |
| Q04996 | Homeobox protein HAT3.1 | Arabidopsis thaliana | KNKTMNK | 2 Acetyl (K),Ox (M) | 2 | 962 .49 | -1 .88 | 825620 | 57 |
| Q06735 | ATP synthase subunit alpha, mitocho... | Beta vulgaris | VVSVGDGIAR | Umod | 2 | 971 .54 | 0 .22 | 513730 | 116 |
| Q06735;P05492 | Protein Ycf2 | Drimys granadensis | VVDALGVPIDGR | Umod | 2 | 1209 .67 | -1 .91 | 473260 | 96 |
| Q06GT4 | Protein Ycf2 | Jasminum nudiflorum | EFFDRLSPRK | Umod | 3 | 1293 .68 | -3 .97 | 428420 | 48 |
| Q06R68 | Uncharacterized membrane protein yc... | Stigeoclonium helveticum | NMVESKTLYLR | Ox (M),O-2H (Y) | 2 | 1382 .69 | -4 .15 | 9178500 | 61 |
| Q06SH2 | Kinesin-like protein KIN-14C | Arabidopsis thaliana | SKNYLQSLNLQKSEELNQK | 3 Deam (NQ) | 3 | 2266 .13 | -0 .60 | 1227900 | 51 |
| Q07970 | 30S ribosomal protein S8, chloropla... | Citrus sinensis | IEQMTDIIKR | Acetyl (K),Deam (NQ),di-Ox (M) | 2 | 1320 .66 | -3 .18 | 817380 | 67 |
| Q09ME2 | Protein ADP-ribosyltransferase PARP... | Oryza sativa subsp. japonica | PGLRIYFNYR | Deam (NQ),2 diOx Y | 2 | 1362 .66 | -3 .68 | 1227700 | 49 |
| Q0E0Q3 | BURP domain-containing protein 4 | Oryza sativa subsp. japonica | SRTLAASQEEGK | Deam (NQ) | 3 | 1276 .63 | 0 .21 | 2010800 | 71 |
| Q0E1Z0 | Serine/threonine protein phosphatas... | Oryza sativa subsp. japonica | EAGRYVDDSSQAAR | Glu->pyro-Glu,Deam (NQ) | 2 | 1506 .67 | -1 .21 | 161270 | 41 |
| Q0E2P1 | DNA-directed RNA polymerase subunit... | Daucus carota | RPQPQPQPR | 2 Deam (NQ) | 2 | 1104 .57 | 1 .41 | 2518500 | 69 |
| Q0G9X1 | Probable cytokinin riboside 5'-mono... | Oryza sativa subsp. japonica | STPGELIMCQEK | Ox (M),Deam (NQ) | 2 | 1408 .62 | 2 .25 | 2472800 | 73 |
| Q0JBP5 | Cyclin-B1-3 | Oryza sativa subsp. japonica | AVADMHQRKAEMAR | Ox (M),di-Ox (M) | 2 | 1660 .78 | -2 .37 | 2499800 | 50 |
| Q0JNK6 | Cellulose synthase-like protein G3 | Arabidopsis thaliana | YEETWAPLVK | Umod | 2 | 1234 .62 | 4 .39 | 3860000 | 106 |
| Q0WVN5 | Protein TIC 214 | Vitis vinifera | IGFRYGSLVEDYYTGYR | 2 Ox Y | 2 | 2089 .97 | 2 .32 | 988750 | 46 |
| Q0ZIW0 | Class II metallothionein-like prote... | Oryza sativa subsp. japonica | QKDSIINNQMIHK | Ox (M),Gln->pyro-Glu | 2 | 1566 .78 | -2 .16 | 2473700 | 47 |
| Q109B0 | 30S ribosomal protein S3, chloropla... | Populus alba | ESQPTGRENRR | Glu->pyro-Glu,2 Deam (NQ) | 2 | 1312 .61 | -3 .99 | 235120 | 40 |
| Q14FB9 | DNA-directed RNA polymerase subunit... | Tetradesmus obliquus | ELQINVQKELNCMNRK | Ox (M),3 Deam (NQ) | 2 | 2034 .97 | 1 .42 | 953890 | 43 |
| Q1KVT3 | DNA-directed RNA polymerase subunit... | Tetradesmus obliquus | ENIFYSQNFR | Glu->pyro-Glu,Deam (NQ),Ox Y | 2 | 1315 .58 | -3 .32 | 3206000 | 70 |
| Q1KVX8 | Transcription factor bHLH19 | Arabidopsis thaliana | KASEISTIQK | Acetyl (K) | 2 | 1145 .63 | 0 .90 | 1074600 | 84 |
| Q1PF16 | Kinesin-like protein KIN-12D | Arabidopsis thaliana | MKQLQEQLRTLK | 3 Deam (NQ) | 2 | 1517 .81 | -1 .07 | 4241500 | 79 |
| Q27IK6 | DNA-directed RNA polymerase subunit... | Helicosporidium sp. subsp. Simulium jonesii | EINDLQAMVSDIR | Ox (M) | 2 | 1518 .73 | -0 .53 | 1120200 | 79 |
| Q2EEX2 | Maturase K | Cucumis sativus | LINNNFKDENNDNLKNMR | Acetyl (K),Deam (NQ) | 4 | 2248 .05 | -4 .42 | 4488600 | 90 |
| Q2QDA7 | B3 domain-containing protein Os12g0... | Oryza sativa subsp. japonica | GLFERIYFYGK | 2 diOx Y | 2 | 1455 .70 | -1 .41 | 4413600 | 63 |
| Q2QMT7 | PP2A regulatory subunit TAP46 | Oryza sativa subsp. japonica | DQNMRLRLPETK | Ox (M),2 Deam (NQ) | 3 | 1517 .75 | 3 .28 | 1920900 | 48 |
| Q2QY04 | Disease resistance protein PIK6-NP | Oryza sativa subsp. japonica | ERMAAQVFQPSYR | Glu->pyro-Glu,Ox Y | 2 | 1579 .76 | 2 .00 | 64725000 | 60 |
| Q2QZF1 | Tryptamine benzoyltransferase 1 | Oryza sativa subsp. japonica | MYELLTWWPRR | Ox (M),O-2H (Y) | 2 | 1579 .76 | -0 .69 | 64754000 | 64 |
| Q2R0K3 | Auxin response factor 23 | Oryza sativa subsp. japonica | VAVNCRRR | Deam (NQ) | 2 | 1030 .55 | -4 .21 | 479960 | 90 |
| Q2R3F5 | Clathrin heavy chain 1 | Oryza sativa subsp. japonica | MNPGTLNSRSEDSHANSMERGSVGR | Deam (NQ),di-Ox (M) | 3 | 2721 .18 | 0 .56 | 4518400 | 43 |
| Q2RBN7 | Phototropin-1B | Oryza sativa subsp. japonica | VMDYVNR | Ox (M),Deam (NQ),Ox Y | 2 | 928 .40 | -0 .04 | 854110 | 65 |
| Q2RBR1 | 11S globulin seed storage protein J... | Juglans regia | GLVTNNRTDMF | Ox (M),2 Deam (NQ) | 2 | 1284 .57 | -2 .28 | 1123900 | 71 |
| Q2TPW5 | Uncharacterized protein ycf66 | Marchantia polymorpha | PRWSREEQER | Umod | 3 | 1371 .66 | -0 .97 | 208540 | 43 |
| Q32616 | Protein TIC 214 | Zygnema circumcarinatum | NIYKYIYENFK | Deam (NQ),Ox Y,diOx Y | 2 | 1542 .72 | 1 .21 | 980940 | 81 |
| Q32RK5 | Photosystem II reaction center prot... | Staurastrum punctulatum | KQTDIQQMPEIR | Deam (NQ),di-Ox (M) | 2 | 1518 .73 | -1 .04 | 7173200 | 115 |
| Q32RU4 | DNA-directed RNA polymerase subunit... | Staurastrum punctulatum | ATQIIKDANSKGR | Deam (NQ) | 2 | 1401 .76 | 3 .43 | 2922300 | 89 |
| Q32RY2 | Peptidyl-prolyl cis-trans isomerase... | Arabidopsis thaliana | VFLLSIYNR | diOx Y | 2 | 1155 .63 | 2 .75 | 2224400 | 97 |
| Q38931 | Receptor-like serine/threonine-prot... | Arabidopsis thaliana | AQAYMELSDLDLAEFDVK | Deam (NQ),di-Ox (M) | 2 | 2089 .94 | 3 .34 | 1088700 | 67 |
| Q39086 | Dynein alpha chain, flagellar outer... | Chlamydomonas reinhardtii | NKGFYNSNRDLNLLGFVWR | 2 Deam (NQ),Ox Y | 3 | 2330 .14 | -3 .75 | 1072600 | 41 |
| Q39610 | Probable transcription factor At4g0... | Arabidopsis thaliana | DGLLSYYMR | Ox (M),diOx Y,O-2H (Y) | 2 | 1178 .49 | -0 .74 | 797600 | 49 |
| Q3EAE7 | Probable F-box protein At1g53815 | Arabidopsis thaliana | DAKKENSQMLK | 2 Deam (NQ) | 2 | 1292 .63 | -1 .70 | 1247200 | 108 |
| Q3ECQ9 | Protein Ycf2 | Acorus calamus | QKQYVSDVNLTVCGSNK | 2 Deam (NQ) | 2 | 1940 .91 | -4 .40 | 1335700 | 52 |
| Q3V4Z0 | Elongation factor Tu, chloroplastic | Tupiella akineta | KLQEMCLEYLSDRER | Ox (M),Deam (NQ) | 3 | 1985 .92 | -0 .17 | 824190 | 44 |
| Q3ZJ24 | 30S ribosomal protein S19, chloropl... | Tupiella akineta | LKMVVELIQPIAVENGMR | Acetyl (K),Deam (NQ),di-Ox (M) | 2 | 2114 .11 | -1 .31 | 59465000 | 68 |
| Q3ZJ86 | Protein translocase subunit SecA, c... | Pisum sativum | LNDLDEKQVIK | Acetyl (K),Deam (NQ) | 2 | 1356 .71 | 0 .20 | 3158200 | 115 |
| Q41062 | Flavonol synthase/flavanone 3-hydro... | Solanum tuberosum | NVIYSIYQFKPVLLKQDQDK | Iodination | 3 | 2564 .22 | 0 .40 | 4107300 | 53 |
| Q41452 | Protochlorophyllide reductase A, ch... | Triticum aestivum | EANEEYAKWLRK | Deam (NQ),Ox (W) | 2 | 1552 .75 | 4 .34 | 567510 | 61 |
| Q41578 | Sucrose synthase 2 | Tulipa gesneriana | LLMEDLQKSDYPSR | Acetyl (K),Ox (M),Deam (NQ) | 2 | 1752 .82 | 3 .10 | 2835500 | 63 |
| Q41607 | Late embryogenesis abundant protein... | Zea mays | MYSLIEEYKLDGHIRWISAQMNR | Ox (M) | 3 | 2868 .41 | -2 .72 | 9971400 | 41 |
| Q42376 | Uridine 5'-monophosphate synthase | Arabidopsis thaliana | QKAAEAGQYAK | 2 Deam (NQ),Ox Y | 2 | 1181 .56 | -0 .05 | 2160700 | 80 |
| Q42586 | DNA-directed RNA polymerase II subu... | Solanum lycopersicum | VQGWNAYLEK | Deam (NQ),O-2H (W) | 2 | 1221 .57 | -3 .15 | 2212100 | 58 |
| Q42877 | Beta-fructofuranosidase, cell wall ... | Pisum sativum | NTYQSAMGK | Ox (M),Deam (NQ) | 2 | 1015 .43 | 1 .90 | 814500 | 51 |
| Q43089 | Isocitrate lyase | Pinus taeda | QLVQWPVK | Deam (NQ),Ox (W) | 2 | 1013 .55 | -1 .29 | 356820 | 61 |
| Q43097 | Glucose-6-phosphate 1-dehydrogenase... | Arabidopsis thaliana | LGISNLFWDWDLPR | Deam (NQ),Ox (W),Trp->Kynurenine | 2 | 1751 .85 | -4 .11 | 2205300 | 86 |
| Q43727 | Expansin-A22 | Oryza sativa subsp. japonica | VQPDEGIYLRINNK | 2 Deam (NQ) | 2 | 1659 .85 | 0 .92 | 2544900 | 87 |
| Q4PR44 | Cytochrome P450 704C1 | Pinus taeda | KRAPQWCR | Deam (NQ),Di-Ox W | 2 | 1133 .54 | 3 .73 | 334940 | 49 |
| Q50EK3 | Probable ethylene response sensor 1 | Oryza sativa subsp. japonica | PERWIQDGIFHPK | Deam (NQ),Trp->Kynurenine | 2 | 1626 .82 | 3 .28 | 1325100 | 60 |
| Q53RH0 | Heat shock 70 kDa protein BIP2 | Oryza sativa subsp. japonica | LMQTILNISGNAVK | Ox (M),Deam (NQ) | 2 | 1517 .81 | -2 .16 | 5354400 | 81 |
| Q53RJ5 | Transcription factor bHLH71 | Arabidopsis thaliana | IINEPTAAAIAYGLDR | Umod | 2 | 1686 .89 | -1 .11 | 831240 | 100 |
| Q56XR0 | WPP domain-associated protein | Solanum lycopersicum | RQMNQHLSVLR | Deam (NQ),di-Ox (M) | 2 | 1413 .71 | -1 .96 | 1368100 | 56 |
| Q5BQN5 | DExH-box ATP-dependent RNA helicase... | Arabidopsis thaliana | LMQMEKLVNEKEK | Acetyl (K),2 Ox (M) | 2 | 1692 .84 | 2 .93 | 970670 | 47 |
| Q5D892 | Plant intracellular Ras-group-relat... | Arabidopsis thaliana | QGISKLAVTLSR | Acetyl (K),Deam (NQ) | 3 | 1314 .75 | -0 .63 | 587070 | 51 |
| Q5G5D8 | DnaJ protein P58IPK homolog B | Oryza sativa subsp. japonica | MNGKLVNTAAKK | Acetyl (K),2 Deam (NQ),di-Ox (M) | 2 | 1349 .69 | -1 .04 | 668960 | 59 |
| Q5JNB5 | Nucleosome assembly protein 1;3 | Oryza sativa subsp. indica | EALMRAEKQLK | Acetyl (K),Deam (NQ) | 2 | 1358 .72 | 4 .05 | 2367000 | 89 |
| Q5MGA9 | Probable ion channel POLLUX | Oryza sativa subsp. japonica | YHKLYGPLYSKR | 2 O-2H (Y) | 2 | 1551 .78 | 4 .14 | 700730 | 68 |
| Q5N941 | Probable protein phosphatase 2C 54 | Oryza sativa subsp. japonica | VDSWRKGK | O-2H (W) | 2 | 988 .51 | -1 .13 | 3171200 | 67 |
| Q5SMK6 | Zinc finger CCCH domain-containing ... | Oryza sativa subsp. japonica | TLLIANAGDCR | Deam (NQ),TriOx (C) | 2 | 1251 .58 | 4 .38 | 3373500 | 78 |
| Q5SNN4 | Probable isoprenylcysteine alpha-ca... | Oryza sativa subsp. japonica | MLRAEYDK | Umod | 1 | 1024 .50 | 4 .29 | 1352800 | 43 |
| Q5VNW5 | Calcium permeable stress-gated cati... | Arabidopsis thaliana | ESSGQSISWSVTQIK | Glu->pyro-Glu,Deam (NQ) | 2 | 1618 .78 | 0 .17 | 2164800 | 75 |
| Q5XEZ5 | Probable histone acetyltransferase ... | Oryza sativa subsp. japonica | LQNWLDYYQLK | 2 Deam (NQ),Di-Ox W | 2 | 1516 .71 | -0 .29 | 1172500 | 51 |
| Q5Z8V7 | ABC transporter G family member 42 | Oryza sativa subsp. japonica | ESVTVVDVKKK | Glu->pyro-Glu | 2 | 1212 .71 | 1 .12 | 2008400 | 104 |
| Q5Z9S8 | Mitogen-activated protein kinase 10 | Oryza sativa subsp. japonica | LMSIGSNEAGPRR | Ox (M),Deam (NQ) | 2 | 1403 .68 | -2 .65 | 5283900 | 65 |
| Q5ZCI1 | Lectin-domain containing receptor k... | Arabidopsis thaliana | QFAHLEENGGNGPVIPMDR | Ox (M),2 Deam (NQ) | 3 | 2097 .94 | -3 .75 | 585040 | 42 |
| Q66GN2 | NO-associated protein 1, chloroplas... | Arabidopsis thaliana | SSSDQIAVKKITPNSMQGVR | 2 Acetyl (K),Deam (NQ),di-Ox (M) | 2 | 2262 .12 | -0 .82 | 2597500 | 62 |
| Q66GP9 | Protein NETWORKED 3A | Arabidopsis thaliana | NQMQEWKGLQSHR | Acetyl (K),2 Deam (NQ),di-Ox (M) | 2 | 1716 .75 | 2 .35 | 557620 | 60 |
| Q66GR8 | Protein root UVB sensitive 4 | Arabidopsis thaliana | VTNTVVAR | Deam (NQ) | 2 | 859 .48 | 0 .80 | 4913000 | 163 |
| Q67YT8 | Zinc-finger homeodomain protein 6 | Oryza sativa subsp. japonica | RAARDDNIALNK | Deam (NQ) | 2 | 1356 .71 | 0 .71 | 3158200 | 100 |
| Q688U3 | CBL-interacting protein kinase 19 | Oryza sativa subsp. japonica | VWMHNNKSSIGSSSGGGSR | Acetyl (K),Deam (NQ),di-Ox (M) | 2 | 2021 .89 | -3 .67 | 3025700 | 52 |
| Q68Y49 | Glucose-1-phosphate adenylyltransfe... | Oryza sativa subsp. japonica | EVMESRWFQK | Glu->pyro-Glu,Ox (W) | 2 | 1336 .62 | 2 .02 | 2102100 | 51 |
| Q69T99 | Cytochrome P450 734A4 | Oryza sativa subsp. japonica | AYGNNIGGYK | Deam (NQ),Ox Y,diOx Y | 2 | 1104 .47 | 0 .59 | 337440 | 49 |
| Q69XM6 | Protein ALWAYS EARLY 1 | Arabidopsis thaliana | VADAVWWRPRR | Trp->Kynurenine,Di-Ox W | 2 | 1446 .75 | -0 .79 | 3717600 | 96 |
| Q6A331 | bZIP transcription factor 39 | Oryza sativa subsp. japonica | FLQDERDKLQEYR | Deam (NQ),Ox Y | 2 | 1755 .84 | -1 .07 | 2601200 | 82 |
| Q6AU90 | DNA-directed RNA polymerases IV and... | Arabidopsis thaliana | QASPGPSSDAAKCR | Acetyl (K) | 2 | 1472 .67 | 0 .20 | 66929000 | 90 |
| Q6DBA5 | ABC transporter G family member 47 | Oryza sativa subsp. japonica | CGKSDKPTK | TriOx (C) | 2 | 1067 .49 | -2 .87 | 2513800 | 65 |
| Q6EQ60 | Protein SABRE | Arabidopsis thaliana | TSKYGVSMKELLQANIDR | Acetyl (K),Ox (M),2 Deam (NQ) | 3 | 2112 .04 | 3 .58 | 3245900 | 59 |
| Q6IMT1 | Protein SABRE | Arabidopsis thaliana | VQVLISDLEVVMR | Ox (M),Deam (NQ) | 2 | 1516 .82 | -1 .40 | 719750 | 50 |
| Q6K8S0 | Cyclin-F2-2 | Oryza sativa subsp. japonica | DMEKDAAQR | Acetyl (K),Ox (M),Deam (NQ) | 2 | 1121 .47 | 2 .61 | 2451700 | 66 |
| Q6NQH4 | Transcription initiation factor TFI... | Arabidopsis thaliana | SKAAGTSQPQEKR | 2 Deam (NQ) | 2 | 1388 .69 | 2 .43 | 863910 | 60 |
| Q6PV68 | AP2-like ethylene-responsive transc... | Arabidopsis thaliana | QSSIFLPMATMK | Ox (M),Gln->pyro-Glu | 2 | 1351 .65 | 1 .09 | 1563500 | 51 |
| Q6QNI4 | Psoralen synthase | Ammi majus | GCPGIQFAMCINELVVANLVHK | Deam (NQ) | 3 | 2470 .22 | -2 .55 | 8240300 | 46 |
| Q6UTZ2 | Probable monogalactosyldiacylglycer... | Oryza sativa subsp. japonica | DLGKEYGGWPLNDMERSYK | Acetyl (K),Ox (M),Deam (NQ) | 3 | 2316 .04 | -2 .56 | 2537500 | 46 |
| Q6YZI0 | Probable UDP-N-acetylglucosamine--p... | Oryza sativa subsp. japonica | AIFANSTYAEAYNNLGVLYR | Deam (NQ),Ox Y | 2 | 2266 .09 | 2 .55 | 441310 | 41 |
| Q6YZX6 | Putative aconitate hydratase, cytop... | Oryza sativa subsp. japonica | SPNAVQSNMELEFKR | Ox (M),2 Deam (NQ) | 2 | 1766 .81 | 0 .73 | 870590 | 45 |
| Q6Z3A8 | Clathrin light chain 3 | Oryza sativa subsp. japonica | LNGDTNRAQNR | 2 Deam (NQ) | 2 | 1259 .59 | -1 .51 | 1927300 | 66 |
| Q6ZJ48 | Probable adenylate kinase 7, mitoch... | Oryza sativa subsp. japonica | LAEVLAVPYISMGTLVR | Ox (M),Iodination | 2 | 1972 .92 | 1 .83 | 502720 | 43 |
| Q6ZL57 | Auxin-responsive protein IAA24 | Oryza sativa subsp. japonica | QQQQGGGLYVK | Gln->pyro-Glu,diOx Y | 2 | 1219 .58 | 0 .15 | 719500 | 48 |
| Q700D9 | Putative Myb family transcription f... | Arabidopsis thaliana | FQSHHSLEAENTKNIWK | 3 Deam (NQ),Trp->Kynurenine | 3 | 2074 .96 | 3 .35 | 2719100 | 81 |
| Q7G6K7 | Formin-like protein 3 | Oryza sativa subsp. japonica | GIGLAQQSNPPKK | 2 Deam (NQ) | 2 | 1338 .71 | -0 .03 | 535350 | 62 |
| Q7X659 | Vacuolar protein sorting-associated... | Arabidopsis thaliana | LLKKPDQCR | Deam (NQ) | 2 | 1157 .62 | -0 .65 | 251870 | 85 |
| Q7X996 | CBL-interacting protein kinase 2 | Oryza sativa subsp. japonica | GLDAKLLRYNLQPK | Deam (NQ),Iodination | 2 | 1754 .82 | -0 .72 | 1596100 | 48 |
| Q7XQP4 | Serine/threonine-protein kinase SAP... | Oryza sativa subsp. japonica | EIRNHPWFLKNLPR | Deam (NQ),O-2H (W) | 3 | 1833 .96 | 2 .46 | 6259400 | 76 |
| Q7XRA1 | Arginine decarboxylase 2 | Oryza sativa subsp. japonica | GLTTMPYLNDYKPPK | Deam (NQ),O-2H (Y) | 2 | 1751 .84 | 3 .81 | 2566800 | 90 |
| Q7XTH4 | Endoglucanase 11 | Oryza sativa subsp. japonica | TLRALARK | Umod | 1 | 927 .60 | 0 .33 | 49770000 | 40 |
| Q7XUP6 | Peptide methionine sulfoxide reduct... | Oryza sativa subsp. japonica | ESLEAKQEEWK | Acetyl (K),Glu->pyro-Glu,Deam (NQ) | 2 | 1400 .65 | -2 .95 | 1094300 | 88 |
| Q7XYS3 | Allene oxide synthase 2 | Oryza sativa subsp. japonica | KLLQYVYWSNGR | Deam (NQ),Ox (W) | 2 | 1542 .78 | 0 .17 | 632820 | 55 |
| Q7XZU0 | Probable phosphoinositide phosphata... | Arabidopsis thaliana | ADTQVIYIDPTTGILRYNGK | Deam (NQ),Ox Y | 3 | 2254 .15 | 1 .15 | 521400 | 54 |
| Q7Y1Z0 | Chitinase 5 | Oryza sativa subsp. japonica | NNKQWPCQPGK | Deam (NQ),Ox (W) | 2 | 1372 .62 | 3 .18 | 826620 | 56 |
| Q7YJS6 | Protein TIC 214 | Calycanthus floridus var. glaucus | YFSNISNKKSR | 2 Acetyl (K),2 Deam (NQ) | 2 | 1428 .69 | -1 .26 | 258200 | 62 |
| Q84JT7 | AT-rich interactive domain-containi... | Arabidopsis thaliana | ESASNDAVKEFQGSKLAER | Acetyl (K),Glu->pyro-Glu | 2 | 2089 .01 | 1 .21 | 978760 | 51 |
| Q84LB2 | (E,E)-alpha-farnesene synthase | Malus domestica | KNIKGMIDNAWK | Acetyl (K),2 Deam (NQ),di-Ox (M) | 2 | 1492 .72 | -2 .29 | 379800 | 46 |
| Q84T65 | Protein Brevis radix-like 4 | Oryza sativa subsp. japonica | ERIQAQYL | Glu->pyro-Glu,Ox Y | 2 | 1017 .52 | 0 .26 | 2920100 | 43 |
| Q84TH4 | Serine/arginine-rich splicing facto... | Arabidopsis thaliana | ESRGFGFISMKSVGDANR | Glu->pyro-Glu,Deam (NQ) | 2 | 1939 .92 | 4 .27 | 2390600 | 43 |
| Q84TI3 | Protein OBERON 4 | Arabidopsis thaliana | ENIRQMMLNMDK | Ox (M),Deam (NQ) | 2 | 1538 .69 | -4 .49 | 963420 | 79 |
| Q84UP7 | Probable mixed-linked glucan syntha... | Oryza sativa subsp. japonica | KEYDDFKAR | Acetyl (K) | 2 | 1212 .58 | 0 .10 | 4051900 | 96 |
| Q84UU4 | Alpha-humulene/(-)-(E)-beta-caryoph... | Arabidopsis thaliana | AVEVMGNIVSDAWK | Ox (M),Di-Ox W | 2 | 1565 .74 | -2 .56 | 5343600 | 46 |
| Q851W4 | B3 domain-containing protein Os03g0... | Oryza sativa subsp. japonica | KDQVGYILHGR | Iodination | 3 | 1410 .59 | 2 .75 | 1234600 | 67 |
| Q851W5 | B3 domain-containing protein Os03g0... | Oryza sativa subsp. japonica | SNAWQVKMRPR | 2 Deam (NQ) | 2 | 1373 .69 | -1 .27 | 1492000 | 74 |
| Q8GT06 | Crossover junction endonuclease MUS... | Oryza sativa subsp. japonica | ENLAHTLYKSYR | 2 diOx Y | 2 | 1557 .74 | -3 .57 | 2254900 | 67 |
| Q8GWB7 | Inositol phosphorylceramide glucuro... | Arabidopsis thaliana | PWDWWTAWLVK | O-2H (W),Di-Ox W | 2 | 1532 .71 | 2 .01 | 829950 | 50 |
| Q8GY79 | Double-stranded RNA-binding protein... | Arabidopsis thaliana | YNFVGGCSVNPYSLAPAVQMR | Deam (NQ),diOx Y | 2 | 2362 .07 | -4 .34 | 350200 | 46 |
| Q8GZA6 | Pentatricopeptide repeat-containing... | Arabidopsis thaliana | LLDEIKLVGTVNMDR | Ox (M),Deam (NQ) | 3 | 1731 .91 | -1 .69 | 796770 | 44 |
| Q8GZU0 | Minovincinine 19-hydroxy-O-acetyltr... | Catharanthus roseus | LKNLSQEKLNYVAR | Acetyl (K) | 2 | 1716 .95 | -1 .37 | 555760 | 77 |
| Q8H191 | Probable polyamine oxidase 4 | Arabidopsis thaliana | ILEETEKIR | Acetyl (K) | 2 | 1171 .64 | 0 .23 | 1451300 | 90 |
| Q8H3P9 | Potassium transporter 7 | Oryza sativa subsp. japonica | YLFDLQNKVSMK | Acetyl (K),Ox (M),2 Deam (NQ) | 2 | 1544 .74 | -3 .07 | 942080 | 73 |
| Q8H7T4 | Expansin-B10 | Oryza sativa subsp. japonica | LVANDVIPDNWK | Di-Ox W | 2 | 1414 .71 | 1 .22 | 1102900 | 85 |
| Q8HVY3 | DNA-directed RNA polymerase subunit... | Glycine max | KDQDQMNIHYLSTGERDFCNLLASK | Deam (NQ),Ox Y | 3 | 2999 .38 | -4 .09 | 1725900 | 41 |
| Q8L817 | Alpha carbonic anhydrase 7 | Arabidopsis thaliana | NVGMIDPTK | Ox (M),Deam (NQ) | 2 | 990 .47 | 0 .80 | 633090 | 41 |
| Q8L840 | ATP-dependent DNA helicase Q-like 4... | Arabidopsis thaliana | ESQKSQFLSSTATR | Glu->pyro-Glu,Deam (NQ) | 2 | 1551 .75 | 2 .59 | 299620 | 49 |
| Q8LGB6 | Non-functional pseudokinase ZED1 | Arabidopsis thaliana | GVIGFVDPDYYWTMKVTEK | Ox (M) | 2 | 2263 .09 | 0 .90 | 2483400 | 59 |
| Q8LGH4 | Cullin-4 | Arabidopsis thaliana | QALSSYVR | Gln->pyro-Glu,diOx Y | 2 | 937 .45 | -1 .09 | 602610 | 42 |
| Q8LK56 | Transcriptional activator DEMETER | Arabidopsis thaliana | NQLIGFPFGNQQPR | 3 Deam (NQ) | 2 | 1617 .78 | 1 .08 | 382850 | 48 |
| Q8LNZ2 | Kinesin-like protein KIN-7B | Arabidopsis thaliana | NAAEENIRNIK | 2 Deam (NQ) | 2 | 1272 .63 | 0 .12 | 5762000 | 110 |
| Q8LPF8 | O-fucosyltransferase 29 | Arabidopsis thaliana | LANDLDEDMQKLR | Ox (M) | 2 | 1575 .76 | -2 .44 | 830850 | 77 |
| Q8M9V0 | 30S ribosomal protein S3, chloropla... | Chaetosphaeridium globosum | IRHYIYQYVQKHIR | Ox Y,O-2H (Y) | 2 | 1946 .03 | 3 .84 | 3692400 | 64 |
| Q8MA11 | DNA-directed RNA polymerase subunit... | Chaetosphaeridium globosum | IGNQIMKDRNNR | Ox (M),3 Deam (NQ) | 3 | 1476 .70 | -2 .54 | 619660 | 59 |
| Q8MCN0 | Maturase K | Trifolium hybridum | LITRMSQQNHLIISANDSNK | 2 Deam (NQ) | 3 | 2284 .15 | -3 .59 | 2102000 | 49 |
| Q8RWK8 | Coilin | Arabidopsis thaliana | PGEMLLANEEFQK | Deam (NQ),di-Ox (M) | 2 | 1537 .70 | 4 .08 | 1837900 | 47 |
| Q8RWL2 | Calcium-dependent protein kinase 29 | Arabidopsis thaliana | GLKQTFKNMDTDESGTITFDELR | Acetyl (K),Ox (M),2 Deam (NQ) | 3 | 2705 .24 | -3 .51 | 8641200 | 45 |
| Q8RXY0 | Probable inactive protein kinase At... | Arabidopsis thaliana | QGGISLASWK | Deam (NQ),Ox (W) | 2 | 1062 .53 | 2 .39 | 3258800 | 55 |
| Q8RY24 | Probable sucrose-phosphate synthase... | Arabidopsis thaliana | KQLEWEDSQR | Deam (NQ) | 2 | 1318 .62 | -2 .07 | 3436500 | 120 |
| Q8RY67 | Wall-associated receptor kinase-lik... | Arabidopsis thaliana | GKLQNDEWVAIKR | O-2H (W) | 2 | 1569 .83 | 0 .81 | 1222600 | 79 |
| Q8S0G4 | Probable glycerol-3-phosphate dehyd... | Oryza sativa subsp. japonica | NAWYGQMLAK | Ox (M),Deam (NQ),O-2H (Y) | 2 | 1211 .53 | 4 .33 | 846590 | 45 |
| Q8S151 | MADS-box transcription factor 32 | Oryza sativa subsp. japonica | VGSMCDLLEKQLR | Acetyl (K),Deam (NQ),di-Ox (M) | 2 | 1622 .76 | -1 .11 | 338410 | 49 |
| Q8S2T0 | Protein GRIP | Arabidopsis thaliana | SQFEGLKDEVAQGRSLQK | 3 Deam (NQ) | 2 | 2021 .99 | -2 .89 | 2425400 | 54 |
| Q8S6N5 | Acetyl-CoA carboxylase 1 | Oryza sativa subsp. japonica | PPWYLSVVGGALYEASSR | diOx Y,O-2H (Y) | 2 | 1996 .95 | -1 .39 | 4769200 | 45 |
| Q8S8Q6 | Tetraspanin-8 | Arabidopsis thaliana | IRSCLVESKVCSK | 2 TriOx (C) | 2 | 1660 .78 | -1 .57 | 2499800 | 49 |
| Q8S8Y3 | ATP synthase subunit alpha, chlorop... | Atropa belladonna | EAIQEQMKR | Acetyl (K),Deam (NQ),di-Ox (M) | 2 | 1206 .56 | -2 .94 | 637870 | 75 |
| Q8S9J2 | Tyrosine--tRNA ligase 1, cytoplasmi... | Arabidopsis thaliana | VINVNKMTSAGCR | TriOx (C),di-Ox (M) | 2 | 1528 .70 | 1 .76 | 515860 | 51 |
| Q8VY05 | SWI/SNF complex subunit SWI3D | Arabidopsis thaliana | DKYNIEKLK | Deam (NQ),Iodination | 2 | 1276 .52 | 4 .47 | 647300 | 49 |
| Q8VYU4 | Carbon catabolite repressor protein... | Arabidopsis thaliana | SLYFHIPRNMLSWGWRK | Deam (NQ),Ox Y | 3 | 2207 .11 | -1 .85 | 1135600 | 63 |
| Q8VZ17 | Histone-lysine N-methyltransferase,... | Arabidopsis thaliana | SKKNLYWR | Deam (NQ),Di-Ox W | 2 | 1126 .58 | -2 .43 | 1252800 | 63 |
| Q8W2F2 | Transcription factor bHLH11 | Arabidopsis thaliana | SDIEILNAQYQHR | 2 Deam (NQ),Ox Y | 2 | 1603 .75 | 0 .03 | 866150 | 64 |
| Q8W4B2 | Protein APEM9 | Arabidopsis thaliana | IWVYKDNHYILNDAGVSTK | Deam (NQ) | 3 | 2236 .12 | -0 .74 | 2604000 | 51 |
| Q8W4H7 | Elongation factor 1-alpha 2 | Arabidopsis thaliana | QTVAVGVIK | Umod | 2 | 913 .56 | 0 .27 | 1074400 | 96 |
| Q8W566 | Uncharacterized exonuclease domain-... | Arabidopsis thaliana | EARGMVSMMRQCGIK | Ox (M),TriOx (C) | 2 | 1816 .81 | -4 .08 | 1147300 | 41 |
| Q8WHZ8 | 50S ribosomal protein L20, chloropl... | Psilotum nudum | ALTYAYRDR | 2 diOx Y | 2 | 1191 .55 | 0 .44 | 630610 | 44 |
| Q8WI26 | DNA-directed RNA polymerase subunit... | Psilotum nudum | MMDRTAIKQLISR | Acetyl (K),Ox (M),di-Ox (M) | 2 | 1651 .84 | -0 .63 | 1785300 | 50 |
| Q8WJP2 | Maturase K | Prunus laurocerasus | KNLGYNK | diOx Y | 2 | 867 .45 | 0 .07 | 1166800 | 65 |
| Q93V43 | Transcription factor TCP2 | Arabidopsis thaliana | LSRWHHNSSR | Di-Ox W | 2 | 1310 .62 | -0 .61 | 3578900 | 89 |
| Q93Y91 | Sugar transport protein 5 | Arabidopsis thaliana | GIPVDSMYQVWEKHWYWQR | Ox (M),2 Deam (NQ),Ox Y,O-2H (Y) | 3 | 2555 .12 | 1 .00 | 2300900 | 71 |
| Q941A4 | Oil body-associated protein 2A | Arabidopsis thaliana | TVVMDKGAAMMQSLK | Acetyl (K),di-Ox (M) | 3 | 1682 .80 | 3 .74 | 2448600 | 63 |
| Q949W6 | Protein KAKU4 | Arabidopsis thaliana | VQGTPLPYSAGNFSSSK | O-2H (Y) | 2 | 1752 .83 | -1 .33 | 2835500 | 40 |
| Q94A08 | Probable galactinol--sucrose galact... | Arabidopsis thaliana | SVVDNAKQR | 2 Deam (NQ) | 2 | 1017 .51 | 0 .01 | 2541000 | 82 |
| Q94AK4 | E3 ubiquitin-protein ligase RZF1 | Arabidopsis thaliana | MSSIRNTHWCHR | Ox (M),Deam (NQ),Trp->Kynurenine | 2 | 1604 .69 | 1 .37 | 952190 | 47 |
| Q94BY4 | O-fucosyltransferase 35 | Arabidopsis thaliana | QMRYEVSHWKEK | Ox (M),Gln->pyro-Glu | 2 | 1618 .76 | 2 .17 | 448990 | 43 |
| Q94C48 | Protein WVD2-like 5 | Arabidopsis thaliana | ETQEAELRMLRK | Glu->pyro-Glu | 2 | 1484 .78 | 2 .62 | 916750 | 60 |
| Q94CG5 | Kinase-interacting protein 1 | Petunia integrifolia | LQNIENLNK | 3 Deam (NQ) | 2 | 1087 .54 | 2 .44 | 428360 | 55 |
| Q94II3 | Probable methyltransferase PMT21 | Arabidopsis thaliana | SNQNWLRKEGEK | 2 Deam (NQ) | 2 | 1489 .72 | 0 .83 | 616480 | 81 |
| Q94KK7 | Syntaxin-52 | Arabidopsis thaliana | QVMREQDEGLEK | Ox (M),Deam (NQ) | 2 | 1477 .67 | -0 .76 | 25790000 | 65 |
| Q96266 | Glutathione S-transferase F8, chlor... | Arabidopsis thaliana | AITQYLAEEYSEKGEK | 2 diOx Y | 2 | 1921 .88 | -1 .10 | 534410 | 49 |
| Q9AST1 | Transcription initiation factor TFI... | Arabidopsis thaliana | GRGGGADGGAPGK | Umod | 2 | 1055 .51 | 2 .54 | 1803500 | 86 |
| Q9ATB4 | Transcriptional adapter ADA2b | Arabidopsis thaliana | IDDKKAEQNMK | 2 Deam (NQ),di-Ox (M) | 2 | 1352 .61 | -1 .77 | 1171900 | 47 |
| Q9AXE3 | S-adenosylmethionine decarboxylase ... | Daucus carota | MSSEVSAIGFEGFEKR | Ox (M) | 2 | 1788 .84 | 1 .11 | 904360 | 67 |
| Q9AXU3 | Senescence-associated protein SPA15... | Ipomoea batatas | MTERITLEKNGK | Ox (M),Deam (NQ) | 2 | 1435 .73 | -0 .66 | 625090 | 54 |
| Q9BBN6 | Protein TIC 214 | Lotus japonicus | IINQSIYNK | 3 Deam (NQ) | 2 | 1094 .55 | 3 .29 | 521180 | 44 |
| Q9C7S5 | Tyrosine-sulfated glycopeptide rece... | Arabidopsis thaliana | ELVAWVHTMK | Glu->pyro-Glu,Di-Ox W | 2 | 1226 .61 | 1 .52 | 662350 | 45 |
| Q9C7V5 | Putative pentatricopeptide repeat-c... | Arabidopsis thaliana | LIIYEFTR | diOx Y | 2 | 1085 .58 | -3 .15 | 3063600 | 100 |
| Q9C866 | Pentatricopeptide repeat-containing... | Arabidopsis thaliana | AVFDSMRDKNVK | Ox (M),Deam (NQ) | 2 | 1425 .69 | -0 .25 | 506520 | 49 |
| Q9C869 | MATH domain and coiled-coil domain-... | Arabidopsis thaliana | MQQLEQNLKDLK | Ox (M) | 2 | 1502 .78 | -2 .67 | 8670500 | 115 |
| Q9C884 | Probable methyltransferase PMT18 | Arabidopsis thaliana | DTVEMLTKIQSITNGMRWK | Ox (M),Deam (NQ) | 3 | 2267 .13 | -3 .82 | 24826000 | 49 |
| Q9C8E6 | Protein PLASTID MOVEMENT IMPAIRED 1 | Arabidopsis thaliana | TVAAVKTMANAMSSGRR | Ox (M) | 2 | 1765 .89 | -4 .12 | 461830 | 42 |
| Q9C8F1 | Probable eukaryotic translation ini... | Arabidopsis thaliana | EQLSAVTAEMVMLSTVEEK | di-Ox (M) | 2 | 2126 .01 | 2 .65 | 1116900 | 82 |
| Q9C952 | Cleavage and polyadenylation specif... | Arabidopsis thaliana | LVIRVDGNVAQLDK | 2 Deam (NQ) | 2 | 1540 .85 | 1 .37 | 1348200 | 82 |
| Q9C9C4 | Enolase 1, chloroplastic | Arabidopsis thaliana | IGMDVAASEFFMKDGR | Ox (M),di-Ox (M) | 2 | 1820 .81 | -0 .85 | 2144200 | 44 |
| Q9C9Q4 | J domain-containing protein require... | Arabidopsis thaliana | STPDIPAMNR | Ox (M),Deam (NQ) | 2 | 1117 .51 | -2 .09 | 494050 | 45 |
| Q9CA93 | Mitochondrial arginine transporter ... | Arabidopsis thaliana | RQGLQGLYR | Deam (NQ),O-2H (Y) | 2 | 1104 .57 | 0 .59 | 85243000 | 44 |
| Q9FFK8 | NF-X1-type zinc finger protein NFXL... | Arabidopsis thaliana | ILMVVMLVAMLAAVSYYGYK | Ox (M),di-Ox (M) | 3 | 2282 .18 | -0 .82 | 6919200 | 45 |
| Q9FG65 | Cytochrome P450 81D1 | Arabidopsis thaliana | SDIEYYTDQIIK | Ox Y,diOx Y | 2 | 1534 .70 | 4 .36 | 1493200 | 80 |
| Q9FGD1 | Protein RKD3 | Arabidopsis thaliana | LRNALELLEMEKK | Deam (NQ),di-Ox (M) | 2 | 1618 .86 | 3 .66 | 527280 | 47 |
| Q9FHJ6 | Replication protein A 70 kDa DNA-bi... | Arabidopsis thaliana | LTHYICNLIQTR | 2 Deam (NQ),diOx Y | 2 | 1564 .76 | -1 .06 | 647680 | 52 |
| Q9FHM4 | 65-kDa microtubule-associated prote... | Arabidopsis thaliana | RLSLGAAMHQTPKPNK | Acetyl (K),Ox (M),2 Deam (NQ) | 2 | 1807 .93 | -2 .99 | 3603700 | 81 |
| Q9FJ25 | GDSL esterase/lipase At5g41890 | Arabidopsis thaliana | VIGENGTKEMLK | Acetyl (K),Ox (M) | 2 | 1375 .70 | -0 .44 | 13708000 | 95 |
| Q9FJN9 | Polyadenylate-binding protein 2 | Arabidopsis thaliana | QLKVLQKR | Acetyl (K),Deam (NQ) | 2 | 1054 .65 | 0 .79 | 1216900 | 104 |
| Q9FJX2 | 60S ribosomal protein L26-2 | Arabidopsis thaliana | NKHNVRSMPIR | Acetyl (K),Ox (M),Deam (NQ) | 2 | 1409 .72 | -2 .15 | 1378300 | 81 |
| Q9FJX8 | Protein DA1-related 6 | Arabidopsis thaliana | GPNNKLVGMATESQKVTR | Acetyl (K),Ox (M),2 Deam (NQ) | 3 | 1988 .98 | -1 .40 | 1105700 | 48 |
| Q9FK76 | Subtilisin-like protease SBT5.6 | Arabidopsis thaliana | PLKNQVMNATEKGQYQFGWFSWTDK | Ox (M),Deam (NQ),O-2H (W),Ox (W) | 3 | 3049 .39 | -2 .21 | 931370 | 56 |
| Q9FKF0 | Ribonuclease 3-like protein 3 | Arabidopsis thaliana | GPKNEPKFVCSVK | Acetyl (K),Deam (NQ),TriOx (C) | 2 | 1579 .76 | 3 .15 | 64725000 | 86 |
| Q9FKG5 | U-box domain-containing protein 51 | Arabidopsis thaliana | LEDALEGGPLQRQQYMK | Ox (M),Deam (NQ),diOx Y | 2 | 2023 .95 | 1 .49 | 1169500 | 46 |
| Q9FKN7 | Protein DA1-related 4 | Arabidopsis thaliana | TPQYSKL | Acetyl (K) | 2 | 877 .45 | -3 .07 | 1086300 | 76 |
| Q9FKR3 | Pentatricopeptide repeat-containing... | Arabidopsis thaliana | GNWKNILKHK | Deam (NQ),Ox (W) | 2 | 1253 .69 | 2 .87 | 3165100 | 48 |
| Q9FKW6 | Ferredoxin--NADP reductase, leaf is... | Arabidopsis thaliana | QLKRSEQWNVEVY | Deam (NQ),Ox Y | 3 | 1694 .83 | -2 .28 | 308380 | 42 |
| Q9FL44 | Protein MAINTENANCE OF PSII UNDER H... | Arabidopsis thaliana | SNVLLSPNGYVFASPKPLGR | Deam (NQ),O-2H (Y) | 2 | 2130 .11 | -3 .95 | 660290 | 53 |
| Q9FLV9 | S-type anion channel SLAH3 | Arabidopsis thaliana | QNSQHRWLDQLR | 2 Deam (NQ),Trp->Kynurenine | 2 | 1585 .76 | 4 .11 | 1483800 | 56 |
| Q9FMD3 | Pentatricopeptide repeat-containing... | Arabidopsis thaliana | VYDALYMFDQMVGMGYK | Deam (NQ),Iodination,Di-iodination | 3 | 2408 .57 | -2 .15 | 11951000 | 47 |
| Q9FMF6 | Pentatricopeptide repeat-containing... | Arabidopsis thaliana | VAANVFYDMLSRK | Ox (M),Deam (NQ) | 2 | 1529 .75 | -3 .08 | 846750 | 48 |
| Q9FNN9 | Putative pentatricopeptide repeat-c... | Arabidopsis thaliana | SVLNMYAKCRR | TriOx (C),di-Ox (M) | 2 | 1476 .68 | 3 .81 | 2255500 | 44 |
| Q9FNY3 | Transcription factor-like protein D... | Arabidopsis thaliana | GLPITCKK | TriOx (C) | 2 | 963 .51 | -2 .80 | 267240 | 70 |
| Q9FQ04 | 5'-3' exoribonuclease 4 | Arabidopsis thaliana | IKQSMDNNEEMKQR | Acetyl (K),2 Deam (NQ),di-Ox (M) | 2 | 1825 .78 | -0 .42 | 5923700 | 60 |
| Q9FQ19 | Sister chromatid cohesion 1 protein... | Arabidopsis thaliana | ALAQYLKQR | 2 Deam (NQ) | 2 | 1091 .60 | 1 .95 | 1243400 | 59 |
| Q9FRV0 | Basic endochitinase C | Secale cereale | GPIQLSHNYNYGPAGR | Deam (NQ),Ox Y,O-2H (Y) | 2 | 1773 .81 | 3 .20 | 1167000 | 43 |
| Q9FSE4 | 30S ribosomal protein S4, chloropla... | Plagiochila adianthoides | STPNKKVSQYR | 2 Deam (NQ),O-2H (Y) | 2 | 1322 .65 | 0 .53 | 2153600 | 74 |
| Q9FV46 | Zeta-carotene desaturase, chloropla... | Tagetes erecta | QIVKADVYIAACDVPGIKR | Gln->pyro-Glu,Ox Y | 2 | 2114 .12 | -2 .04 | 52348000 | 74 |
| Q9FVW4 | Regulator of nonsense transcripts U... | Arabidopsis thaliana | RGSGGGPSSHEKQMWIQK | Ox (M),Deam (NQ),Di-Ox W | 2 | 2017 .93 | 1 .45 | 924920 | 43 |
| Q9FX67 | Transcription factor HRS1 | Arabidopsis thaliana | QRRCWSSQLHR | Deam (NQ),Di-Ox W | 2 | 1545 .72 | -0 .32 | 1020000 | 48 |
| Q9FZ03 | Mannan endo-1,4-beta-mannosidase 2 | Solanum lycopersicum | EASSAGLSVCR | Glu->pyro-Glu | 2 | 1117 .52 | -2 .81 | 2503100 | 63 |
| Q9FZ37 | Putative UDP-glucuronate:xylan alph... | Arabidopsis thaliana | VLFNSGIMVLEPSACMFKDLMEKSFK | 2 Acetyl (K),Ox (M) | 3 | 3120 .50 | 0 .90 | 1025000 | 41 |
| Q9FZD9 | DUF724 domain-containing protein 3 | Arabidopsis thaliana | KPMRSCSAAK | TriOx (C),di-Ox (M) | 2 | 1214 .54 | 2 .11 | 1099800 | 40 |
| Q9GFL3 | 30S ribosomal protein S7, chloropla... | Ginkgo biloba | ARRVGGSTYQVPIEIR | Deam (NQ),diOx Y | 3 | 1833 .97 | -1 .27 | 5570200 | 99 |
| Q9LDM2 | Exosome complex component RRP45A | Arabidopsis thaliana | GLYDYRK | Ox Y,diOx Y | 2 | 961 .45 | -1 .04 | 1190600 | 41 |
| Q9LDN0 | Kinesin-like protein KIN-12A | Arabidopsis thaliana | NDGNNPTNPNVAYSTAWNAR | Deam (NQ),Ox (W) | 2 | 2191 .95 | 3 .42 | 762080 | 44 |
| Q9LF41 | Probable ubiquitin conjugation fact... | Arabidopsis thaliana | TRLFHSQENIVRIDMK | Ox (M),Deam (NQ) | 2 | 2003 .03 | -1 .68 | 492290 | 56 |
| Q9LFH0 | ABC transporter G family member 37 | Arabidopsis thaliana | DQGYDQKK | Umod | 1 | 980 .46 | 1 .35 | 527530 | 63 |
| Q9LFP7 | Serine/threonine-protein kinase PBL... | Arabidopsis thaliana | GLDAVKAKGNWK | Deam (NQ),Trp->Kynurenine | 2 | 1290 .69 | 1 .20 | 737170 | 54 |
| Q9LIN9 | Protein PECTIC ARABINOGALACTAN SYNT... | Arabidopsis thaliana | MAPLRNMFPNLVTKEDLAGK | Deam (NQ),di-Ox (M) | 3 | 2277 .15 | 0 .31 | 1259600 | 49 |
| Q9LIR2 | Putative F-box/FBD/LRR-repeat prote... | Arabidopsis thaliana | LSLDGNDASYLKPWIDAFVK | Deam (NQ) | 3 | 2252 .14 | 2 .73 | 611420 | 44 |
| Q9LK36 | Adenosylhomocysteinase 2 | Arabidopsis thaliana | VAVICGYGDVGKGCAAAMK | Ox (M),Ox Y | 2 | 1957 .91 | -3 .68 | 1226400 | 42 |
| Q9LK38 | Selenium-binding protein 3 | Arabidopsis thaliana | GGPQMFQLSLDGKR | Acetyl (K),2 Deam (NQ) | 2 | 1576 .76 | -1 .50 | 418650 | 45 |
| Q9LK86 | Putative F-box/kelch-repeat protein... | Arabidopsis thaliana | RGQQGEIWGK | Deam (NQ),Trp->Kynurenine | 2 | 1162 .57 | -0 .96 | 2904800 | 75 |
| Q9LKI5 | DNA repair endonuclease UVH1 | Arabidopsis thaliana | LPGVSDANYR | diOx Y | 2 | 1122 .53 | -1 .04 | 2584500 | 101 |
| Q9LM46 | Agamous-like MADS-box protein AGL10... | Arabidopsis thaliana | RPDIQNKECLLR | Acetyl (K),Deam (NQ),TriOx (C) | 2 | 1631 .79 | -2 .25 | 1934600 | 59 |
| Q9LM59 | Serine hydroxymethyltransferase 6 | Arabidopsis thaliana | LQKEPLKSIYHCK | Deam (NQ),diOx Y | 2 | 1675 .86 | 2 .15 | 554880 | 70 |
| Q9LMH5 | Putative pentatricopeptide repeat-c... | Arabidopsis thaliana | SMYGKLIGAWCR | TriOx (C),di-Ox (M) | 2 | 1520 .68 | -0 .06 | 1228100 | 47 |
| Q9LMM2 | Probable ferric reduction oxidase 1 | Arabidopsis thaliana | EIWLTSMR | Ox (M),Trp->Kynurenine | 2 | 1054 .51 | -3 .54 | 2816700 | 45 |
| Q9LPV3 | Putative 60S ribosomal protein L27a... | Arabidopsis thaliana | GNAGVGMR | Ox (M) | 1 | 776 .36 | 0 .51 | 266740 | 43 |
| Q9LQ04 | Bifunctional dTDP-4-dehydrorhamnose... | Arabidopsis thaliana | ESLIKFVFEPNK | Acetyl (K),Glu->pyro-Glu | 2 | 1473 .79 | 4 .33 | 1005200 | 49 |
| Q9LQV2 | RNA-dependent RNA polymerase 1 | Arabidopsis thaliana | MGKTIQVFGFPNGVSAEEVK | Deam (NQ) | 3 | 2138 .07 | 2 .36 | 1944200 | 48 |
| Q9LRM2 | Putative cysteine-rich repeat secre... | Arabidopsis thaliana | FQNCWKSGKR | Deam (NQ),Ox (W) | 2 | 1326 .61 | -3 .49 | 1405500 | 41 |
| Q9LRM4 | B-box domain protein 31 | Arabidopsis thaliana | MCRGLNNEESRR | Deam (NQ),di-Ox (M) | 2 | 1553 .67 | -2 .90 | 1067900 | 53 |
| Q9LTC6 | Ethylene-responsive transcription f... | Arabidopsis thaliana | ERIESYNTNEMK | Ox (M),Deam (NQ),diOx Y | 2 | 1561 .66 | -1 .47 | 510420 | 62 |
| Q9LTQ0 | Ribonuclease 3-like protein 2 | Arabidopsis thaliana | RIDIKNWK | Ox (W) | 2 | 1087 .61 | -0 .02 | 6890200 | 102 |
| Q9LTT9 | Varicose-related protein | Arabidopsis thaliana | GIGKHTSATQQR | 2 Deam (NQ) | 2 | 1284 .64 | 2 .34 | 1542700 | 57 |
| Q9LUC2 | Pentatricopeptide repeat-containing... | Arabidopsis thaliana | YEEVLDVR | Umod | 2 | 1021 .51 | 0 .13 | 3474700 | 101 |
| Q9LUG9 | Mediator of RNA polymerase II trans... | Arabidopsis thaliana | LKVWYRQHQR | Ox Y | 2 | 1428 .77 | -2 .91 | 250650 | 77 |
| Q9LUI8 | Putative F-box/kelch-repeat protein... | Arabidopsis thaliana | KVAVCYNENFEK | Deam (NQ),diOx Y | 2 | 1532 .68 | 3 .31 | 1054700 | 61 |
| Q9LVP9 | Vesicle transport v-SNARE 13 | Arabidopsis thaliana | ILTTMTRRMNR | Deam (NQ),di-Ox (M) | 2 | 1424 .72 | -1 .65 | 5149200 | 64 |
| Q9LXL9 | NAC domain-containing protein 60 | Arabidopsis thaliana | GKKYPHGSQNR | 2 Acetyl (K),2 Deam (NQ) | 2 | 1356 .64 | -2 .01 | 932120 | 67 |
| Q9LZF8 | Serine/threonine-protein kinase PCR... | Arabidopsis thaliana | LWNPKNVRAC | Umod | 1 | 1256 .64 | 4 .32 | 697310 | 42 |
| Q9LZJ8 | Putative two-component response reg... | Arabidopsis thaliana | YMQEELNVQGLTR | 2 Deam (NQ),O-2H (Y) | 2 | 1595 .71 | -4 .16 | 2840100 | 63 |
| Q9LZM7 | PRA1 family protein A1 | Arabidopsis thaliana | EVDWSSPPR | Glu->pyro-Glu,Di-Ox W | 2 | 1085 .48 | 1 .80 | 303020 | 46 |
| Q9M008 | Cation/H(+) antiporter 26 | Arabidopsis thaliana | IGSFCYAVLMPCYVIGIGNK | Deam (NQ),2 O-2H (Y) | 2 | 2290 .05 | 4 .47 | 637290 | 44 |
| Q9M066 | 3-epi-6-deoxocathasterone 23-monoox... | Arabidopsis thaliana | RKLELGEEYK | Ox Y | 2 | 1279 .68 | -0 .92 | 2183300 | 84 |
| Q9M0B9 | Transcription factor IBH1-like 1 | Arabidopsis thaliana | QPTSSMNEEFLKK | Ox (M),Deam (NQ) | 2 | 1554 .72 | 2 .33 | 521360 | 44 |
| Q9M0U9 | F-box protein SKIP19 | Arabidopsis thaliana | LGAIEILENAQK | Umod | 2 | 1297 .72 | 2 .40 | 1017200 | 101 |
| Q9M175 | Protein NRT1/ PTR FAMILY 2.3 | Arabidopsis thaliana | NLKDNDQEQDPK | Acetyl (K),3 Deam (NQ) | 2 | 1487 .63 | 1 .14 | 657000 | 43 |
| Q9M2I2 | MATH domain and coiled-coil domain-... | Arabidopsis thaliana | AVDKKFCWEIK | TriOx (C) | 2 | 1470 .72 | -4 .00 | 1927100 | 77 |
| Q9M2W2 | Glutathione S-transferase L2, chlor... | Arabidopsis thaliana | FRLILSDVMNVDITSGR | Deam (NQ) | 3 | 1936 .01 | 1 .55 | 1298600 | 46 |
| Q9M358 | UV-stimulated scaffold protein A ho... | Arabidopsis thaliana | VKGTNPQQLAQGNDEKCR | Deam (NQ),TriOx (C) | 2 | 2090 .97 | -0 .22 | 7865100 | 55 |
| Q9M8L3 | VQ motif-containing protein 11 | Arabidopsis thaliana | QSSKKMELK | Ox (M),Gln->pyro-Glu | 2 | 1076 .55 | -1 .26 | 1200900 | 46 |
| Q9M9B3 | Zinc finger protein CONSTANS-LIKE 8 | Arabidopsis thaliana | DVKNNTSSFQLVPPGIEEK | Acetyl (K),3 Deam (NQ) | 2 | 2146 .03 | -0 .33 | 1240800 | 43 |
| Q9M9T4 | Protein PHYTOCHROME KINASE SUBSTRAT... | Arabidopsis thaliana | QSSKNSSETPSLR | Gln->pyro-Glu | 2 | 1402 .67 | 4 .42 | 1094300 | 49 |
| Q9MA15 | Protein ACTIVITY OF BC1 COMPLEX KIN... | Arabidopsis thaliana | QGNLEKNMKK | Ox (M),2 Deam (NQ) | 2 | 1206 .59 | -3 .50 | 4572900 | 70 |
| Q9MUR4 | Pyruvate dehydrogenase E1 component... | Mesostigma viride | GPGGVGKQLGAEHSQR | 2 Deam (NQ) | 2 | 1578 .78 | -0 .92 | 58068000 | 59 |
| Q9S713 | Serine/threonine-protein kinase STN... | Arabidopsis thaliana | QGLLALSVMQNLRMQYFR | 2 Deam (NQ) | 3 | 2169 .11 | -1 .83 | 1191400 | 57 |
| Q9SA77 | UDP-arabinose 4-epimerase 1 | Arabidopsis thaliana | ISGACFDAARGIMPGLQIK | Umod | 2 | 2004 .03 | -2 .05 | 441300 | 48 |
| Q9SAH2 | Pentatricopeptide repeat-containing... | Arabidopsis thaliana | QPENALKIWAEMDR | 2 Deam (NQ),O-2H (W) | 2 | 1715 .78 | 2 .22 | 869020 | 43 |
| Q9SB68 | Manganese-dependent ADP-ribose/CDP-... | Arabidopsis thaliana | AVETWNQHGNLK | 2 Deam (NQ),Di-Ox W | 2 | 1429 .65 | 0 .18 | 1264900 | 48 |
| Q9SCU8 | Beta-galactosidase 14 | Arabidopsis thaliana | STPHMWPSIIDK | Ox (M),Di-Ox W | 2 | 1458 .68 | 2 .27 | 2,48E+08 | 57 |
| Q9SFU6 | Callose synthase 9 | Arabidopsis thaliana | GMMYYRKALMLQSYLER | 2 Ox (M) | 2 | 2184 .05 | -3 .00 | 1094100 | 43 |
| Q9SFW5 | Probable acyl-activating enzyme 21 | Arabidopsis thaliana | LGNKNMKMIPK | Acetyl (K),Deam (NQ),2 di-Ox (M) | 2 | 1379 .68 | -2 .85 | 3937000 | 61 |
| Q9SFX0 | Probable serine/threonine-protein k... | Arabidopsis thaliana | PNGEQYLVAWAR | 2 Deam (NQ),diOx Y | 2 | 1436 .66 | 0 .83 | 420840 | 45 |
| Q9SGH2 | Methyl-CpG-binding domain-containin... | Arabidopsis thaliana | GVAAADEDKVLCTLLGRK | Umod | 3 | 1915 .02 | -1 .02 | 4491300 | 44 |
| Q9SHG5 | Cytochrome P450 72C1 | Arabidopsis thaliana | AVNWVWLRPK | Deam (NQ),O-2H (W) | 2 | 1282 .68 | -2 .98 | 704190 | 71 |
| Q9SHL7 | Exosome complex exonuclease RRP44 h... | Arabidopsis thaliana | NMNTLAKIMRQR | Acetyl (K),Ox (M),2 Deam (NQ) | 2 | 1534 .76 | 1 .32 | 701950 | 62 |
| Q9SI19 | Protein SHI RELATED SEQUENCE 4 | Arabidopsis thaliana | GILYNQGPENKSMR | Acetyl (K),di-Ox (M) | 3 | 1679 .79 | 2 .89 | 1230400 | 44 |
| Q9SJA4 | Probable cyclic nucleotide-gated io... | Arabidopsis thaliana | IGRSSCMTTDLK | TriOx (C) | 2 | 1415 .64 | 3 .89 | 869700 | 75 |
| Q9SJG8 | Protein MEI2-like 2 | Arabidopsis thaliana | PNTAGRVSVEHPNGEHPSR | Deam (NQ) | 3 | 2040 .97 | -4 .06 | 1254700 | 49 |
| Q9SK09 | Vicilin-like seed storage protein A... | Arabidopsis thaliana | KDKPSFDNK | Acetyl (K) | 2 | 1119 .56 | 1 .02 | 5863700 | 86 |
| Q9SK74 | Zinc finger CCCH domain-containing ... | Arabidopsis thaliana | AKLQTATQMTPALFMEWK | Acetyl (K),Ox (M) | 3 | 2152 .07 | -4 .10 | 864970 | 41 |
| Q9SKB3 | Poly(ADP-ribose) glycohydrolase 1 | Arabidopsis thaliana | IVAIDALCTPKMR | TriOx (C),di-Ox (M) | 2 | 1566 .77 | 2 .30 | 2473700 | 55 |
| Q9SLF3 | Translocase of chloroplast 132, chl... | Arabidopsis thaliana | KDANVQLEMASSVK | Acetyl (K),2 Deam (NQ) | 2 | 1562 .75 | -0 .28 | 669170 | 67 |
| Q9SLK0 | Peroxisomal isocitrate dehydrogenas... | Arabidopsis thaliana | LIDDMVAYAMK | Ox (M),di-Ox (M) | 2 | 1316 .60 | 1 .80 | 506070 | 43 |
| Q9SLN8 | 2-alkenal reductase (NADP(+)-depend... | Nicotiana tabacum | AEEVSNKQVILK | 2 Deam (NQ) | 2 | 1358 .73 | -1 .40 | 2279000 | 86 |
| Q9SM50 | Transcription factor HY5 | Solanum lycopersicum | VSAQQARERK | 2 Deam (NQ) | 2 | 1173 .61 | 3 .36 | 1309500 | 93 |
| Q9SR36 | Glutathione S-transferase U8 | Arabidopsis thaliana | LKGIPYEYIEEDVYGNR | 2 O-2H (Y) | 2 | 2084 .97 | 2 .39 | 1014000 | 42 |
| Q9SSE9 | Lysine-specific demethylase JMJ25 | Arabidopsis thaliana | MGWPLVLKLK | Ox (M) | 2 | 1199 .71 | 1 .25 | 5524600 | 71 |
| Q9ST62 | External alternative NAD(P)H-ubiqui... | Solanum tuberosum | DFMEQVGQEK | Ox (M),Deam (NQ) | 3 | 1226 .51 | 3 .72 | 764520 | 52 |
| Q9STM3 | Lysine-specific demethylase REF6 | Arabidopsis thaliana | SPLYSKQMPYNSIIYK | Deam (NQ),O-2H (Y) | 2 | 1945 .95 | -0 .12 | 412810 | 61 |
| Q9STV0 | Alpha-glucan water dikinase 2 | Arabidopsis thaliana | IYLQQPNKR | Deam (NQ),O-2H (Y) | 2 | 1173 .61 | 2 .22 | 1769000 | 59 |
| Q9STW5 | MACPF domain-containing protein At4... | Arabidopsis thaliana | GPQESPGYWVVSGAR | Deam (NQ),Trp->Kynurenine | 2 | 1593 .74 | -2 .56 | 111090 | 42 |
| Q9SV12 | Heat stress transcription factor A-... | Arabidopsis thaliana | QRQMMSFLAR | Ox (M),Deam (NQ) | 2 | 1283 .61 | 3 .33 | 719420 | 49 |
| Q9SVL0 | Zinc-finger homeodomain protein 7 | Arabidopsis thaliana | NQNVDGKSLMMMMMRK | Ox (M),2 Deam (NQ) | 3 | 1930 .84 | 1 .51 | 5840000 | 42 |
| Q9SW44 | DEAD-box ATP-dependent RNA helicase... | Arabidopsis thaliana | AVQLTSSMSASDMR | Ox (M),di-Ox (M) | 2 | 1530 .67 | 3 .19 | 2736200 | 47 |
| Q9SWA6 | Negative regulator of systemic acqu... | Arabidopsis thaliana | ELLVMIMELDTSKK | Ox (M) | 2 | 1664 .87 | 2 .69 | 715590 | 64 |
| Q9SX79 | Polyadenylate-binding protein RBP47... | Arabidopsis thaliana | TSPTPPPHWMR | Di-Ox W | 2 | 1337 .62 | 1 .15 | 2603200 | 70 |
| Q9SXY1 | Chromatin assembly factor 1 subunit... | Arabidopsis thaliana | LWLINSGQAEKK | Deam (NQ),Ox (W) | 2 | 1402 .75 | 0 .38 | 1217300 | 70 |
| Q9SY07 | Pentatricopeptide repeat-containing... | Arabidopsis thaliana | AGEMALIVEERMAK | Ox (M) | 2 | 1562 .78 | -2 .22 | 882670 | 92 |
| Q9SY91 | Probable serine/threonine-protein k... | Arabidopsis thaliana | RLRCVMDPR | Ox (M),TriOx (C) | 2 | 1265 .60 | 4 .15 | 334640 | 47 |
| Q9SYD0 | Putative F-box only protein 10 | Arabidopsis thaliana | SRPQFMFR | Ox (M),Deam (NQ) | 2 | 1084 .51 | 3 .26 | 684580 | 41 |
| Q9SYP1 | DExH-box ATP-dependent RNA helicase... | Arabidopsis thaliana | QNIGGNLAMDQR | Ox (M),Gln->pyro-Glu | 2 | 1314 .60 | -2 .83 | 839460 | 56 |
| Q9SZL6 | Transcription termination factor MT... | Arabidopsis thaliana | ILKPNYDYLK | 2 O-2H (Y) | 2 | 1293 .66 | -2 .56 | 2492100 | 82 |
| Q9SZL7 | Protein FAR1-RELATED SEQUENCE 9 | Arabidopsis thaliana | QAASLYTR | Gln->pyro-Glu,Ox Y | 2 | 907 .44 | 1 .12 | 280420 | 43 |
| Q9T043 | 60S ribosomal protein L14-2 | Arabidopsis thaliana | IQMNFKR | Ox (M) | 2 | 951 .50 | -1 .41 | 625500 | 71 |
| Q9XIN8 | F-box protein At2g27310 | Arabidopsis thaliana | ERYEEYVR | 2 diOx Y | 2 | 1206 .52 | 0 .70 | 227260 | 49 |
| Q9ZPP1 | Calreticulin | Berberis stolonifera | WNGDANDK | 2 Deam (NQ),O-2H (W) | 2 | 934 .33 | 0 .68 | 8045100 | 54 |
| Q9ZQV9 | Nicotianamine synthase 1 | Hordeum vulgare | LSPEHQRMR | di-Ox (M) | 2 | 1184 .57 | -2 .72 | 17507000 | 84 |
| Q9ZST1 | 30S ribosomal protein S17, chloropl... | Oryza sativa subsp. japonica | VQGNGGSGASPWAGAATALRIQAAK | 2 Deam (NQ) | 4 | 2340 .18 | -2 .16 | 1085700 | 45 |
| Q9ZUM9 | Histone-lysine N-methyltransferase ... | Arabidopsis thaliana | SVRAYGIYPK | 2 diOx Y | 2 | 1216 .61 | 0 .08 | 1110200 | 71 |
| Q9ZUU3 | Pentatricopeptide repeat-containing... | Arabidopsis thaliana | KLEHALQFFRWTER | Deam (NQ),Trp->Kynurenine | 3 | 1864 .96 | -2 .26 | 3031400 | 63 |
| W8JIS5 | Iridoid oxidase | Catharanthus roseus | KRMVDNMSR | 2 Ox (M),Deam (NQ) | 2 | 1168 .53 | 2 .15 | 1992200 | 65 |
| W8JMV1 | Cytochrome P450 76T24 | Catharanthus roseus | NSQILINVWASGR | 2 Deam (NQ),Di-Ox W | 2 | 1490 .74 | 0 .83 | 3085800 | 67 |

Table S4 List of peptides and proteins of Viridiplantae in trunk tip sample: for each peptide all the features (same sequence and different modifications) with the highest intensity are reported.

| Acc. Number | Description | Specie | Peptide Sequence | Modifications | Charge | Mass | Mass error [ppm] | Intensity | Score |
| --- | --- | --- | --- | --- | --- | --- | --- | --- | --- |
| F4HU58 | tRNA-specific adenosine deaminase TAD1 | Arabidopsis thaliana | ATEYYLMSKIFK | 2 O-2H (Y) | 2 | 1520 .72 | 0 .36 | 1055400 | 59 |
|  |  |  | GNGSQVADMVQRK | Deam (NQ),di-Ox (M) | 2 | 1421 .66 | 3 .79 | 106680 | 48 |
| O48653 | DNA polymerase alpha catalytic subunit | Oryza sativa subsp. japonica | FHSCCVVVKNMQR | Deam (NQ),TriOx (C) | 2 | 1712 .74 | 4 .09 | 752470 | 52 |
|  |  |  | VPSNMWSKIGR | Deam (NQ),Trp->Kynurenine | 2 | 1278 .64 | -2 .43 | 878790 | 71 |
| O65316 | Actin | Mesostigma viride | SYELPDGQVITIGNER | Unmodified | 2 | 1789 .88 | 1 .53 | 595330 | 80 |
|  |  |  | VAPEEHPVLLTEAPLNPK | Unmodified | 3 | 1953 .06 | 2 .07 | 1390400 | 61 |
| P08477 | Glyceraldehyde-3-phosphate dehydrogenase 2 | Hordeum vulgare | AASFNIIPSSTGAAK | Unmodified | 2 | 1433 .75 | 0 .45 | 609380 | 84 |
|  |  |  | VLPELNGK | Unmodified | 2 | 868 .50 | -3 .22 | 628540 | 106 |
|  |  |  | VPTVDVSVVDLTVR | Unmodified | 2 | 1497 .84 | 2 .16 | 577350 | 94 |
| Q01781 | Adenosylhomocysteinase | Petroselinum crispum | LVGVSEETTTGVK | Unmodified | 2 | 1318 .70 | 0 .66 | 190710 | 84 |
|  |  |  | LVGVSEETTTGVKR | Unmodified | 3 | 1474 .80 | -1 .49 | 156470 | 98 |
| Q70XV6 | Protein TIC 214 | Amborella trichopoda | DSISNNKTISESPIR | 2 Deam (NQ) | 3 | 1661 .81 | -3 .11 | 160930 | 44 |
|  |  |  | IQNCKPFLDRGK | Unmodified | 3 | 1474 .77 | 4 .35 | 262770 | 47 |
| Q8MA10 | DNA-directed RNA polymerase subunit beta | Chaetosphaeridium globosum | DLSLDINTNLLK | Deam (NQ) | 2 | 1358 .73 | -1 .97 | 3173200 | 107 |
|  |  |  | DLSLDINTNLLK | Unmodified | 2 | 1357 .75 | -1 .67 | 1144100 | 97 |
| Q9FNA4 | Elongator complex protein 1 | Arabidopsis thaliana | EMELLNSSDDIRK | Ox (M),Glu->pyro-Glu | 2 | 1546 .73 | -3 .39 | 7614200 | 62 |
|  |  |  | EMELLNSSDDIRK | Glu->pyro-Glu | 2 | 1530 .73 | -1 .69 | 1337900 | 63 |
| F2Z9C1 | Protopine 6-monooxygenase | Eschscholzia californica | AFQLMTYDNESVAFTPYGSYWR | Ox (M) | 4 | 2661 .18 | -3 .36 | 5037600 | 43 |
|  |  |  | AFQLMTYDNESVAFTPYGSYWR | Unmodified | 4 | 2645 .19 | -4 .39 | 1137500 | 49 |
| A0A024B4E4 | Major strawberry allergen Fra a 1.0... | Fragaria ananassa | IIENYLLGNPDAYN | Deam (NQ),O-2H (Y) | 2 | 1622 .75 | -2 .34 | 1432200 | 76 |
| A0A0P0WFC8 | Transcription factor TGAL6 | Oryza sativa subsp. japonica | LAQLEQELQR | Unmodified | 2 | 1226 .66 | 0 .65 | 2609800 | 136 |
| A0A142ZC57 | Lycopaoctaene synthase | Botryococcus braunii | EIWGNYAKQLDEFKDPK | 2 Deam (NQ) | 3 | 2081 .99 | 1 .26 | 1254900 | 50 |
| A0A222NNM9 | Cocosin 1 | Cocos nucifera | SINGFEETYCSMKIK | Ox (M),Ox Y | 2 | 1837 .82 | -1 .82 | 503680 | 48 |
| A0A291LSD6 | Beta-selinene synthase | Zea mays | LLLQYYLK | Deam (NQ),Ox Y,O-2H (Y) | 2 | 1083 .59 | -2 .45 | 197530 | 86 |
| A0A2I7G3B0 | Aldehyde dehydrogenase 1 | Tanacetum cinerariifolium | ANATTYGLAAGIMTK | Ox (M),Deam (NQ) | 3 | 1498 .73 | 1 .45 | 3608600 | 50 |
| A0A2U1KZS6 | NADPH--cytochrome P450 reductase 2 | Artemisia annua | EYVQHKLTQK | Glu->pyro-Glu,O-2H (Y) | 2 | 1268 .65 | 0 .92 | 1970600 | 74 |
| A0A3Q8GYY4 | (+)-cis,trans-nepetalactol synthase... | Nepeta racemosa | GTSACVKQAAR | TriOx (C) | 2 | 1195 .56 | -3 .45 | 4211700 | 73 |
| A1E9I3 | DNA-directed RNA polymerase subunit... | Hordeum vulgare | YDRTSGGPTPHNRLDR | Deam (NQ),O-2H (Y) | 2 | 1855 .86 | -0 .31 | 1236700 | 52 |
| A1XGT0 | Protein Ycf2 | Ranunculus macranthus | WVQISCGNALEDPK | Trp->Kynurenine | 2 | 1619 .76 | 0 .80 | 483170 | 67 |
| A1XGT3 | Protein TIC 214 | Ranunculus macranthus | GTIKELYSRSSMNEYLIPSIEDPR | Deam (NQ),diOx Y | 3 | 2830 .37 | -4 .49 | 414320 | 40 |
| A2RVU1 | Protein MODIFYING WALL LIGNIN-1 | Arabidopsis thaliana | PSLQVENHDKR | Deam (NQ) | 2 | 1322 .66 | 0 .91 | 7356400 | 75 |
| A2T317 | ATP synthase subunit alpha, chlorop... | Angiopteris evecta | KEIEGYTQEVK | Deam (NQ),Ox Y | 2 | 1339 .65 | 1 .57 | 1603500 | 91 |
| A3BN26 | RNA pseudouridine synthase 6, chlor... | Oryza sativa subsp. japonica | EAQKTFRVTDPNQR | Glu->pyro-Glu | 3 | 1670 .85 | 4 .19 | 555240 | 54 |
| A3BXL8 | ABC transporter G family member 53 | Oryza sativa subsp. japonica | ETVNFSAKCQGIGHR | Acetyl (K),Glu->pyro-Glu | 2 | 1726 .82 | -3 .30 | 2796000 | 78 |
| A3EWL3 | DNA repair protein REV1 | Arabidopsis thaliana | VSGNCTMEK | Ox (M),TriOx (C) | 2 | 1088 .41 | 1 .55 | 245930 | 49 |
| A4GG84 | 30S ribosomal protein S3, chloropla... | Phaseolus vulgaris | IEEFQTNMHKK | Ox (M),Deam (NQ) | 2 | 1420 .67 | -4 .08 | 619800 | 44 |
| A4VCM0 | NAC domain-containing protein 45 | Arabidopsis thaliana | HMNQTGYIKEQK | Ox (M),Deam (NQ) | 2 | 1492 .70 | -3 .82 | 784900 | 41 |
| A6BM11 | ATP synthase subunit b, chloroplast... | Gnetum parvifolium | ANGYLQIEQEK | 2 Deam (NQ) | 2 | 1293 .61 | 0 .99 | 171990 | 63 |
| A7M944 | Protein TIC 214 | Cuscuta gronovii | QQNQTTTKMNTETKNK | Deam (NQ) | 2 | 1894 .91 | 2 .48 | 320770 | 52 |
| A8JB22 | Dynein regulatory complex subunit 2 | Chlamydomonas reinhardtii | EWEERNRALR | Glu->pyro-Glu,Deam (NQ) | 2 | 1340 .66 | 4 .15 | 2678500 | 100 |
| A8W3H9 | Plastid 30S ribosomal protein S2 | Cuscuta obtusiflora | MEQKKGGLNNLPK | Deam (NQ) | 2 | 1456 .77 | -1 .39 | 5661800 | 84 |
| A9RWC9 | Mitogen-activated protein kinase ki... | Physcomitrium patens | WTNETYALKVIHMNIEETTR | O-2H (W) | 3 | 2462 .19 | -2 .93 | 267570 | 48 |
| B1A948 | Cytochrome f | Carica papaya | GRGQIYPDGRK | Deam (NQ),diOx Y | 2 | 1278 .63 | 2 .30 | 878790 | 78 |
| B1NF18 | Salutaridine synthase | Papaver somniferum | ISGLASLADAFKIAKYLPSQR | Deam (NQ) | 3 | 2249 .24 | -0 .63 | 1304300 | 47 |
| B2X1Z4 | DNA-directed RNA polymerase subunit... | Oedogonium cardiacum | EIVVNFNYLKK | Ox Y | 3 | 1381 .76 | -2 .36 | 9169300 | 60 |
| B2Y1V7 | DNA-directed RNA polymerase subunit... | Welwitschia mirabilis | VEQLAEAQSNNPVVQNIEENFR | 3 Deam (NQ) | 3 | 2530 .18 | 0 .10 | 334290 | 46 |
| B3EWR6 | Antimicrobial peptides | Echinochloa crus-galli | EMQRRCAR | Ox (M),Deam (NQ) | 2 | 1122 .50 | -3 .80 | 149640 | 53 |
| B3TN96 | NAD(P)H-quinone oxidoreductase subu... | Brachypodium distachyon | SQNMALMGGLRK | Ox (M) | 2 | 1320 .66 | 2 .40 | 3912500 | 61 |
| B4FR29 | Rubisco accumulation factor 1, chlo... | Zea mays | GEEGWEAFSK | O-2H (W) | 2 | 1152 .47 | 1 .42 | 3345800 | 72 |
| B8ARW2 | Transportin-1 | Oryza sativa subsp. indica | NVTELMLQANK | Ox (M),Deam (NQ) | 2 | 1276 .63 | -4 .31 | 409760 | 68 |
| B9DHT4 | ARM REPEAT PROTEIN INTERACTING WITH... | Arabidopsis thaliana | PLIEMLQSPDVQLK | Ox (M) | 3 | 1625 .87 | 4 .16 | 589320 | 57 |
| C0LGI2 | Probable LRR receptor-like serine/t... | Arabidopsis thaliana | WATVTIQEVSR | Deam (NQ),Di-Ox W | 2 | 1321 .65 | 1 .14 | 2898300 | 79 |
| D3GE74 | ABC transporter G family member STR | Medicago truncatula | TPGWTPGK | Trp->Kynurenine | 1 | 846 .42 | 1 .01 | 241130 | 46 |
| F4HQ05 | Protein DETOXIFICATION 8 | Arabidopsis thaliana | VIEMIPQEII | Ox (M),Deam (NQ) | 2 | 1200 .63 | 1 .77 | 846240 | 60 |
| F4I1T9 | Kinesin-like protein KIN-14T | Arabidopsis thaliana | KEAVMMNLQKMMEK | Ox (M),Deam (NQ) | 2 | 1726 .81 | -0 .68 | 1819500 | 53 |
| F4I893 | Protein ILITYHIA | Arabidopsis thaliana | RSSAYLIGYFFK | diOx Y | 2 | 1482 .75 | -1 .99 | 1329700 | 68 |
| F4I8I0 | SUN domain-containing protein 4 | Arabidopsis thaliana | EAMEMRKWR | Ox (M),Di-Ox W | 2 | 1283 .58 | -2 .86 | 723910 | 42 |
| F4IIZ9 | Stomatal closure-related actin-bind... | Arabidopsis thaliana | LQREIAIIKK | Unmodified | 1 | 1210 .78 | -1 .33 | 3658000 | 48 |
| F4IJV4 | Probable pre-mRNA-splicing factor A... | Arabidopsis thaliana | FSWQEYMKK | Deam (NQ),di-Ox (M) | 2 | 1278 .56 | 3 .58 | 239380 | 45 |
| F4IPE3 | Zinc finger protein SHOOT GRAVITROP... | Arabidopsis thaliana | QREIAENEFANAK | Unmodified | 3 | 1518 .74 | 0 .00 | 3235000 | 94 |
| F4J1H7 | tRNAse Z TRZ4, mitochondrial | Arabidopsis thaliana | KNIAGDEIPGWK | O-2H (W) | 2 | 1340 .67 | -1 .86 | 44173000 | 63 |
| F4J2M6 | Kinesin-like protein KIN-14L | Arabidopsis thaliana | EQIENLKR | Deam (NQ) | 2 | 1029 .55 | -0 .26 | 1447100 | 97 |
| F4J394 | Kinesin-like protein KIN-7G | Arabidopsis thaliana | EKDLQIEKLNK | Glu->pyro-Glu,2 Deam (NQ) | 2 | 1340 .72 | -2 .07 | 780090 | 85 |
| F4J8K6 | rRNA biogenesis protein RRP5 | Arabidopsis thaliana | PCHTDIKK | Acetyl (K),TriOx (C) | 2 | 1087 .50 | 0 .59 | 344030 | 59 |
| F4JRF4 | Adenine DNA glycosylase | Arabidopsis thaliana | RDLPWRNR | Deam (NQ),Trp->Kynurenine | 2 | 1116 .58 | -1 .74 | 2967900 | 53 |
| F4JRS4 | Protein MICRORCHIDIA 7 | Arabidopsis thaliana | LEEASNTIDDLLNKIKK | Acetyl (K),Deam (NQ) | 3 | 1986 .05 | 0 .89 | 608120 | 65 |
| F4JTJ9 | Meiosis-specific protein ASY2 | Arabidopsis thaliana | FSVYISFHIANYR | Iodination,O-2H (Y) | 2 | 1755 .69 | 0 .02 | 64048000 | 59 |
| F4K5T4 | Probable transcription factor At5g2... | Arabidopsis thaliana | LELVQEQIR | 2 Deam (NQ) | 2 | 1128 .60 | 0 .67 | 2301600 | 126 |
| F4KD38 | DNA-directed RNA polymerase III sub... | Arabidopsis thaliana | LELSGQLISLLFEDLFK | Deam (NQ) | 3 | 1965 .07 | -2 .13 | 180320 | 81 |
| F4KF14 | Probable inactive ATP-dependent zin... | Arabidopsis thaliana | HIVDRMPIDGWNDVWK | Deam (NQ) | 3 | 1980 .95 | 2 .54 | 152720 | 42 |
| F4KFX5 | AAA-ATPase At5g40000 | Arabidopsis thaliana | ARTLITNSYIK | Deam (NQ),diOx Y | 2 | 1311 .70 | 0 .12 | 208940 | 81 |
| F4KGU4 | ATP-dependent RNA helicase DEAH12, ... | Arabidopsis thaliana | YNSRSAQSSPPLNHR | O-2H (Y) | 2 | 1726 .81 | -1 .02 | 2040100 | 69 |
| F8WQD0 | Shionone synthase | Aster tataricus | NIYTLWALAQYRK | Deam (NQ),Iodination | 2 | 1765 .77 | 3 .14 | 2065500 | 59 |
| I1L153 | Allantoate deiminase 2 | Glycine max | RSVSCIIEHK | TriOx (C) | 2 | 1275 .62 | 1 .00 | 207970 | 58 |
| O04379 | Protein argonaute 1 | Arabidopsis thaliana | QVQDNLAR | 3 Deam (NQ) | 2 | 945 .44 | -1 .03 | 188180 | 47 |
| O04532 | Formin-like protein 8 | Arabidopsis thaliana | RTTDYYQAGAVTK | Ox Y,O-2H (Y) | 2 | 1502 .70 | 2 .13 | 892830 | 65 |
| O04933 | Probable sucrose-phosphate synthase... | Craterostigma plantagineum | GVVEKGSEELLR | Unmodified | 2 | 1314 .71 | 2 .53 | 188010 | 67 |
| O22208 | bZIP transcription factor 17 | Arabidopsis thaliana | AANGEMQQWFR | Ox (M),3 Deam (NQ) | 2 | 1355 .55 | 1 .66 | 2264000 | 40 |
| O22267 | Histidine kinase CKI1 | Arabidopsis thaliana | ATLINQMEATQQAER | Ox (M),2 Deam (NQ) | 2 | 1720 .79 | 0 .22 | 1916600 | 51 |
| O23372 | Histone-lysine N-methyltransferase ... | Arabidopsis thaliana | TMKMCQGVLK | 2 Ox (M) | 2 | 1226 .58 | 2 .51 | 1257600 | 71 |
| O23736 | Glutamate--cysteine ligase, chlorop... | Brassica juncea | RYMEMRGADGGPWR | Ox (M),Di-Ox W | 2 | 1728 .75 | 0 .19 | 1092600 | 45 |
| O24600 | DNA-directed RNA polymerase 3, chlo... | Arabidopsis thaliana | EVLKSQYFFN | 2 Deam (NQ),O-2H (Y) | 2 | 1289 .58 | 2 .66 | 1202800 | 70 |
| O47027 | 30S ribosomal protein S2, chloropla... | Chlamydomonas reinhardtii | PNLGAKLGDK | Deam (NQ) | 1 | 1012 .56 | 1 .58 | 478020 | 43 |
| O49285 | ACT domain-containing protein ACR3 | Arabidopsis thaliana | ITVEHCEEKGYSVINVSCEDR | TriOx (C) | 3 | 2571 .12 | -1 .57 | 33352000 | 65 |
| O80877 | Protein ELF4-LIKE 1 | Arabidopsis thaliana | AQLYLDQNR | 2 Deam (NQ),Iodination | 2 | 1247 .43 | -0 .01 | 128910 | 44 |
| O81016 | ABC transporter G family member 32 | Arabidopsis thaliana | NSNLCQR | 2 Deam (NQ),TriOx (C) | 2 | 940 .36 | 0 .28 | 200240 | 42 |
| O81776 | Glutamate receptor 2.4 | Arabidopsis thaliana | SQVPIISFSATSPFLDSGRSPYFFR | Iodination | 3 | 2931 .31 | -2 .54 | 10034000 | 42 |
| O82144 | Chalcone synthase | Hydrangea macrophylla | KLALKPEK | Unmodified | 1 | 925 .60 | 1 .60 | 38710000 | 52 |
| O82190 | Probable RNA-dependent RNA polymera... | Arabidopsis thaliana | KVFNKPYLK | Deam (NQ),diOx Y | 2 | 1168 .65 | -4 .27 | 904820 | 74 |
| O82484 | Putative disease resistance protein... | Arabidopsis thaliana | LQQVQVWLK | Deam (NQ),O-2H (W) | 2 | 1155 .63 | -1 .99 | 310890 | 59 |
| P08770 | Putative AC transposase | Zea mays | FSTTMDMWTSCQNK | Ox (M),Trp->Kynurenine | 2 | 1755 .69 | -0 .89 | 560760 | 43 |
| P0C8A0 | Pentatricopeptide repeat-containing... | Arabidopsis thaliana | WGMIDKGYSVLDDMRK | Ox (M) | 3 | 1928 .91 | -2 .11 | 1483300 | 45 |
| P0CV94 | (3S,6E)-nerolidol synthase 1 | Fragaria ananassa | ALYDMTNEFSSKVYLK | Ox (M) | 3 | 1923 .93 | 3 .64 | 1198600 | 49 |
| P12221 | Putative protein TIC 214 N-terminal... | Marchantia polymorpha | SNLNWELILNLSPR | 3 Deam (NQ) | 2 | 1670 .85 | 4 .15 | 959020 | 45 |
| P13911 | DNA-directed RNA polymerase subunit... | Pisum sativum | VDSKRLYYGR | Iodination,Ox Y | 2 | 1397 .56 | -0 .12 | 1871100 | 64 |
| P13934 | Late embryogenesis abundant protein... | Brassica napus | DKTSQTAQKAQQK | 3 Deam (NQ) | 2 | 1463 .71 | -2 .77 | 127940 | 48 |
| P16347 | Endogenous alpha-amylase/subtilisin... | Triticum aestivum | IEKYSGAEVHEYK | Iodination,O-2H (Y) | 2 | 1691 .63 | -2 .39 | 581040 | 46 |
| P17569 | Nitrate reductase [NADH] | Cucurbita maxima | WADWTVEVCGLVKR | Unmodified | 3 | 1717 .86 | -1 .66 | 4807400 | 75 |
| P17597 | Acetolactate synthase, chloroplasti... | Arabidopsis thaliana | PGPVLVDVPKDIQQQLAIPNWEQAMR | Ox (M),2 Deam (NQ) | 3 | 2959 .51 | -2 .42 | 2073500 | 40 |
| P21746 | Embryonic abundant protein USP87 | Vicia faba | TFSDMLIPSGK | di-Ox (M) | 2 | 1226 .59 | 0 .16 | 1257600 | 84 |
| P24226 | Histidinol dehydrogenase, chloropla... | Brassica oleracea var. capitata | AIEEEIAKQCK | Acetyl (K),Deam (NQ),TriOx (C) | 2 | 1408 .64 | -1 .42 | 648380 | 112 |
| P26300 | Enolase | Solanum lycopersicum | AAVPSGASTGIYEALELR | Unmodified | 2 | 1803 .94 | 2 .75 | 305420 | 72 |
| P26859 | 60S ribosomal protein L2, mitochond... | Marchantia polymorpha | VENTPQCIVR | 2 Deam (NQ),TriOx (C) | 2 | 1264 .56 | 4 .00 | 246570 | 76 |
| P27480 | Linoleate 9S-lipoxygenase 1 | Phaseolus vulgaris | IYDYAVYNDLGNPDK | Deam (NQ),Ox Y,diOx Y | 2 | 1807 .78 | -0 .70 | 327830 | 48 |
| P27806 | Histone H1 | Triticum aestivum | PKTATKK | Unmodified | 1 | 772 .48 | 2 .50 | 13109000 | 43 |
| P28734 | Aspartate aminotransferase, cytopla... | Daucus carota | AEQMLVNDQSRVK | Deam (NQ) | 3 | 1517 .75 | 3 .37 | 757100 | 55 |
| P29197 | Chaperonin CPN60, mitochondrial | Arabidopsis thaliana | NVVIEQSWGAPK | 2 Deam (NQ),Di-Ox W | 2 | 1360 .65 | -1 .63 | 9506100 | 68 |
| P36439 | Light-independent protochlorophylli... | Polystichum acrostichoides | RILQIGCDPK | Deam (NQ),TriOx (C) | 2 | 1247 .62 | -1 .73 | 211440 | 56 |
| P36470 | 30S ribosomal protein S4, chloropla... | Rhapis humilis | SGSDLKTQLRSGK | Unmodified | 2 | 1375 .74 | 3 .52 | 97367 | 48 |
| P40782 | Cyprosin | Cynara cardunculus | EPGITFLAAK | Glu->pyro-Glu | 2 | 1027 .57 | 0 .68 | 454290 | 65 |
| P43213 | Pollen allergen Phl p 1 | Phleum pratense | SAGELELQFR | Unmodified | 2 | 1148 .58 | 0 .80 | 294170 | 107 |
| P43295 | Probable cysteine protease RD19B | Arabidopsis thaliana | ANLLRAMRHQK | Ox (M),Deam (NQ) | 2 | 1353 .73 | 4 .37 | 589390 | 82 |
| P46523 | ATP-dependent Clp protease ATP-bind... | Brassica napus | RIGFDLDYEK | O-2H (Y) | 2 | 1268 .60 | -2 .91 | 633060 | 58 |
| P48422 | Cytochrome P450 86A1 | Arabidopsis thaliana | IQKAMGIGSEDK | Ox (M),Deam (NQ) | 2 | 1292 .63 | -1 .84 | 1775500 | 59 |
| P49199 | 40S ribosomal protein S8 | Oryza sativa subsp. japonica | LLACISSRPGQCGR | Deam (NQ),TriOx (C) | 2 | 1622 .75 | -4 .15 | 1432200 | 63 |
| P55195 | Phosphoribosylaminoimidazole carbox... | Vigna aconitifolia | IIQDKYQQK | Deam (NQ),diOx Y | 2 | 1195 .61 | -0 .73 | 986670 | 67 |
| P60290 | DNA-directed RNA polymerase subunit... | Physcomitrium patens | KVDCGTSENIFVTPLQNNYKK | Acetyl (K),TriOx (C) | 3 | 2544 .22 | 3 .77 | 782150 | 47 |
| P69310 | Ubiquitin | Avena sativa | IQDKEGIPPDQQR | Unmodified | 3 | 1522 .77 | 0 .43 | 249030 | 85 |
| P80082 | ATP synthase subunit alpha, mitocho... | Spinacia oleracea | AAELTTLLESR | Unmodified | 2 | 1202 .65 | -0 .28 | 221130 | 100 |
| P82796 | Thylakoid lumenal 22 kDa protein | Spinacia oleracea | EYIDFFDGYSLTY | 2 Di-iodination | 4 | 2135 .29 | -4 .16 | 6261100 | 46 |
| P84733 | Putative cytochrome c oxidase subun... | Pinus strobus | VVEALSPR | Unmodified | 2 | 869 .50 | -0 .62 | 26919000 | 98 |
| P85915 | Elongation factor 1-alpha | Pseudotsuga menziesii | IGGIGTVPVGR | Unmodified | 2 | 1024 .60 | 0 .23 | 1057300 | 121 |
| P86809 | Non-specific lipid-transfer protein... | Apium graveolens var. rapaceum | DPNLKNYVNSPGAR | 2 Deam (NQ),diOx Y | 2 | 1577 .73 | 3 .41 | 525820 | 76 |
| P93236 | Zeaxanthin epoxidase, chloroplastic | Solanum lycopersicum | SIVLPLPQVSEMHAR | Ox (M),Deam (NQ) | 2 | 1692 .89 | -2 .68 | 2274700 | 62 |
| P93757 | Guanylate kinase 1 | Arabidopsis thaliana | NDSIWFLEVDSPYVREQKK | Trp->Kynurenine | 3 | 2356 .17 | 0 .04 | 781030 | 55 |
| Q01859 | ATP synthase subunit beta, mitochon... | Oryza sativa subsp. japonica | VLNTGSPITVPVGR | Unmodified | 2 | 1408 .80 | -0 .01 | 270830 | 85 |
| Q02400 | Late embryogenesis abundant protein... | Hordeum vulgare | EMGHKGGETRK | Ox (M),Glu->pyro-Glu | 2 | 1226 .58 | 2 .58 | 1250200 | 66 |
| Q05579 | Ribulose bisphosphate carboxylase l... | Coriandrum sativum | EATKWSPELAAACEVWK | Ox (W) | 2 | 1990 .95 | 1 .94 | 631300 | 65 |
| Q06215 | Polyphenol oxidase A1, chloroplasti... | Vicia faba | LVEVEVNDGNLRK | Deam (NQ) | 2 | 1484 .78 | 0 .51 | 1590300 | 83 |
| Q06572 | Pyrophosphate-energized vacuolar me... | Hordeum vulgare | QFNTIPGLMEGTAK | Ox (M),Gln->pyro-Glu | 2 | 1504 .72 | 0 .36 | 857570 | 52 |
| Q06735 | ATP synthase subunit alpha, mitocho... | Beta vulgaris | MPLDKISQYER | diOx Y | 2 | 1410 .68 | -1 .43 | 2354700 | 99 |
| Q06SJ3 | 30S ribosomal protein S4, chloropla... | *Stigeoclonium helveticum* | YNFLKDQYLNTYK | 2 Deam (NQ),Ox Y | 2 | 1726 .81 | 2 .06 | 2796000 | 61 |
| Q09X27 | DNA-directed RNA polymerase subunit... | *Morus indica* | EDLIEYGGVKEFK | Acetyl (K),Glu->pyro-Glu | 2 | 1549 .77 | -0 .68 | 155860 | 46 |
| Q0DY59 | 3-hydroxy-3-methylglutaryl-coenzyme... | *Oryza sativa subsp. japonica* | FSCITGDAMGMNMVSK | 3 Ox (M),Deam (NQ) | 2 | 1796 .71 | 2 .75 | 1527800 | 45 |
| Q0G9G1 | Protein TIC 214 | *Liriodendron tulipifera* | LDGNQGYQENWKLEILKDK | Deam (NQ),Trp->Kynurenine | 3 | 2295 .14 | 4 .01 | 681380 | 61 |
| Q0J7T6 | RNA polymerase sigma factor sigA | *Oryza sativa subsp. japonica* | ISTYVYWWIR | O-2H (W),Di-Ox W | 2 | 1431 .68 | 0 .40 | 149010 | 63 |
| Q0JF48 | Polycomb group protein EMF2A | *Oryza sativa subsp. japonica* | IMHMWNSFIR | Ox (M),Deam (NQ),Di-Ox W | 2 | 1382 .61 | 2 .67 | 1667800 | 51 |
| Q0JIF2 | Cyclin-B1-1 | *Oryza sativa subsp. japonica* | PLQQAPAGR | 2 Deam (NQ) | 2 | 938 .48 | 0 .53 | 1066100 | 71 |
| Q0WPR4 | Serine carboxypeptidase-like 34 | *Arabidopsis thaliana* | LISHNEGWR | Deam (NQ),Di-Ox W | 2 | 1143 .53 | -3 .00 | 80058 | 48 |
| Q0WPY0 | Tubby-like F-box protein 6 | *Arabidopsis thaliana* | GRVTVASVKNFQLVAAAAEAGK | Acetyl (K),Deam (NQ) | 3 | 2229 .21 | -2 .01 | 1298700 | 47 |
| Q0WUY1 | Calmodulin calcium-dependent NAD ki... | *Arabidopsis thaliana* | VKPQLNSHK | 2 Deam (NQ) | 2 | 1051 .57 | 0 .63 | 350870 | 48 |
| Q0ZPV7 | Carboxylesterase 1 | *Actinidia eriantha* | LEDPEKAK | Acetyl (K) | 2 | 970 .50 | -0 .16 | 4939000 | 121 |
| Q10MN5 | Kinesin-like protein KIN-14F | *Oryza sativa subsp. japonica* | QYSMLQLQSK | 3 Deam (NQ) | 2 | 1227 .57 | 4 .17 | 884640 | 57 |
| Q10N20 | Mitogen-activated protein kinase 5 | *Oryza sativa subsp. japonica* | YQPPIMPIGRGAYGIVCSVMNFETR | Ox (M),diOx Y | 2 | 2903 .38 | 0 .19 | 3355600 | 45 |
| Q10NQ9 | Eukaryotic translation initiation f... | *Oryza sativa subsp. japonica* | FVLWYTRRTPGAR | Trp->Kynurenine | 2 | 1625 .88 | -1 .71 | 641710 | 69 |
| Q14FE9 | Ribulose bisphosphate carboxylase l... | *Populus alba* | AGVKEYKLNYYTPEYETK | Iodination,Ox Y | 3 | 2336 .97 | 4 .17 | 6944900 | 62 |
| Q1KVQ9 | Uncharacterized membrane protein yc... | *Tetradesmus obliquus* | WYTYMQHYSTMKNQIGGTK | Acetyl (K),Ox (M),2 Deam (NQ),di-Ox (M) | 3 | 2428 .04 | 0 .98 | 893430 | 48 |
| Q1KVX8 | DNA-directed RNA polymerase subunit... | *Tetradesmus obliquus* | KASEISTIQK | Acetyl (K) | 2 | 1145 .63 | -0 .02 | 542280 | 115 |
| Q1KXR1 | Protein TIC 214 | *Helianthus annuus* | KGILIMEPAR | Acetyl (K) | 2 | 1168 .66 | -3 .98 | 2570100 | 89 |
| Q1PFN9 | Pumilio homolog 9 | *Arabidopsis thaliana* | PEEQFRVDPSEFGAR | Deam (NQ) | 2 | 1763 .81 | -2 .84 | 165570 | 45 |
| Q2EEX7 | ATP-dependent zinc metalloprotease ... | *Helicosporidium sp. subsp. Simulium jonesii* | LEENIHSSEWDTLATLEQK | Unmodified | 3 | 2242 .08 | 4 .00 | 1000700 | 55 |
| Q2PMQ0 | 30S ribosomal protein S8, chloropla... | *Glycine max* | ISTPGLRIYSNYQR | 2 Deam (NQ),diOx Y | 2 | 1700 .84 | 1 .19 | 253110 | 43 |
| Q2R3F5 | Auxin response factor 23 | *Oryza sativa subsp. japonica* | MNPGTLNSRSEDSHANSMERGSVGR | Deam (NQ),di-Ox (M) | 3 | 2721 .18 | -1 .04 | 1588000 | 45 |
| Q2WGD3 | Protein TIC 214 | *Selaginella uncinata* | GFAKPWRMGR | Ox (M),Di-Ox W | 2 | 1252 .61 | 0 .73 | 51461 | 40 |
| Q32065 | Uncharacterized 341.7 kDa protein i... | *Chlamydomonas reinhardtii* | SHLFNQK | Unmodified | 1 | 872 .45 | -2 .25 | 255430 | 57 |
| Q32RJ9 | tRNA(Ile)-lysidine synthase, chloro... | *Zygnema circumcarinatum* | DSMNFTDQLLINRSIYLPGK | 2 Deam (NQ) | 3 | 2326 .15 | -0 .34 | 220210 | 42 |
| Q32RK5 | Protein TIC 214 | *Zygnema circumcarinatum* | KQTDIQQMPEIR | Deam (NQ),di-Ox (M) | 2 | 1518 .73 | -1 .88 | 2016100 | 94 |
| Q33584 | Ribulose bisphosphate carboxylase l... | *Lathraea clandestina* | LAYYTPEYETK | Iodination,diOx Y | 2 | 1534 .54 | -1 .50 | 692230 | 48 |
| Q336R3 | E3 ubiquitin-protein ligase BRE1-li... | *Oryza sativa subsp. japonica* | QLEDIQDQLK | Gln->pyro-Glu | 2 | 1211 .60 | 2 .35 | 1292900 | 90 |
| Q38970 | Acetyl-CoA carboxylase 1 | *Arabidopsis thaliana* | LIQDWFCNSDIAK | O-2H (W) | 2 | 1622 .74 | 2 .82 | 1432200 | 77 |
| Q3C1P6 | Protein TIC 214 | *Nicotiana sylvestris* | YDLLAYKSINYEK | Deam (NQ),Iodination,O-2H (Y) | 2 | 1759 .68 | -1 .90 | 2515700 | 69 |
| Q3E9H1 | Putative F-box protein At5g16285 | *Arabidopsis thaliana* | LAVNSLPR | Deam (NQ) | 1 | 869 .50 | 0 .93 | 943670 | 46 |
| Q3S4A7 | Histidine kinase 5 | *Arabidopsis thaliana* | QWEYMQDNAVR | 2 Deam (NQ) | 2 | 1440 .60 | -3 .07 | 347540 | 46 |
| Q3ZIZ0 | Putative septum site-determining pr... | *Tupiella akineta* | KNMQNLVK | Ox (M) | 2 | 989 .53 | -3 .30 | 176340 | 50 |
| Q3ZJ77 | DNA-directed RNA polymerase subunit... | *Tupiella akineta* | KDLYKNQEDFNK | Deam (NQ),Ox Y | 2 | 1557 .73 | 2 .40 | 302570 | 44 |
| Q41351 | Ribulose bisphosphate carboxylase s... | *Stellaria longipes* | KTNLDITSIASNGGR | Acetyl (K) | 3 | 1587 .82 | 0 .80 | 257400 | 61 |
| Q41418 | 14-3-3-like protein | *Solanum tuberosum* | EAAENTLLAYK | O-2H (Y) | 2 | 1235 .60 | -0 .37 | 289610 | 80 |
| Q42059 | Endoglucanase 6 | *Arabidopsis thaliana* | TYYRYSTTVINK | Deam (NQ),2 Di-iodination,O-2H (Y) | 3 | 2026 .32 | -4 .18 | 1791400 | 51 |
| Q42510 | ARF guanine-nucleotide exchange fac... | *Arabidopsis thaliana* | DYRNMEGTLLLAIKLLSK | di-Ox (M) | 3 | 2109 .15 | 2 .55 | 358790 | 54 |
| Q43776 | Lysine--tRNA ligase | *Solanum lycopersicum* | VANISIPR | Deam (NQ) | 2 | 869 .50 | -1 .40 | 27709000 | 104 |
| Q494P0 | Probable inorganic phosphate transp... | *Arabidopsis thaliana* | MAGDQLNVLNALDVAK | Unmodified | 2 | 1670 .87 | -3 .88 | 1190000 | 86 |
| Q49KU0 | Protein TIC 214 | *Eucalyptus globulus subsp. globulus* | HPKMAENR | Ox (M),Deam (NQ) | 2 | 998 .46 | -3 .41 | 335370 | 55 |
| Q4VCM1 | Phospholipid--sterol O-acyltransfer... | *Arabidopsis thaliana* | LLSNSFASSLWLMPFSK | Trp->Kynurenine | 3 | 1930 .99 | 3 .53 | 525650 | 65 |
| Q56P11 | DNA-directed RNA polymerase subunit... | *Lactuca sativa* | IEGWNKSITR | Deam (NQ),Trp->Kynurenine | 2 | 1207 .62 | 0 .64 | 215990 | 73 |
| Q56W59 | F-box/LRR-repeat protein At5g35995 | *Arabidopsis thaliana* | QMEHFLRKLK | Gln->pyro-Glu | 2 | 1311 .71 | 2 .11 | 281830 | 72 |
| Q56WK6 | Patellin-1 | *Arabidopsis thaliana* | IQLQEKCVR | Deam (NQ),TriOx (C) | 2 | 1221 .60 | -3 .26 | 213060 | 46 |
| Q56WM6 | Rop guanine nucleotide exchange fac... | *Arabidopsis thaliana* | ITEQGSNGKSPVR | 2 Deam (NQ) | 2 | 1373 .68 | 0 .73 | 175050 | 47 |
| Q56XP4 | Sodium/hydrogen exchanger 2 | *Arabidopsis thaliana* | ISIKQQVVIWWAGLMR | Trp->Kynurenine,Di-Ox W | 3 | 1963 .07 | -4 .03 | 741330 | 50 |
| Q589B8 | DNA-directed RNA polymerase subunit... | *Silene latifolia* | RNSIFAYFNDPR | Unmodified | 3 | 1498 .73 | 0 .92 | 3871900 | 46 |
| Q5J2X4 | Maturase K | *Blitum bonus-henricus* | NLNSLITPNQLISFLK | 3 Deam (NQ) | 3 | 1816 .98 | 4 .21 | 1292500 | 46 |
| Q5JK68 | Cyclin-dependent kinase C-2 | *Oryza sativa subsp. japonica* | MLTLDPSQRISAK | Ox (M) | 3 | 1474 .78 | -1 .47 | 253270 | 42 |
| Q5K4R0 | MADS-box transcription factor 47 | *Oryza sativa subsp. japonica* | KRMQLIEENLR | Acetyl (K),2 Deam (NQ),di-Ox (M) | 2 | 1504 .76 | -0 .87 | 4512000 | 86 |
| Q5S2C3 | Protein PIR | *Arabidopsis thaliana* | VVRFNYTAEER | Deam (NQ),Ox Y | 2 | 1399 .67 | -0 .01 | 497520 | 61 |
| Q5SCW2 | Photosystem I assembly protein Ycf4 | *Huperzia lucidula* | GQQDVPLTR | 2 Deam (NQ) | 2 | 1014 .50 | -0 .88 | 267750 | 50 |
| Q5W6F1 | Trans-cinnamate 4-monooxygenase | *Oryza sativa subsp. japonica* | ILVNAWFLANDPKR | O-2H (W) | 2 | 1669 .89 | 4 .16 | 1709500 | 76 |
| Q5XF78 | Gibberellic acid methyltransferase ... | *Arabidopsis thaliana* | VLEKESRTWNK | Acetyl (K),Deam (NQ) | 2 | 1431 .74 | 0 .27 | 1399300 | 87 |
| Q652A8 | UDP-glucose 4-epimerase 3 | *Oryza sativa subsp. japonica* | DQWNWAKKNPYGYSANAEQN | Deam (NQ),Iodination,Di-iodination | 4 | 2761 .74 | -3 .41 | 6430000 | 42 |
| Q652V8 | 16.0 kDa heat shock protein, peroxi... | *Oryza sativa subsp. japonica* | GAAPHAAAEKERER | Acetyl (K) | 2 | 1533 .76 | -3 .04 | 1155600 | 86 |
| Q655X0 | Thioredoxin O, mitochondrial | *Oryza sativa subsp. japonica* | YPKIPIYK | Iodination,diOx Y | 2 | 1178 .49 | -1 .01 | 373250 | 53 |
| Q67YM6 | Dirigent protein 11 | *Arabidopsis thaliana* | PYSKTTPFQGNK | 2 Deam (NQ),diOx Y | 2 | 1400 .65 | -2 .65 | 297040 | 46 |
| Q688U3 | Zinc-finger homeodomain protein 6 | *Oryza sativa subsp. japonica* | VWMHNNKSSIGSSSGGGSR | Acetyl (K),Deam (NQ),di-Ox (M) | 2 | 2021 .89 | -1 .02 | 3455100 | 44 |
| Q69T31 | Cytosolic invertase 1 | *Oryza sativa subsp. japonica* | WEELIGEMPLKICYPAIENHEWR | di-Ox (M) | 3 | 2944 .39 | 2 .99 | 825460 | 48 |
| Q6ESI7 | Tripeptidyl-peptidase 2 | *Oryza sativa subsp. japonica* | YTPLLAKILECIVQK | O-2H (Y) | 3 | 1802 .00 | -2 .33 | 970320 | 69 |
| Q6I621 | Serine/threonine protein phosphatas... | *Oryza sativa subsp. japonica* | IYGKFMVHR | Acetyl (K),Ox (M) | 2 | 1207 .62 | 3 .38 | 243210 | 68 |
| Q6K2M1 | BURP domain-containing protein 14 | *Oryza sativa subsp. japonica* | MEVSKIEAAAR | di-Ox (M) | 2 | 1235 .62 | -2 .49 | 1007300 | 88 |
| Q6K8S0 | Cyclin-F2-2 | *Oryza sativa subsp. japonica* | DMEKDAAQR | Acetyl (K),Ox (M),Deam (NQ) | 2 | 1121 .47 | 0 .21 | 9069200 | 65 |
| Q6L438 | Putative late blight resistance pro... | *Solanum demissum* | VMAYLVMQK | Deam (NQ),2 di-Ox (M) | 2 | 1146 .53 | 4 .17 | 193630 | 40 |
| Q6NLP7 | GDSL esterase/lipase At3g62280 | *Arabidopsis thaliana* | AIQTVYLYGGR | Iodination,O-2H (Y) | 2 | 1379 .54 | -4 .06 | 135420 | 58 |
| Q6NPR7 | Probable methyltransferase PMT24 | *Arabidopsis thaliana* | EKTQLEESSEENK | Glu->pyro-Glu | 2 | 1531 .70 | -4 .18 | 580230 | 88 |
| Q6VAB0 | UDP-glycosyltransferase 85C2 | *Stevia rebaudiana* | RLVQELMGEGGHKMR | 2 Ox (M) | 2 | 1771 .88 | -0 .37 | 416880 | 62 |
| Q6X7K0 | WUSCHEL-related homeobox 1 | *Arabidopsis thaliana* | FNMAVVTMTAEQNKR | Ox (M),Deam (NQ) | 2 | 1755 .83 | 4 .44 | 388820 | 50 |
| Q6Y9P5 | Coronatine-insensitive protein homo... | *Oryza sativa subsp. japonica* | MGGEVPEPR | Ox (M) | 2 | 986 .45 | 0 .23 | 243060 | 46 |
| Q6YTF1 | Oryzalexin D synthase | *Oryza sativa subsp. japonica* | LAHIRDNKPR | Deam (NQ) | 2 | 1219 .68 | 2 .12 | 1508100 | 81 |
| Q6YU88 | Kinesin-like protein KIN-6 | *Oryza sativa subsp. japonica* | KSPIEQSEEER | Acetyl (K),Deam (NQ) | 2 | 1373 .63 | 1 .01 | 1133300 | 78 |
| Q6Z0D2 | Putative B3 domain-containing prote... | *Oryza sativa subsp. japonica* | NSYTVRVDKSQEK | Deam (NQ),diOx Y | 2 | 1585 .76 | 4 .47 | 507110 | 60 |
| Q6Z2X3 | Molybdopterin synthase catalytic su... | *Oryza sativa subsp. japonica* | ADAMEACRYVIDEVK | Iodination | 2 | 1894 .71 | 4 .45 | 998180 | 56 |
| Q6Z3A8 | Clathrin light chain 3 | *Oryza sativa subsp. japonica* | LNGDTNRAQNR | 2 Deam (NQ) | 2 | 1259 .59 | 0 .20 | 6146200 | 67 |
| Q6Z3U3 | B3 domain-containing protein Os07g0... | *Oryza sativa subsp. japonica* | RLHCGCIASK | TriOx (C) | 2 | 1248 .57 | 2 .43 | 1105900 | 63 |
| Q6Z965 | 12-oxophytodienoate reductase 7 | *Oryza sativa subsp. japonica* | LNALQQQSGR | 4 Deam (NQ) | 2 | 1117 .53 | -0 .58 | 181970 | 66 |
| Q6ZDY8 | Succinate dehydrogenase [ubiquinone... | *Oryza sativa subsp. japonica* | RDDEQWMKHSLGYWENEK | Ox (M),2 Deam (NQ) | 3 | 2368 .01 | -1 .82 | 1051000 | 49 |
| Q70US9 | Volkensin | *Adenia volkensii* | YIEGLVRQSIVGPGDYRTFR | Deam (NQ),O-2H (Y) | 3 | 2340 .19 | -3 .55 | 453870 | 47 |
| Q7FK82 | Probable L-type lectin-domain conta... | *Arabidopsis thaliana* | AYASSYLGLFNR | Ox Y | 3 | 1376 .67 | -0 .93 | 974830 | 46 |
| Q7X659 | Vacuolar protein sorting-associated... | *Arabidopsis thaliana* | LLKKPDQCR | Deam (NQ) | 2 | 1157 .62 | -0 .87 | 901720 | 101 |
| Q7XA72 | ABC transporter G family member 21 | *Arabidopsis thaliana* | QSLISSYKKNLYPPLK | 2 Acetyl (K),Deam (NQ) | 2 | 1963 .07 | -0 .64 | 2244900 | 58 |
| Q7XCA7 | Expansin-B6 | *Oryza sativa subsp. japonica* | TLVANNVIPANWSPNSNYR | O-2H (Y) | 3 | 2143 .04 | -1 .82 | 3068300 | 50 |
| Q7Y1C5 | Protein BREAST CANCER SUSCEPTIBILIT... | *Arabidopsis thaliana* | RDTAYAYNKQDSTQK | 2 Deam (NQ) | 2 | 1789 .81 | -1 .14 | 240100 | 44 |
| Q84ST4 | Chlorophyll(ide) b reductase NOL, c... | *Oryza sativa subsp. japonica* | EAINMMRNQPR | Ox (M),Deam (NQ) | 2 | 1375 .63 | 0 .21 | 225470 | 56 |
| Q84TA3 | Leucine aminopeptidase | *Oryza sativa subsp. japonica* | QIGRPAFDEFLK | Deam (NQ) | 3 | 1420 .74 | -3 .41 | 143780 | 56 |
| Q84TD8 | Protein FLX-like 2 | *Arabidopsis thaliana* | SEREQRMMGLAEK | Unmodified | 2 | 1563 .75 | 0 .97 | 1111300 | 69 |
| Q84TQ7 | DELLA protein GAI | *Gossypium hirsutum* | QICNVVACEGMDR | Ox (M),TriOx (C) | 2 | 1614 .64 | 4 .24 | 1307400 | 60 |
| Q84WJ0 | Protein DA1-related 5 | *Arabidopsis thaliana* | NQLQYMR | Ox (M),2 Deam (NQ) | 2 | 969 .42 | -0 .12 | 1328200 | 57 |
| Q84WJ9 | Protein phosphatase 1 regulatory in... | *Arabidopsis thaliana* | VMENLENFTK | Ox (M),2 Deam (NQ) | 2 | 1241 .55 | 4 .28 | 46485 | 41 |
| Q84ZW8 | Acyclic sesquiterpene synthase | *Zea mays* | KLQKPIDTCR | Deam (NQ),TriOx (C) | 2 | 1306 .66 | -3 .26 | 408430 | 43 |
| Q85AI6 | 30S ribosomal protein S7, chloropla... | *Adiantum capillus-veneris* | LSDELIDAARNSGSAIR | Unmodified | 2 | 1786 .92 | 3 .25 | 4953200 | 119 |
| Q85FJ7 | Photosystem II CP47 reaction center... | *Adiantum capillus-veneris* | LGEAENLSLSQVWSK | Deam (NQ) | 2 | 1660 .83 | -0 .01 | 1596700 | 42 |
| Q8GU87 | ABC transporter G family member 31 | *Oryza sativa subsp. japonica* | NINYYVDVPAELK | Iodination,Ox Y | 2 | 1678 .67 | -2 .68 | 275580 | 70 |
| Q8GUI4 | Uncharacterized GPI-anchored protei... | *Arabidopsis thaliana* | GLANCKINRVCPLVFPHMK | 2 Deam (NQ) | 3 | 2255 .14 | 0 .72 | 1300000 | 54 |
| Q8GUK1 | Protein DGS1, mitochondrial | *Arabidopsis thaliana* | IMQYQSYIEQGR | Ox (M),Deam (NQ) | 2 | 1531 .70 | -3 .80 | 140230 | 49 |
| Q8GY42 | NAC transcription factor 25 | *Arabidopsis thaliana* | IYKKNSSQR | 2 Acetyl (K),Deam (NQ) | 2 | 1207 .62 | 0 .55 | 215990 | 80 |
| Q8H151 | Malonate--CoA ligase | *Arabidopsis thaliana* | SFNYLSLINHR | Deam (NQ),Ox Y | 2 | 1379 .68 | 2 .94 | 122010 | 46 |
| Q8H1B3 | Heat shock 70 kDa protein BIP3 | *Arabidopsis thaliana* | QATKDAGAIAGLNVVR | Acetyl (K),Deam (NQ) | 3 | 1625 .87 | 1 .13 | 589320 | 64 |
| Q8H1U4 | Anaphase-promoting complex subunit ... | *Arabidopsis thaliana* | LIQHLALYKGYK | diOx Y | 3 | 1477 .83 | 1 .08 | 2902500 | 40 |
| Q8H3C7 | IAA-amino acid hydrolase ILR1-like ... | *Oryza sativa subsp. japonica* | PYPAVVNDEGMYAHAR | Deam (NQ),Ox Y,diOx Y | 2 | 1837 .79 | 1 .89 | 382180 | 51 |
| Q8L633 | tRNase Z TRZ2, chloroplastic | *Arabidopsis thaliana* | KLQKQYAHLK | Deam (NQ),O-2H (Y) | 2 | 1270 .70 | -2 .92 | 1412800 | 76 |
| Q8L6Z7 | Exonuclease 1 | *Arabidopsis thaliana* | TVINMSSASKR | Ox (M),Deam (NQ) | 2 | 1209 .60 | -1 .86 | 577880 | 46 |
| Q8L7G4 | Terpenoid synthase 9 | *Arabidopsis thaliana* | GYVVNAVNCYMK | Deam (NQ),O-2H (Y) | 2 | 1431 .62 | 0 .77 | 91904 | 50 |
| Q8LDG7 | Peroxisome biogenesis protein 3-1 | *Arabidopsis thaliana* | LFFTLLYANMPQ | Ox (M),diOx Y | 2 | 1504 .73 | -1 .34 | 857570 | 67 |
| Q8LFN2 | Probable inactive leucine-rich repe... | *Arabidopsis thaliana* | LQQLLYYPK | Deam (NQ),diOx Y,O-2H (Y) | 2 | 1211 .61 | -3 .77 | 1292900 | 121 |
| Q8LK56 | Transcriptional activator DEMETER | *Arabidopsis thaliana* | NMELQEGDMSK | 2 Ox (M),2 Deam (NQ) | 2 | 1314 .50 | 4 .10 | 588810 | 40 |
| Q8M9W2 | 30S ribosomal protein S4, chloropla... | *Chaetosphaeridium globosum* | QKLRYHYGLHER | Gln->pyro-Glu | 3 | 1581 .82 | -2 .56 | 120080 | 44 |
| Q8MEX2 | Maturase K | *Ginkgo biloba* | FKRDGEEYISYQR | 2 Di-iodination | 3 | 2193 .40 | 2 .23 | 95330 | 42 |
| Q8RWE5 | Plant intracellular Ras-group-relat... | *Arabidopsis thaliana* | QIDGIASPR | Gln->pyro-Glu | 2 | 938 .48 | 0 .65 | 1056600 | 67 |
| Q8RWY6 | CLIP-associated protein | *Arabidopsis thaliana* | LGDSKQPVR | Deam (NQ) | 2 | 999 .53 | -2 .58 | 194880 | 46 |
| Q8RXS6 | Chromatin-remodeling ATPase INO80 | *Arabidopsis thaliana* | TILSEGGVLQVHYVK | Deam (NQ),diOx Y | 2 | 1674 .88 | 0 .47 | 974800 | 48 |
| Q8RY24 | Probable sucrose-phosphate synthase... | *Arabidopsis thaliana* | KQLEWEDSQR | Deam (NQ) | 2 | 1318 .62 | -2 .28 | 2556100 | 82 |
| Q8VWF8 | DNA-directed RNA polymerase 2, chlo... | *Nicotiana sylvestris* | DKQKEDGEHVTQEQEK | 2 Deam (NQ) | 3 | 1928 .86 | 1 .48 | 1254300 | 65 |
| Q8VYZ7 | WAT1-related protein At3g28070 | *Arabidopsis thaliana* | MPIMAGTASPWRR | Ox (W) | 2 | 1488 .73 | -0 .91 | 364750 | 43 |
| Q8VZ17 | Histone-lysine N-methyltransferase,... | *Arabidopsis thaliana* | GVSVLENGGVCKLDRMSGLK | Acetyl (K),Deam (NQ),TriOx (C) | 2 | 2209 .07 | -2 .26 | 1043400 | 44 |
| Q8VZ74 | GTPase ERA-like, chloroplastic | *Arabidopsis thaliana* | PPMLLVMNK | Deam (NQ),2 di-Ox (M) | 2 | 1106 .54 | 1 .56 | 198140 | 49 |
| Q8W3K0 | Probable disease resistance protein... | *Arabidopsis thaliana* | NVWQNILGDLK | Deam (NQ),O-2H (W) | 2 | 1313 .66 | -1 .63 | 333910 | 74 |
| Q93VR3 | GDP-mannose 3,5-epimerase | *Arabidopsis thaliana* | MGTTNGTDYGAYTYK | Ox (M),Iodination,Di-iodination,diOx Y | 3 | 2067 .37 | -4 .11 | 19765000 | 43 |
| Q93ZC9 | Glucuronokinase 1 | *Arabidopsis thaliana* | LLEEECR | TriOx (C) | 2 | 995 .42 | 0 .43 | 5076300 | 82 |
| Q944C2 | Phosphoinositide phospholipase C 5 | *Arabidopsis thaliana* | ALWMMHGMFR | 3 Ox (M) | 2 | 1326 .57 | 2 .36 | 498260 | 45 |
| Q946J4 | Expansin-B13 | *Oryza sativa subsp. japonica* | IIAEDAIPDGWK | O-2H (W) | 2 | 1340 .66 | 3 .62 | 3087300 | 88 |
| Q947G8 | Galactinol synthase 1 | *Solanum lycopersicum* | EEHMDREDIKMLVK | Ox (M),Glu->pyro-Glu | 2 | 1769 .84 | -0 .66 | 427660 | 60 |
| Q94B60 | ATP-dependent Clp protease proteoly... | *Arabidopsis thaliana* | LNSSSSASSSSFPK | Unmodified | 2 | 1384 .65 | -0 .46 | 920010 | 82 |
| Q94BN2 | Spermine synthase | *Arabidopsis thaliana* | SDFQEVLVFESATYGK | Deam (NQ) | 3 | 1819 .85 | -0 .97 | 564420 | 44 |
| Q94C32 | Protein MRG1 | *Arabidopsis thaliana* | QLTDDWEYIAQKDKVVK | 2 Deam (NQ) | 3 | 2080 .04 | -4 .42 | 388530 | 42 |
| Q94F39 | Ninja-family protein AFP3 | *Arabidopsis thaliana* | KINGFLYR | Deam (NQ),diOx Y | 2 | 1042 .54 | -1 .95 | 1612600 | 65 |
| Q94II3 | Probable methyltransferase PMT21 | *Arabidopsis thaliana* | SNQNWLRKEGEK | 2 Deam (NQ) | 2 | 1489 .72 | 0 .50 | 200040 | 72 |
| Q96533 | Alcohol dehydrogenase class-3 | *Arabidopsis thaliana* | MATQGQVITCK | 2 Deam (NQ) | 2 | 1237 .57 | -2 .35 | 283810 | 58 |
| Q9ATB4 | Transcriptional adapter ADA2b | *Arabidopsis thaliana* | NVVSEYRMVKR | Ox (M),O-2H (Y) | 2 | 1409 .71 | 3 .65 | 162300 | 70 |
| Q9AY27 | Iron-phytosiderophore transporter y... | *Zea mays* | TYELAGASPANVPGSYK | diOx Y | 2 | 1755 .83 | 2 .10 | 388820 | 67 |
| Q9AYT5 | Putative GTP diphosphokinase RSH1, ... | *Oryza sativa subsp. japonica* | QDVKAEDLRQMFLAMTEEVR | Acetyl (K),Deam (NQ),di-Ox (M) | 3 | 2483 .17 | 0 .65 | 404710 | 48 |
| Q9AYU1 | Quinone-oxidoreductase QR1, chlorop... | *Triphysaria versicolor* | LQNGMVRPFLPR | Unmodified | 2 | 1426 .79 | -2 .94 | 4362700 | 99 |
| Q9C517 | Probable transcription factor At1g6... | *Arabidopsis thaliana* | NSISFEASVQQYIGK | 2 Deam (NQ) | 2 | 1671 .80 | -1 .59 | 501780 | 50 |
| Q9C5B9 | Probable aldo-keto reductase 1 | *Arabidopsis thaliana* | LGVSCIDLYYQHR | diOx Y | 3 | 1654 .78 | -0 .71 | 1591300 | 77 |
| Q9C5V6 | DNA topoisomerase 6 subunit B | *Arabidopsis thaliana* | VLKARGEASYYK | Ox Y,diOx Y | 2 | 1431 .74 | 0 .86 | 1393400 | 74 |
| Q9C5W6 | 14-3-3-like protein GF14 iota | *Arabidopsis thaliana* | EESKGNESNVKQIK | Acetyl (K),2 Deam (NQ) | 2 | 1632 .78 | 0 .78 | 434520 | 54 |
| Q9C638 | Protein WEAK CHLOROPLAST MOVEMENT U... | *Arabidopsis thaliana* | EEKCTEIAK | Acetyl (K),TriOx (C) | 2 | 1196 .52 | -4 .10 | 210000 | 72 |
| Q9C869 | MATH domain and coiled-coil domain-... | *Arabidopsis thaliana* | MQQLEQNLKDLK | Ox (M),Deam (NQ) | 2 | 1503 .76 | -1 .56 | 596410 | 86 |
| Q9C950 | Protein RTF1 homolog | *Arabidopsis thaliana* | LAEMNKKNR | Ox (M),2 Deam (NQ) | 2 | 1120 .55 | -1 .93 | 144690 | 58 |
| Q9CA78 | Nuclear intron maturase 4, mitochon... | *Arabidopsis thaliana* | EKVRLFALQK | Glu->pyro-Glu,Deam (NQ) | 2 | 1213 .72 | -4 .17 | 4489200 | 40 |
| Q9CAN6 | Pentatricopeptide repeat-containing... | *Arabidopsis thaliana* | GEPDLALNLLNKMEK | Acetyl (K),Deam (NQ) | 3 | 1726 .88 | 2 .06 | 143170 | 43 |
| Q9CAR4 | Probable WRKY transcription factor ... | *Arabidopsis thaliana* | VCVKASCEDPSINDGCQWRK | Deam (NQ) | 3 | 2409 .05 | 1 .37 | 2804200 | 69 |
| Q9FFW5 | Proline-rich receptor-like protein ... | *Arabidopsis thaliana* | EVAVKQLKIGGSQGER | 2 Deam (NQ) | 3 | 1699 .91 | -4 .42 | 609470 | 60 |
| Q9FI17 | Putative UDP-arabinose 4-epimerase ... | *Arabidopsis thaliana* | FTNLQDSLQVAWRWQK | 3 Deam (NQ) | 2 | 2021 .98 | -2 .06 | 1585100 | 47 |
| Q9FIK8 | Protein C2-DOMAIN ABA-RELATED 11 | *Arabidopsis thaliana* | ESTISCIDGEVVQSVWLR | Deam (NQ),Trp->Kynurenine | 2 | 2081 .99 | 1 .81 | 7236500 | 76 |
| Q9FJ13 | COBRA-like protein 9 | *Arabidopsis thaliana* | KIPPNDTADQPYR | Ox Y | 2 | 1529 .75 | -0 .24 | 146230 | 42 |
| Q9FJK7 | Cyclin-C1-2 | *Arabidopsis thaliana* | LLVFYMK | Ox (M),Ox Y | 2 | 944 .50 | 2 .36 | 409220 | 52 |
| Q9FJN8 | Ras-related protein RABA4a | *Arabidopsis thaliana* | SNMCCNS | Ox (M),2 Deam (NQ) | 1 | 889 .23 | -3 .21 | 135790 | 42 |
| Q9FKL2 | Transcription factor MYB36 | *Arabidopsis thaliana* | YGTGGNWIALPQK | Deam (NQ),O-2H (W) | 2 | 1418 .68 | 1 .31 | 100810 | 54 |
| Q9FN69 | Transcription factor GLABRA 3 | *Arabidopsis thaliana* | LGQIQEQQR | 3 Deam (NQ) | 2 | 1101 .53 | 0 .92 | 120760 | 45 |
| Q9FNA3 | Alpha-N-acetylglucosaminidase | *Arabidopsis thaliana* | EWIMMSHK | Ox (M),Glu->pyro-Glu | 2 | 1058 .47 | 0 .30 | 3225400 | 51 |
| Q9FNQ1 | DExH-box ATP-dependent RNA helicase... | *Arabidopsis thaliana* | SSDMSMSMLVK | 3 Ox (M) | 2 | 1262 .52 | -1 .53 | 1074800 | 50 |
| Q9FNZ5 | Protein NIM1-INTERACTING 1 | *Arabidopsis thaliana* | ALDLNLAL | Deam (NQ) | 1 | 842 .47 | -0 .31 | 1290900 | 55 |
| Q9FUA4 | Transcription factor SPATULA | *Arabidopsis thaliana* | SRINEKMK | Acetyl (K),Ox (M),Deam (NQ) | 2 | 1063 .53 | -1 .47 | 223220 | 46 |
| Q9FUH9 | Microtubule-binding protein TANGLED... | *Zea mays* | FGGGVGGEGAQWR | Deam (NQ),Di-Ox W | 2 | 1309 .57 | -0 .81 | 1742800 | 55 |
| Q9FVJ3 | ADP-ribosylation factor GTPase-acti... | *Arabidopsis thaliana* | AQSTVVK | Deam (NQ) | 1 | 732 .40 | 1 .00 | 253470 | 52 |
| Q9FW70 | Kinesin-like protein KIN-7K, chloro... | *Oryza sativa subsp. japonica* | VEIYAARNRMIDEK | Ox (M),Deam (NQ) | 3 | 1723 .86 | -0 .25 | 153580 | 67 |
| Q9FX32 | Sucrose synthase 6 | *Arabidopsis thaliana* | SKDGQEQHDVKVGER | Acetyl (K) | 2 | 1752 .84 | -3 .57 | 802660 | 79 |
| Q9FX68 | Zinc finger protein WIP6 | *Arabidopsis thaliana* | ETLSGGNNQEGLTAR | Deam (NQ) | 2 | 1546 .72 | 3 .98 | 132380 | 42 |
| Q9FY60 | Transcription factor MYB64 | *Arabidopsis thaliana* | GAAKDYTCK | Acetyl (K),TriOx (C) | 2 | 1102 .46 | 1 .79 | 1763400 | 55 |
| Q9FZ89 | UPF0426 protein At1g28150, chloropl... | *Arabidopsis thaliana* | LDLNEEPLK | Unmodified | 2 | 1069 .57 | 2 .39 | 13570000 | 119 |
| Q9LEW0 | Ubiquitin C-terminal hydrolase 22 | *Arabidopsis thaliana* | GLWYRCDDAWINEVEEEVVR | Deam (NQ),Di-Ox W | 3 | 2570 .14 | -0 .62 | 461590 | 50 |
| Q9LFE4 | WEB family protein At5g16730, chlor... | *Arabidopsis thaliana* | EMNRLDNLLK | Deam (NQ) | 2 | 1245 .64 | -2 .13 | 3087100 | 115 |
| Q9LFS4 | Protein NSP-INTERACTING KINASE 1 | *Arabidopsis thaliana* | IVLLQNNNIK | 3 Deam (NQ) | 2 | 1170 .65 | -1 .87 | 420180 | 50 |
| Q9LHK4 | Putative ABC transporter B family m... | *Arabidopsis thaliana* | IMASRNMTTVVVAHRLNTLK | Deam (NQ),di-Ox (M) | 3 | 2287 .21 | -1 .07 | 512470 | 42 |
| Q9LHN9 | IQ domain-containing protein IQM2 | *Arabidopsis thaliana* | ETKSFQLGKQLSCK | Acetyl (K),Deam (NQ),TriOx (C) | 2 | 1743 .83 | 3 .07 | 620640 | 61 |
| Q9LII8 | Protein KINESIN LIGHT CHAIN-RELATED... | *Arabidopsis thaliana* | SYCENALKIYLK | Ox Y,O-2H (Y) | 2 | 1530 .74 | 3 .06 | 1462100 | 61 |
| Q9LM13 | 2,3-bisphosphoglycerate-dependent p... | *Arabidopsis thaliana* | TIPDNSQKK | Deam (NQ) | 2 | 1030 .53 | 0 .41 | 460920 | 51 |
| Q9LME8 | UDP-glycosyltransferase 85A7 | *Arabidopsis thaliana* | EVERSKR | Unmodified | 1 | 902 .49 | 4 .02 | 1315600 | 45 |
| Q9LNV5 | Zinc finger CCCH domain-containing ... | *Arabidopsis thaliana* | TTPYYKR | diOx Y | 2 | 959 .47 | -3 .84 | 404680 | 57 |
| Q9LRK5 | Putative cysteine-rich repeat secre... | *Arabidopsis thaliana* | ELSKCCEGK | Acetyl (K),2 TriOx (C) | 2 | 1247 .46 | 4 .36 | 888880 | 55 |
| Q9LRK8 | Cysteine-rich repeat secretory prot... | *Arabidopsis thaliana* | QLVLYAAGEKR | Deam (NQ),O-2H (Y) | 2 | 1261 .67 | 0 .42 | 130590 | 71 |
| Q9LRW6 | F-box protein At3g13820 | *Arabidopsis thaliana* | STCKKWNNLSK | Di-Ox W | 3 | 1396 .68 | 3 .52 | 286240 | 47 |
| Q9LSV0 | Glyoxylate/succinic semialdehyde re... | *Arabidopsis thaliana* | AMSMNLLK | 2 Ox (M),Deam (NQ) | 2 | 939 .44 | -3 .84 | 1180700 | 76 |
| Q9LU01 | Ycf3-interacting protein 1, chlorop... | *Arabidopsis thaliana* | NRDMIFSEVKLTIMIEDPR | di-Ox (M) | 3 | 2338 .17 | -0 .54 | 178760 | 52 |
| Q9LU73 | Protein SMAX1-LIKE 5 | *Arabidopsis thaliana* | EDVELNIK | Glu->pyro-Glu,Deam (NQ) | 1 | 941 .47 | 0 .30 | 474240 | 59 |
| Q9LUJ7 | Vicilin-like seed storage protein A... | *Arabidopsis thaliana* | EKEEDKDLR | Acetyl (K),Glu->pyro-Glu | 2 | 1184 .57 | 1 .00 | 4968000 | 68 |
| Q9LV91 | 4-alpha-glucanotransferase DPE1, ch... | *Arabidopsis thaliana* | QWQKVREYAR | Deam (NQ),O-2H (Y) | 2 | 1377 .68 | -3 .65 | 487160 | 76 |
| Q9LYX1 | L-type lectin-domain containing rec... | *Arabidopsis thaliana* | EFTYKELK | Acetyl (K),Glu->pyro-Glu | 2 | 1080 .55 | -2 .55 | 137310 | 66 |
| Q9LYY3 | F-box/kelch-repeat protein At5g0302... | *Arabidopsis thaliana* | VSIFDYRTYQWR | Deam (NQ),Iodination | 2 | 1759 .69 | -3 .13 | 1864400 | 53 |
| Q9M0B9 | Transcription factor IBH1-like 1 | *Arabidopsis thaliana* | AIKLSADVAMASLR | Acetyl (K),Ox (M) | 2 | 1502 .81 | -3 .67 | 445320 | 54 |
| Q9M1D1 | Beta-glucosidase 27 | *Arabidopsis thaliana* | TQRKNMYSK | Acetyl (K),2 Deam (NQ),di-Ox (M) | 2 | 1230 .56 | -0 .52 | 530370 | 51 |
| Q9M354 | Probable ADP-ribosylation factor GT... | *Arabidopsis thaliana* | GVMAMATQK | Ox (M),Deam (NQ),di-Ox (M) | 2 | 984 .43 | -2 .98 | 118830 | 46 |
| Q9M3J4 | NAD(P)H-quinone oxidoreductase subu... | *Spinacia oleracea* | TINQKISLLNLLTMNNKER | Acetyl (K),2 Deam (NQ) | 3 | 2286 .23 | 1 .95 | 569380 | 48 |
| Q9M3W5 | Chlorophyll synthase, chloroplastic | *Avena sativa* | QETNIWKIRLQLTK | Di-Ox W | 3 | 1802 .01 | -3 .63 | 970320 | 75 |
| Q9M643 | Cycloeucalenol cycloisomerase | *Arabidopsis thaliana* | STDEWDLSR | O-2H (W) | 1 | 1121 .46 | 3 .42 | 621780 | 57 |
| Q9M658 | Helicase protein MOM1 | *Arabidopsis thaliana* | EYEENGQIQHGKSSDPK | 2 Deam (NQ),diOx Y | 3 | 1978 .84 | -1 .04 | 745290 | 43 |
| Q9M6D9 | Homeobox protein SHOOT MERISTEMLESS | *Brassica oleracea* | QQLLDWWSR | Deam (NQ),O-2H (W),Di-Ox W | 2 | 1277 .57 | 1 .19 | 1835200 | 57 |
| Q9MAU6 | Protein disulfide-isomerase like 2-... | *Arabidopsis thaliana* | MERKMYK | Acetyl (K),di-Ox (M) | 2 | 1058 .49 | 3 .70 | 818280 | 57 |
| Q9MBF8 | Dynein-1-beta heavy chain, flagella... | *Chlamydomonas reinhardtii* | IKSLNDFHTYAVYK | diOx Y,O-2H (Y) | 2 | 1743 .85 | -4 .47 | 724410 | 48 |
| Q9MVV9 | Maturase K | *Sciadopitys verticillata* | IWLLKDPFIHYLR | diOx Y | 3 | 1744 .97 | 1 .80 | 1483000 | 48 |
| Q9S834 | ATP-dependent Clp protease proteoly... | *Arabidopsis thaliana* | MAHACVSTSASSLR | TriOx (C),di-Ox (M) | 2 | 1556 .66 | 2 .61 | 273350 | 43 |
| Q9SA82 | Transcription factor bHLH52 | *Arabidopsis thaliana* | EMQFLLGSQEIQEK | 3 Deam (NQ) | 2 | 1681 .78 | -4 .39 | 641510 | 49 |
| Q9SAC6 | Alpha-glucan water dikinase 1, chlo... | *Arabidopsis thaliana* | NNDSDFYVDFAKEEK | 2 Deam (NQ),diOx Y | 2 | 1853 .75 | -3 .07 | 141070 | 47 |
| Q9SAI1 | Factor of DNA methylation 5 | *Arabidopsis thaliana* | EETLNKILQLEKELDSK | 2 Deam (NQ) | 3 | 2031 .06 | 2 .63 | 415130 | 46 |
| Q9SB48 | NADPH--cytochrome P450 reductase 1 | *Arabidopsis thaliana* | LQQLAYGVFALGNR | 3 Deam (NQ) | 2 | 1551 .79 | -2 .09 | 1268000 | 60 |
| Q9SCT2 | Pentatricopeptide repeat-containing... | *Arabidopsis thaliana* | FLWNQMKEQGIK | Ox (M),Ox (W) | 2 | 1552 .77 | 1 .34 | 1286600 | 82 |
| Q9SE97 | Formin-like protein 1 | *Arabidopsis thaliana* | RNQDLNFSDDSK | 3 Deam (NQ) | 2 | 1440 .60 | -4 .12 | 347540 | 60 |
| Q9SFC4 | O-fucosyltransferase 24 | *Arabidopsis thaliana* | SSMTSNERK | Ox (M) | 2 | 1054 .47 | 4 .25 | 696030 | 45 |
| Q9SH22 | Probable disease resistance protein... | *Arabidopsis thaliana* | VQDEIAQKLGLGGDEWTQKDK | Deam (NQ),O-2H (W) | 3 | 2372 .15 | -2 .04 | 483900 | 42 |
| Q9SHZ0 | Protease Do-like 4, mitochondrial | *Arabidopsis thaliana* | MLFRFLQTLAR | Ox (M),Deam (NQ) | 2 | 1411 .76 | 3 .74 | 529600 | 52 |
| Q9SJ02 | Stress enhanced protein 2, chloropl... | *Arabidopsis thaliana* | QIQIQQR | 2 Deam (NQ) | 2 | 914 .48 | -1 .16 | 168200 | 60 |
| Q9SKT7 | External alternative NAD(P)H-ubiqui... | *Arabidopsis thaliana* | IDASNKKIHCR | TriOx (C) | 2 | 1388 .68 | -3 .19 | 634750 | 71 |
| Q9SL29 | Putative cyclic nucleotide-gated io... | *Arabidopsis thaliana* | AEDLQFVASQFR | 2 Deam (NQ) | 2 | 1411 .66 | -1 .75 | 758540 | 71 |
| Q9SLN8 | 2-alkenal reductase (NADP(+)-depend... | *Nicotiana tabacum* | AEEVSNKQVILK | 2 Deam (NQ) | 2 | 1358 .73 | 0 .65 | 1083400 | 74 |
| Q9SMZ3 | F-box only protein 13 | *Arabidopsis thaliana* | MSSVCKRWK | Ox (M) | 2 | 1196 .58 | 0 .74 | 421220 | 92 |
| Q9SQ64 | Non-functional NADPH-dependent code... | *Papaver somniferum* | NISELPQRR | 2 Deam (NQ) | 2 | 1113 .58 | 0 .69 | 359050 | 57 |
| Q9SQV1 | DEAD-box ATP-dependent RNA helicase... | *Arabidopsis thaliana* | TNANLAMAQK | Ox (M),2 Deam (NQ) | 2 | 1078 .50 | -3 .65 | 791880 | 44 |
| Q9SR00 | Pentatricopeptide repeat-containing... | *Arabidopsis thaliana* | GLKPDMFTYNTIIR | Ox (M),Iodination | 2 | 1809 .76 | 2 .11 | 100740 | 41 |
| Q9SR01 | Pentatricopeptide repeat-containing... | *Arabidopsis thaliana* | EAMENRGVK | Deam (NQ),di-Ox (M) | 2 | 1065 .48 | 0 .47 | 384550 | 80 |
| Q9SR66 | DEMETER-like protein 2 | *Arabidopsis thaliana* | LFEELDINKEGLCLPHNR | Acetyl (K),Deam (NQ) | 3 | 2239 .09 | -2 .09 | 140510 | 44 |
| Q9STK9 | Cytochrome P450 71A24 | *Arabidopsis thaliana* | INLSEILVNLTNNVICR | 2 Deam (NQ) | 3 | 1986 .05 | 1 .79 | 608120 | 53 |
| Q9STM3 | Lysine-specific demethylase REF6 | *Arabidopsis thaliana* | SSPVASSPSKPKVSGK | Unmodified | 2 | 1541 .84 | 0 .35 | 1734400 | 96 |
| Q9SVG4 | Berberine bridge enzyme-like 19 | *Arabidopsis thaliana* | LFKIQYSVNWK | Deam (NQ),Di-Ox W | 2 | 1457 .76 | -2 .56 | 966150 | 96 |
| Q9SVS1 | Beta-glucosidase 47 | *Arabidopsis thaliana* | KSIVYEIMETK | Ox (M) | 2 | 1355 .70 | 3 .92 | 1363300 | 72 |
| Q9SX86 | Exocyst complex component SEC3B | *Arabidopsis thaliana* | NRLLVCILNICK | 2 Deam (NQ) | 2 | 1516 .81 | 0 .76 | 1341600 | 120 |
| Q9SY59 | NF-X1-type zinc finger protein NFXL... | *Arabidopsis thaliana* | VTCRQKCGAPR | Acetyl (K),TriOx (C) | 2 | 1421 .65 | 3 .39 | 766780 | 78 |
| Q9SYG1 | 17.4 kDa class III heat shock prote... | *Arabidopsis thaliana* | TVQIAVS | Deam (NQ) | 1 | 717 .39 | 1 .49 | 1031000 | 65 |
| Q9T0I1 | DNA (cytosine-5)-methyltransferase ... | *Arabidopsis thaliana* | YSVGDFVYQIPNYLSK | 2 Iodination,Di-iodination | 3 | 2395 .52 | 0 .10 | 41810000 | 41 |
| Q9TIR8 | Maturase K | *Lathraea clandestina* | DFRTNLWFVK | Deam (NQ),O-2H (W) | 2 | 1339 .66 | -0 .38 | 1603500 | 82 |
| Q9XHR2 | Eukaryotic translation initiation f... | *Zea mays* | SVEDIHGLMTMVK | Ox (M) | 2 | 1474 .72 | 3 .07 | 320550 | 54 |
| Q9ZPP1 | Calreticulin | *Berberis stolonifera* | WNGDANDK | 2 Deam (NQ),O-2H (W) | 2 | 934 .33 | -1 .30 | 10563000 | 65 |
| Q9ZQC5 | Interactor of constitutive active R... | *Arabidopsis thaliana* | KLESDVMELRANLMDK | Ox (M),Deam (NQ) | 3 | 1907 .93 | 0 .57 | 402780 | 60 |
| Q9ZQV9 | Nicotianamine synthase 1 | *Hordeum vulgare* | LSPEHQRMR | di-Ox (M) | 2 | 1184 .57 | -2 .75 | 14293000 | 92 |
| Q9ZSB1 | Subtilisin-like protease SBT3.10 | *Arabidopsis thaliana* | WKGGCESGELFNGSIHCNRK | Acetyl (K),TriOx (C) | 3 | 2425 .05 | -2 .36 | 4579500 | 46 |
| Q9ZST1 | 30S ribosomal protein S17, chloropl... | *Oryza sativa subsp. japonica* | VQGNGGSGASPWAGAATALRIQAAK | 2 Deam (NQ) | 4 | 2340 .18 | -2 .85 | 1251000 | 54 |
| Q9ZUL5 | Protein CHROMATIN REMODELING 19 | *Arabidopsis thaliana* | TLAELLPSMKK | Ox (M) | 2 | 1245 .70 | -3 .13 | 200790 | 58 |
| Q9ZUU3 | Pentatricopeptide repeat-containing... | *Arabidopsis thaliana* | KLEHALQFFRWTER | Deam (NQ),Trp->Kynurenine | 2 | 1864 .96 | -2 .01 | 367180 | 58 |
| Q9ZVF5 | WUSCHEL-related homeobox 6 | *Arabidopsis thaliana* | TLNLFPVREYQEK | Deam (NQ),Ox Y | 2 | 1652 .84 | -2 .50 | 182480 | 66 |
| R9WS04 | Lipoxygenase 2, chloroplastic | *Tanacetum cinerariifolium* | TLSVQKCYR | Deam (NQ),TriOx (C) | 2 | 1202 .56 | 3 .15 | 138310 | 43 |
| V9M398 | Disease resistance protein RUN1 | *Vitis rotundifolia* | AIEESRSSVIVFSENYARSR | Unmodified | 3 | 2299 .16 | -1 .54 | 38043000 | 41 |

Table S5 . List of peptides and proteins of Bacteria/Nematoda in trunk sample: for each peptide all the features (same sequence and different modifications) with the highest intensity are reported.

| Acc. Number | **Description** | ***Specie*** | **Peptide Sequence** | **Modifications** | **Charge** | **Mass** | **Mass error [ppm]** | **Intensity** | **Score** |
| --- | --- | --- | --- | --- | --- | --- | --- | --- | --- |
| B5EFI7 | ATP synthase subunit beta | *Geobacter bemidjiensis* | FTQAGSEVSALLGR | Unmod | 2 | 1434.75 | -0.95 | 2621000 | 183 |
|  |  |  | VVDLLAPYAR | Unmod | 2 | 1115.63 | 1.86 | 1724200 | 138 |
| Q0I1B8 | DNA mismatch repair protein MutS | *Haemophilus somnus* | CVTPMGSRLLKR | Acetyl (K),TriOx (C),di-Ox (M) | 2 | 1538.76 | 0.78 | 2446400 | 54 |
|  |  |  | NIEKLQCR | Acetyl (K),Deam (NQ),TriOx (C) | 2 | 1150.53 | -3.72 | 451030 | 54 |
| Q27443 | Histone H4 | *Ascaris suum* | ISGLIYEETR | Unmod | 2 | 1179.61 | 0.35 | 3591500 | 159 |
|  |  |  | TLYGFGG | Unmod | 1 | 713.34 | 1.04 | 2601500 | 64 |
|  |  |  | VFLENVIR | Unmod | 2 | 988.57 | -1.94 | 1314200 | 139 |
| Q7N0K1 | UPF0234 protein plu3881 | *Photorhabdus laumondii subsp. laumondii* | IASESDFQVNQLVDIMREK | Ox (M),Deam (NQ) | 3 | 2238.08 | 3.79 | 1801600 | 64 |
|  |  |  | IASESDFQVNQLVDIMREKLAK | Ox (M),Deam (NQ) | 3 | 2550.30 | 2.92 | 7771800 | 55 |
| Q9NL98 | Peroxiredoxin | *Ascaris suum* | QITVNDLPVGR | Gln->pyro-Glu | 2 | 1193.64 | 1.02 | 880190 | 138 |
|  |  |  | QITVNDLPVGR | Unmod | 2 | 1210.67 | 2.13 | 528040 | 127 |
| A0LLG0 | ATP synthase subunit alpha | *Syntrophobacter fumaroxidans* | VLSVGDGIAR | Unmod | 2 | 985.56 | -0.01 | 1335700 | 144 |
| A0LXZ7 | DNA mismatch repair protein MutS | *Gramella forsetii* | EEQQIIMITGPNMSGK | Ox (M),3 Deam (NQ) | 2 | 1793.81 | 0.81 | 15515000 | 76 |
| A0PZP4 | Endonuclease MutS2 | *Clostridium novyi* | LGAIMPIISEDGHFNIINAK | Ox (M),Deam (NQ) | 3 | 2169.11 | -3.90 | 1191400 | 61 |
| A0QRN6 | Imidazolonepropionase | *Mycolicibacterium smegmatis* | AAYPDGR | O-2H (Y) | 1 | 762.33 | 0.54 | 4594100 | 57 |
| A4G2M4 | Probable nicotinate-nucleotide aden... | *Herminiimonas arsenicoxydans* | VTVNIDQQEILR | Unmod | 2 | 1426.78 | -1.15 | 7425700 | 166 |
| A4XZJ1 | LPS-assembly protein LptD | *Pseudomonas mendocina* | DRIGDENK | Deam (NQ) | 1 | 946.44 | 3.92 | 971740 | 44 |
| A5GVF3 | DNA-directed RNA polymerase subunit... | *Synechococcus sp.* | EAAKQPGR | Acetyl (K),Glu->pyro-Glu,Deam (NQ) | 2 | 880.44 | 0.81 | 641570 | 54 |
| A8F962 | Cysteine--tRNA ligase | *Bacillus pumilus* | TINIYNTLTRK | Iodination | 2 | 1461.65 | -1.54 | 6799200 | 91 |
| A8G9Z9 | DNA mismatch repair protein MutS | *Serratia proteamaculans* | QDNLLAAIWQDAR | 2 Deam (NQ),Trp->Kynurenine | 2 | 1518.73 | 0.54 | 7173200 | 138 |
| A8XP14 | Golgi SNAP receptor complex member ... | *Caenorhabditis briggsae* | MRVDQLRMDVQR | Ox (M),2 Deam (NQ) | 2 | 1563.75 | 2.42 | 1257200 | 82 |
| A9B5I5 | Imidazole glycerol phosphate syntha... | *Herpetosiphon aurantiacus* | NIMVEVVENVAR | 2 Deam (NQ) | 2 | 1373.69 | -1.68 | 1492000 | 97 |
| A9BCN3 | 50S ribosomal protein L6 | *Prochlorococcus marinus* | SLVANMVEGVSKGYSK | Deam (NQ),O-2H (Y) | 3 | 1682.82 | -3.56 | 2287200 | 98 |
| A9KNI7 | Uronate isomerase | *Lachnoclostridium phytofermentans* | TAFMVSVGREYNR | Ox (M),Ox Y | 2 | 1560.74 | -2.07 | 951380 | 62 |
| B0SSU3 | Phosphoglycerate kinase | *Leptospira biflexa serovar Patoc* | PKGGPEPKYSMK | Ox (M),O-2H (Y) | 2 | 1347.65 | -4.03 | 8478700 | 74 |
| B0T3J0 | Methionine--tRNA ligase | *Caulobacter sp.* | KIEDEQIAEWTR | O-2H (W) | 2 | 1530.73 | -1.46 | 647140 | 103 |
| B0TWP7 | NADH-quinone oxidoreductase subunit... | *Francisella philomiragia subsp. philomiragia* | PGGVARDLPTQMPQYK | Deam (NQ),O-2H (Y) | 2 | 1771.86 | -2.82 | 37938000 | 122 |
| B1GZ89 | 30S ribosomal protein S3 | *Endomicrobium trichonymphae* | GGQGIESLR | Deam (NQ) | 1 | 916.46 | 3.35 | 2016000 | 71 |
| B3H269 | Malate dehydrogenase | *Actinobacillus pleuropneumoniae serotype 7* | LFGVTTLDVVR | Unmod | 2 | 1218.70 | -0.52 | 3877200 | 141 |
| B3QZT2 | 50S ribosomal protein L27 | *Phytoplasma mali* | LGAKISDGQYATAGSIIYRQR | Deam (NQ),diOx Y | 3 | 2300.18 | 1.74 | 2316700 | 55 |
| B4EW46 | Bifunctional protein HldE | *Proteus mirabilis* | PGGAANVAMNIASLGANSR | Ox (M),2 Deam (NQ) | 2 | 1787.85 | -1.12 | 2108700 | 58 |
| B4STN9 | Histidine biosynthesis bifunctional... | *Stenotrophomonas maltophilia* | PGIGLMTAYLQDR | Ox (M),Deam (NQ) | 2 | 1450.71 | -3.64 | 958610 | 78 |
| B5E4W4 | Formate--tetrahydrofolate ligase | *Streptococcus pneumoniae serotype 19F* | VNFEKKAQTQIAQVVQNGWDK | 3 Deam (NQ) | 3 | 2433.22 | -4.40 | 1338500 | 77 |
| B6YS43 | Serine hydroxymethyltransferase | *Azobacteroides pseudotrichonymphae genomovar. CFP2* | QCEGIELIASENFVSPQVLK | 3 Deam (NQ) | 2 | 2263.09 | -1.40 | 2149200 | 84 |
| B7GH28 | Probable GTP-binding protein EngB | *Anoxybacillus flavithermus* | PGKTQTLNFYR | Deam (NQ),Ox Y | 2 | 1340.67 | -1.11 | 2642900 | 96 |
| B7I5G8 | Histidine--tRNA ligase | *Acinetobacter baumannii* | EWEAQQLAVK | Glu->pyro-Glu,2 Deam (NQ) | 2 | 1184.57 | -3.66 | 13744000 | 96 |
| B7KN46 | DNA-directed RNA polymerase subunit... | *Methylorubrum extorquens* | DNLILQQRSAENAANAAELSELPPAAAE | 3 Deam (NQ) | 4 | 2908.39 | -3.61 | 641680 | 54 |
| B8E1E7 | 30S ribosomal protein S8 | *Dictyoglomus turgidum* | IKNANMR | Ox (M),2 Deam (NQ) | 2 | 863.42 | -3.91 | 7468300 | 91 |
| B8F789 | Aspartate--tRNA ligase | *Haemophilus parasuis serovar 5* | MQQTVFGILGINEEEQKEK | Ox (M) | 3 | 2236.11 | 3.51 | 2065500 | 61 |
| B9MJU0 | DNA mismatch repair protein MutS | *Caldicellulosiruptor bescii* | SNYSLVPDRYIR | Deam (NQ),Ox Y,diOx Y | 2 | 1530.73 | 1.37 | 4145000 | 65 |
| C0ZL89 | Lysylphosphatidylglycerol biosynthe... | *Rhodococcus erythropolis* | MESPPENPRQAR | Ox (M),Deam (NQ) | 2 | 1427.65 | -0.34 | 2821600 | 87 |
| C4LA32 | Protein translocase subunit SecA | *Tolumonas auensis* | ARQAYLTENGQIFVEGWLK | 2 Deam (NQ) | 3 | 2224.12 | -2.63 | 42477000 | 63 |
| C5D9H6 | DNA mismatch repair protein MutL | *Geobacillus sp.* | QSSPVQEPK | Gln->pyro-Glu | 1 | 981.48 | -1.35 | 293020 | 61 |
| D3KYU3 | Geranyl diphosphate 2-C-methyltrans... | *Streptomyces lasalocidi* | VEGVTLSAAQAEFGNR | Deam (NQ) | 3 | 1648.81 | -2.06 | 1165300 | 68 |
| E3PY95 | D-ornithine 4,5-aminomutase subunit... | *Acetoanaerobium sticklandii* | MEKDLQLRVNEK | Deam (NQ) | 2 | 1502.78 | -2.51 | 8670500 | 144 |
| I6LDA6 | 2,3-bisphosphoglycerate-independent... | *Onchocerca volvulus* | VATYDLEPAMSSAGVADKMIEQLNRK | Ox (M) | 3 | 2852.41 | 1.95 | 3909500 | 43 |
| O17323 | Histone deacetylase 4 | *Caenorhabditis elegans* | NGLIGSSSTSSLASNVSMGSHQYQSLLK | Unmod | 3 | 2852.40 | 4.22 | 60983000 | 46 |
| O18086 | Nuclear hormone receptor family mem... | *Caenorhabditis elegans* | PLNKQEKR | 2 Deam (NQ) | 2 | 1013.55 | 0.82 | 1159800 | 115 |
| O30862 | Protein GrpE | *Vibrio cholerae serotype O1* | EQQDSVLRARAEVENMR | Ox (M),Glu->pyro-Glu | 2 | 2027.98 | 0.46 | 971590 | 51 |
| O31557 | Uncharacterized protein YfjB | *Bacillus subtilis* | IAENYAKYQDK | Deam (NQ),Ox Y | 2 | 1358.64 | -0.30 | 781160 | 78 |
| O34439 | UPF0065 protein YflP | *Bacillus subtilis* | EWQTVALPK | Glu->pyro-Glu,Deam (NQ) | 2 | 1053.55 | -2.90 | 1645800 | 70 |
| O44568 | Probable peptide chain release fact... | *Caenorhabditis elegans* | SELSQLR | Deam (NQ) | 2 | 832.43 | 0.69 | 1053900 | 192 |
| O51149 | 30S ribosomal protein S2 | *Borrelia burgdorferi* | KEISQLNR | Acetyl (K),Deam (NQ) | 2 | 1029.55 | 0.07 | 8699300 | 144 |
| O86793 | tRNA N6-adenosine threonylcarbamoyl... | *Streptomyces coelicolor* | EGDPNAIAFPR | Glu->pyro-Glu,Deam (NQ) | 2 | 1168.55 | -1.65 | 534220 | 74 |
| P0AA92 | Uncharacterized lipoprotein YeaY | *Escherichia coli O6:H1* | IGNTPYKFMVMQVTGYK | 2 Ox (M),Deam (NQ) | 2 | 2008.96 | -1.02 | 736660 | 58 |
| P0AEF3 | DNA replication protein DnaC | *Shigella flexneri* | MKNVGDLMQR | Ox (M) | 2 | 1206.59 | 1.45 | 985260 | 96 |
| P17331 | Glyceraldehyde-3-phosphate dehydrog... | *Caenorhabditis elegans* | VPTPDVSVVDLTVR | Unmod | 2 | 1495.83 | 1.02 | 2675900 | 152 |
| P21880 | Dihydrolipoyl dehydrogenase | *Bacillus subtilis* | VLNSTGALALK | Deam (NQ) | 2 | 1086.63 | 3.28 | 3790100 | 129 |
| P23200 | Galactoside transport system permea... | *Escherichia coli* | QVGLAAVVAATLLQSMDNANK | Ox (M),Deam (NQ) | 2 | 2130.10 | -0.44 | 1307600 | 63 |
| P23315 | Anthranilate synthase component 1 | *Acinetobacter calcoaceticus* | LSNVPEADPVGLPDIWMMLSK | Ox (M) | 3 | 2327.15 | 0.18 | 1279800 | 55 |
| P24398 | MAPK phosphothreonine lyase | *Salmonella typhimurium* | SGRDGGEMQRQALR | Ox (M) | 2 | 1575.75 | -0.64 | 830850 | 94 |
| P25051 | Vancomycin/teicoplanin A-type resis... | *Enterococcus faecium* | PARSGSSFGVKK | Unmod | 2 | 1219.67 | 0.33 | 2312700 | 118 |
| P31728 | Probable D-methionine-binding lipop... | *Haemophilus influenzae* | KIKNVNELQDGAK | 3 Deam (NQ) | 3 | 1458.76 | -0.36 | 23698000 | 75 |
| P37646 | Cyclic di-GMP phosphodiesterase Pde... | *Escherichia coli* | QQPKILRQIER | 3 Deam (NQ) | 2 | 1410.78 | 2.14 | 1934900 | 85 |
| P41185 | DNA-directed RNA polymerase subunit... | *Buchnera aphidicola subsp. Schizaphis graminum* | NVELNIIDNFRR | 2 Deam (NQ) | 2 | 1503.77 | -0.39 | 14838000 | 112 |
| P49632 | Ubiquitin-60S ribosomal protein L40 | *Caenorhabditis elegans* | IQDKEGIPPDQQR | Unmod | 3 | 1522.77 | -1.75 | 471300 | 83 |
| P55038 | Ferredoxin-dependent glutamate synt... | *Synechocystis sp.* | PGEGGQLPGKKVSEYIAMLR | Deam (NQ) | 2 | 2130.11 | -3.47 | 1535900 | 82 |
| P56005 | Signal recognition particle protein | *Helicobacter pylori* | EQENPEILNGSR | Glu->pyro-Glu | 2 | 1366.65 | 1.89 | 1598800 | 89 |
| P59580 | Cytidylate kinase | *Buchnera aphidicola subsp. Baizongia pistaciae* | GINSCNYKK | 2 Deam (NQ) | 2 | 1084.49 | 1.09 | 1762300 | 53 |
| P60001 | Chlorate reductase assembly chapero... | *Ideonella dechloratans* | LGDQPQKSREMQ | Ox (M),2 Deam (NQ) | 2 | 1433.65 | -1.88 | 346120 | 69 |
| P71063 | UDP-N-acetylbacillosamine N-acetylt... | *Bacillus subtilis* | EWYTGPPK | Di-Ox W | 1 | 1008.46 | -3.80 | 620800 | 61 |
| P9WG49 | DNA topoisomerase 1 | *Mycobacterium tuberculosis* | ERDRMAFR | Ox (M),Glu->pyro-Glu | 2 | 1077.51 | -3.07 | 682750 | 50 |
| P9WNY1 | Probable aldehyde dehydrogenase | *Mycobacterium tuberculosis* | AVRQGDPLDTETMLGSQASNDQLEK | Ox (M),2 Deam (NQ) | 3 | 2720.25 | -0.16 | 6886300 | 53 |
| Q00513 | Type II secretion system protein F | *Pseudomonas aeruginosa* | EQSGQGGRLTFAR | Glu->pyro-Glu,Deam (NQ) | 2 | 1388.68 | 0.23 | 313770 | 54 |
| Q03DT9 | DNA ligase | *Pediococcus pentosaceus* | NAMNIMGLGPK | 2 Ox (M),Deam (NQ) | 2 | 1177.55 | -1.70 | 22614000 | 69 |
| Q09348 | Uncharacterized protein T05H10.4 | *Caenorhabditis elegans* | LQSKCRYR | Deam (NQ),O-2H (Y) | 2 | 1124.54 | -3.01 | 937280 | 107 |
| Q0BRH7 | Catalase-peroxidase | *Granulibacter bethesdensis* | SPGGAVQWKPKDASADQLVPDAFDASK | O-2H (W) | 3 | 2798.35 | -0.86 | 3637600 | 50 |
| Q1AVI6 | NADH-quinone oxidoreductase subunit... | *Rubrobacter xylanophilus* | DPSFVNMQLLQEK | Ox (M) | 2 | 1563.76 | -4.31 | 1257200 | 105 |
| Q1IX99 | DNA-directed RNA polymerase subunit... | *Deinococcus geothermalis* | DQKRPQLK | 2 Deam (NQ) | 2 | 1013.55 | -1.01 | 2520700 | 104 |
| Q1IY56 | Kynurenine formamidase | *Deinococcus geothermalis* | LEEVELNR | Unmod | 2 | 1000.52 | 0.94 | 3844200 | 143 |
| Q1MQ45 | Glycerol-3-phosphate dehydrogenase ... | *Lawsonia intracellularis* | NITNSMNMIAEGIK | 2 Ox (M),Deam (NQ) | 2 | 1567.72 | 1.59 | 17335000 | 74 |
| Q1R0J2 | L-lactate dehydrogenase | *Chromohalobacter salexigens* | QLDGVPSTAR | Gln->pyro-Glu | 2 | 1025.51 | -0.35 | 749690 | 74 |
| Q20655 | 14-3-3-like protein 2 | *Caenorhabditis elegans* | DSTLIMQLLR | Ox (M) | 2 | 1204.65 | 1.00 | 398590 | 99 |
| Q20875 | Pre-mRNA-splicing factor ATP-depend... | *Caenorhabditis elegans* | QIGAVGNMK | Ox (M),Deam (NQ) | 1 | 933.46 | -0.04 | 892880 | 71 |
| Q21412 | 3'-5' exoribonuclease parn-1 | *Caenorhabditis elegans* | IRAQNQATKIQR | 3 Deam (NQ) | 2 | 1428.77 | 0.10 | 250650 | 68 |
| Q27355 | Transcription factor lin-26 | *Caenorhabditis elegans* | LSNNKFNQMLSK | Ox (M),Deam (NQ) | 2 | 1439.71 | -3.56 | 2694000 | 87 |
| Q27532 | Histone H3.3-like type 2 | *Caenorhabditis elegans* | STELLLR | Unmod | 2 | 830.49 | -0.10 | 3085300 | 143 |
| Q2G7S5 | 1,4-alpha-glucan branching enzyme G... | *Novosphingobium aromaticivorans* | GVHFAVWAPNAR | Ox (W) | 2 | 1339.68 | 3.33 | 1164500 | 122 |
| Q2HJN9 | Elongation factor 1-alpha 4 | *Oscheius tipulae* | IGGIGTVPVGR | Unmod | 2 | 1024.60 | 2.21 | 3274000 | 184 |
| Q2JW63 | DNA ligase | *Synechococcus sp.* | EWQERLWR | Glu->pyro-Glu,Deam (NQ) | 2 | 1184.57 | -0.28 | 6419000 | 98 |
| Q3A091 | GTP cyclohydrolase FolE2 | *Pelobacter carbinolicus* | ARSLMEYR | Ox (M),Ox Y | 2 | 1056.50 | -4.10 | 2748700 | 95 |
| Q3YRX3 | Isoleucine--tRNA ligase | *Ehrlichia canis* | EWKQYVDR | Glu->pyro-Glu,diOx Y | 2 | 1136.53 | -4.04 | 1910800 | 63 |
| Q49V12 | Transcription-repair-coupling facto... | *Staphylococcus saprophyticus subsp. saprophyticus* | RYQELNEVFGK | Deam (NQ) | 2 | 1382.68 | -1.84 | 9178500 | 82 |
| Q59331 | Acetate kinase | *Thermoanaerobacterium thermosaccharolyticum* | VNVMVVPTNEEYMIAK | di-Ox (M) | 3 | 1867.91 | -1.12 | 3807800 | 66 |
| Q5E304 | tRNA-modifying protein YgfZ | *Aliivibrio fischeri* | SVGENWRK | Deam (NQ) | 2 | 975.48 | 1.31 | 604980 | 86 |
| Q5F5X0 | 1-deoxy-D-xylulose 5-phosphate redu... | *Neisseria gonorrhoeae* | QVEKLAAQCQTFR | 2 Deam (NQ) | 3 | 1579.77 | -2.65 | 1010900 | 82 |
| Q5FN05 | 50S ribosomal protein L9 | *Lactobacillus acidophilus* | EATKGNMNTLKR | Ox (M),Glu->pyro-Glu | 2 | 1359.69 | -3.40 | 1639800 | 72 |
| Q5SF94 | Appendage-associated protein | *Anaplasma marginale* | KELDAIDAECR | Unmod | 2 | 1318.62 | -3.86 | 3071400 | 116 |
| Q5Z3P6 | Lysylphosphatidylglycerol biosynthe... | *Nocardia farcinica* | VLIQNAALAANGSMVAMR | Ox (M) | 2 | 1844.96 | 1.08 | 1078500 | 84 |
| Q5ZUJ9 | Alanine--tRNA ligase | *Legionella pneumophila subsp. pneumophila* | VSQLLLDNKNQEK | 3 Deam (NQ) | 2 | 1530.78 | 0.88 | 807550 | 75 |
| Q61D31 | BAG family molecular chaperone regu... | *Caenorhabditis briggsae* | GFLEKPKQVEMGK | Acetyl (K) | 2 | 1531.81 | -1.40 | 30950000 | 146 |
| Q6DAM0 | Phosphomethylpyrimidine synthase | *Pectobacterium atrosepticum* | QGMITPEMEFIAIR | Ox (M),Gln->pyro-Glu | 2 | 1633.78 | -0.43 | 21313000 | 98 |
| Q6NF90 | tRNA(Ile)-lysidine synthase | *Corynebacterium diphtheriae* | AVDALITNWHGQK | Deam (NQ),Ox (W) | 2 | 1468.73 | 1.10 | 1773300 | 104 |
| Q6YPM1 | Chaperone protein DnaK | *Onion yellows phytoplasma* | NEAENMIFHTKK | Ox (M),Deam (NQ) | 2 | 1477.69 | 2.80 | 38048000 | 147 |
| Q7N8Q7 | NADPH-dependent 7-cyano-7-deazaguan... | *Photorhabdus laumondii subsp. laumondii* | DHQALEQLTLGK | Deam (NQ) | 3 | 1352.69 | 0.50 | 2733000 | 70 |
| Q7NRT5 | N-acetyl-gamma-glutamyl-phosphate r... | *Chromobacterium violaceum* | VDEMFPSLR | Ox (M) | 2 | 1108.52 | -1.39 | 2765400 | 118 |
| Q7U3P8 | tRNA uridine 5-carboxymethylaminome... | *Synechococcus sp.* | QQQQIDQVKR | 3 Deam (NQ) | 2 | 1272.63 | 2.45 | 4877100 | 125 |
| Q7V520 | 30S ribosomal protein S9 | *Prochlorococcus marinus* | MSSSNNSVVYWGTGR | Ox (M),O-2H (W) | 2 | 1673.71 | 4.01 | 3667900 | 91 |
| Q7VK64 | Phenylalanine--tRNA ligase alpha su... | *Helicobacter hepaticus* | LEQLENLNDLER | 3 Deam (NQ) | 2 | 1487.70 | -0.10 | 1429600 | 100 |
| Q88CS3 | Biosynthetic peptidoglycan transgly... | *Pseudomonas putida* | PSAYVASR | O-2H (Y) | 2 | 863.41 | -0.19 | 935970 | 88 |
| Q88U41 | ATP-dependent helicase/nuclease sub... | *Lactobacillus plantarum* | LESQINKR | Acetyl (K) | 2 | 1028.56 | 0.09 | 3239100 | 128 |
| Q88Y75 | Pantothenate kinase | *Lactobacillus plantarum* | DIYMPFAHLLQAK | Ox (M),Deam (NQ) | 2 | 1562.78 | -0.50 | 882670 | 79 |
| Q88YC0 | Xaa-Pro dipeptidyl-peptidase | *Lactobacillus plantarum* | GNQDLQYSLNLNDIRLHVPMK | 3 Deam (NQ) | 3 | 2470.22 | -2.60 | 8133700 | 53 |
| Q89U81 | Holliday junction ATP-dependent DNA... | *Bradyrhizobium diazoefficiens* | GLIDSYGEDFVILDVGGVGYQVHCSTR | Deam (NQ) | 3 | 2956.39 | 1.44 | 806470 | 45 |
| Q8D124 | Apolipoprotein N-acyltransferase | *Yersinia pestis* | AVNIAMVQGNIAQSMK | Ox (M),3 Deam (NQ) | 2 | 1692.81 | 1.33 | 708370 | 52 |
| Q8EQA8 | GTPase Der | *Oceanobacillus iheyensis* | AMKEFEKNIR | Acetyl (K),Ox (M) | 3 | 1322.67 | 0.64 | 6313600 | 80 |
| Q8F663 | LexA repressor | *Leptospira interrogans serogroup Icterohaemorrhagiae serovar Lai* | VQGDSMIEVGINDGDIAIIEK | Ox (M),Deam (NQ) | 2 | 2232.08 | -1.90 | 1388000 | 71 |
| Q8GP19 | Swarming motility regulation sensor... | *Serratia marcescens* | QPGNLSPINVSEQYQEIR | 3 Deam (NQ) | 2 | 2073.99 | -0.48 | 4312000 | 59 |
| Q8KCM7 | Lysine--tRNA ligase | *Chlorobaculum tepidum* | GKSEAQLR | Acetyl (K) | 2 | 929.49 | 0.25 | 8560500 | 147 |
| Q8PLF1 | Elongation factor P-like protein | *Xanthomonas axonopodis pv. citri* | ANDIKKGNVVEYNGGIYQIR | 2 Deam (NQ) | 3 | 2252.14 | 0.44 | 705690 | 57 |
| Q8YGV9 | Anhydro-N-acetylmuramic acid kinase | *Brucella melitensis biotype 1* | FDPGGATAMSGSVDR | Ox (M) | 3 | 1482.64 | 2.21 | 2631200 | 69 |
| Q8ZJV8 | Deoxyribose-phosphate aldolase | *Salmonella typhimurium* | KTLKEQGTPDIR | Acetyl (K) | 2 | 1426.78 | 0.30 | 6093700 | 120 |
| Q97KS6 | Spermidine/putrescine import ATP-bi... | *Clostridium acetobutylicum* | EMQLELKNIQQK | 2 Deam (NQ) | 2 | 1502.77 | 4.09 | 7515500 | 193 |
| Q9CB26 | Chaperone protein ClpB | *Mycobacterium leprae* | EQLETLRGESER | Glu->pyro-Glu,Deam (NQ) | 2 | 1428.68 | 1.14 | 181800 | 50 |
| Q9I472 | Corrinoid adenosyltransferase | *Pseudomonas aeruginosa* | QRDIAKAEAAWK | Gln->pyro-Glu | 2 | 1368.72 | 1.83 | 4162700 | 85 |
| Q9PBJ1 | 3-deoxy-D-manno-octulosonic acid ki... | *Xylella fastidiosa* | GNSKETHWEQIGR | Di-Ox W | 2 | 1572.73 | 1.98 | 4087400 | 76 |
| A0AFI3 | Rhamnulose-1-phosphate aldolase | *Listeria welshimeri serovar 6b* | NGWAEKNGGNISQLLEASEIK | Deam (NQ),Trp->Kynurenine | 2 | 2262.11 | 2.82 | 2597500 | 63 |
| A0KQV3 | ADP-L-glycero-D-manno-heptose-6-epi... | *Aeromonas hydrophila subsp. hydrophila* | YQSFTQADMTKLR | Ox (M),O-2H (Y) | 2 | 1617.75 | -2.94 | 851260 | 65 |
| A0LII4 | DNA-directed RNA polymerase subunit... | *Syntrophobacter fumaroxidans* | KDVSYLTAMDEK | Acetyl (K),di-Ox (M) | 2 | 1472.67 | -3.87 | 66929000 | 78 |
| A0Q125 | GTPase Der | *Clostridium novyi* | ECYGNYCKRIK | Glu->pyro-Glu,Deam (NQ) | 3 | 1472.65 | -3.27 | 927580 | 42 |
| A0Q556 | Methionine--tRNA ligase | *Francisella tularensis subsp. novicida* | GTFIQARTYLDNLEPSYLRYYFASR | Deam (NQ),diOx Y,O-2H (Y) | 3 | 3090.47 | -1.60 | 600090 | 44 |
| A0QM44 | Trans-aconitate 2-methyltransferase | *Mycobacterium avium* | GIDAVTGDLRNWK | Trp->Kynurenine | 2 | 1447.74 | -2.43 | 725930 | 74 |
| A0QZA1 | Universal stress protein MSMEG_3950... | *Mycolicibacterium smegmatis* | LAGWQER | Deam (NQ),Trp->Kynurenine | 2 | 863.41 | -0.56 | 935970 | 101 |
| A1AS39 | Ketol-acid reductoisomerase (NADP(+... | *Pelobacter propionicus* | GPRVVNEETKWEMK | Deam (NQ),Di-Ox W | 2 | 1734.83 | -2.52 | 1320000 | 49 |
| A1JML4 | Adenosine deaminase | *Yersinia enterocolitica serotype O:8 / biotype 1B* | ARDAGWRITVHAGEAAGPESIWQAIR | Di-Ox W | 3 | 2849.43 | -1.83 | 3791500 | 54 |
| A1JSF2 | HTH-type transcriptional regulator ... | *Yersinia enterocolitica serotype O:8 / biotype 1B* | GDLDNANMYIQR | Ox (M),Deam (NQ) | 2 | 1425.62 | -4.12 | 974330 | 45 |
| A1K4G2 | 23S rRNA (uracil(1939)-C(5))-methyl... | *Azoarcus sp.* | KADEAQLR | Unmod | 2 | 929.49 | 0.84 | 8392600 | 115 |
| A1SET2 | Cyclic pyranopterin monophosphate s... | *Nocardioides sp.* | GEGVPKGDALGVARVAGIMAAK | di-Ox (M) | 3 | 2098.12 | 1.03 | 12510000 | 62 |
| A1TSK3 | Translation initiation factor IF-2 | *Acidovorax citrulli* | GDASGGVGRNNWRSGPR | 2 Deam (NQ) | 2 | 1743.80 | 0.85 | 262040 | 50 |
| A3CQ18 | Translation initiation factor IF-2 | *Streptococcus sanguinis* | NSNWNKNKK | Deam (NQ),O-2H (W) | 2 | 1146.54 | 2.56 | 1856100 | 82 |
| A3PSZ4 | Uncharacterized methyltransferase M... | *Mycobacterium sp.* | LGNEMVR | Ox (M),Deam (NQ) | 1 | 834.39 | -2.09 | 736180 | 54 |
| A3QEV1 | D-alanine--D-alanine ligase | *Shewanella loihica* | VELDAKGQYQSK | diOx Y | 3 | 1396.68 | 2.04 | 1032000 | 68 |
| A3QGT8 | Peptide chain release factor 3 | *Shewanella loihica* | GLDNPELDEAIGSYAQDLR | diOx Y | 3 | 2106.97 | -4.05 | 1463200 | 42 |
| A4G5X2 | Trigger factor | *Herminiimonas arsenicoxydans* | VADKSVAFDELMGNNAQG | Deam (NQ) | 2 | 1865.85 | 2.42 | 446020 | 43 |
| A4IJG8 | Cysteine--tRNA ligase | *Geobacillus thermodenitrificans* | GLERLRTAYGNLQHR | diOx Y | 2 | 1814.95 | 0.76 | 1536700 | 113 |
| A4IJK9 | Adenylate kinase | *Geobacillus thermodenitrificans* | PDDNEATVANRLEVNTKQMK | Acetyl (K),3 Deam (NQ) | 3 | 2317.07 | -3.33 | 759670 | 49 |
| A4IST9 | Protein translocase subunit SecA 1 | *Geobacillus thermodenitrificans* | EDGEEPKKK | Acetyl (K),Glu->pyro-Glu | 2 | 1082.52 | -1.62 | 773970 | 81 |
| A4J0Y7 | Glutamate--tRNA ligase | *Desulfotomaculum reducens* | LTKLAVPYLQGAGYLK | 2 diOx Y | 2 | 1797.99 | -0.90 | 559230 | 72 |
| A4J5Y5 | 1-deoxy-D-xylulose 5-phosphate redu... | *Desulfotomaculum reducens* | QELENVTPEMALK | 2 Deam (NQ),di-Ox (M) | 2 | 1534.71 | 4.09 | 1332100 | 112 |
| A4JR39 | 1,4-alpha-glucan branching enzyme G... | *Burkholderia vietnamiensis* | VGLPAPGQWR | Deam (NQ),Trp->Kynurenine | 2 | 1084.57 | -2.66 | 1079300 | 88 |
| A4SF67 | Ribonuclease Y | *Chlorobium phaeovibrioides* | KQAQDNR | Acetyl (K),Deam (NQ) | 2 | 901.43 | 0.72 | 1469200 | 97 |
| A4SYD2 | ATP phosphoribosyltransferase regul... | *Polynucleobacter asymbioticus* | LRQWATCLPAK | Deam (NQ),Di-Ox W | 2 | 1375.69 | 4.21 | 12444000 | 119 |
| A4T5Z8 | 30S ribosomal protein S18 2 | *Mycolicibacterium gilvum* | TVTGLTVQQQR | 3 Deam (NQ) | 2 | 1232.63 | 1.85 | 690280 | 67 |
| A4W6T3 | tRNA(Ile)-lysidine synthase | *Enterobacter sp.* | RWLAAHHAQMPSRTMLNR | 2 Ox (M),2 Deam (NQ) | 3 | 2209.06 | 0.18 | 5928900 | 43 |
| A4W948 | Glyoxylate/hydroxypyruvate reductas... | *Enterobacter sp.* | QKSHWEPLADYQR | Deam (NQ),O-2H (W) | 2 | 1671.76 | 0.37 | 654420 | 54 |
| A5CXJ7 | Trigger factor | *Vesicomyosocius okutanii subsp. Calyptogena okutanii* | LTEYKAVQCKSK | Deam (NQ),TriOx (C) | 2 | 1502.73 | 3.71 | 767400 | 75 |
| A5FWJ5 | DNA ligase | *Acidiphilium cryptum* | SLAGLPPG | Unmod | 1 | 710.40 | -1.03 | 1072800 | 75 |
| A5FZV0 | 50S ribosomal protein L6 | *Acidiphilium cryptum* | ANVANMVQGVSQGYAK | Ox (M),3 Deam (NQ) | 2 | 1654.75 | 4.29 | 566640 | 66 |
| A5GVX6 | 30S ribosomal protein S5 | *Synechococcus sp.* | ERGITLEQIYS | Glu->pyro-Glu,diOx Y | 2 | 1321.65 | -0.51 | 5747400 | 52 |
| A5GX14 | 30S ribosomal protein S6 | *Synechococcus sp.* | YRDLVVEAGGEVIDSQMRGK | Ox (M),diOx Y | 3 | 2269.10 | 1.52 | 2101300 | 55 |
| A5IZH0 | Ribosome-binding factor A | *Mycoplasma agalactiae* | RLSKTLNWR | Deam (NQ),Di-Ox W | 2 | 1205.65 | 1.91 | 262490 | 44 |
| A6GWB6 | Aspartate--tRNA ligase | *Flavobacterium psychrophilum* | EIDALIEWVKR | Di-Ox W | 2 | 1402.75 | 1.74 | 1217300 | 110 |
| A6GYU1 | 50S ribosomal protein L7/L12 | *Flavobacterium psychrophilum* | ELTGLGLKEAKDLVDAAPSNVK | Acetyl (K),Glu->pyro-Glu | 3 | 2291.24 | 2.12 | 2219400 | 40 |
| A6LN93 | Peptide chain release factor 2 | *Thermosipho melanesiensis* | QLIDDIERKVK | Acetyl (K),Deam (NQ) | 2 | 1398.77 | 1.04 | 624110 | 102 |
| A6LZF4 | Virginiamycin B lyase | *Clostridium beijerinckii* | IGRISNLGEVIEYK | Deam (NQ),Iodination | 2 | 1716.76 | 1.83 | 837650 | 75 |
| A6VMS2 | Chaperone protein TorD | *Actinobacillus succinogenes* | LLILNWLR | Deam (NQ),O-2H (W) | 2 | 1054.62 | 1.83 | 5433800 | 91 |
| A6VUQ2 | 23S rRNA (uracil(1939)-C(5))-methyl... | *Marinomonas sp.* | NAWEDWAEK | Deam (NQ),Trp->Kynurenine | 2 | 1152.47 | 0.22 | 1361600 | 81 |
| A6W849 | tRNA dimethylallyltransferase | *Kineococcus radiotolerans* | PGEVVNADAMQLYR | 2 Deam (NQ),di-Ox (M) | 2 | 1595.71 | 3.50 | 1220900 | 49 |
| A7GKH8 | Phosphoribosylformylglycinamidine s... | *Bacillus cytotoxicus* | QAGDLIYVMGETK | Ox (M),Deam (NQ) | 2 | 1440.68 | 4.15 | 2095900 | 78 |
| A7NH85 | Glucose-6-phosphate isomerase | *Roseiflexus castenholzii* | GLRARIDAMFR | Ox (M) | 2 | 1320.71 | -2.34 | 1338700 | 72 |
| A8AZA7 | Proline--tRNA ligase | *Streptococcus gordonii* | AKNIMRQEFEK | Ox (M),2 Deam (NQ) | 2 | 1410.68 | -2.02 | 4867100 | 78 |
| A8EV98 | Putative membrane protein insertion... | *Arcobacter butzleri* | KNQNINYNK | Acetyl (K),2 Deam (NQ) | 2 | 1178.56 | -0.46 | 1264900 | 54 |
| A8EVH6 | Trigger factor | *Arcobacter butzleri* | VKEQMLAEDK | Acetyl (K),Ox (M) | 2 | 1247.61 | -0.19 | 4032100 | 88 |
| A8EVQ3 | Uroporphyrinogen decarboxylase | *Arcobacter butzleri* | YMVEIAEYLKEK | Ox (M),O-2H (Y) | 2 | 1544.74 | -0.69 | 942080 | 92 |
| A8EXD2 | Arginine--tRNA ligase | *Rickettsia canadensis* | AESWQIAIK | Deam (NQ),Trp->Kynurenine | 2 | 1049.54 | -0.39 | 1367200 | 111 |
| A8EZ86 | NADH-quinone oxidoreductase subunit... | *Rickettsia canadensis* | CRIKTPGFAHLQGLNFMSK | Acetyl (K),2 Deam (NQ) | 3 | 2248.11 | -0.75 | 581610 | 52 |
| A8FFW3 | 3-isopropylmalate dehydratase large... | *Bacillus pumilus* | RPQNTFATMDHNIPTVNR | 2 Deam (NQ) | 3 | 2113.00 | -2.59 | 1094100 | 46 |
| A8FSE9 | tRNA (guanine-N(1)-)-methyltransfer... | *Shewanella sediminis* | NGLLELQTWNPR | 2 Deam (NQ),O-2H (W) | 2 | 1455.70 | 1.14 | 4262500 | 59 |
| A8GKB6 | Glycerol-3-phosphate acyltransferas... | *Serratia proteamaculans* | SNPDLDIQMLPVSVMFGR | Ox (M) | 3 | 2033.99 | -0.02 | 339440 | 41 |
| A8GZ14 | CinA-like protein | *Shewanella pealeana* | ERLENWFTR | Glu->pyro-Glu,O-2H (W) | 2 | 1245.59 | 0.88 | 1667500 | 83 |
| A8LXY1 | Aspartate--tRNA(Asp/Asn) ligase | *Salinispora arenicola* | ELDGWQDWAKAR | Deam (NQ),O-2H (W) | 2 | 1488.66 | -4.42 | 205270 | 42 |
| A8WN14 | E3 UFM1-protein ligase 1 homolog | *Caenorhabditis briggsae* | MYDYLVK | Ox (M),diOx Y | 2 | 978.44 | 3.72 | 946940 | 55 |
| A8XJQ6 | cAMP-dependent protein kinase, cata... | *Caenorhabditis briggsae* | IGCMKNGTQDVKDHK | Acetyl (K),Deam (NQ),TriOx (C) | 2 | 1820.80 | 2.53 | 1909800 | 54 |
| A8XQC7 | Receptor-type guanylate cyclase gcy... | *Caenorhabditis briggsae* | PGADIICNLLKNMMPKK | TriOx (C),di-Ox (M) | 2 | 2022.00 | -3.57 | 1153600 | 53 |
| A8Z5T3 | DNA-directed RNA polymerase subunit... | *Sulcia muelleri* | GLTYNVYIR | 2 Ox Y | 2 | 1129.58 | 0.01 | 338800 | 53 |
| A9BHQ6 | Anthranilate phosphoribosyltransfer... | *Petrotoga mobilis* | YQLMGIYDPK | Deam (NQ),diOx Y,O-2H (Y) | 2 | 1273.55 | 4.47 | 495280 | 47 |
| A9BMK7 | Phosphoglucosamine mutase | *Delftia acidovorans* | LQPGQDWKR | Deam (NQ),Trp->Kynurenine | 2 | 1131.57 | 0.84 | 701170 | 53 |
| A9F1Y8 | Ribosomal protein S12 methylthiotra... | *Sorangium cellulosum* | VVPYVDMPLQHAADAMLR | Deam (NQ),2 di-Ox (M) | 2 | 2089.98 | 0.58 | 11572000 | 60 |
| A9IGW1 | Uracil-DNA glycosylase | *Bordetella petrii* | QGVLLLNTSLTVEDGQPASHAR | Deam (NQ) | 3 | 2306.19 | -2.63 | 2916800 | 43 |
| A9KYF8 | Alanine--tRNA ligase | *Shewanella baltica* | MYQTTAELR | Ox (M),Deam (NQ),O-2H (Y) | 2 | 1142.49 | 0.48 | 430740 | 49 |
| A9N9G2 | Arginine--tRNA ligase | *Coxiella burnetii* | HGYVYEK | diOx Y,O-2H (Y) | 2 | 940.39 | 3.54 | 663760 | 57 |
| B0TF89 | Aspartate--tRNA(Asp/Asn) ligase | *Heliobacterium modesticaldum* | GGDLRIDHAGQTVTLMGWVQRR | Deam (NQ),Di-Ox W | 3 | 2498.25 | -1.83 | 1146300 | 57 |
| B0UUG0 | Deoxyguanosinetriphosphate triphosp... | *Histophilus somni* | MKLQINSSWQER | 2 Deam (NQ),O-2H (W) | 2 | 1534.71 | 3.84 | 1493200 | 82 |
| B1GZ25 | Serine--tRNA ligase | *Endomicrobium trichonymphae* | MNIKVKYSQNK | Ox (M),Iodination | 2 | 1493.62 | -4.11 | 2951900 | 51 |
| B1KM58 | Protoheme IX farnesyltransferase 1 | *Shewanella woodyi* | SWELKFDDKPGLAMQVFR | Deam (NQ),Di-Ox W | 3 | 2199.07 | -2.43 | 8647800 | 51 |
| B1VS94 | Cysteine--tRNA ligase | *Streptomyces griseus subsp. griseus* | PGEPSWETPWGRGR | O-2H (W),Di-Ox W | 2 | 1656.73 | 1.75 | 1758100 | 52 |
| B1YJ17 | DNA ligase | *Exiguobacterium sibiricum* | VTHDLPMLSLGNVFDETEIR | Unmod | 3 | 2285.14 | -3.56 | 5302400 | 70 |
| B2A1H4 | Glutamate-1-semialdehyde 2,1-aminom... | *Natranaerobius thermophilus* | AGIETLKVLRQEGSYDQLESK | Deam (NQ),Ox Y | 3 | 2380.21 | 4.23 | 858470 | 44 |
| B2A2K2 | Methionyl-tRNA formyltransferase | *Natranaerobius thermophilus* | AVMDGKEQTGVTIMEMCDK | Acetyl (K),Ox (M),di-Ox (M) | 2 | 2231.94 | 4.40 | 1530700 | 40 |
| B2A826 | Translational regulator CsrA | *Natranaerobius thermophilus* | DVEIHRAEVYQK | Deam (NQ),Ox Y | 2 | 1502.74 | -1.78 | 633020 | 100 |
| B2HLS5 | Putative S-adenosyl-L-methionine-de... | *Mycobacterium marinum* | ALLGQWLNEHGWR | O-2H (W),Ox (W) | 2 | 1608.78 | 4.39 | 921720 | 50 |
| B2IJH6 | Dihydroxy-acid dehydratase | *Beijerinckia indica subsp. indica* | RSNSEMVQTFYK | Deam (NQ),Ox Y | 2 | 1505.68 | 0.97 | 519010 | 53 |
| B2TQR6 | ATP-dependent 6-phosphofructokinase | *Clostridium botulinum* | DALCRTILDGK | TriOx (C) | 2 | 1308.63 | -2.20 | 4847100 | 74 |
| B2VGW1 | p-hydroxybenzoic acid efflux pump s... | *Erwinia tasmaniensis* | NQLGVSAMSREAIEQANNDYQTTEHQLAK | Ox (M),3 Deam (NQ) | 3 | 3264.48 | 4.43 | 495540 | 40 |
| B3DVG8 | 50S ribosomal protein L27 | *Methylacidiphilum infernorum* | PGKNVGMGR | Ox (M),Deam (NQ) | 2 | 931.45 | -1.17 | 1429800 | 71 |
| B3EI42 | Biotin synthase | *Chlorobium limicola* | MTSSVIHQAVIDAYR | Ox (M),Deam (NQ),O-2H (Y) | 2 | 1720.81 | 3.46 | 2097800 | 61 |
| B3ET45 | Phosphoenolpyruvate carboxykinase (... | *Amoebophilus asiaticus* | VYIRDGYASALPEYK | 2 O-2H (Y) | 2 | 1771.84 | 3.16 | 39772000 | 81 |
| B3R0I8 | Threonine--tRNA ligase | *Phytoplasma mali* | KMSDHKYINK | O-2H (Y) | 3 | 1276.62 | 2.37 | 1486300 | 84 |
| B4S5G5 | Polyribonucleotide nucleotidyltrans... | *Prosthecochloris aestuarii* | DAQMIDTLTDDADKR | Ox (M) | 3 | 1722.77 | 1.99 | 1580100 | 53 |
| B4S9T4 | Argininosuccinate lyase | *Pelodictyon phaeoclathratiforme* | ADVSVNSKR | Acetyl (K),Deam (NQ) | 2 | 1017.51 | 0.65 | 2474700 | 107 |
| B4STF6 | Orotate phosphoribosyltransferase | *Stenotrophomonas maltophilia* | EAKAHGEGGQLIGADMNGK | Ox (M),Glu->pyro-Glu,Deam (NQ) | 2 | 1880.87 | 3.12 | 439480 | 50 |
| B5FR09 | Ribosomal RNA large subunit methylt... | *Salmonella dublin* | TGYYLDQR | diOx Y,O-2H (Y) | 2 | 1060.45 | -0.15 | 415950 | 54 |
| B5XYZ8 | Ketol-acid reductoisomerase (NADP(+... | *Klebsiella pneumoniae* | ANYFNTLNLRQQLAQLGKCR | Acetyl (K),Deam (NQ),TriOx (C) | 3 | 2498.23 | 3.51 | 1146300 | 53 |
| B5Y8F3 | tRNA (guanine-N(1)-)-methyltransfer... | *Coprothermobacter proteolyticus* | ESFTNNLLEEPQFTR | Glu->pyro-Glu,2 Deam (NQ) | 2 | 1807.83 | -2.16 | 720160 | 55 |
| B5YE43 | tRNA dimethylallyltransferase | *Dictyoglomus thermophilum* | GIDERFVSMQGIGYK | Ox (M),Ox Y | 2 | 1730.83 | -1.06 | 291340 | 57 |
| B5YHX5 | Hydroxylamine reductase | *Thermodesulfovibrio yellowstonii* | PGRSYYTEFVEK | 2 diOx Y | 2 | 1538.69 | -3.40 | 963420 | 82 |
| B6EJW7 | Lipid-A-disaccharide synthase | *Aliivibrio salmonicida* | SVKAQYPNAEFVGIGGPK | Acetyl (K),2 Deam (NQ) | 2 | 1904.95 | -1.04 | 305750 | 55 |
| B6EM99 | Nucleotide-binding protein VSAL_I04... | *Aliivibrio salmonicida* | EQIQHFLMTWLPALEK | Ox (M),Trp->Kynurenine | 2 | 2003.02 | 0.95 | 536310 | 69 |
| B7HQT6 | DNA-directed RNA polymerase subunit... | *Bacillus cereus* | ERIDYMDVSPK | Ox (M),Ox Y | 2 | 1383.63 | -1.94 | 3759400 | 68 |
| B7KC08 | N-(5'-phosphoribosyl)anthranilate i... | *Gloeothece citriformis* | VAELFQQLHR | Deam (NQ) | 3 | 1240.66 | -1.84 | 3271300 | 64 |
| B7VLR2 | Nucleoid-associated protein VS_0951 | *Vibrio atlanticus* | KQVADYCNEQLK | Acetyl (K),Deam (NQ) | 2 | 1537.71 | -1.14 | 2478300 | 75 |
| B8D0Y6 | Gamma-glutamyl phosphate reductase | *Halothermothrix orenii* | ENNVELRGCENTRAILPGIK | 2 Deam (NQ) | 3 | 2284.15 | 3.65 | 3258100 | 54 |
| B8DC21 | CCA-adding enzyme | *Listeria monocytogenes serotype 4a* | ETWLTDELYHAGK | Trp->Kynurenine | 2 | 1565.74 | -1.31 | 5343600 | 97 |
| B8I305 | Chaperone protein DnaK | *Ruminiclostridium cellulolyticum* | QAMQDAGLTPDK | Gln->pyro-Glu | 2 | 1256.57 | -2.37 | 578380 | 48 |
| B9E1H0 | Chromosome partition protein Smc | *Clostridium kluyveri* | QIENDKIKK | 2 Acetyl (K),Deam (NQ) | 2 | 1199.64 | 0.91 | 289070 | 95 |
| B9KE24 | Ribosome-recycling factor | *Campylobacter lari* | LNEIYTKQKQQSDK | 2 Deam (NQ) | 2 | 1723.86 | 2.91 | 1109600 | 54 |
| C0R0B8 | Gamma-glutamyl phosphate reductase | *Brachyspira hyodysenteriae* | LYNLFKENEIKMNADAEVK | Acetyl (K),Ox (M),Deam (NQ) | 3 | 2327.14 | 2.51 | 1103000 | 54 |
| C0Z6W4 | Translational regulator CsrA | *Brevibacillus brevis* | LGIDAPRNLDVYRK | Deam (NQ),Iodination | 2 | 1755.78 | 0.49 | 202400 | 44 |
| C1D5S5 | Malonyl-[acyl-carrier protein] O-me... | *Laribacter hongkongensis* | QAWQRIEAAYDR | 2 Deam (NQ),Ox Y | 2 | 1523.70 | 0.62 | 1367300 | 82 |
| C1F718 | Lipid-A-disaccharide synthase | *Acidobacterium capsulatum* | IYGEYLKLK | Ox Y,O-2H (Y) | 2 | 1155.62 | -3.49 | 2157000 | 86 |
| C3PMH7 | DNA-directed RNA polymerase subunit... | *Rickettsia africae* | DPANDEVLAKIGEMITADMLNVINDLK | Acetyl (K),di-Ox (M) | 3 | 3015.48 | -1.35 | 1134300 | 46 |
| C4L8W2 | Tetraacyldisaccharide 4'-kinase | *Tolumonas auensis* | LGNGWLMPMGPLR | Ox (M),Di-Ox W | 2 | 1488.72 | -0.52 | 894420 | 52 |
| C4Z2R7 | 30S ribosomal protein S7 | *Lachnospira eligens* | QALALRWMTTFSRAR | Deam (NQ),Trp->Kynurenine | 2 | 1811.95 | -4.32 | 1192200 | 79 |
| C4Z900 | Diaminopimelate epimerase | *Agathobacter rectalis* | VSIYVSDR | O-2H (Y) | 2 | 951.47 | 0.39 | 478620 | 89 |
| C5D8T2 | Phosphate acyltransferase | *Geobacillus sp.* | QSSMVLMAEEVKEGR | Ox (M),Gln->pyro-Glu | 2 | 1691.79 | -3.84 | 665860 | 46 |
| C5DAE0 | Probable malate:quinone oxidoreduct... | *Geobacillus sp.* | IGAPPMSVPHLDTRYIDNKK | Deam (NQ),di-Ox (M) | 3 | 2284.15 | -2.91 | 288700 | 55 |
| C6E2Q0 | Translation initiation factor IF-2 | *Geobacter sp.* | EYQRTAPGER | Glu->pyro-Glu,Deam (NQ) | 2 | 1188.55 | 1.17 | 669680 | 45 |
| E1X022 | Chromosome partition protein Smc | *Halobacteriovorax marinus* | AVSVQLH | Deam (NQ) | 1 | 753.40 | -0.47 | 5790100 | 59 |
| E7EAU8 | Receptor-type guanylate cyclase gcy... | *Caenorhabditis elegans* | NEEFVFVIPWLAHQNDHYPWEAANVDK | 3 Deam (NQ),Ox (W) | 4 | 3286.49 | -3.64 | 1187900 | 65 |
| E9P860 | NFX1-type zinc finger-containing pr... | *Caenorhabditis elegans* | KLIEEGLQKCETIYEK | Deam (NQ),diOx Y | 2 | 2013.00 | 0.74 | 5690100 | 101 |
| G5ECX0 | Latrophilin-like protein LAT-2 | *Caenorhabditis elegans* | ASIVRWLRTGICCLPETSSAAYNSR | Deam (NQ),Ox (W) | 3 | 2884.40 | 0.98 | 2038400 | 42 |
| G5EED4 | Protein nipi-3 | *Caenorhabditis elegans* | AEYHYRVR | Iodination,diOx Y | 2 | 1250.43 | -3.10 | 1456300 | 41 |
| O05268 | Ferredoxin--NADP reductase 2 | *Bacillus subtilis* | IRAQELINNLKEQMAK | Acetyl (K),Ox (M),2 Deam (NQ) | 2 | 1958.01 | 1.74 | 1098800 | 49 |
| O07006 | Phenolic acid decarboxylase PadC | *Bacillus subtilis* | EKYETYPK | Glu->pyro-Glu,O-2H (Y) | 2 | 1052.48 | -3.36 | 650610 | 57 |
| O16850 | Forkhead box protein O | *Caenorhabditis elegans* | QLEQKSSLHCSKCR | Acetyl (K),TriOx (C) | 2 | 1849.84 | -2.32 | 523130 | 48 |
| O16962 | Nuclear hormone receptor family mem... | *Caenorhabditis elegans* | VANLMKINNHIQLDIYR | Acetyl (K),2 Deam (NQ),di-Ox (M) | 2 | 2130.08 | 0.04 | 470290 | 47 |
| O25754 | Putative biopolymer transport prote... | *Helicobacter pylori* | ATQDKMIEIRMDK | 2 Ox (M),Deam (NQ) | 2 | 1610.76 | -3.62 | 730620 | 65 |
| O32127 | Putative antitoxin YutD | *Bacillus subtilis* | MILIQNAEFELVHNFKDGFNEEAFK | Ox (M),2 Deam (NQ) | 3 | 3000.42 | -3.76 | 869550 | 43 |
| O32243 | Glycine betaine/carnitine/choline-b... | *Bacillus subtilis* | LGVDNAWLK | Ox (W) | 2 | 1030.55 | -3.16 | 479960 | 110 |
| O32765 | L-lactate dehydrogenase | *Lactobacillus helveticus* | EQELMTASADQLKKVMDK | 2 Ox (M) | 2 | 2096.01 | 1.43 | 1047700 | 49 |
| O34843 | RNA polymerase sigma factor SigO | *Bacillus subtilis* | IVKYMNSMIR | Ox (M),di-Ox (M) | 2 | 1301.65 | 3.87 | 1228500 | 77 |
| O66516 | Uncharacterized protein aq_113 | *Aquifex aeolicus* | EGVGLHWGYAYLKPFK | Acetyl (K),Glu->pyro-Glu | 3 | 1887.97 | -2.72 | 585450 | 49 |
| O67026 | UPF0753 protein aq_863 | *Aquifex aeolicus* | EVMENLFSEFGK | Ox (M),Glu->pyro-Glu,Deam (NQ) | 2 | 1427.63 | 3.13 | 1747100 | 57 |
| O67679 | Probable GTP-binding protein EngB | *Aquifex aeolicus* | TRAVNYFLLDK | Deam (NQ),Ox Y | 2 | 1355.71 | 0.43 | 1985800 | 79 |
| O69192 | Aminopeptidase C | *Listeria monocytogenes serovar 1/2a* | MQTELTFEQLENFSR | 2 Deam (NQ) | 3 | 1873.84 | 4.12 | 471530 | 57 |
| O83195 | Proline--tRNA ligase | *Treponema pallidum* | LGDKYTR | diOx Y | 1 | 883.44 | -1.39 | 3689500 | 60 |
| O83809 | Threonine--tRNA ligase | *Treponema pallidum* | GLCVEENITMLQK | di-Ox (M) | 2 | 1565.74 | -3.78 | 5343600 | 100 |
| P0A0C4 | Replication protein | *Bacillus sp.* | INKNLVGFMR | Acetyl (K),Ox (M),Deam (NQ) | 2 | 1249.65 | -2.66 | 591790 | 85 |
| P0A211 | Protein FliZ | *Salmonella typhi* | IALRKYQQYK | diOx Y,O-2H (Y) | 2 | 1355.72 | 0.86 | 1499300 | 78 |
| P0CZ94 | ATP synthase subunit delta | *Streptococcus pyogenes serotype M3* | EQALIEQYGK | 2 Deam (NQ),Ox Y | 2 | 1195.56 | -4.20 | 392020 | 60 |
| P11568 | (R)-2-hydroxyglutaryl-CoA dehydrata... | *Acidaminococcus fermentans* | TSPLAQYNGALGAALYAYKK | Acetyl (K),2 Deam (NQ) | 3 | 2143.08 | 1.19 | 333240 | 71 |
| P13039 | Iron(III) enterobactin esterase | *Escherichia coli* | TALKVGSESWWQSKHGPEWQR | Deam (NQ),Ox (W) | 3 | 2513.21 | -3.40 | 1189400 | 62 |
| P17896 | SpoIVB peptidase | *Bacillus subtilis* | EYLLIPTQMR | Ox (M),Glu->pyro-Glu,Deam (NQ) | 2 | 1261.64 | -2.17 | 1040200 | 61 |
| P24467 | Cytochrome P450-pinF2, plant-induci... | *Rhizobium radiobacter* | QQCWGQAFEEGLRWVAPIQASSR | O-2H (W),Trp->Kynurenine | 3 | 2721.27 | 2.71 | 2165900 | 71 |
| P27128 | Lipopolysaccharide 1,3-galactosyltr... | *Escherichia coli* | PNNSNQLRYSAKHMLK | Acetyl (K),3 Deam (NQ) | 3 | 1944.94 | 0.21 | 709850 | 48 |
| P28249 | Protein AsmA | *Escherichia coli* | DYMVKQVAAR | Ox (M),Deam (NQ),Ox Y | 2 | 1212.58 | -1.80 | 3318800 | 59 |
| P29039 | Protein XpsM | *Xanthomonas campestris pv. campestris* | VQLQQAPQVSQR | 2 Deam (NQ) | 2 | 1382.72 | 2.30 | 7219700 | 78 |
| P29761 | Glucoamylase | *Clostridium sp.* | GYSIGYYKVNDIMTDLDENK | Ox (M),Iodination,Di-iodination,Ox Y | 4 | 2746.76 | 2.43 | 17013000 | 48 |
| P30193 | Uncharacterized protein in epiA 5'r... | *Staphylococcus epidermidis* | LDKSQFSKYEWR | Deam (NQ),Di-Ox W | 2 | 1618.76 | -3.70 | 458470 | 65 |
| P31517 | Hemin transport protein HemS | *Yersinia enterocolitica* | MSKSIYEQYLQAKADNPGK | Acetyl (K),Ox (M),Deam (NQ) | 3 | 2229.06 | -1.20 | 2602000 | 47 |
| P33696 | UTP--glucose-1-phosphate uridylyltr... | *Rhizobium meliloti* | SGKSAQISELEAMLPAAGSVSFTR | di-Ox (M) | 3 | 2468.22 | 3.58 | 5853000 | 58 |
| P34424 | Cyclin-T1.2 | *Caenorhabditis elegans* | EAAQQKIHR | Acetyl (K),Glu->pyro-Glu,Deam (NQ) | 2 | 1104.57 | 0.27 | 2186700 | 54 |
| P34430 | Uncharacterized protein F44B9.9 | *Caenorhabditis elegans* | SHNSLDHRNMRLSK | Deam (NQ),di-Ox (M) | 2 | 1726.82 | -0.61 | 2429300 | 75 |
| P35655 | Hypersensitivity response secretion... | *Pseudomonas syringae pv. syringae* | TAAVAPEMNGK | di-Ox (M) | 2 | 1119.52 | -1.86 | 1774500 | 93 |
| P37588 | Flagellar biosynthetic protein FliV | *Salmonella muenchen* | TSLVLQMQNYFR | 2 Deam (NQ),di-Ox (M) | 2 | 1532.72 | -4.12 | 829950 | 67 |
| P39636 | Amino-acid permease RocC | *Bacillus subtilis* | MQNHKNELQRSMK | Acetyl (K),Ox (M),Deam (NQ) | 2 | 1701.79 | 2.49 | 535740 | 57 |
| P42573 | Cell death protein 3 | *Caenorhabditis elegans* | PQVQQVWR | 2 Deam (NQ),O-2H (W) | 2 | 1055.50 | -3.13 | 1109200 | 84 |
| P43158 | Thiol protease/hemagglutinin PrtT | *Porphyromonas gingivalis* | NTISILYRTDGMADWKELK | Acetyl (K),Ox (M) | 3 | 2311.15 | -0.33 | 645920 | 52 |
| P44206 | Molybdate-binding protein MolA | *Haemophilus influenzae* | KQLGKNYVR | Deam (NQ),Iodination | 2 | 1231.52 | -3.51 | 500610 | 41 |
| P44847 | Putative cell division protein FtsP | *Haemophilus influenzae* | AEILVNMMK | 2 Ox (M),Deam (NQ) | 2 | 1080.52 | 2.12 | 10185000 | 49 |
| P45181 | Probable zinc protease PqqL | *Haemophilus influenzae* | LTQLNEKQLNIR | Acetyl (K),Deam (NQ) | 2 | 1511.83 | -0.47 | 756230 | 111 |
| P52016 | Peptidyl-prolyl cis-trans isomerase... | *Caenorhabditis elegans* | EKEEAERNAR | Acetyl (K),Glu->pyro-Glu | 2 | 1254.60 | -2.60 | 7579600 | 79 |
| P55496 | Uncharacterized protein y4iM | *Sinorhizobium fredii* | PNGYFYPPAYFGK | Deam (NQ),2 Di-iodination,diOx Y | 3 | 2056.27 | 4.20 | 13121000 | 41 |
| P57519 | Leucine--tRNA ligase | *Buchnera aphidicola subsp. Acyrthosiphon pisum* | IENYVQEFWKK | Deam (NQ),diOx Y | 2 | 1515.73 | 0.96 | 939770 | 68 |
| P57621 | UPF0265 protein BU556 | *Buchnera aphidicola subsp. Acyrthosiphon pisum* | KIIFMMKSDYEDR | Acetyl (K),Ox (M),di-Ox (M) | 2 | 1764.81 | 1.12 | 387660 | 52 |
| P58010 | Uncharacterized protein TM_1467.1 | *Thermotoga maritima* | NVLKYNYK | Deam (NQ),Ox Y,diOx Y | 2 | 1089.53 | -3.67 | 8093100 | 91 |
| P59031 | 30S ribosomal protein S8 | *Chlorobaculum tepidum* | EAREQNVGGEVLFR | Glu->pyro-Glu,Deam (NQ) | 2 | 1585.79 | -4.47 | 1080500 | 67 |
| P59739 | Adenylate cyclase | *Shigella flexneri* | MYLYIETLK | Ox (M),O-2H (Y) | 2 | 1202.59 | 3.38 | 528820 | 50 |
| P69969 | Protein TyeA | *Yersinia pseudotuberculosis serotype I* | VRFYQDLKR | Deam (NQ),Ox Y | 3 | 1240.66 | -3.47 | 4137000 | 51 |
| P74007 | Probable guanosine-3',5'-bis(diphos... | *Synechocystis sp.* | QLLDWQSDLK | Di-Ox W | 3 | 1276.63 | -3.73 | 2010800 | 92 |
| P76056 | Prophage integrase IntR | *Escherichia coli* | RNLTILDMFGPPK | Ox (M) | 2 | 1516.81 | 3.91 | 3491200 | 112 |
| P77810 | Lon protease | *Azospirillum brasilense* | VGESQELEALGRAVVSQFEQYIK | Iodination | 3 | 2705.22 | 3.45 | 7382900 | 49 |
| P9WNR3 | ESX-3 secretion system ATPase EccB3 | *Mycobacterium tuberculosis* | NNNSYGLQQPPR | Deam (NQ) | 2 | 1387.65 | 2.86 | 329100 | 87 |
| Q00014 | rRNA adenine N-6-methyltransferase | *Lactobacillus reuteri* | SYKIFGNIPYNISTDIIR | Deam (NQ) | 3 | 2114.11 | 1.76 | 27115000 | 75 |
| Q02D35 | tRNA uridine 5-carboxymethylaminome... | *Solibacter usitatus* | GPAVWSPRAQMDK | Deam (NQ),O-2H (W) | 2 | 1456.68 | -2.16 | 5927700 | 74 |
| Q02YY5 | Dihydroxy-acid dehydratase | *Lactococcus lactis subsp. cremoris* | EDECDEIGLAIKNLLEKDIK | Glu->pyro-Glu,Deam (NQ) | 3 | 2327.16 | -3.41 | 1504700 | 48 |
| Q03FX2 | Phosphate acyltransferase | *Pediococcus pentosaceus* | KALPDVEFILYGK | O-2H (Y) | 2 | 1505.81 | -3.55 | 894170 | 54 |
| Q04508 | Ammonia monooxygenase beta subunit | *Nitrosomonas europaea* | TVQWYDIK | O-2H (Y) | 2 | 1065.51 | -0.58 | 751270 | 82 |
| Q04855 | Uncharacterized protein AZC_3085 | *Azorhizobium caulinodans* | GIAATCVIDWRR | Ox (W) | 2 | 1432.73 | -2.70 | 684850 | 69 |
| Q049W4 | Heat-inducible transcription repres... | *Lactobacillus delbrueckii subsp. bulgaricus* | TVMNQLSIK | Ox (M),Deam (NQ) | 2 | 1049.54 | -0.48 | 1488700 | 109 |
| Q04CG8 | Phosphonates import ATP-binding pro... | *Lactobacillus delbrueckii subsp. bulgaricus* | AEQPMIEMKNVTK | Ox (M),Deam (NQ) | 2 | 1534.74 | -2.10 | 1529900 | 95 |
| Q04Z87 | Enolase-phosphatase E1 | *Leptospira borgpetersenii serovar Hardjo-bovis* | PGNAPQPK | 2 Deam (NQ) | 1 | 809.39 | 2.00 | 279940 | 41 |
| Q05865 | Dihydrofolate synthase/folylpolyglu... | *Bacillus subtilis* | DKPYQNMIKR | Ox (M),2 Deam (NQ) | 2 | 1309.63 | -0.48 | 2159200 | 80 |
| Q07HT2 | Protein translocase subunit SecA 2 | *Rhodopseudomonas palustris* | VAATLEANGWQRSRDR | Deam (NQ),Di-Ox W | 2 | 1861.90 | -4.48 | 765740 | 63 |
| Q07KM1 | 50S ribosomal protein L2 | *Rhodopseudomonas palustris* | RTKVDVPAK | Acetyl (K) | 2 | 1054.61 | 0.57 | 3097400 | 135 |
| Q0AFP4 | Glutamyl-tRNA(Gln) amidotransferase... | *Nitrosomonas eutropha* | EAQMLNAAHR | Glu->pyro-Glu,Deam (NQ) | 1 | 1122.52 | 3.56 | 296570 | 49 |
| Q0P8J9 | Cytidine diphosphoramidate kinase | *Campylobacter jejuni subsp. jejuni serotype O:2* | EILGHYAYDRQGRIDMALK | O-2H (Y) | 2 | 2262.12 | -2.84 | 2597500 | 73 |
| Q0PAS0 | Fructose-bisphosphate aldolase | *Campylobacter jejuni subsp. jejuni serotype O:2* | PGNVSLQPEILKNSQKFVK | Acetyl (K),3 Deam (NQ) | 2 | 2170.15 | -2.51 | 1016400 | 49 |
| Q0S0J3 | Triosephosphate isomerase | *Rhodococcus jostii* | EAGEHVSYNVEQLR | Glu->pyro-Glu,Ox Y | 2 | 1627.76 | 3.78 | 810180 | 54 |
| Q0S766 | Phosphoribosylformylglycinamidine s... | *Rhodococcus jostii* | KWITEQYDR | O-2H (W) | 2 | 1251.59 | -1.12 | 3373500 | 75 |
| Q0SFF3 | Elongation factor G | *Rhodococcus jostii* | MAQEVLTDLNK | Ox (M) | 3 | 1276.63 | -3.94 | 1486300 | 91 |
| Q0SQC1 | Cysteine--tRNA ligase | *Clostridium perfringens* | MIKKANEEGITVK | Acetyl (K),Ox (M) | 2 | 1517.81 | 1.41 | 4218800 | 132 |
| Q0VRS0 | Phosphoenolpyruvate carboxylase | *Alcanivorax borkumensis* | QLEAQLADR | Gln->pyro-Glu | 2 | 1025.51 | 2.50 | 749690 | 56 |
| Q0VSE5 | Glutamate 5-kinase | *Alcanivorax borkumensis* | GLDCALMQTWVDR | Ox (M),Ox (W) | 2 | 1595.71 | -0.39 | 2840100 | 73 |
| Q11HS3 | Adenylate kinase | *Chelativorans sp.* | RAEDAQAAGQPVRR | 2 Deam (NQ) | 2 | 1525.76 | -2.94 | 719280 | 84 |
| Q11PQ4 | Glutamate--tRNA ligase | *Cytophaga hutchinsonii* | VASPSYNMVTR | Ox (M),Deam (NQ),O-2H (Y) | 2 | 1254.56 | -3.66 | 678600 | 51 |
| Q11R22 | Fructose-1,6-bisphosphatase class 1 | *Cytophaga hutchinsonii* | MERNLSTVK | Ox (M),Deam (NQ) | 2 | 1093.54 | -1.39 | 365940 | 79 |
| Q12SX8 | Gamma-glutamyl phosphate reductase | *Shewanella denitrificans* | NTVLLDMAKSLR | Acetyl (K),Deam (NQ) | 2 | 1402.75 | -0.91 | 1217300 | 98 |
| Q137C6 | Lipoyl synthase | *Rhodopseudomonas palustris* | TGLPGALDPNEPAYVAEATR | Deam (NQ),O-2H (Y) | 3 | 2055.98 | -3.41 | 30852000 | 100 |
| Q13WF7 | Threonine--tRNA ligase | *Paraburkholderia xenovorans* | RVNDAGYLEIK | Deam (NQ),Ox Y | 2 | 1293.66 | 0.41 | 2136700 | 76 |
| Q13XC6 | UDP-3-O-acylglucosamine N-acyltrans... | *Paraburkholderia xenovorans* | IGADSHLYPNVAVYYGCK | 2 diOx Y | 2 | 2089.94 | 2.69 | 921920 | 63 |
| Q19542 | Cytoplasmic dynein 2 heavy chain 1 | *Caenorhabditis elegans* | TAADWESQFKILK | Deam (NQ),Ox (W) | 2 | 1552.78 | -0.67 | 926740 | 81 |
| Q1CW50 | CRISPR-associated exonuclease Cas4/... | *Myxococcus xanthus* | EGTLVVYEHKRGR | Glu->pyro-Glu,diOx Y | 2 | 1556.81 | -3.00 | 1526400 | 69 |
| Q1GKK5 | Polyribonucleotide nucleotidyltrans... | *Ruegeria sp.* | VGYEDGEYILNPTVDDMQDLRLNPEQR | Ox Y | 3 | 3194.48 | 1.30 | 353220 | 42 |
| Q1IMC7 | Phosphate import ATP-binding protei... | *Koribacter versatilis* | SLEASALWNEVK | Deam (NQ),Ox (W) | 2 | 1362.67 | -3.90 | 1195200 | 96 |
| Q1IVL5 | tRNA uridine 5-carboxymethylaminome... | *Koribacter versatilis* | GPAVWSPRAQCDKQQYR | Deam (NQ),TriOx (C) | 2 | 2094.95 | -0.22 | 1437200 | 42 |
| Q1QX67 | UPF0434 protein Csal_1588 | *Chromohalobacter salexigens* | ELLAMLVCPRCQGKLK | Deam (NQ),TriOx (C),di-Ox (M) | 2 | 1995.98 | -2.99 | 21077000 | 122 |
| Q1RH01 | 30S ribosomal protein S2 | *Rickettsia bellii* | KEILDMNR | Acetyl (K),Ox (M) | 2 | 1075.53 | -2.07 | 1380300 | 48 |
| Q1RHT1 | Tyrosine recombinase XerD | *Rickettsia bellii* | ALSKNSILSYKR | O-2H (Y) | 3 | 1392.77 | 0.11 | 749460 | 76 |
| Q1RJJ2 | Putative membrane protein insertion... | *Rickettsia bellii* | EALNTHGGIK | Glu->pyro-Glu,Deam (NQ) | 2 | 1021.52 | 0.06 | 983810 | 70 |
| Q20060 | Structural maintenance of chromosom... | *Caenorhabditis elegans* | ESMLADRLK | Ox (M),Glu->pyro-Glu | 2 | 1059.54 | -0.45 | 592920 | 73 |
| Q21JL6 | Carboxy-S-adenosyl-L-methionine syn... | *Saccharophagus degradans* | TNGYSELEIAQKR | Deam (NQ),O-2H (Y) | 2 | 1522.73 | -3.96 | 1246000 | 70 |
| Q21JS2 | Na(+)-translocating NADH-quinone re... | *Saccharophagus degradans* | PTMNVQVGDKVK | Acetyl (K),Ox (M),Deam (NQ) | 2 | 1373.69 | -0.19 | 1946600 | 103 |
| Q22616 | Biogenesis of lysosome-related orga... | *Caenorhabditis elegans* | AYEAKNPPLPPNQANPASH | 2 Deam (NQ),diOx Y | 2 | 2048.94 | 3.02 | 1354700 | 77 |
| Q24Q31 | Cobalt-precorrin-5B C(1)-methyltran... | *Desulfitobacterium hafniense* | MGGAADMRKK | Ox (M) | 2 | 1079.52 | 1.95 | 1079300 | 71 |
| Q250N7 | 30S ribosomal protein S12 | *Desulfitobacterium hafniense* | STAPAMQWGYNSLQR | Ox (M),Deam (NQ),O-2H (Y) | 2 | 1739.76 | 2.96 | 677230 | 45 |
| Q2GBX1 | Threonine--tRNA ligase | *Novosphingobium aromaticivorans* | EVKTPQVMDAR | Ox (M),Glu->pyro-Glu,Deam (NQ) | 2 | 1271.62 | 0.25 | 1796300 | 53 |
| Q2GEB1 | Threonine--tRNA ligase | *Neorickettsia sennetsu* | IPPEEKISIYKQGDFIDLCR | Acetyl (K),TriOx (C) | 3 | 2510.24 | 2.91 | 1710200 | 55 |
| Q2K711 | Probable potassium transport system... | *Rhizobium etli* | TPNTGLPGWQER | Deam (NQ),Trp->Kynurenine | 2 | 1359.64 | 0.58 | 813740 | 41 |
| Q2LX49 | Deoxyguanosinetriphosphate triphosp... | *Syntrophus aciditrophicus* | YVMDLYMMMFEPYEK | Iodination,Di-iodination | 3 | 2366.53 | 0.01 | 3232700 | 42 |
| Q2RFQ0 | 50S ribosomal protein L2 | *Moorella thermoacetica* | PGKGGQMARSAGAGAQLMAK | Ox (M),2 Deam (NQ) | 2 | 1903.92 | 0.89 | 945400 | 50 |
| Q2RYT3 | Phenylalanine--tRNA ligase beta sub... | *Salinibacter ruber* | YVALLVRGVDVTESPLWLR | Iodination | 3 | 2311.12 | -0.77 | 837050 | 43 |
| Q2S0U3 | Erythronate-4-phosphate dehydrogena... | *Salinibacter ruber* | LGDGAWLLNTSR | Deam (NQ),Trp->Kynurenine | 2 | 1306.65 | 1.88 | 708180 | 75 |
| Q2SMS4 | 5-methyltetrahydropteroyltriglutama... | *Hahella chejuensis* | ISQEELLNVGAELRQR | Deam (NQ) | 3 | 1854.98 | -3.71 | 2310800 | 69 |
| Q2SRA7 | Deoxyribose-phosphate aldolase | *Mycoplasma capricolum subsp. capricolum* | AANDHVVKVILENCLLTR | TriOx (C) | 3 | 2112.10 | 0.09 | 196400 | 52 |
| Q39I70 | Pyridoxine 5'-phosphate synthase | *Burkholderia lata* | AACKQLADVGVR | Acetyl (K),TriOx (C) | 3 | 1376.67 | -0.01 | 862540 | 68 |
| Q3AFF4 | UvrABC system protein B | *Carboxydothermus hydrogenoformans* | RDVIIVASVSCIYGLGDPQEYR | Deam (NQ) | 3 | 2510.25 | -3.86 | 1710200 | 46 |
| Q3IEW3 | Diaminopimelate epimerase | *Pseudoalteromonas translucida* | ANIGFMQVISKEHIK | Acetyl (K),2 Deam (NQ),di-Ox (M) | 2 | 1789.89 | -2.09 | 474280 | 83 |
| Q3IK30 | Bifunctional protein GlmU | *Pseudoalteromonas translucida* | PGAIMEEDSHVGNFVEMKK | Ox (M) | 2 | 2132.99 | -3.71 | 1208300 | 49 |
| Q3K4R6 | Tyrosine recombinase XerC | *Pseudomonas fluorescens* | GLYHYLNRECLCDHDPATGLAPPK | Unmod | 3 | 2796.31 | 3.36 | 2592100 | 47 |
| Q3KLJ1 | 50S ribosomal protein L17 | *Chlamydia trachomatis serovar A* | EARQVKAGDLSAYNVDR | Glu->pyro-Glu,Deam (NQ) | 2 | 1873.93 | -3.24 | 927650 | 67 |
| Q3SLK1 | 50S ribosomal protein L13 | *Thiobacillus denitrificans* | GLDKKYYR | diOx Y,O-2H (Y) | 2 | 1087.53 | -0.44 | 643750 | 49 |
| Q3YRR6 | Chaperone protein DnaK | *Ehrlichia canis* | IINEPTAAALAYGLNK | Deam (NQ) | 2 | 1658.89 | 0.95 | 1161900 | 104 |
| Q3Z556 | 2-keto-4-pentenoate hydratase | *Shigella sonnei* | PAGLDLKNCAMKMTR | Acetyl (K),TriOx (C),di-Ox (M) | 2 | 1826.83 | 2.93 | 535550 | 47 |
| Q3Z9L3 | tRNA (guanine-N(1)-)-methyltransfer... | *Dehalococcoides mccartyi* | GWDIPEVLLSGNHAR | O-2H (W) | 3 | 1676.83 | -3.60 | 3921900 | 67 |
| Q3ZZT8 | ATP synthase gamma chain | *Dehalococcoides mccartyi* | AMEMIAASKMKK | 2 Ox (M) | 2 | 1369.68 | -1.93 | 908420 | 52 |
| Q44118 | GTP 3',8-cyclase | *Paenarthrobacter nicotinovorans* | WQDAMWLK | Ox (M),Trp->Kynurenine | 2 | 1096.50 | 4.29 | 736350 | 56 |
| Q46149 | Toxin A | *Clostridium novyi* | GEQLTLV | Deam (NQ) | 1 | 759.40 | 0.01 | 6871800 | 50 |
| Q46GU9 | Glutamate--tRNA ligase | *Prochlorococcus marinus* | NGLAPKYDNR | Deam (NQ),O-2H (Y) | 2 | 1161.54 | 1.28 | 2914700 | 128 |
| Q46RR4 | Serine hydroxymethyltransferase 2 | *Cupriavidus pinatubonensis* | MSNTQSFFSQPLAER | Ox (M),3 Deam (NQ) | 2 | 1760.76 | 2.45 | 1564600 | 45 |
| Q47BK5 | Isoleucine--tRNA ligase | *Dechloromonas aromatica* | ALGKILEQGYLYQGLK | Deam (NQ),2 O-2H (Y) | 2 | 1821.95 | -2.21 | 472800 | 58 |
| Q47WP5 | Ribonuclease 3 | *Colwellia psychrerythraea* | PGNEQKDPK | Acetyl (K),Deam (NQ) | 1 | 1054.49 | 1.48 | 812230 | 48 |
| Q47Z09 | Ribosomal RNA large subunit methylt... | *Colwellia psychrerythraea* | QAPAINRQSWGFEAWLSHDNAVWQK | 2 Deam (NQ),Di-Ox W | 3 | 2972.38 | 3.30 | 1685700 | 40 |
| Q493H3 | Queuine tRNA-ribosyltransferase | *Blochmannia pennsylvanicus* | LMEELRQAIKTK | Ox (M) | 2 | 1474.82 | -1.17 | 257130 | 72 |
| Q4A5D7 | 50S ribosomal protein L18 | *Mycoplasma synoviae* | SGKYGGNVAAAK | Deam (NQ),Ox Y | 2 | 1138.56 | 3.39 | 630970 | 53 |
| Q4A5S9 | Glucose-6-phosphate isomerase | *Mycoplasma synoviae* | MEEKVAQWLKDK | Deam (NQ),O-2H (W) | 2 | 1518.74 | -0.74 | 471810 | 106 |
| Q4A667 | Ribosomal RNA small subunit methylt... | *Mycoplasma synoviae* | NYFQELTK | Deam (NQ),Ox Y | 2 | 1058.49 | -0.29 | 1865400 | 81 |
| Q4FVB5 | Peptidyl-tRNA hydrolase | *Psychrobacter arcticus* | LLMPLTFMNKSGQSVVPMVK | Ox (M),2 di-Ox (M) | 3 | 2299.16 | 3.85 | 6806400 | 70 |
| Q4L4Y7 | Coenzyme A disulfide reductase | *Staphylococcus haemolyticus* | IIVVGAVAGGATCASQIRR | TriOx (C) | 2 | 1946.04 | 0.77 | 3692400 | 61 |
| Q4L7L3 | Aspartyl/glutamyl-tRNA(Asn/Gln) ami... | *Staphylococcus haemolyticus* | GADVKLTSNWLMGGVNEYLNK | Deam (NQ),Trp->Kynurenine | 3 | 2313.13 | -3.29 | 784040 | 60 |
| Q53584 | Sodium/proline symporter | *Staphylococcus aureus* | QATGNLSEYMLGGR | Ox (M),diOx Y | 2 | 1543.69 | 4.49 | 3307800 | 76 |
| Q55242 | Levansucrase | *Streptococcus salivarius* | MTNQGDWIWDK | O-2H (W),Ox (W) | 2 | 1422.59 | 4.21 | 726260 | 60 |
| Q55738 | DNA gyrase subunit A | *Synechocystis sp.* | YELRKAEER | Acetyl (K) | 2 | 1234.63 | -2.50 | 4689600 | 113 |
| Q5GTB0 | 4-diphosphocytidyl-2-C-methyl-D-ery... | *Wolbachia sp. subsp. Brugia malayi* | ISNKYNTVMR | 2 Deam (NQ),O-2H (Y) | 2 | 1240.58 | -3.77 | 782500 | 49 |
| Q5KWP6 | UPF0735 ACT domain-containing prote... | *Geobacillus kaustophilus* | GVFYKYR | 2 O-2H (Y) | 2 | 959.45 | -3.64 | 366670 | 53 |
| Q5MZY1 | Arginine biosynthesis bifunctional ... | *Synechococcus sp.* | MMQAAWQEISGGLTAPR | Ox (M),Deam (NQ),Trp->Kynurenine | 2 | 1866.86 | 2.57 | 1136200 | 64 |
| Q5NG38 | GMP synthase [glutamine-hydrolyzing... | *Francisella tularensis subsp. tularensis* | KLGLGLGLPYNMLYR | Acetyl (K),Deam (NQ),di-Ox (M) | 2 | 1781.94 | -0.72 | 678400 | 65 |
| Q5ZXN6 | Phosphocholine transferase AnkX | *Legionella pneumophila subsp. pneumophila* | LGNNILHSAMRR | Deam (NQ),di-Ox (M) | 2 | 1413.72 | -1.14 | 992780 | 75 |
| Q60QM8 | DOMON domain-containing protein CBG... | *Caenorhabditis briggsae* | YDSSGFQSYWR | Deam (NQ),Trp->Kynurenine | 2 | 1399.57 | -0.74 | 910780 | 81 |
| Q619W7 | Beta-hexosaminidase A | *Caenorhabditis briggsae* | AENAWPRMHELR | Deam (NQ),O-2H (W) | 2 | 1523.69 | 2.57 | 1367300 | 74 |
| Q63QL1 | Cell division protein ZapD | *Burkholderia pseudomallei* | AVIPGGTCKFDLPSYYAWQQWPAEQR | Deam (NQ),Ox Y,diOx Y | 3 | 3116.43 | -2.98 | 2104900 | 43 |
| Q63TF9 | Alanine--tRNA ligase | *Burkholderia pseudomallei* | DAQGNMTRLPK | Deam (NQ),di-Ox (M) | 2 | 1262.59 | 2.12 | 1965700 | 86 |
| Q650L8 | tRNA pseudouridine synthase B | *Bacteroides fragilis* | NFKEGEVLYFNK | diOx Y | 2 | 1518.74 | -0.79 | 471810 | 106 |
| Q65S78 | 3-phosphoshikimate 1-carboxyvinyltr... | *Mannheimia succiniciproducens* | QLGVNYSLSEDK | 2 Deam (NQ) | 2 | 1353.63 | 0.94 | 846380 | 74 |
| Q65T53 | Sulfite reductase [NADPH] flavoprot... | *Mannheimia succiniciproducens* | DQAEKIYVQDK | diOx Y | 2 | 1367.66 | -0.99 | 1158300 | 97 |
| Q67KN9 | NADH-quinone oxidoreductase subunit... | *Symbiobacterium thermophilum* | KNLPYAIYDR | Ox Y,O-2H (Y) | 2 | 1281.64 | -2.74 | 2826500 | 62 |
| Q6F0U3 | GTPase Obg | *Mesoplasma florum* | SELVKYNYK | diOx Y,O-2H (Y) | 2 | 1188.57 | 4.23 | 307330 | 51 |
| Q6F1J8 | Glycine--tRNA ligase | *Mesoplasma florum* | IQINKENYR | 2 Deam (NQ),O-2H (Y) | 2 | 1192.57 | 0.20 | 1071000 | 87 |
| Q6FDS2 | Aspartate/glutamate leucyltransfera... | *Acinetobacter baylyi* | SYQPKSLLNDLQYYITPPHDCSYLDNK | Deam (NQ),Ox Y,diOx Y | 3 | 3320.52 | 2.98 | 878630 | 44 |
| Q6FF64 | UvrABC system protein C | *Acinetobacter baylyi* | QCENSYFAQR | 3 Deam (NQ) | 2 | 1304.50 | -2.76 | 15646000 | 85 |
| Q6FYZ7 | Glycine dehydrogenase (decarboxylat... | *Bartonella quintana* | LSQAYSILYR | Deam (NQ),Ox Y,diOx Y | 2 | 1261.62 | 0.09 | 982520 | 80 |
| Q6N1B7 | Chromosome partition protein Smc | *Rhodopseudomonas palustris* | KSALAEAQSR | Acetyl (K),Deam (NQ) | 2 | 1102.56 | 1.09 | 6119600 | 84 |
| Q72RT5 | Bifunctional purine biosynthesis pr... | *Leptospira interrogans serogroup Icterohaemorrhagiae serovar copenhageni* | EIQNLISSSGISEEISASYMR | Deam (NQ) | 3 | 2314.10 | 3.64 | 588250 | 45 |
| Q73T66 | Chaperone protein ClpB | *Mycolicibacterium paratuberculosis* | TVIALDLGSMVAGAKYR | Ox (M),diOx Y | 2 | 1811.95 | -3.43 | 1192200 | 80 |
| Q7A6A2 | Teichoic acid D-alanine hydrolase | *Staphylococcus aureus* | LKEVYNSKDPK | Ox Y | 3 | 1335.70 | 1.12 | 20744000 | 129 |
| Q7MWU9 | Holliday junction DNA helicase RuvB | *Porphyromonas gingivalis* | EVTELAYTHLGR | Glu->pyro-Glu,diOx Y | 2 | 1401.69 | 2.25 | 344950 | 54 |
| Q7N216 | Serine hydroxymethyltransferase | *Photorhabdus laumondii subsp. laumondii* | VLAICAKYPVYA | Ox Y,diOx Y | 2 | 1414.72 | -4.34 | 1125000 | 61 |
| Q7NBF8 | Cytadherence high molecular weight ... | *Mycoplasma gallisepticum* | ENDLNNQKR | Acetyl (K),2 Deam (NQ) | 2 | 1173.53 | 2.03 | 836370 | 53 |
| Q7NJR0 | Cobyric acid synthase | *Gloeobacter violaceus* | HWLNALRERR | Deam (NQ),Ox (W) | 2 | 1366.72 | -2.37 | 2737300 | 80 |
| Q7VI68 | L-seryl-tRNA(Sec) selenium transfer... | *Helicobacter hepaticus* | TLLEGDEER | Unmod | 2 | 1060.50 | -0.99 | 6674900 | 131 |
| Q7WQB2 | 7-cyano-7-deazaguanine synthase | *Bordetella bronchiseptica* | LGDDHLLDLGILAQVGDTAMTSDR | Deam (NQ) | 3 | 2526.23 | 3.34 | 440080 | 43 |
| Q824A9 | Proline--tRNA ligase | *Chlamydophila caviae* | QAYQNIFDR | Deam (NQ),O-2H (Y) | 2 | 1168.52 | 1.97 | 7992900 | 85 |
| Q82G68 | UPF0678 fatty acid-binding protein-... | *Streptomyces avermitilis* | TAAAGPYSGGKR | Ox Y | 2 | 1150.57 | -0.60 | 539300 | 77 |
| Q82TF6 | Protein RecA | *Nitrosomonas europaea* | GEEMVGNETR | di-Ox (M) | 2 | 1152.47 | -0.04 | 2932000 | 104 |
| Q82U08 | DNA mismatch repair protein MutS | *Nitrosomonas europaea* | EKMLYER | Glu->pyro-Glu,Ox Y | 2 | 965.46 | 4.44 | 639840 | 50 |
| Q839G9 | Elongation factor G | *Enterococcus faecalis* | VYSGVLESGSYVLNASKGK | Deam (NQ),2 diOx Y | 2 | 2021.98 | 1.09 | 1059000 | 59 |
| Q83C88 | tRNA N6-adenosine threonylcarbamoyl... | *Coxiella burnetii* | WSMAELNIIN | Ox (M),Trp->Kynurenine | 2 | 1209.57 | -3.19 | 993700 | 61 |
| Q83LC9 | UPF0259 membrane protein YciC | *Shigella flexneri* | MGIFASMR | di-Ox (M) | 1 | 943.43 | -0.52 | 863720 | 51 |
| Q83RK8 | D-guloside 3-dehydrogenase | *Shigella flexneri* | LRKMPQGSSWK | Ox (M),Deam (NQ),Ox (W) | 2 | 1349.68 | -0.74 | 589040 | 51 |
| Q86DC6 | Intermediate filament protein ifd-1 | *Caenorhabditis elegans* | EQLKHLSDLESETAYIK | Glu->pyro-Glu,Deam (NQ) | 2 | 1986.00 | -0.36 | 1091400 | 67 |
| Q88WM7 | Exodeoxyribonuclease 7 large subuni... | *Lactobacillus plantarum* | QRLNKLQTSYVFTQPNR | Acetyl (K),2 Deam (NQ) | 3 | 2136.10 | 0.53 | 1011200 | 54 |
| Q88XQ0 | DNA ligase | *Lactobacillus plantarum* | EAAGEPVFANPRNAAAGTLR | 2 Deam (NQ) | 3 | 2012.99 | 1.35 | 4186300 | 57 |
| Q891L4 | Homoserine kinase | *Clostridium tetani* | NDKNLFYVSMKK | Deam (NQ),di-Ox (M) | 2 | 1518.74 | -2.77 | 7173200 | 111 |
| Q893H9 | UPF0324 membrane protein CTC_01844 | *Clostridium tetani* | NENFKGNESTYHKDK | 2 Deam (NQ),O-2H (Y) | 2 | 1825.78 | 1.84 | 5923700 | 79 |
| Q89B09 | DNA primase | *Buchnera aphidicola subsp. Baizongia pistaciae* | NKKQCYNQK | Acetyl (K),3 Deam (NQ) | 2 | 1254.56 | -0.47 | 319200 | 44 |
| Q89SB5 | Adenine phosphoribosyltransferase | *Bradyrhizobium diazoefficiens* | AVDELVNPWAGNK | 2 Deam (NQ),Ox (W) | 2 | 1429.67 | 1.62 | 238320 | 56 |
| Q8A7Z7 | Histidine biosynthesis bifunctional... | *Bacteroides thetaiotaomicron* | NEEPVMFLKALQDFIDKR | Deam (NQ),di-Ox (M) | 3 | 2225.10 | -2.21 | 2908700 | 63 |
| Q8CNZ7 | GTPase Obg | *Staphylococcus epidermidis* | IINQELINYKQR | Deam (NQ),Iodination | 2 | 1657.73 | 1.99 | 1033700 | 50 |
| Q8CSD5 | Glycine--tRNA ligase | *Staphylococcus epidermidis* | PGEEIEWQNYWK | 2 Deam (NQ),Ox (W) | 2 | 1595.68 | -0.51 | 280250 | 46 |
| Q8CXQ1 | tRNA pseudouridine synthase A | *Mycoplasma penetrans* | DENLITVQSELEK | 2 Deam (NQ) | 2 | 1518.73 | 2.20 | 7173200 | 104 |
| Q8DIA3 | Elongation factor Ts | *Thermosynechococcus elongatus* | IGVLVEVNCETDFVARNEKFK | 2 Deam (NQ) | 3 | 2468.23 | 2.91 | 4027700 | 72 |
| Q8E4F4 | Chorismate synthase | *Streptococcus agalactiae serotype III* | FSSDNMYELK | Ox (M),O-2H (Y) | 2 | 1262.51 | 4.11 | 956500 | 72 |
| Q8EHN9 | Endoribonuclease YbeY | *Shewanella oneidensis* | TAIGNSMK | Deam (NQ) | 1 | 821.40 | -3.70 | 1708200 | 57 |
| Q8EPQ8 | S-adenosylmethionine:tRNA ribosyltr... | *Oceanobacillus iheyensis* | EAILHAYNEAVK | Glu->pyro-Glu,O-2H (Y) | 2 | 1352.67 | 0.69 | 833040 | 78 |
| Q8EWJ2 | Aspartyl/glutamyl-tRNA(Asn/Gln) ami... | *Mycoplasma penetrans* | DATDFQLNQLLKMMK | Ox (M),Deam (NQ) | 3 | 1811.88 | 3.19 | 1596100 | 53 |
| Q8EWR9 | 4-hydroxy-3-methylbut-2-enyl diphos... | *Mycoplasma penetrans* | QIESHLIWNTK | Gln->pyro-Glu,Di-Ox W | 2 | 1382.68 | -1.82 | 8018200 | 81 |
| Q8G3I2 | Isoleucine--tRNA ligase | *Bifidobacterium longum* | ASVLKYTNEWQNYVHR | Deam (NQ),Ox Y,diOx Y | 3 | 2055.97 | 1.39 | 30852000 | 67 |
| Q8GB18 | Crotonobetaine/carnitine--CoA ligas... | *Proteus sp.* | TSMAQPVSPDEKQHK | Acetyl (K),Ox (M),Deam (NQ) | 2 | 1740.80 | -1.17 | 934520 | 48 |
| Q8K9L7 | Septum site-determining protein Min... | *Buchnera aphidicola subsp. Schizaphis graminum* | NITPIKEYLLLTR | Deam (NQ),Iodination | 2 | 1699.81 | -2.28 | 1019400 | 83 |
| Q8K9S7 | 1-deoxy-D-xylulose 5-phosphate redu... | *Buchnera aphidicola subsp. Schizaphis graminum* | VAISYAMSWPNR | Ox (M),Deam (NQ),Trp->Kynurenine | 2 | 1414.66 | -2.04 | 4511500 | 68 |
| Q8RDI4 | Recombination protein RecR | *Caldanaerobacter subterraneus subsp. tengcongensis* | SLSQAIIEAKEK | Acetyl (K),Deam (NQ) | 2 | 1358.73 | 0.20 | 2166900 | 95 |
| Q8REG1 | Glucosamine-6-phosphate deaminase | *Fusobacterium nucleatum subsp. nucleatum* | ENVNILNGMAK | Ox (M),Glu->pyro-Glu,Deam (NQ) | 2 | 1200.58 | -2.87 | 805720 | 54 |
| Q8RI94 | DNA ligase | *Fusobacterium nucleatum subsp. nucleatum* | EDLNKYR | Glu->pyro-Glu,diOx Y | 2 | 950.45 | 1.38 | 531660 | 48 |
| Q8Y206 | tRNA-2-methylthio-N(6)-dimethylally... | *Ralstonia solanacearum* | LIEAYATNR | Deam (NQ),diOx Y | 2 | 1082.52 | -0.03 | 1189400 | 74 |
| Q8YWW1 | Probable phosphoketolase 1 | *Nostoc sp.* | MKKEIINNR | Deam (NQ),di-Ox (M) | 2 | 1177.61 | -3.39 | 1238600 | 98 |
| Q8Z5G6 | Tyrosine-protein kinase wzc | *Salmonella typhi* | NYLQQDIAWKSEEAGK | Deam (NQ),Di-Ox W | 3 | 1911.89 | -1.01 | 2155700 | 73 |
| Q8ZC64 | Trigger factor | *Yersinia pestis* | QAAQRFGGNEKQAAELPR | Acetyl (K),Deam (NQ) | 2 | 2013.00 | -0.88 | 5610500 | 97 |
| Q8ZL66 | Uncharacterized protein YibT | *Salmonella typhimurium* | LYRPKEEER | Ox Y | 2 | 1234.63 | -3.68 | 4689600 | 117 |
| Q92GM6 | Queuine tRNA-ribosyltransferase | *Rickettsia conorii* | NYSKAYLHHLVR | Deam (NQ),diOx Y,O-2H (Y) | 2 | 1546.75 | 0.70 | 1660200 | 67 |
| Q92JC1 | Uncharacterized lipoprotein RC0146 | *Rickettsia conorii* | SLFENWLR | Deam (NQ),Di-Ox W | 2 | 1096.52 | 1.03 | 1197800 | 63 |
| Q934F5 | Formate dehydrogenase subunit alpha | *Desulfovibrio gigas* | GPDMDPKK | Acetyl (K) | 1 | 928.43 | -0.88 | 368770 | 52 |
| Q93D97 | Putative ABC transporter ATP-bindin... | *Streptococcus mutans serotype c* | VLKVLKTCGLYEFR | Acetyl (K),TriOx (C) | 2 | 1814.96 | -2.43 | 1536700 | 94 |
| Q93RV9 | Ectoine dioxygenase | *Streptomyces coelicolor* | TISVSIALTENYDTNGGLMIMPGSHK | Ox (M),2 Deam (NQ) | 3 | 2766.31 | 3.87 | 295860 | 43 |
| Q97H75 | Probable manganese-dependent inorga... | *Clostridium acetobutylicum* | IGVSQVTTMDIEGFDEYKK | Acetyl (K),Ox (M) | 2 | 2217.05 | 2.88 | 618280 | 43 |
| Q97KG0 | Chaperone protein ClpB | *Clostridium acetobutylicum* | KIIAEEGYDPVYGAR | 2 diOx Y | 2 | 1743.83 | 3.12 | 889710 | 70 |
| Q98MZ6 | 2-dehydro-3-deoxyphosphooctonate al... | *Mesorhizobium japonicum* | TSYDKANR | diOx Y | 1 | 985.45 | 2.12 | 1741400 | 59 |
| Q98Q16 | Transcription elongation factor Gre... | *Mycoplasma pulmonis* | ITELENILSNAISIQESHSDK | Deam (NQ) | 4 | 2341.17 | -0.83 | 455040 | 43 |
| Q98QV2 | Translation initiation factor IF-3 | *Mycoplasma pulmonis* | ELGIDTLNR | Unmod | 2 | 1029.55 | -0.95 | 1591900 | 130 |
| Q99TD5 | Tyrosine--tRNA ligase | *Staphylococcus aureus* | TNVLIEDLKWR | Deam (NQ),Ox (W) | 2 | 1402.75 | 0.19 | 849720 | 103 |
| Q99VR8 | Histidine protein kinase SaeS | *Staphylococcus aureus* | MASEITQQMNQIK | Ox (M),2 Deam (NQ) | 2 | 1538.70 | 3.69 | 374960 | 62 |
| Q9AKD5 | Transcription-repair-coupling facto... | *Rickettsia typhi* | YFLKLYPKIK | Iodination,Ox Y | 3 | 1453.69 | -0.63 | 832190 | 68 |
| Q9CNA6 | Copper homeostasis protein CutC | *Pasteurella multocida* | QLVSQASNRIR | 2 Deam (NQ) | 2 | 1272.68 | -4.05 | 2682100 | 131 |
| Q9FAX1 | DNA gyrase subunit B | *Eisenibacter elegans* | ELIEQGYLYVALPPLYIVK | 2 Ox Y | 2 | 2252.23 | 3.02 | 523560 | 65 |
| Q9HYT3 | Uncharacterized signaling protein P... | *Pseudomonas aeruginosa* | MPKPAVCR | TriOx (C) | 2 | 1005.47 | 3.58 | 5634500 | 55 |
| Q9I676 | D-hydantoinase/dihydropyrimidinase | *Pseudomonas aeruginosa* | QSLLEAFHTWR | Gln->pyro-Glu,Di-Ox W | 2 | 1401.67 | -0.21 | 5654900 | 62 |
| Q9K7E4 | Homoserine kinase | *Bacillus halodurans* | GEWVQKQLQK | Deam (NQ),O-2H (W) | 2 | 1257.64 | 1.63 | 573440 | 65 |
| Q9KDJ5 | L-aspartate oxidase | *Bacillus halodurans* | EATGDGIALAYR | Glu->pyro-Glu,diOx Y | 2 | 1249.59 | 1.29 | 566310 | 65 |
| Q9KGK8 | Methionine--tRNA ligase | *Bacillus halodurans* | KVICVTNLKPVK | Deam (NQ) | 2 | 1398.83 | -2.71 | 399360 | 78 |
| Q9KU47 | Isoleucine--tRNA ligase | *Vibrio cholerae serotype O1* | GDLAKREPEMLQR | Deam (NQ),di-Ox (M) | 2 | 1574.77 | 2.23 | 1374100 | 69 |
| Q9KZX7 | Virginiamycin B lyase | *Streptomyces coelicolor* | VTVHPVGDGPTVIAPGPDGALWFTEYR | Ox (W) | 3 | 2866.43 | -3.41 | 9373900 | 42 |
| Q9LAI1 | Type-2 restriction enzyme BslI subu... | *Bacillus sp.* | ENYYKTYR | Glu->pyro-Glu,Ox Y | 2 | 1133.51 | -3.57 | 465900 | 44 |
| Q9N4G7 | Phosphatidate cytidylyltransferase,... | *Caenorhabditis elegans* | LNLLPSEVLNRIQKNMNR | Ox (M),3 Deam (NQ) | 3 | 2170.14 | 2.53 | 1256800 | 45 |
| Q9PE76 | 50S ribosomal protein L3 | *Xylella fastidiosa* | KMSGHMGAVRQSVQNLEVVK | Deam (NQ),2 di-Ox (M) | 2 | 2262.11 | 1.08 | 2778600 | 67 |
| Q9PIZ3 | Homoserine kinase | *Campylobacter jejuni subsp. jejuni serotype O:2* | FASQDKLHEINRMK | 2 Deam (NQ),di-Ox (M) | 2 | 1749.84 | 0.50 | 1825300 | 72 |
| Q9PK41 | Peptide deformylase | *Chlamydia muridarum* | IRDLEYYDSPILRK | 2 O-2H (Y) | 2 | 1807.91 | 2.86 | 3603700 | 98 |
| Q9PL40 | Uncharacterized protein TC_0268 | *Chlamydia muridarum* | NSARSYYDVPRVPPQNEVEEMHVTK | Ox Y | 3 | 2960.41 | -4.44 | 1815300 | 56 |
| Q9RGX2 | 30S ribosomal protein S21 | *Haemophilus ducreyi* | ENARNTRLY | Deam (NQ),diOx Y | 2 | 1168.55 | 2.33 | 537960 | 85 |
| Q9RMW6 | Uncharacterized protein pXO2-68/BXB... | *Bacillus anthracis* | EGYEVRKNSR | Deam (NQ),Iodination | 2 | 1363.50 | -3.84 | 1793100 | 53 |
| Q9WX76 | Ribosome-recycling factor | *Thermus thermophilus* | TLKELYAETRSHMQK | Deam (NQ),diOx Y | 3 | 1866.92 | -0.60 | 20248000 | 78 |
| Q9WY30 | Cyclic di-GMP phosphodiesterase TM_... | *Thermotoga maritima* | NRVFHMYRNSYLK | Ox (M),Deam (NQ),2 Di-iodination | 4 | 2247.44 | 0.07 | 71168000 | 44 |
| Q9X295 | Probable 2,3-bisphosphoglycerate-in... | *Thermotoga maritima* | TPLQAANTPNLDNLAK | 3 Deam (NQ) | 3 | 1682.84 | 3.24 | 742740 | 59 |
| Q9XTG7 | Serine/threonine-protein kinase akt... | *Caenorhabditis elegans* | EDQPLPEPLNNFMIR | Ox (M),Deam (NQ) | 3 | 1828.87 | -3.02 | 1814200 | 55 |
| Q9Z7C9 | 60 kDa chaperonin 2 | *Chlamydia pneumoniae* | ETFTFLEGGGDAEIIQARK | Deam (NQ) | 3 | 2082.03 | -2.09 | 935160 | 42 |
| Q9ZCT8 | Glutamate--tRNA ligase 2 | *Rickettsia prowazekii* | LKIIYYNNLNF | Deam (NQ),diOx Y,O-2H (Y) | 2 | 1460.72 | 3.92 | 1473100 | 85 |
| Q9ZD71 | Uncharacterized protein RP473 | *Rickettsia prowazekii* | YTHYLLNVNLLVYNFIQDKDYSKELR | Iodination,Ox Y | 3 | 3402.58 | -4.39 | 545550 | 43 |
| Q9ZGG6 | Glutamyl-tRNA reductase | *Heliobacillus mobilis* | WLNSLFVIPTIVGLK | Deam (NQ),O-2H (W) | 2 | 1713.97 | 0.22 | 2037700 | 86 |
| Q9ZJU8 | Tryptophan biosynthesis protein Trp... | *Helicobacter pylori* | AVQLYGYSQKEIAQLK | Deam (NQ),Ox Y | 3 | 1854.97 | -2.46 | 2310800 | 67 |
| V9TSX0 | Chaperone protein LppX | *Paenibacillus barcinonensis* | ENLKPYYTIDKETTDPDNK | 2 Acetyl (K),Deam (NQ) | 3 | 2368.10 | 4.47 | 2581000 | 44 |

Table S6 List of peptides and proteins of Bacteria/Nematoda in trunk tip sample: for each peptide all the features (same sequence and different modifications) with the highest intensity are reported.

| **Leading razor protein** | **Description** | **Specie** | **Peptide Sequence** | **Modifications** | **Charge** | **Mass** | **Mass error [ppm]** | **Intensity** | **Score** |
| --- | --- | --- | --- | --- | --- | --- | --- | --- | --- |
| Q6F1R8 | Cytidylate kinase | Mesoplasma florum | IKELEGN | Deam (NQ) | 1 | 802.41 | 0.17 | 134680 | 50 |
|  |  |  | QNERLGIKNNNLNEIK | 2 Deam (NQ) | 3 | 1897.99 | 2.46 | 5094300 | 81 |
| P00370 | NADP-specific glutamate dehydrogenase | Escherichia coli | NQIQVNRAWR | Deam (NQ),Trp->Kynurenine | 2 | 1288.66 | -0.72 | 357370 | 67 |
|  |  |  | NQIQVNRAWR | Deam (NQ),Di-Ox W | 2 | 1316.66 | 0.21 | 336170 | 65 |
| Q0A7E2 | Protein GrpE | Alkalilimnicola ehrlichii | AEMQNIQRR | Deam (NQ) | 2 | 1145.56 | 0.96 | 921120 | 104 |
|  |  |  | AEMQNIQRR | 2 Deam (NQ) | 2 | 1146.55 | -0.67 | 369980 | 122 |
| A0K1I7 | tRNA (guanine-N(7)-)-methyltransferasemethyltransferase) | Arthrobacter sp. | QQAWEEHSDR | 2 Deam (NQ),Di-Ox W | 2 | 1318.51 | 0.08 | 3567000 | 78 |
| A0LUG7 | Elongation factor P, EF-P | Acidothermus cellulolyticus | RTMQYLYR | Ox (M),Deam (NQ) | 2 | 1146.55 | -4.47 | 2039900 | 67 |
| A0M764 | Fructose-1,6-bisphosphatase class 1 2 | Gramella forsetii | LRLLYECNPFAFITEQAGGKASDGFQR | Deam (NQ),diOx Y | 3 | 3120.50 | 3.72 | 262600 | 40 |
| A0QNG1 | Serine/threonine-protein kinase PknB | Mycolicibacterium smegmatis | AGAATQDMPVPR | Ox (M),Deam (NQ) | 2 | 1229.57 | 3.83 | 158420 | 67 |
| A0RP01 | Macrolide export ATP-binding/permease protein | Campylobacter fetus subsp. fetus | SNIIIDQFTKDAFFK | Deam (NQ) | 2 | 1786.91 | 2.39 | 3929700 | 134 |
| A1AWP4 | UDP-N-acetylglucosamine 1-carboxyvinyltransferase | Ruthia magnifica subsp. Calyptogena magnifica | VENGYIYATAKK | Deam (NQ),diOx Y | 2 | 1388.68 | -1.54 | 810440 | 109 |
| A1JRZ2 | Methionyl-tRNA formyltransferase | Yersinia enterocolitica serotype O:8 / biotype 1B | EWFTLGNQLA | 2 Deam (NQ),Di-Ox W | 2 | 1211.53 | -2.41 | 984480 | 62 |
| A1S7S0 | Putative pterin-4-alpha-carbinolamine dehydratase, | Shewanella amazonensis | NFKLAMAFSNK | Ox (M),2 Deam (NQ) | 2 | 1287.62 | -1.59 | 76117 | 66 |
| A1S8L1 | Gamma-glutamyl phosphate reductase | Shewanella amazonensis | LLDNGMR | Ox (M),Deam (NQ) | 1 | 834.39 | -4.20 | 569680 | 65 |
| A1T087 | 4-hydroxybenzoate octaprenyltransferase | Psychromonas ingrahamii | QQLQIKNR | 3 Deam (NQ) | 2 | 1029.55 | 1.48 | 1447100 | 116 |
| A1TZ46 | CTP synthase | Marinobacter hydrocarbonoclasticus | IRTVQYARENK | Deam (NQ),diOx Y | 3 | 1409.73 | -2.27 | 253910 | 74 |
| A1URS7 | Protein translocase subunit SecA | Bartonella bacilliformis | IEKIKQDVQK | Acetyl (K),Deam (NQ) | 2 | 1270.71 | 3.36 | 1761900 | 98 |
| A2CE29 | Argininosuccinate synthase | Prochlorococcus marinus | TQIYVNGLVR | Deam (NQ),O-2H (Y) | 2 | 1176.61 | -1.22 | 395350 | 67 |
| A4IQA1 | Glycerol-3-phosphate dehydrogenase [NAD(P)+] | Geobacillus thermodenitrificans | QEMNDLMNIFAEQ | 2 Ox (M),Deam (NQ) | 2 | 1614.65 | -2.64 | 10154000 | 59 |
| A4IRD5 | Probable septum site-determining protein MinC | Geobacillus thermodenitrificans | AEAIEWVQK | Deam (NQ),Ox (W) | 2 | 1089.53 | -3.98 | 313390 | 81 |
| A4SNE3 | tRNA 5-methylaminomethyl-2-thiouridine biosynthesis bifunctional protein MnmC, | Aeromonas salmonicida | NPEMWTQDLFDGLAR | 2 Deam (NQ),O-2H (W) | 2 | 1807.77 | 3.07 | 283550 | 75 |
| A4VIG6 |  | Pseudomonas stutzeri | DLNWIRNAFES | Deam (NQ),Trp->Kynurenine | 2 | 1368.63 | -2.15 | 647140 | 54 |
| A4WB84 | Cardiolipin synthase A | Enterobacter sp. | MIMIDNYIAYTGSMNMVDPR | 2 Ox (M),2 Deam (NQ) | 2 | 2368.01 | -3.70 | 836110 | 58 |
| A4X6K7 | Catalase-peroxidase | Salinispora tropica | MSDTQDNAPVSAQGVDQK | 2 Deam (NQ),di-Ox (M) | 2 | 1923.80 | -1.76 | 229760 | 44 |
| A4XIQ3 | UvrABC system protein C | Caldicellulosiruptor saccharolyticus | NGKLINK | Deam (NQ) | 1 | 786.46 | -0.59 | 5615700 | 50 |
| A5CX33 | Dual-specificity RNA methyltransferase RlmN | Vesicomyosocius okutanii subsp. Calyptogena okutanii | NLTTAEIIAQVLIANK | 3 Deam (NQ) | 2 | 1713.94 | 0.14 | 624220 | 115 |
| A5EVQ4 | Carboxy-S-adenosyl-L-methionine synthase | Dichelobacter nodosus | ASQGYSALAIAQK | Deam (NQ),O-2H (Y) | 2 | 1321.65 | 0.74 | 3107700 | 115 |
| A5GSL0 | Adenine phosphoribosyltransferase, APRT | Synechococcus sp. | LEIQEGALSGCQR | 2 Deam (NQ),TriOx (C) | 2 | 1509.66 | -1.98 | 948440 | 92 |
| A6M1P1 | S-adenosylmethionine decarboxylase proenzyme, | Clostridium beijerinckii | LEIENNLR | Deam (NQ) | 2 | 1000.52 | 1.42 | 3434700 | 148 |
| A6Q7E9 | Putative pre-16S rRNA nuclease, | Sulfurovum sp. | IVMPQNAILR | Ox (M),2 Deam (NQ) | 2 | 1171.63 | 1.76 | 293460 | 87 |
| A6QBN8 | ATP-dependent zinc metalloprotease FtsH | Sulfurovum sp. | MANPNNNNDNK | Ox (M),3 Deam (NQ) | 2 | 1263.47 | -3.17 | 571920 | 44 |
| A7LXS8 | Beta-glucosidase BoGH3A | Bacteroides ovatus | VQLLPNEEK | Deam (NQ) | 2 | 1069.57 | 0.54 | 12538000 | 108 |
| A7NBJ3 | Ferrochelatase | Francisella tularensis subsp. holarctica | GINPYYDNK | Deam (NQ),Ox Y,diOx Y | 2 | 1131.47 | 1.55 | 263210 | 54 |
| A7ZAW7 | Chromosomal replication initiator protein DnaA | Campylobacter concisus | NIKQNQVNVK | Acetyl (K),3 Deam (NQ) | 2 | 1228.63 | 1.24 | 152980 | 51 |
| A8EXD2 | Arginine--tRNA ligase | Rickettsia canadensis | AESWQIAIK | Deam (NQ),Trp->Kynurenine | 2 | 1049.54 | 0.99 | 1573500 | 115 |
| A8EXY1 | 50S ribosomal protein L13 | Rickettsia canadensis | PSFTPHLDCGDNIIIINAAHVK | 2 Deam (NQ) | 3 | 2433.20 | 0.52 | 470130 | 44 |
| A8XP14 | Golgi SNAP receptor complex member 2 homolog memb-1 | Caenorhabditis briggsae | MRVDQLRMDVQR | Ox (M),2 Deam (NQ) | 2 | 1563.75 | 4.09 | 503850 | 74 |
| A8XW88 | cAMP-dependent protein kinase catalytic subunit | Caenorhabditis briggsae | WFGSTDWIAIYQR | Deam (NQ),Ox (W) | 2 | 1658.77 | 0.13 | 746330 | 74 |
| A9ALQ1 | Enoyl-[acyl-carrier-protein] reductase [NADH] | Burkholderia multivorans | ELDPQIQK | Deam (NQ) | 2 | 970.50 | 1.24 | 4939000 | 144 |
| A9BAE3 | Glucose-6-phosphate isomerase | Prochlorococcus marinus | MNVNEHELKELK | 2 Deam (NQ),di-Ox (M) | 2 | 1516.71 | 1.71 | 1214900 | 62 |
| B0S1G9 | Triosephosphate isomerase, TIM, TPI | Finegoldia magna | EDDELLNKKIASALK | Glu->pyro-Glu,Deam (NQ) | 2 | 1668.89 | 3.56 | 938920 | 71 |
| B0TZD6 | Lipoyl synthase | Francisella philomiragia subsp. philomiragia | KQDSKEYLK | Deam (NQ),O-2H (Y) | 2 | 1152.57 | 0.35 | 1887500 | 122 |
| B1HMW1 | 50S ribosomal protein L15 | Lysinibacillus sphaericus | VLGNGTLNKK | Acetyl (K),Deam (NQ) | 2 | 1085.61 | -2.96 | 217110 | 79 |
| B1MY62 | Proline--tRNA ligase | Leuconostoc citreum | LPLVVYQIQPK | 2 Deam (NQ),O-2H (Y) | 2 | 1312.73 | -1.58 | 215320 | 53 |
| B1Y2Y8 | Transaldolase | Leptothrix cholodnii | SAGANWDEAAMAGANDPGVK | Deam (NQ) | 3 | 1931.83 | 2.89 | 1060400 | 58 |
| B1YGA8 | tRNA modification GTPase MnmE- | Exiguobacterium sibiricum | QASQMIEDALGAAEASMPIDMVQIDLR | Ox (M),Deam (NQ) | 3 | 2919.37 | 1.61 | 1485100 | 41 |
| B2JFK0 | Elongation factor 4 | Paraburkholderia phymatum | GVQINMQYHGR | Ox (M),2 Deam (NQ) | 2 | 1319.59 | 2.11 | 326140 | 60 |
| B2RZW1 | Leucine--tRNA ligase | Borrelia hermsii | IMQNIQNKQIIK | Ox (M),3 Deam (NQ) | 2 | 1488.79 | 4.04 | 176720 | 80 |
| B2V7Y8 | UDP-N-acetylglucosamine--N-acetylmuramyl-(pentapeptide) pyrophosphoryl-undecaprenol N-acetylglucosamine transferase | Sulfurihydrogenibium sp. | AKNYVYKPNQTK | Acetyl (K),2 Deam (NQ) | 2 | 1496.75 | 4.42 | 4233300 | 106 |
| B3GZC5 | Ribosomal RNA small subunit methyltransferase J | Actinobacillus pleuropneumoniae serotype 7 | MTIQLINESSNTEK | Ox (M),2 Deam (NQ) | 2 | 1624.75 | -2.83 | 449230 | 56 |
| B3GZN4 | Isoleucine--tRNA ligase, | Actinobacillus pleuropneumoniae serotype 7 | EQALGEIKSVR | Glu->pyro-Glu,Deam (NQ) | 2 | 1211.65 | -0.31 | 1198800 | 76 |
| B3QY46 | NADH-quinone oxidoreductase subunit C | Chloroherpeton thalassium | VQEVYHGIK | Deam (NQ),O-2H (Y) | 2 | 1086.53 | -3.18 | 205680 | 74 |
| B3XPQ7 | Glucosyltransferase 3 | Lactobacillus reuteri | YLLPQFIDYYNK | Deam (NQ),Ox Y,diOx Y | 2 | 1624.77 | 1.45 | 734490 | 70 |
| B4F0D4 | Protein ViaA (VWA domain protein interacting with AAA ATPase) | Proteus mirabilis | LQGDSWQER | 2 Deam (NQ),O-2H (W) | 2 | 1133.46 | 3.39 | 161270 | 57 |
| B4SK03 | S-adenosylmethionine synthase, AdoMet synthase | Stenotrophomonas maltophilia | NSPLSWLRPDAK | Deam (NQ),O-2H (W) | 2 | 1397.69 | -2.26 | 167390 | 69 |
| B5XT16 | Probable intracellular septation protein A | Klebsiella pneumoniae | ELALPQQVWSR | 2 Deam (NQ),Di-Ox W | 2 | 1359.67 | -2.20 | 3768200 | 99 |
| B5Y1G4 | Hydroxyacylglutathione hydrolase | Klebsiella pneumoniae | INLFLRVNDIDLIDK | 2 Deam (NQ) | 3 | 1801.98 | 3.81 | 970320 | 101 |
| B5YIL6 | Methionyl-tRNA formyltransferase | Thermodesulfovibrio yellowstonii | GKNLQAPEIKK | 2 Acetyl (K),2 Deam (NQ) | 2 | 1310.71 | 0.76 | 786860 | 76 |
| B5YJT0 | Ketol-acid reductoisomerase (NADP(+)), KARI | Thermodesulfovibrio yellowstonii | VINSSVKAEMKK | Acetyl (K),Deam (NQ),di-Ox (M) | 2 | 1407.73 | -1.27 | 426690 | 76 |
| B6A878 | Toxin subunit YenA2 | Yersinia entomophaga | QEWELQYK | 2 Deam (NQ),O-2H (W) | 2 | 1138.48 | -3.92 | 223640 | 59 |
| B7GJ95 | 50S ribosomal protein L17 | Anoxybacillus flavithermus | KEVANTETGQDAIQK | 2 Deam (NQ) | 2 | 1632.78 | -0.73 | 383730 | 82 |
| B7I7B5 | Peptidyl-tRNA hydrolase, PTH, | Acinetobacter baumannii | LLLPMTYMNRSGQSVVPFSK | Ox (M),Deam (NQ) | 3 | 2284.16 | 0.00 | 223090 | 47 |
| B7LL09 | Maltoporin (Maltose-inducible porin) | Escherichia fergusonii | WGYDYNDNSK | 2 Deam (NQ),Ox (W) | 2 | 1278.47 | -1.63 | 894380 | 44 |
| B9DVB7 | Translation initiation factor IF-2 | Streptococcus uberis | GSQQNNR | 2 Deam (NQ) | 1 | 804.34 | 4.12 | 133040 | 51 |
| B9JML7 | Acetaldehyde dehydrogenase | Agrobacterium radiobacter | TLIEENA | Deam (NQ) | 1 | 789.38 | -0.58 | 321880 | 53 |
| C0QH22 | Phosphoribosylaminoimidazole-succinocarboxamide synthase | Desulfobacterium autotrophicum | DLSIQIYLKGAQTALKK | Deam (NQ),Ox Y | 2 | 1906.08 | -1.35 | 3261400 | 69 |
| C0R5G4 | Ribosomal RNA small subunit methyltransferase A | Wolbachia sp. subsp. Drosophila simulans | SLGQNFILSSEITKK | 2 Deam (NQ) | 2 | 1665.88 | -2.83 | 1804200 | 74 |
| C1DU51 | UPF0753 protein SULAZ_0653 | Sulfurihydrogenibium azorense | RNLERVDNYK | Deam (NQ),Ox Y | 2 | 1322.66 | -0.44 | 448760 | 76 |
| C1F030 | 5-oxoprolinase subunit A, 5-OPase subunit A | Bacillus cereus | LNNISICAPK | 2 Deam (NQ) | 2 | 1130.56 | -3.33 | 507950 | 71 |
| C3MBY8 | 2,3-bisphosphoglycerate-dependent phosphoglycerate mutase, BPG-dependent PGAM, PGAM, Phosphoglyceromutase, dPGM, EC 5.4.2.11 | Sinorhizobium fredii | LTKEQVLNLNLATGVPMVYK | Deam (NQ),O-2H (Y) | 3 | 2245.20 | -1.99 | 2347400 | 56 |
| C3MF10 | Endoribonuclease YbeY, EC 3.1.-.- | Sinorhizobium fredii | EINAEWRNQDK | Deam (NQ),Trp->Kynurenine | 2 | 1406.64 | 0.04 | 440120 | 52 |
| C4L7S5 | N-acetyl-gamma-glutamyl-phosphate reductase, AGPR | Tolumonas auensis | AVYGLAEWNAEQIK | Deam (NQ),Ox (W) | 2 | 1607.78 | -1.44 | 1140500 | 63 |
| C4LA32 | Protein translocase subunit SecA, EC 7.4.2.8 | Tolumonas auensis | ARQAYLTENGQIFVEGWLK | 2 Deam (NQ) | 3 | 2224.12 | -1.49 | 7348300 | 65 |
| C6DKU3 | Dihydroorotase, DHOase, EC 3.5.2.3 | Pectobacterium carotovorum subsp. carotovorum | RNTHQQALREAVASGCER | Deam (NQ) | 2 | 2083.00 | -3.31 | 501070 | 42 |
| G5ECQ3 | Serine/threonine-protein kinase sel-5 | Caenorhabditis elegans | KEPKQLSENK | Deam (NQ) | 2 | 1200.64 | 0.03 | 291520 | 101 |
| G5EGM3 | Transcription initiation factor TFIID subunit 1 (TBP-associated transcription factor 1) | Caenorhabditis elegans | ISDPMDLSIMEQK | 2 Ox (M),Deam (NQ) | 2 | 1538.68 | 0.06 | 1422800 | 67 |
| O01803 | Ras-related protein rab-11.1 (Rab GTPase rab-11.1) | Caenorhabditis elegans | AQIWDTAGQERYR | Deam (NQ),diOx Y | 2 | 1625.74 | -1.53 | 166700 | 62 |
| O07567 | NTD biosynthesis operon regulator NtdR | Bacillus subtilis | DMILKAINELDYTPNYLAR | Deam (NQ),diOx Y | 3 | 2285.12 | 0.93 | 257380 | 47 |
| O17514 | Histone-lysine N-methyltransferase mes-2 | Caenorhabditis elegans | ENGVCSYMCK | Ox (M),Deam (NQ),diOx Y | 2 | 1295.45 | 3.99 | 170280 | 64 |
| O31662 | Methylthioribose-1-phosphate isomerase, M1Pi, MTR-1-P isomerase, EC 5.3.1.23 (S-methyl-5-thioribose-1-phosphate isomerase) | Bacillus subtilis | LIGQNALQLFK | 2 Deam (NQ) | 2 | 1245.70 | 0.12 | 324160 | 89 |
| O44568 | Probable peptide chain release factor 1, mitochondrial, MRF-1, MtRF-1 | Caenorhabditis elegans | SELSQLR | Deam (NQ) | 2 | 832.43 | -0.25 | 2802400 | 205 |
| O45717 | Protein nud-2 | Caenorhabditis elegans | IDDNKNIQEK | Acetyl (K),2 Deam (NQ) | 2 | 1259.59 | -0.65 | 6146200 | 107 |
| O51125 | Endonuclease MutS2, EC 3.1.-.- | Borrelia burgdorferi | TNKVNSLNNKR | 3 Deam (NQ) | 2 | 1289.66 | 0.51 | 294510 | 68 |
| O84879 | Probable outer membrane protein PmpG (Polymorphic membrane protein G) | Chlamydia trachomatis | LAWDPNTANNGPYTLK | 2 Deam (NQ),O-2H (W) | 2 | 1789.82 | -2.73 | 224740 | 48 |
| O86029 | Uncharacterized HTH-type transcriptional regulator RB1450 | Rhizobium meliloti | LSPGAIVMETQSVNADLEGR | 2 Deam (NQ),di-Ox (M) | 2 | 2119.99 | 2.06 | 303720 | 42 |
| P00863 | Histidine decarboxylase small chain, HDC, EC 4.1.1.22 | Micrococcus sp. | KYSKGFMEDGDIGVQY | Deam (NQ),O-2H (Y) | 2 | 1850.80 | -4.17 | 393850 | 60 |
| P07883 | Extracellular agarase, EC 3.2.1.81 | Streptomyces coelicolor | GRWLDQHK | Deam (NQ),Ox (W) | 2 | 1055.51 | -0.31 | 4406000 | 83 |
| P0CF93 | Putative transposase InsL for insertion sequence element IS186C | Escherichia coli | NGETTVMIGNSGNKK | Ox (M),Deam (NQ) | 2 | 1565.74 | -2.45 | 2499600 | 108 |
| P0CW32 | Putative antitoxin VapB23 | Mycobacterium tuberculosis | QAGLRQLEAQRQR | 2 Deam (NQ) | 2 | 1554.82 | 1.93 | 522990 | 70 |
| P11797 | Chitinase B, EC 3.2.1.14 | Serratia marcescens | QLEQMLQGNYGYQR | Deam (NQ),Ox Y | 2 | 1743.79 | -3.34 | 710330 | 47 |
| P15362 | ABC transport system permease protein p69 | Mycoplasma hyorhinis | LNNVTQWIPLR | 2 Deam (NQ),Di-Ox W | 2 | 1386.71 | -2.45 | 1999600 | 97 |
| P23731 | Intermediate filament protein B, IF-B | Ascaris suum | SAKGNVTISECDPNGKFITLENTHR | 2 Acetyl (K),Deam (NQ) | 3 | 2872.37 | 1.29 | 624130 | 48 |
| P24203 | P-loop guanosine triphosphatase YjiA, EC 3.6.-.- (GTP-binding protein YjiA) | Escherichia coli | ATQIKTLTNGCICCSR | 2 Deam (NQ),TriOx (C) | 3 | 1931.84 | 0.00 | 1060400 | 121 |
| P26024 | N-acetylglucosaminyltransferase | Bradyrhizobium diazoefficiens | MHDPGIGAAMGQLIASNR | Ox (M),2 Deam (NQ) | 2 | 1855.86 | 0.12 | 1236700 | 47 |
| P28808 | Thermoregulatory protein LcrF | Yersinia pestis | MSIVDIAMEAGFSSQSYFTQSYRRR | Deam (NQ),O-2H (Y) | 3 | 2944.35 | 1.20 | 471960 | 41 |
| P30195 | Epidermin biosynthesis protein EpiB | Staphylococcus epidermidis | SDNIDYDLILQNDIFK | Deam (NQ),Ox Y | 2 | 1941.92 | -3.34 | 892290 | 54 |
| P34644 | Probable vesicular glutamate transporter eat-4 (Abnormal pharyngeal pumping eat-4) | Caenorhabditis elegans | VLQVMEQTWIGK | 2 Deam (NQ) | 2 | 1432.73 | -4.38 | 289920 | 78 |
| P34892 | Receptor-like tyrosine-protein kinase kin-16 | Caenorhabditis elegans | INLPNQQDVIYR | Deam (NQ),O-2H (Y) | 2 | 1486.74 | -1.87 | 21553000 | 89 |
| P37029 | Uncharacterized protein blr1755 | Bradyrhizobium diazoefficiens | VMIEAGGCNGFK | Ox (M),Deam (NQ),TriOx (C) | 2 | 1346.55 | -1.62 | 147250 | 48 |
| P39434 | Soluble lytic murein transglycosylase, EC 4.2.2.n1 (Peptidoglycan lytic exotransglycosylase) (Slt70) | Salmonella typhimurium | YAQIKQAWDNR | Deam (NQ),Ox (W) | 2 | 1408.67 | -2.52 | 852500 | 82 |
| P39450 | S-(hydroxymethyl)glutathione dehydrogenase | Photobacterium damsela subsp. piscicida | ETQGKGVMPDATSR | Ox (M),Deam (NQ) | 2 | 1492.68 | 2.81 | 720750 | 79 |
| P40294 | Yop proteins translocation protein O | Yersinia pseudotuberculosis serotype I | NTTLNCKDLEKWQR | Deam (NQ),O-2H (W) | 2 | 1819.85 | -1.57 | 1434900 | 81 |
| P43752 | Anaerobic ribonucleoside-triphosphate reductase | Haemophilus influenzae | GYYTNSYHLDVEKKVNPYDK | Deam (NQ),Iodination,Di-iodination,O-2H (Y) | 4 | 2824.82 | 3.92 | 2715900 | 40 |
| P46119 | Uncharacterized protein YbjC | Escherichia coli | RLAPQLMNR | Ox (M),Deam (NQ) | 2 | 1114.59 | -2.17 | 780580 | 51 |
| P47282 | Aspartate--tRNA ligase | Mycoplasma genitalium | EQIQQLTR | Deam (NQ) | 2 | 1015.53 | 0.71 | 2551400 | 136 |
| P47376 | Ribonuclease Y, RNase Y, EC 3.1.-.- | Mycoplasma genitalium | LLKNQNFTESNK | 3 Deam (NQ) | 3 | 1437.70 | -3.26 | 166250 | 63 |
| P50620 | Ribonucleoside-diphosphate reductase subunit alpha | Bacillus subtilis | SQNQVPKWIQLNNEIMIQK | 3 Deam (NQ) | 3 | 2313.17 | 1.77 | 958100 | 44 |
| P50736 | Uncharacterized protein YpdA | Bacillus subtilis | IQALSYYR | Deam (NQ),O-2H (Y) | 2 | 1027.50 | -3.11 | 71202 | 74 |
| P50739 | Spore cortex-lytic enzyme, SCLE | Bacillus subtilis | EYVMEQLNK | Ox (M),Deam (NQ),Iodination | 2 | 1295.42 | 2.76 | 93670 | 44 |
| P57432 | 3-oxoacyl-[acyl-carrier-protein] reductase FabG | Buchnera aphidicola subsp. Acyrthosiphon pisum | INDYLEGNGFGFVLNLK | Deam (NQ),diOx Y | 2 | 1944.95 | -0.69 | 774140 | 66 |
| P57515 | Thymidylate synthase, TS, TSase | Buchnera aphidicola subsp. Acyrthosiphon pisum | VGNQKKDR | 2 Acetyl (K),2 Deam (NQ) | 2 | 1029.51 | 1.97 | 519140 | 78 |
| P72242 | Pectate lyase, PL | Pseudomonas amygdali pv. lachrymans | TSDMKQRAR | Acetyl (K),Deam (NQ) | 2 | 1134.55 | 3.64 | 391790 | 87 |
| P76584 | Uncharacterized protein YphB | Escherichia coli | VSGNRFVWQGR | Deam (NQ),Ox (W) | 2 | 1321.65 | 0.80 | 418090 | 71 |
| P90897 | DEAD-box ATP-dependent RNA helicase rde-12 | Caenorhabditis elegans | GFGNNGGGSFGNPNNSYR | Deam (NQ),O-2H (Y) | 2 | 1829.74 | 1.71 | 350810 | 61 |
| P9WFJ7 | UPF0353 protein Rv1481 | Mycobacterium tuberculosis | AVYSSLQQQIGYETIKGDASVGWLR | 3 Deam (NQ) | 3 | 2771.37 | 0.55 | 215640 | 47 |
| P9WHX5 | Uncharacterized PPE family protein PPE63 | Mycobacterium tuberculosis | QIGVPDWIVGGLNNVLK | 2 Deam (NQ),Di-Ox W | 2 | 1854.97 | -3.17 | 641420 | 78 |
| Q02NN7 | Phenylalanine--tRNA ligase alpha subunitt | Pseudomonas aeruginosa | EKVQDALNAR | Glu->pyro-Glu,2 Deam (NQ) | 2 | 1126.56 | 0.80 | 617070 | 139 |
| Q03CY3 | Tryptophan synthase beta chain, | Lactobacillus paracasei | KTLNETTQQSTR | 3 Deam (NQ) | 2 | 1408.67 | 3.55 | 754770 | 76 |
| Q03FS8 | DNA polymerase III PolC-type, PolIII, | Pediococcus pentosaceus | LVSMSNVK | Ox (M),Deam (NQ) | 2 | 893.45 | -3.68 | 2858400 | 111 |
| Q09268 | Uncharacterized RING finger protein C32D5.10 | Caenorhabditis elegans | LMNQIDEIIGATSSRSDSQK | Ox (M),2 Deam (NQ) | 3 | 2210.04 | 4.30 | 510550 | 55 |
| Q09994 | Anillin-like protein 2 | Caenorhabditis elegans | LQLDEVRR | Deam (NQ) | 2 | 1028.56 | -0.52 | 2502000 | 120 |
| Q0AIH1 | DNA-directed RNA polymerase subunit alpha, RNAP subunit alpha | Nitrosomonas eutropha | RVSYTIENAR | Deam (NQ),diOx Y | 2 | 1240.60 | 0.54 | 172480 | 67 |
| Q0AWF3 | Lon protease | Syntrophomonas wolfei subsp. wolfei | QILKMPGGTMR | Acetyl (K),Ox (M),Deam (NQ) | 2 | 1289.65 | 1.11 | 124930 | 98 |
| Q0BVB3 | Phosphatidylserine decarboxylase pr | Granulibacter bethesdensis | SAQMSMVQSVK | Deam (NQ) | 2 | 1195.56 | 0.28 | 4428300 | 117 |
| Q0TQG2 | Glutamyl-tRNA reductase | Clostridium perfringens | EVVILNTCNR | 2 Deam (NQ),TriOx (C) | 2 | 1266.58 | -1.98 | 99775 | 52 |
| Q111G4 | UPF0758 protein Tery_2667 | Trichodesmium erythraeum | NRLLGTQVITIGTATETLAHPR | 2 Deam (NQ) | 3 | 2363.28 | -0.93 | 1087900 | 62 |
| Q12PZ4 | Enolase | Shewanella denitrificans | GVLNAVANINGVIK | 2 Deam (NQ) | 3 | 1382.78 | 1.01 | 2892700 | 59 |
| Q15MZ1 | Glycerol-3-phosphate acyltransferas... | Pseudoalteromonas atlantica | RRQNLIGPMLLER | Deam (NQ) | 3 | 1595.89 | -4.00 | 1329500 | 64 |
| Q18081 | Putative UDP-glucuronosyltransferas... | Caenorhabditis elegans | KEKFDAYFGEQIHLCGMGLAHLIGIK | Deam (NQ) | 3 | 2975.50 | -0.88 | 2334800 | 45 |
| Q180P8 | Nucleotide-binding protein CD630_34... | Clostridioides difficile | ENYEYEMVYLDCNDDVLLK | Deam (NQ) | 3 | 2425.03 | 3.17 | 3172000 | 55 |
| Q181C1 | Ribosomal RNA small subunit methylt... | Clostridioides difficile | TLLNSLGGLGFLNKDEIR | Acetyl (K),Deam (NQ) | 2 | 2002.07 | -4.31 | 748030 | 63 |
| Q1CUF5 | Elongation factor 4 | Helicobacter pylori | RGIQEKMEYLNQSR | 2 Deam (NQ) | 3 | 1752.85 | -0.24 | 129880 | 60 |
| Q1GS43 | Protein translocase subunit SecA | Sphingopyxis alaskensis | KQVVEYDNVMNDQR | Deam (NQ),Ox Y | 2 | 1753.79 | -1.37 | 475580 | 48 |
| Q1IKF4 | UPF0502 protein Acid345_3645 | Koribacter versatilis | AEIETLKEQVK | Acetyl (K),Deam (NQ) | 2 | 1329.70 | 0.70 | 6827000 | 136 |
| Q1ILZ0 | Dihydroxy-acid dehydratase | Koribacter versatilis | EAGGTPMEFNTVSISDGITMGSEGMK | Glu->pyro-Glu,Deam (NQ) | 3 | 2628.14 | 0.62 | 875100 | 52 |
| Q1LTL1 | 3-phosphoshikimate 1-carboxyvinyltr... | Baumannia cicadellinicola subsp. Homalodisca coagulata | SLVAALCLQNKNIIITGDKR | 3 Deam (NQ) | 3 | 2229.20 | 2.17 | 1555100 | 71 |
| Q1MFL8 | Aliphatic sulfonates import ATP-bin... | Rhizobium leguminosarum bv. viciae | ISMQELINR | 2 Deam (NQ),di-Ox (M) | 2 | 1136.54 | 1.55 | 263070 | 48 |
| Q21029 | Mitogen-activated protein kinase ki... | Caenorhabditis elegans | WGPMNENAMNYYGK | Deam (NQ),O-2H (W) | 2 | 1688.66 | -3.69 | 920980 | 50 |
| Q21MS7 | LPS-assembly protein LptD | Saccharophagus degradans | AQLDGNVQISQGAR | 4 Deam (NQ) | 2 | 1459.68 | -1.78 | 4583300 | 73 |
| Q250N6 | 30S ribosomal protein S7 | Desulfitobacterium hafniense | IAGELMDAANNTGGSIK | Ox (M),2 Deam (NQ) | 2 | 1678.77 | -4.33 | 353110 | 61 |
| Q27355 | Transcription factor lin-26 | Caenorhabditis elegans | LSNNKFNQMLSK | Ox (M),Deam (NQ) | 2 | 1439.71 | -2.04 | 1413600 | 106 |
| Q2G7S5 | 1,4-alpha-glucan branching enzyme G... | Novosphingobium aromaticivorans | GVHFAVWAPNAR | Deam (NQ),Ox (W) | 2 | 1340.66 | 3.03 | 111250000 | 105 |
| Q2JW63 | DNA ligase | Synechococcus sp. | EWQERLWR | Glu->pyro-Glu,Deam (NQ) | 2 | 1184.57 | -3.67 | 13229000 | 102 |
| Q2NQM5 | 50S ribosomal protein L2 | Sodalis glossinidius | SAGAYVQIVAR | Deam (NQ),O-2H (Y) | 2 | 1148.58 | 0.22 | 294170 | 76 |
| Q2RM40 | Protein-arginine kinase | Moorella thermoacetica | LLNQLMVRIQPAFLQFSAGK | Ox (M),2 Deam (NQ) | 3 | 2291.23 | 1.72 | 9491800 | 49 |
| Q2RRX2 | Protein-glutamate methylesterase/pr... | Rhodospirillum rubrum | IAGQLNAWMSR | Ox (M),2 Deam (NQ),O-2H (W) | 2 | 1277.57 | -3.65 | 1863700 | 64 |
| Q2Y6B3 | GTP cyclohydrolase 1 | Nitrosospira multiformis | MDSNDSEFGIGDWQR | Ox (M),2 Deam (NQ),O-2H (W) | 2 | 1787.66 | 1.89 | 2393300 | 61 |
| Q3ALZ3 | 60 kDa chaperonin 1 | Synechococcus sp. | QVCEFDNPLILLTDR | Deam (NQ),TriOx (C) | 2 | 1880.88 | 2.30 | 353570 | 48 |
| Q3ATN3 | Alanine--tRNA ligase | Chlorobium chlorochromatii | ELQQIRQLLK | 3 Deam (NQ) | 2 | 1270.71 | -0.01 | 2276600 | 117 |
| Q3J0T3 | Methionine--tRNA ligase | Rhodobacter sphaeroides | GAEDVRYVQFMGK | Deam (NQ),diOx Y | 2 | 1531.70 | -2.74 | 503250 | 74 |
| Q3KKZ6 | tRNA modification GTPase MnmE | Chlamydia trachomatis serovar A | WLNTTQLGK | 2 Deam (NQ),Di-Ox W | 2 | 1093.53 | 1.47 | 372500 | 54 |
| Q3KMC2 | Recombination protein RecR | Chlamydia trachomatis serovar A | QEFSYLPIK | Deam (NQ),O-2H (Y) | 2 | 1138.55 | -2.20 | 457140 | 92 |
| Q3KN03 | Isoleucine--tRNA ligase | Chlamydia trachomatis serovar A | QPLQNVYIVGSK | Deam (NQ),O-2H (Y) | 2 | 1359.70 | 3.84 | 262210 | 69 |
| Q44581 | Nickel-cobalt-cadmium resistance pr... | Alcaligenes xylosoxydans xylosoxydans | IAVGLYQK | Deam (NQ),Ox Y | 2 | 907.50 | 0.88 | 484170 | 65 |
| Q4A5F3 | Lysine--tRNA ligase | Mycoplasma synoviae | LNINPFEKIEKNLNYVYSK | 2 Deam (NQ),2 Di-iodination | 4 | 2830.79 | -2.67 | 25978000 | 41 |
| Q4JV48 | Ribosome maturation factor RimP | Corynebacterium jeikeium | HWVRNIGR | Deam (NQ),Trp->Kynurenine | 2 | 1041.55 | -1.76 | 755740 | 53 |
| Q4UL51 | Translation initiation factor IF-2 | Rickettsia felis | VEASAKTVQNNEDIQPQTSKK | Acetyl (K),2 Deam (NQ) | 3 | 2358.16 | 0.24 | 176270 | 44 |
| Q52994 | Protein nfe2 | Rhizobium meliloti | FIEQNNQRLAPK | 2 Deam (NQ) | 3 | 1458.75 | 4.35 | 3461700 | 88 |
| Q5FMA9 | Peptidyl-tRNA hydrolase | Lactobacillus acidophilus | DSQYLMNK | Ox (M),Deam (NQ),Iodination | 2 | 1140.33 | 2.65 | 149290 | 42 |
| Q5GTF2 | Elongation factor P | Wolbachia sp. subsp. Brugia malayi | VVTYQDKIIFAHVPDYVR | Deam (NQ),Iodination,Di-iodination | 3 | 2540.83 | 3.61 | 250920 | 42 |
| Q5HX70 | GTPase Obg | Campylobacter jejuni | NNKKILTI | Acetyl (K),2 Deam (NQ) | 2 | 986.56 | 0.44 | 802770 | 57 |
| Q5KUJ0 | ATP synthase subunit delta | Geobacillus kaustophilus | MNQEVIAK | 2 Deam (NQ) | 1 | 933.45 | -1.31 | 638690 | 52 |
| Q5KVM7 | Lipoyl synthase | Geobacillus kaustophilus | LNTNENYIGLKK | Deam (NQ),O-2H (Y) | 2 | 1420.72 | 2.83 | 2966100 | 96 |
| Q5M243 | Energy-coupling factor transporter ... | Streptococcus thermophilus | EMVSRVQEALSFVGMMDFKDR | 2 Ox (M),Deam (NQ) | 3 | 2507.15 | 2.60 | 516580 | 47 |
| Q5M648 | Holliday junction resolvase RecU | Streptococcus thermophilus | MVNYPHQISR | 2 Deam (NQ),di-Ox (M) | 2 | 1277.57 | -0.34 | 1835200 | 70 |
| Q5NIK2 | ATP synthase epsilon chain | Francisella tularensis subsp. tularensis | AENLNQAEAEKAR | Acetyl (K),3 Deam (NQ) | 2 | 1487.67 | -1.06 | 172280 | 52 |
| Q5NZH5 | 30S ribosomal protein S2 | Aromatoleum aromaticum | TMGKYNEAMNYVR | Ox (M),Deam (NQ),2 Di-iodination | 3 | 2096.28 | -2.08 | 1617100 | 41 |
| Q5ZVG6 | Pimeloyl-[acyl-carrier protein] met... | Legionella pneumophila subsp. pneumophila | LAIEEPARVQYLINITSSPR | Deam (NQ),Ox Y | 3 | 2286.22 | 1.97 | 569380 | 51 |
| Q64X24 | Dephospho-CoA kinase | Bacteroides fragilis | DNASCEQIMQRIRSQMSDEEK | 2 Ox (M),Deam (NQ) | 3 | 2587.09 | -1.22 | 3651200 | 45 |
| Q65V34 | Thymidylate kinase | Mannheimia succiniciproducens | TAVKNWVNSISL | Deam (NQ),Di-Ox W | 2 | 1363.70 | -0.33 | 662910 | 69 |
| Q67Q27 | Isoleucine--tRNA ligase | Symbiobacterium thermophilum | NTLRYLLGNLYDFNPDTDMVER | 2 Deam (NQ),di-Ox (M) | 3 | 2692.23 | -2.17 | 616940 | 41 |
| Q6FD45 | UPF0102 protein ACIAD1132 | Acinetobacter baylyi | PDMKPVQDIHAHHLGK | Deam (NQ),di-Ox (M) | 3 | 1854.90 | 1.54 | 195850 | 48 |
[truncated: 31,484 more chars]
